# A Comprehensive CYP2D6 Drug–Drug–Gene Interaction Network for Application in Precision Dosing and Drug Development

**DOI:** 10.1002/cpt.3604

**Published:** 2025-02-14

**Authors:** Simeon Rüdesheim, Helena Leonie Hanae Loer, Denise Feick, Fatima Zahra Marok, Laura Maria Fuhr, Dominik Selzer, Donato Teutonico, Annika R. P. Schneider, Juri Solodenko, Sebastian Frechen, Maaike van der Lee, Dirk Jan A. R. Moes, Jesse J. Swen, Matthias Schwab, Thorsten Lehr

**Affiliations:** ^1^ Clinical Pharmacy Saarland University Saarbrücken Germany; ^2^ Dr. Margarete Fischer‐Bosch‐Institute of Clinical Pharmacology Stuttgart Germany; ^3^ Drug Metabolism and Pharmacokinetics Sanofi R&D Frankfurt am Main Germany; ^4^ Translational Medicine & Early Development Sanofi R&D Vitry‐sur‐Seine France; ^5^ Bayer AG, Pharmaceuticals, Research & Development Model‐Informed Drug Development Leverkusen Germany; ^6^ Department of Clinical Pharmacy & Toxicology Leiden University Medical Center Leiden The Netherlands; ^7^ Departments of Clinical Pharmacology, Pharmacy and Biochemistry University of Tübingen Tübingen Germany; ^8^ Cluster of Excellence iFIT (EXC2180) “Image‐guided and Functionally Instructed Tumor Therapies” University of Tübingen Tübingen Germany

## Abstract

Conducting clinical studies on drug–drug‐gene interactions (DDGIs) and extrapolating the findings into clinical dose recommendations is challenging due to the high complexity of these interactions. Here, physiologically‐based pharmacokinetic (PBPK) modeling networks present a new avenue for exploring such complex scenarios, potentially informing clinical guidelines and handling patient‐specific DDGIs at the bedside. Moreover, they provide an established framework for drug–drug interaction (DDI) submissions to regulatory agencies. The cytochrome P450 (CYP) 2D6 enzyme is particularly prone to DDGIs due to the high prevalence of genetic variation and common use of CYP2D6 inhibiting drugs. In this study, we present a comprehensive PBPK network covering CYP2D6 drug–gene interactions (DGIs), DDIs, and DDGIs. The network covers sensitive and moderate sensitive substrates, and strong and weak inhibitors of CYP2D6 according to the United States Food and Drug Administration (FDA) guidance. For the analyzed CYP2D6 substrates and inhibitors, DD(G)Is mediated by CYP3A4 and P‐glycoprotein were included. Overall, the network comprises 23 compounds and was developed based on 30 DGI, 45 DDI, and seven DDGI studies, covering 32 unique drug combinations. Good predictive performance was demonstrated for all interaction types, as reflected in mean geometric mean fold errors of 1.40, 1.38, and 1.56 for the DD(G)I area under the curve ratios as well as 1.29, 1.43, and 1.60 for DD(G)I maximum plasma concentration ratios. Finally, the presented network was utilized to calculate dose adaptations for CYP2D6 substrates atomoxetine (sensitive) and metoprolol (moderate sensitive) for clinically untested DDGI scenarios, showcasing a potential clinical application of DDGI model networks in the field of model‐informed precision dosing.

**Study Highlights**

**WHAT IS THE CURRENT KNOWLEDGE ON THE TOPIC?**

The cytochrome P450 (CYP) 2D6 enzyme, responsible for the metabolism of 20%–25% of clinically used drugs, is particularly prone to drug–drug–gene interactions (DDGIs) due to the high prevalence of structural and allelic variants as well as its propensity to be affected by enzyme inhibition during drug–drug interactions (DDIs).

**WHAT QUESTION DID THIS STUDY ADDRESS?**

This study presents the development of a comprehensive physiologically‐based pharmacokinetic (PBPK) CYP2D6 DD(G)I modeling network comprising various important CYP2D6 victim and perpetrator drug models. For selected CYP2D6 victim drugs, clinically untested DDGI scenarios were simulated, and model‐based dose adjustments were calculated, following the matching‐exposure principle.

**WHAT DOES THIS STUDY ADD TO OUR KNOWLEDGE?**

The newly established CYP2D6 DDGI network can predict interactions for a wide range of drugs, providing reliable forecasts for tested DDGIs and predictions of untested scenarios. This enhances our ability to make informed decisions regarding dose adjustments for untested DDGI scenarios in clinical settings, particularly for patients with varying CYP2D6 enzyme activity.

**HOW MIGHT THIS CHANGE CLINICAL PHARMACOLOGY OR TRANSLATIONAL SCIENCE?**

The approach presented in this study facilitates more precise model‐informed dosing strategies considering both individual genetic profiles as well as multiple drug interactions. Additionally, it presents a robust framework for simulating and understanding complex DDGIs, supplementing knowledge gained from clinical DDGI trials and potentially accelerating drug development and regulatory processes.

Drug–drug interactions (DDIs) and drug–gene interactions (DGIs) are key drivers of adverse drug reactions (ADRs), significantly contributing to hospitalizations and in‐hospital mortality.^1^, ^2^ DDIs arise when the perpetrator drug alters the pharmacokinetics (PK) or pharmacodynamics of the victim drug. PK interactions frequently involve cytochrome P450 (CYP) isozymes or important drug‐transporting proteins, such as P‐glycoprotein (P‐gp), which are essential in the absorption, distribution, metabolism, and excretion (ADME) processes of many clinically used drugs.^3^, ^4^ In the case of DGIs, genetic variation of specific pharmacogenes can result in varying activities of the affected enzyme or transporter influencing a drug's PK.

In clinical practice, DDIs and DGIs often occur simultaneously in the form of drug–drug–gene interactions (DDGIs).^5^ However, separate alerts arise in electronic prescribing systems for DDIs and DGIs, aggravating clinical decision making. Here, CYP2D6 is one of the most susceptible enzymes to DDGIs.^4^, ^6^ This liability arises from two primary factors: the relatively high prevalence of structural and allelic variants of the *CYP2D6* gene which can have substantial effects on enzymatic activity, and the enzyme's propensity for DDIs due to its role in metabolizing an estimated 20%–25% of clinically used drugs.^4^, ^7^ Genetic variants in the *CYP2D6* gene result in different metabolizer phenotypes, ranging from poor metabolizers, carrying two loss‐of‐function alleles, to ultrarapid metabolizers, typically possessing more than two active *CYP2D6* alleles.^3^, ^8^ In addition to the *CYP2D6* genotype, administration of strong CYP2D6 inhibitors, such as paroxetine or bupropion, can markedly decrease CYP2D6 activity, often resulting in a change of the apparent phenotype commonly referred to as “phenoconversion”.^9^ To improve the safety and efficacy of CYP2D6 substrates, it is essential to consider the influence of CYP2D6 activity and concomitant medications on the PK of substrates not only individually but also in their combined effect.

Assessing DDGIs in clinical settings is inherently challenging due to the complexity of simultaneously occurring interactions given the vast number of potential combinations of genetically determined activity levels and relevant drug combinations. Thus, DDGIs pose a significant obstacle in clinical research and practice as it is impossible to perform formal studies covering all combinations.^10^ Additionally, applying the findings of published clinical DDGI trials to the real world is challenging, due to the limited transferability arising from the often narrow scope of these studies. These studies typically focus on a small selection of drugs and genetic variations and tend to include predominantly young, healthy male participants.^5^ Here, physiologically‐based pharmacokinetic (PBPK) modeling has become an indispensable tool in model‐informed drug discovery and development (MID3),^11^ providing a suitable approach to investigate and predict DDGIs.^5^ PBPK models offer a mechanistic framework, integrating system‐dependent parameters (e.g., age, sex, ethnicity, body weight, organ volumes, and perfusion rates) and drug‐dependent parameters (e.g., solubility, permeability, transport, protein binding, and metabolic pathways). Moreover, they allow the detailed implementation of DDI processes. Hence, PBPK modeling can be used to conduct virtual clinical DD(G)I trials, enabling the prediction of DD(G)I scenarios prior to conducting a dedicated clinical trial, potentially reducing time, cost and risks associated with such studies. Regulatory authorities, such as the US Food and Drug Administration (FDA) and the European Medicines Agency (EMA) advocate for the utilization of PBPK modeling to address various research challenges, such as the investigation of DDI or organ impairment, and regularly publish guidelines to support its application.^12^, ^13^ Examining and predicting the DDI potential of investigational drugs requires a well‐established library of PBPK models for index perpetrator and victim drugs. These libraries can facilitate the assessment of the DDI potential, and therefore, accelerate the process of MID3.^14^ Here, the FDA table of Drug Development and Drug Interactions provides comprehensive guidance on the selection of substrates, inhibitors, and inducers for concomitant use in clinical DDI studies and drug labeling.^15^

The objectives of this work were to (i) establish and evaluate a comprehensive CYP2D6 DDGI network by extending and combining previously published PBPK DD(G)I networks and models of CYP2D6 substrates and inhibitors, (ii) to apply the network to predict a selection of not yet clinically studied DD(G)I scenarios, (iii) to derive model‐based dose adjustments for these DD(G)I scenarios. All model files will be made publicly available in the Clinical Pharmacy Saarland University PBPK model library (http://models.clinicalpharmacy.me).

## METHODS

### Software

PBPK modeling, DD(G)I simulations and simulations of sensitivity analyses were performed using PK‐Sim® and MoBi® (version 11, OSP Suite, http://www.open‐systems‐pharmacology.org). Plasma concentration–time profiles were digitized from the published literature using Engauge Digitizer 10.12 (© M. Mitchell, https://markummitchell.github.io/engauge‐digitizer). The R programming language version 4.3.0 was used for model evaluation purposes, including the generation of plots and the calculation of pharmacokinetic parameters and statistics.

### DD(G)I network development

A comprehensive literature review in PubMed was undertaken to gather clinical DD(G)I studies, adhering to the following criteria: (i) availability of corresponding PBPK models developed with the OSP suite for the compounds investigated in the DD(G)I studies; (ii) DD(G)Is involving CYP2D6 victim drugs; (iii) availability of plasma concentration–time profiles of the victim drug, preferably measured during as well as prior to perpetrator co‐administration, and (iv) stratification by CYP2D6 phenotype, genotype or activity score. Afterward, the CYP2D6 DD(G)I network was established by linking and combining published PBPK models with pre‐existing networks^16^, ^17^, ^18^ to simulate the DD(G)I scenarios collected from the literature. In addition, a new PBPK model for the CYP2D6 substrate desipramine and its metabolite 2‐hydroxydesipramine was developed, as described in **Section S1**.

For each study cohort, a virtual population of 1,000 individuals was created based on the reported study population characteristics. Specifically, age, weight, and height ranges, as well as ethnicity were considered to vary organ and tissue volumes and perfusion rates according to the PK‐Sim® database. Additional variability was implemented by varying expressions of metabolizing enzymes, transport proteins, and protein binding partners according to the PK‐Sim® expression database as well as the parameters reported in **Table** **S8**. Relative expressions of metabolizing enzymes, transport proteins, and protein binding partners used for the creation of virtual populations are given in **Tables** **S9**, **S10**. CYP2D6 activity levels were adjusted according to the respective study report. If no information on the CYP2D6 phenotype/genotype of a study population was available, a CYP2D6 normal metabolizer phenotype was assumed, as it is the most commonly reported phenotype in the included clinical studies.

### Effect model evaluation

To evaluate the effects of DGIs, DDIs, and DDGIs, population predictions of victim drug concentrations were plotted alongside their respective observed plasma concentrations alone and during perpetrator co‐administration. Furthermore, predicted compared with observed DD(G)I ratios were calculated according to Eq. 1 and visually compared in goodness‐of‐fit plots.

(1) $$\text{Effect}\;\mathrm{PK}\;\text{ratio}=\frac{{\mathrm{PK}}_{\text{Effect}}}{{\mathrm{PK}}_{\text{Reference}}}$$

where effect PK ratio = ratio of the PK parameter (area under the plasma concentration–time curve from the time of the first measurement to the time of the last measurement (AUC_last_) or maximum plasma concentration (*C*_max_)) for the investigated effect (variant CYP2D6 activity and/or drug co‐administration), PK_Effect_ = value of the PK parameter for the investigated effect and PK_Reference_ = value of the PK parameter for the respective reference (i.e., normal CYP2D6 activity and/or victim drug alone). Normal CYP2D6 activity was defined as the normal metabolizer phenotype or an activity score of 2 if genotypes or activity scores were reported.

DD(G)I ratios were assessed according to limits proposed by Guest *et al*. including 20% variability.^19^ Additionally, geometric mean fold errors (GMFEs) of DD(G)I AUC_last_ and *C*_max_ ratios were calculated according to Eq. 2.

(2) $$\text{GMFE}=10^{x};x=\frac{\sum_{i=1}^{n}\;\left|l{\mathrm{og}}_{10}\;\left(\frac{\hat{\rho_{i}}}{\rho_{i}}\right)\right|\;}{n}$$

where $\hat{\rho_{i}}$ = predicted AUC_last_ or *C*_max_ value of study *i*, $\rho_{i}$ = corresponding observed AUC_last_ or *C*_max_ value of study *i*, *n* = number of studies.

### Model‐informed dose adaptations

The final DDGI network was used to simulate drug exposure for the sensitive CYP2D6 substrate atomoxetine and the moderately sensitive CYP2D6 substrate metoprolol in untested D(D)GI scenarios. The analysis evaluated the exposures of both victim drugs during the co‐administration of one or multiple perpetrator drugs (CYP2D6 and CYP3A4 inhibitors), considering the range of CYP2D6 activity scores evaluated during DD(G)I network development, that is, from 0 (poor metabolizer) to 3 (ultrarapid metabolizer). For each activity score group, a virtual European male, aged 30, weighing 73 kg and measuring 176 cm of height, was created based on the International Commission on Radiological Protection (ICRP) database for use in the simulations. CYP2D6 activity for the different virtual individuals was adjusted based on the respective activity score.^20^ Administration protocols for the victim and perpetrator drugs were simulated according to the standard doses stated in the respective prescribing information, as listed in **Section** **S8**. Subsequently, dose adjustments were performed for the simulated D(D)GI scenarios matching the exposure of victim monotherapy for activity score 2 (normal metabolizer, wild‐type). Since simulated DD(G)I scenarios were expected to typically result in dose reductions, victim doses were adjusted more finely, using 1% steps for doses <100% of the original. For doses exceeding 100%, adjustments were simulated in 10% steps to optimally align the steady‐state AUC (AUC_ss_) with the reference exposure following the matching‐exposure principle.

## RESULTS

### DD(G)I network development

**Figure 1:**
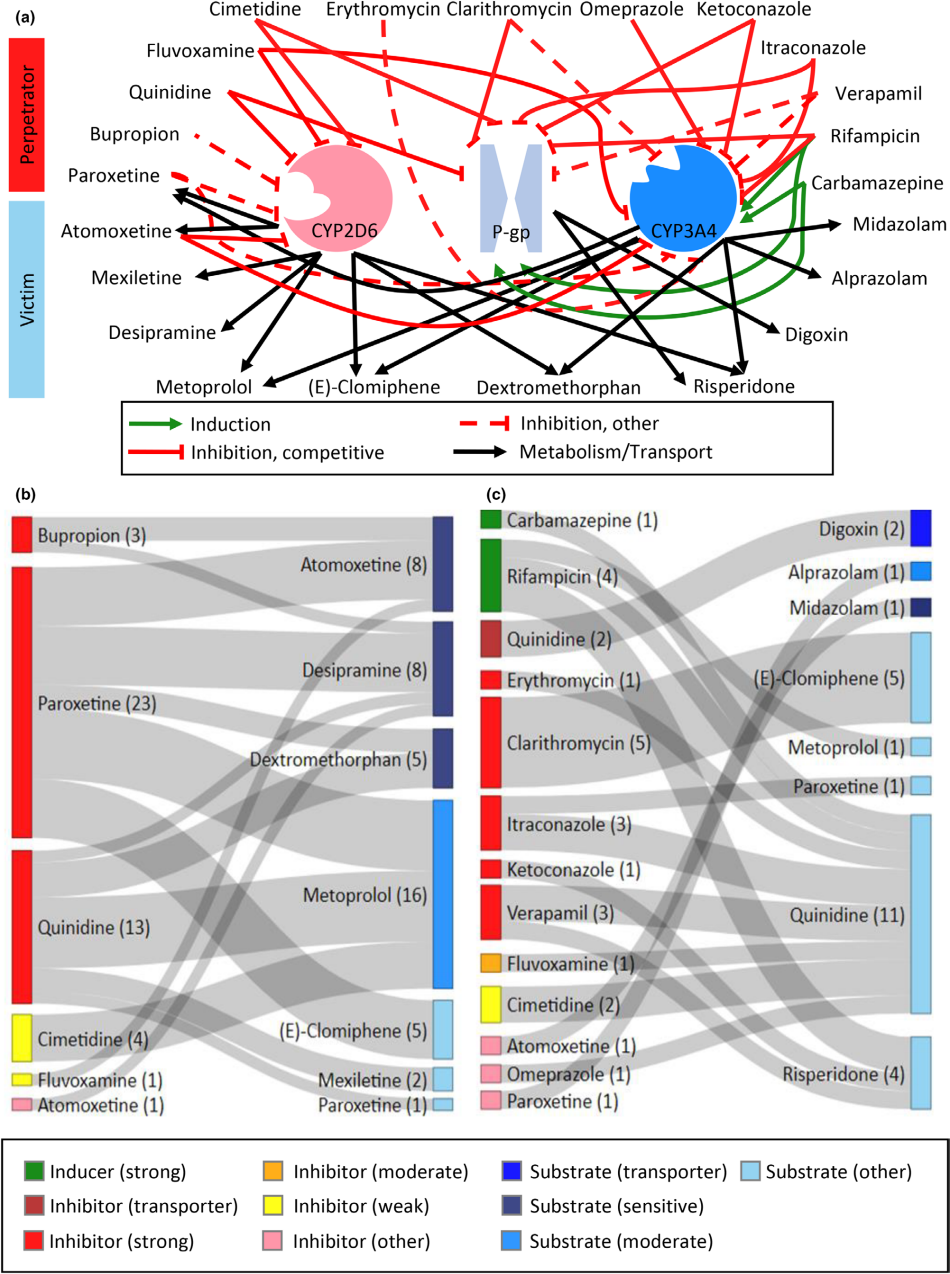
(**a**) Physiologically‐based pharmacokinetic drug–drug–gene interaction network. Schematic illustration of the modeled interactions of CYP2D6 perpetrator and victim drugs. Black arrows indicate metabolism or transport, green arrows indicate induction, red solid lines indicate competitive inhibition, red dashed lines down‐regulation (bupropion), noncompetitive inhibition (verapamil P‐gp inhibition), or mechanism‐based inactivation (others). (**b, c**) Drug–drug‐(gene) interaction matrix for modeled interactions mediated by (**a**) CYP2D6 and (**b**) CYP3A4 and P‐gp. Colors indicate categories according to the *FDA's Examples of Drugs that Interact with CYP Enzymes and Transporter Systems*.^41^ Height of the gray ribbons indicates the number of clinical studies for the respective interaction covered by the network, numbers in brackets indicate the number of clinical interaction studies for the corresponding compound. CYP, cytochrome P450; P‐gp, P‐glycoprotein.

The presented network includes a total of 23 drugs, eight of which are CYP2D6 substrates. Of these substrates, atomoxetine, desipramine, and dextromethorphan are categorized by the FDA as sensitive substrates, and metoprolol as a moderately sensitive substrate of CYP2D6. Four drugs ((*E*)‐clomiphene, mexiletine, paroxetine, and risperidone) are currently not yet classified as substrates of CYP2D6 by the FDA but show a considerable susceptibility to CYP2D6 DDGIs.^16^, ^17^, ^20^ Additionally, to cover DD(G)Is mediated by CYP3A4 and P‐gp involving substrates or inhibitors of CYP2D6, various perpetrators and victims of CYP3A4 and P‐gp were included in the network. **Figure** **1** **a** provides an overview of the modeled DD(G)I network. **Figure** **1** **b,c** shows an overview of model compounds and drug combinations included in the network as well as their FDA substrate, inhibitor, or inducer category alongside the corresponding number of modeled clinical studies.

Previously published models of paroxetine,^20^ quinidine,^18^ dextromethorphan,^21^ (*E*)‐clomiphene,^16^ mexiletine,^17^ carbamazepine,^18^, ^22^ rifampicin,^14^ clarithromycin,^23^ erythromycin,^24^ itraconazole,^14^ ketoconazole,^25^ verapamil,^26^ omeprazole,^17^ digoxin,^23^ midazolam,^23^ alprazolam, and risperidone^20^ were used without modifications to the model structure. The bupropion model^25^ was modified to include down‐regulation and competitive inhibition of CYP2D6 by bupropion and its metabolites as described in the literature.^27^, ^28^ CYP2D6 competitive inhibition was implemented in the fluvoxamine^29^, cimetidine,^26^ and atomoxetine^20^ models using published values.^30^, ^31^, ^32^ Competitive inhibition of CYP2C19 was additionally implemented in the fluvoxamine model, as well as competitive inhibition of CYP3A4 in the atomoxetine model, both according to literature reports.^32^, ^33^ A CYP3A4‐mediated clearance of metoprolol was incorporated as a surrogate pathway in the corresponding model^34^ to account for the inducible residual metabolism of metoprolol.^35^ All parameters implemented in the respective models related to the inhibition/induction of as well as metabolism/transport by CYP2D6, CYP3A4, and P‐gp are listed in **Tables** **S11**, **S12**. Detailed results for the building and evaluation of the newly developed desipramine model are presented in **Sections** **S2** **and** **S3**.

The overall CYP2D6 DGI model performance of CYP2D6 substrates was evaluated using a total of 30 clinical DGI studies in which subjects had been stratified by CYP2D6 activity score or phenotype. The effect of varying CYP2D6 activity was assessed for 85 DGI scenarios involving 17 compounds (parent drug and respective metabolites or enantiomers), comprising atomoxetine, clomiphene (including metabolites (*E*)‐4‐hydroxy‐N‐desethylclomiphene, (*E*)‐N‐desethylclomiphene and (*E*)‐4‐hydroxyclomiphene), desipramine (including metabolite 2‐hydroxydesipramine), dextromethorphan (including metabolite dextrorphan), metoprolol (including enantiomers (*S*)‐metoprolol and (*R*)‐metoprolol, as well as their metabolite α‐hydroxymetoprolol), mexiletine, paroxetine, and risperidone (including metabolite 9‐hydroxyrisperidone).

Furthermore, DD(G)I network modeling was performed using 45 clinical DDI and seven clinical DDGI studies covering 121 DDI and 42 DDGI scenarios as well as 32 unique drug combinations. In total, six CYP2D6 perpetrator drugs were included with bupropion, paroxetine, and quinidine being categorized as strong CYP2D6 inhibitors and cimetidine and fluvoxamine as weak CYP2D6 inhibitors. Additionally, atomoxetine was included as an inhibitor of CYP2D6 as it has been reported to inhibit CYP2D6 *in vitro*.^32^ Moreover, DD(G)Is mediated by CYP3A4 and P‐gp involving substrates or inhibitors of CYP2D6 were added to the presented network due to their relevance for predicting complex DD(G)Is. For instance, the model of the CYP2D6 substrate risperidone also includes metabolism by CYP3A4 and transport via P‐gp,^36^ warranting the inclusion of CYP3A4 and P‐gp inhibitor models in the network to cover the respective DDIs. Conversely, the model of the strong CYP2D6 inhibitor paroxetine includes inhibition of CYP3A4.^20^ Therefore, the PBPK models of CYP3A4 victims were included in the network to describe the corresponding DDIs reported in the literature. Here, eight victim drugs cover one substrate of P‐gp (digoxin), one sensitive substrate of CYP3A4 (midazolam), one moderate sensitive substrate of CYP3A4 (alprazolam) as well as five currently uncategorized substrates of CYP3A4 ((*E*)‐clomiphene, metoprolol, paroxetine, quinidine, and risperidone). Perpetrators include one inhibitor of P‐gp (quinidine), five strong inhibitors of CYP3A4 (clarithromycin, erythromycin, itraconazole, ketoconazole, and verapamil), one moderate inhibitor of CYP3A4 (fluvoxamine, also a strong inhibitor of CYP2C19), one weak inhibitor of CYP3A4 (cimetidine), two strong inducers of CYP3A4 (carbamazepine and rifampicin) as well as three uncategorized inhibitors of CYP3A4 (atomoxetine, omeprazole, and paroxetine). Detailed information on all modeled DD(G)I studies is provided in **Section** **S6**.

### DGI model evaluation

**Figure 2:**
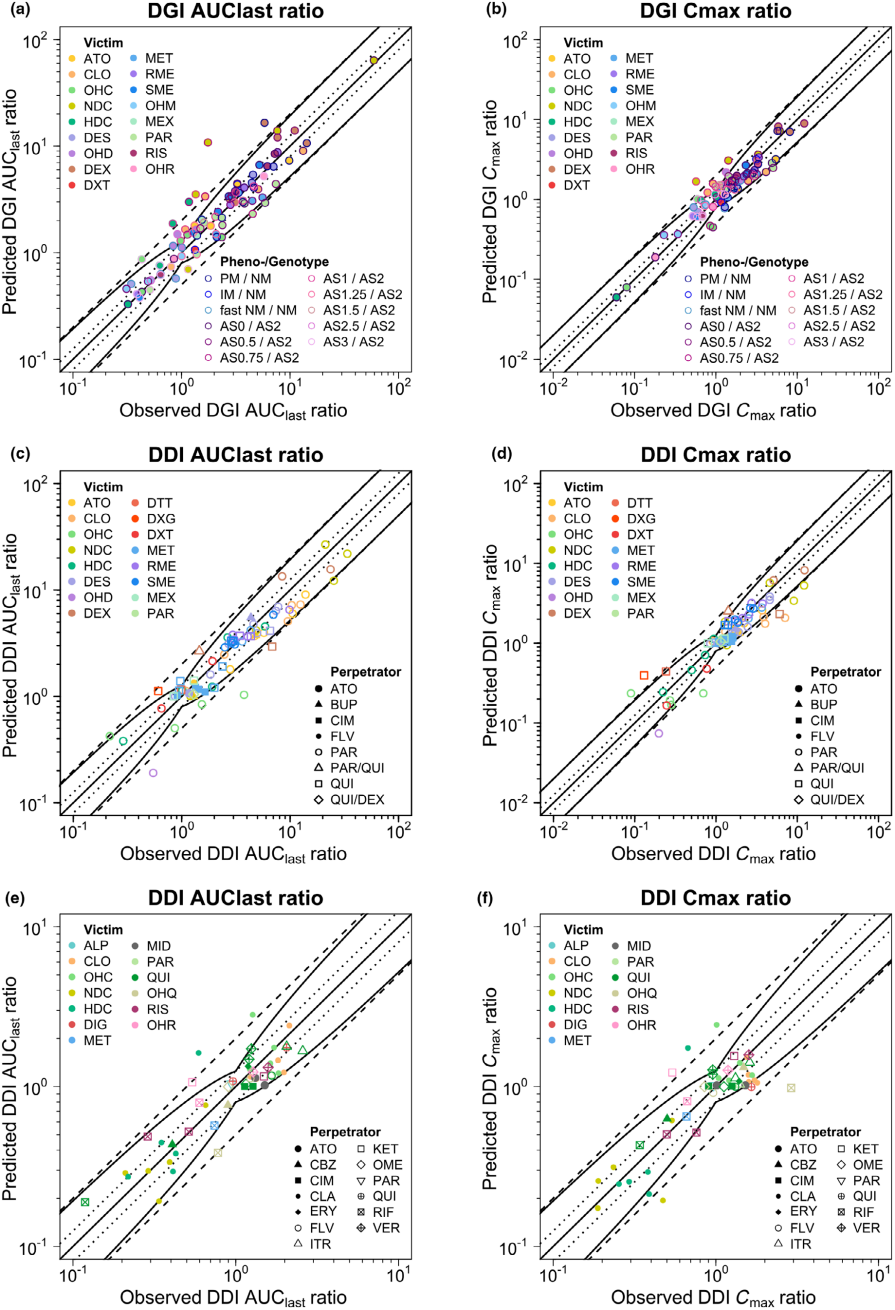
Predicted vs. observed DGI (**a**) AUC_last_ and (**b**) *C*_max_ ratios as well as DDI (**c, e**) AUC_last_ and (**d, f**) *C*_max_ ratios, stratified by (**c, d**) CYP2D6‐mediated DDIs and (**e, f**) non‐CYP2D6‐mediated DDIs, of CYP2D6 substrates included in the network. Colored symbols represent the victim drugs; their shape corresponds to the respective perpetrator and their colored borders correspond to the respective phenotype or genotype as well as the reference genotype or phenotype. The solid straight black line marks the line of identity. Curved black lines show prediction success limits according to Guest *et al*.^19^, including 1.25‐fold variability. Black dotted lines show the 1.25‐fold range, dashed black lines indicate the twofold range. ALP, alprazolam; AS, CYP2D6 activity score; ATO, atomoxetine; AUC_last_, area under the plasma concentration–time curve from the time of the first measurement to the time of the last measurement; BUP, bupropion; CBZ, carbamazepine; CIM, cimetidine; CLA, clarithromycin; CLO, (*E*)‐clomiphene; *C*_max_, maximum plasma concentration; DDI, drug–drug interaction; DES, desipramine; DEX, dextromethorphan; DGI, drug–gene interaction; DIG, digoxin; DTT, total dextrorphan; DXT, dextrorphan; ERY, erythromycin; FLV, fluvoxamine; HDC, (*E*)‐4‐hydroxy‐N‐desethylclomiphene; IM, intermediate metabolizer; ITR, itraconazole; KET, ketoconazole; MET, metoprolol racemate; MEX, mexiletine; MID, midazolam; NDC, (*E*)‐N‐desethylclomiphene; NM, normal metabolizer; OHC, (*E*)‐4‐hydroxyclomiphene; OHD, 2‐hydroxydesipramine; OHM, α‐hydroxymetoprolol; OHQ, 3‐hydroxyquinidine; OHR, 9‐hydroxyrisperidone; OME, omeprazole; PAR, paroxetine; PM, poor metabolizer; QUI, quinidine; RIF, rifampicin; RIS, risperidone; RME, (*R*)‐metoprolol; SME, (*S*)‐metoprolol; VER, verapamil.

Predicted DGI AUC_last_ and *C*_max_ ratios were in good agreement with observed DGI ratios as depicted in **Figure** **2** **a,b**. Mean GMFEs for predicted DGI AUC_last_ and *C*_max_ ratios for all CYP2D6 substrates were 1.40 and 1.29, respectively, as listed in **Table** **1**. Predicted and observed DGI AUC_last_ and *C*_max_ ratios for all studies are presented in **Section** **S5**.

**Table 1:** Summary of geometric mean fold errors (GMFEs) for DGI, DDI, and DDGI predictions

Scenario	*n*	Mean GMFE (range)	
		AUC_last_	*C*_max_
CYP2D6 DGIs	85	1.40 (1.00–6.19)	1.29 (1.00–2.95)
CYP2D6 DDIs	72	1.40 (1.00–3.64)	1.45 (1.01–3.41)
Competitive inhibitors	21	1.32 (1.04–2.33)	1.39 (1.01–3.03)
MBI/other inhibitors	51	1.43 (1.00–3.64)	1.48 (1.02–3.41)
Other DDIs	49	1.33 (1.02–2.75)	1.38 (1.00–2.96)
Competitive inhibitors	15	1.28 (1.09–1.97)	1.31 (1.00–2.27)
MBI	26	1.34 (1.02–2.75)	1.40 (1.00–2.59)
Inducers	8	1.40 (1.02–2.02)	1.41 (1.01–2.96)
All DDIs	121	1.38 (1.00–3.64)	1.43 (1.01–3.41)
CYP2D6 DDGIs	26	1.61 (1.01–3.55)	1.64 (1.05–3.71)
Competitive inhibitions	3	1.61 (1.09–1.96)	1.30 (1.17–1.40)
MBI/other inhibitors	23	1.61 (1.01–3.55)	1.68 (1.05–3.71)
Other DDGIs – MBI	16	1.49 (1.02–2.42)	1.54 (1.02–3.31)
All DDGIs	42	1.56 (1.01–3.55)	1.60 (1.02–3.71)

AUC_last_, Area under the plasma concentration–time profile from the time of the first measurement to the time of the last measurement; *C*_max_, maximum plasma concentration; DDGIs, Drug–drug–gene interactions; DDIs, drug–drug interactions; DGIs, drug–gene interactions; GMFE, geometric mean fold error; MBI, mechanism‐based inhibition; *n* = number of investigated interaction scenarios.

### DDI model evaluation

The developed network showed good predictive performance regarding DDI AUC_last_ and *C*_max_ ratios, as demonstrated in **Figure** **2** **c–f**. Mean GMFE values of 1.40 and 1.45 were obtained for the predicted DDI AUC_last_ and *C*_max_ ratios of the CYP2D6 DDIs, respectively, while for the non‐CYP2D6 DDIs, GMFE values were 1.33 and 1.38 for DDI AUC_last_ and *C*_max_ ratios, respectively. **Table** **1** shows the mean GMFE values and ranges stratified by mechanisms of interaction. Additionally, **Table** **2** shows mean GMFE values and ranges for the different DDIs grouped by CYP2D6 perpetrator and victim drugs, respectively. Plasma concentration–time profiles in semilogarithmic and linear representation as well as predicted vs. observed DDI AUC_last_ and *C*_max_ ratios of all DDI scenarios are provided in **Section** **S6**.

**Table 2:** Summary of geometric mean fold errors (GMFEs) for CYP2D6 DDI predictions by perpetrator and victim

Compound	*n*	Mean GMFE (range)	
		AUC_last_	*C*_max_
CYP2D6 perpetrators			
Atomoxetine	1	1.09	1.06
Bupropion	3	1.30 (1.27–1.36)	1.18 (1.02–1.36)
Cimetidine	4	1.24 (1.10–1.49)	1.31 (1.10–1.48)
Fluvoxamine	1	1.04	1.17
Paroxetine	48	1.44 (1.00–3.64)	1.50 (1.02–3.41)
Quinidine	15	1.37 (1.08–2.33)	1.44 (1.01–3.03)
CYP2D6 victims			
Atomoxetine	8	1.41 (1.04–1.77)	1.22 (1.02–1.43)
(*E*)‐Clomiphene	20	1.56 (1.00–3.64)	1.72 (1.04–3.41)
Desipramine	11	1.35 (1.04–2.86)	1.38 (1.06–2.68)
Dextromethorphan	8	1.62 (1.11–2.33)	1.94 (1.20–3.03)
Metoprolol	22	1.21 (1.00–1.66)	1.19 (1.01–1.48)
Mexiletine	2	1.13 (1.08–1.18)	1.19 (1.11–1.26)
Paroxetine	1	1.23	1.10

AUC_last_, Area under the plasma concentration–time profile from the time of the first measurement to the time of the last measurement; *C*_max_, maximum plasma concentration; DDIs, drug–drug interactions; GMFE, geometric mean fold error; *n* = number of investigated interaction scenarios.

### DDGI model evaluation

**Figure 3:**
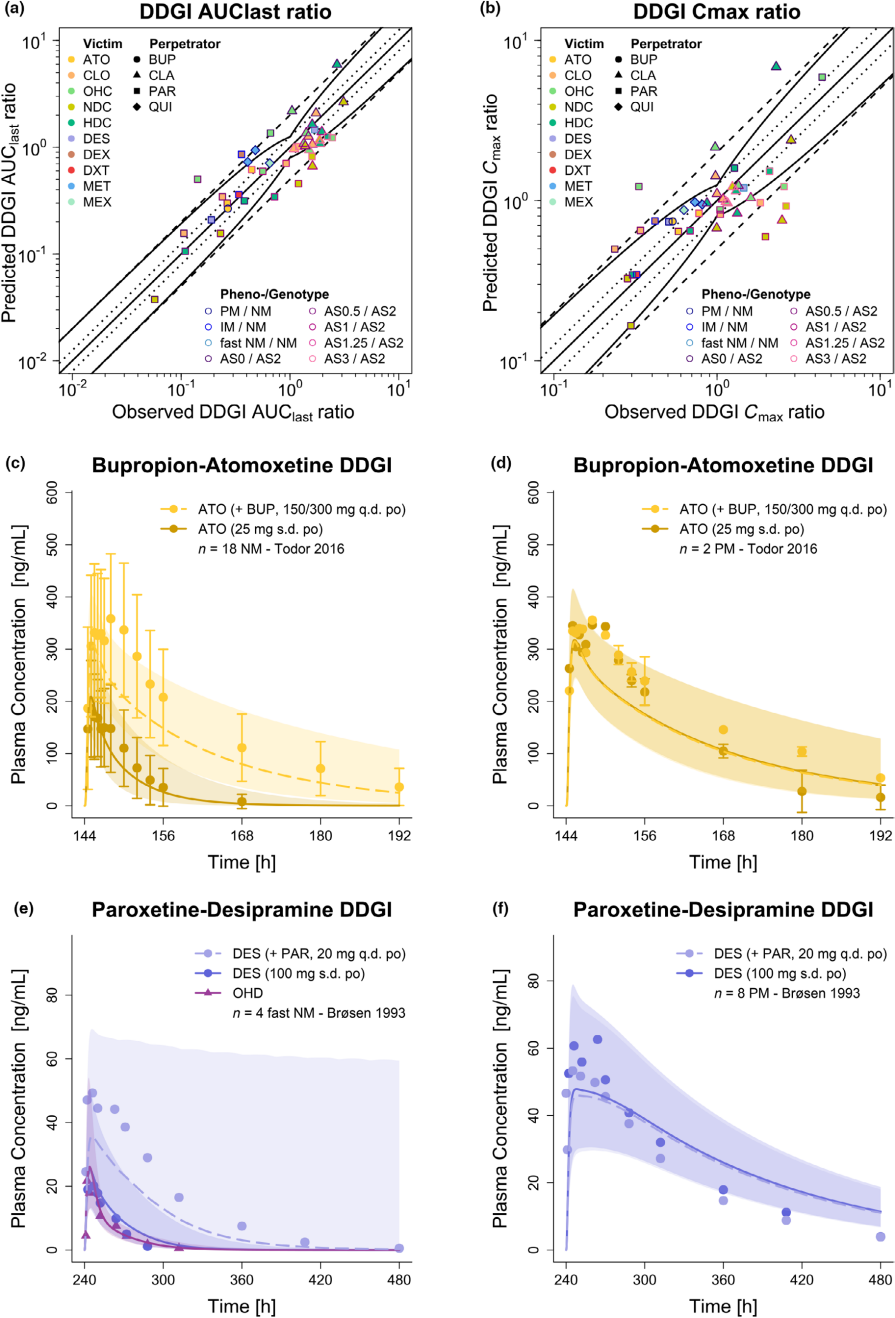
Predicted vs. observed DDGI (**a**) AUC_last_ and (**b**) *C*_max_ ratios of CYP2D6 substrates included in the network. Colored symbols represent the victim drugs; their shape corresponds to the respective perpetrator; their colored borders correspond to the respective phenotype or genotype as well as the reference genotype or phenotype. The solid straight black line marks the line of identity. Curved black lines show prediction success limits according to Guest *et al*.,^19^ including 1.25‐fold variability. Black dotted lines show the 1.25‐fold range, dashed black lines indicate the twofold range. (**c–f**) Selection of modeled DDGIs. Predicted compared with observed plasma concentration–time profiles of the respective victim drug alone and after pretreatment with and/or concomitant administration of a perpetrator drug: (**c, d**) atomoxetine with and without bupropion pretreatment in (**c**) CYP2D6 normal metabolizers and (**d**) poor metabolizers and (**e, f**) desipramine with and without paroxetine pretreatment in (**e**) fast CYP2D6 normal metabolizers^49^ and (**f**) poor metabolizers. Predicted population geometric means are shown as lines (solid: victim drug alone, dashed: victim drug during DDGI), predicted geometric standard deviations are shown as shaded areas and observed data are shown as dots (parent compound) and triangles (metabolite, if available) (± standard deviation, if reported).^43^, ^49^ AS, CYP2D6 activity score; ATO, atomoxetine; AUC_last_, area under the plasma concentration–time curve from the time of the first measurement to the time of the last measurement; b.i.d., twice daily; BUP, bupropion; CLA, clarithromycin; CLO, (*E*)‐clomiphene; *C*_max_, maximum plasma concentration; DDGI, drug–drug–gene interaction; DES, desipramine; DEX, dextromethorphan; DXT, dextrorphan; HDC, (*E*)‐4‐hydroxy‐N‐desethylclomiphene; IM, intermediate metabolizer; MET, metoprolol racemate; MEX, mexiletine; MID, midazolam; *n*, number of study participants; NDC, (*E*)‐N‐desethylclomiphene; NM, normal metabolizer; OHC, (*E*)‐4‐hydroxyclomiphene; OHD, 2‐hydroxydesipramine; OHQ, 3‐hydroxyquinidine; PAR, paroxetine; PM, poor metabolizer; po, oral; q.d., once daily; QUI, quinidine; s.d., single dose.

**Figure** **3** **a,b** shows the predicted vs. observed DDGI AUC_last_ and *C*_max_ ratios of the included DDGI scenarios, demonstrating overall good predictive performance of the network. Mean GMFE values of 1.61 and 1.64 were obtained for the predicted DDGI AUC_last_ and *C*_max_ ratios of the CYP2D6 DDGIs, respectively, while for the non‐CYP2D6 DDGIs values of 1.49 and 1.54 were calculated. **Table** **1** lists the mean GMFE values and range stratified by mechanism of interaction. A selection of simulated DDGIs involving the CYP2D6 victim drugs atomoxetine and desipramine as well as perpetrator drugs bupropion and paroxetine for different CYP2D6 phenotypes is presented as plasma concentration–time profiles in **Figure** **3** **c–f**. Plasma concentration–time profiles in semilogarithmic and linear representation as well as predicted vs. observed DDGI AUC_last_ and *C*_max_ ratios of all DDGI scenarios can be found in **Sections** **S6** **and** **S7**.

### Model‐informed dose adaptations

**Figure 4:**
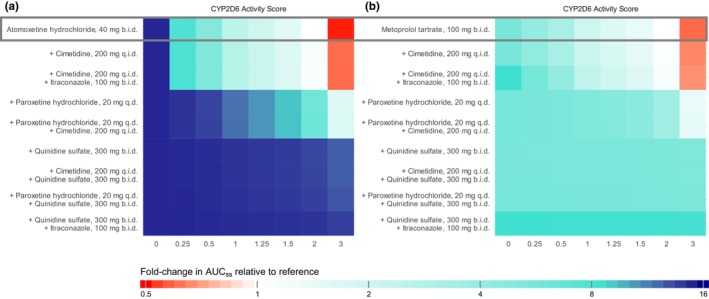
Fold‐change in AUC_ss_ relative to the reference AUC_ss_ (activity score 2, no DDI) across different D(D)GI scenarios before dose adaptations for (**a**) atomoxetine and (**b**) metoprolol. Colors indicate the extent and direction of the deviation from the reference AUC_ss_. AUC_ss_, area under the concentration–time curve during steady state; b.i.d., twice a day; D(D)GI, drug(−drug)–gene interaction; q.d., once daily; q.i.d., four times a day.

**Figure 5:**
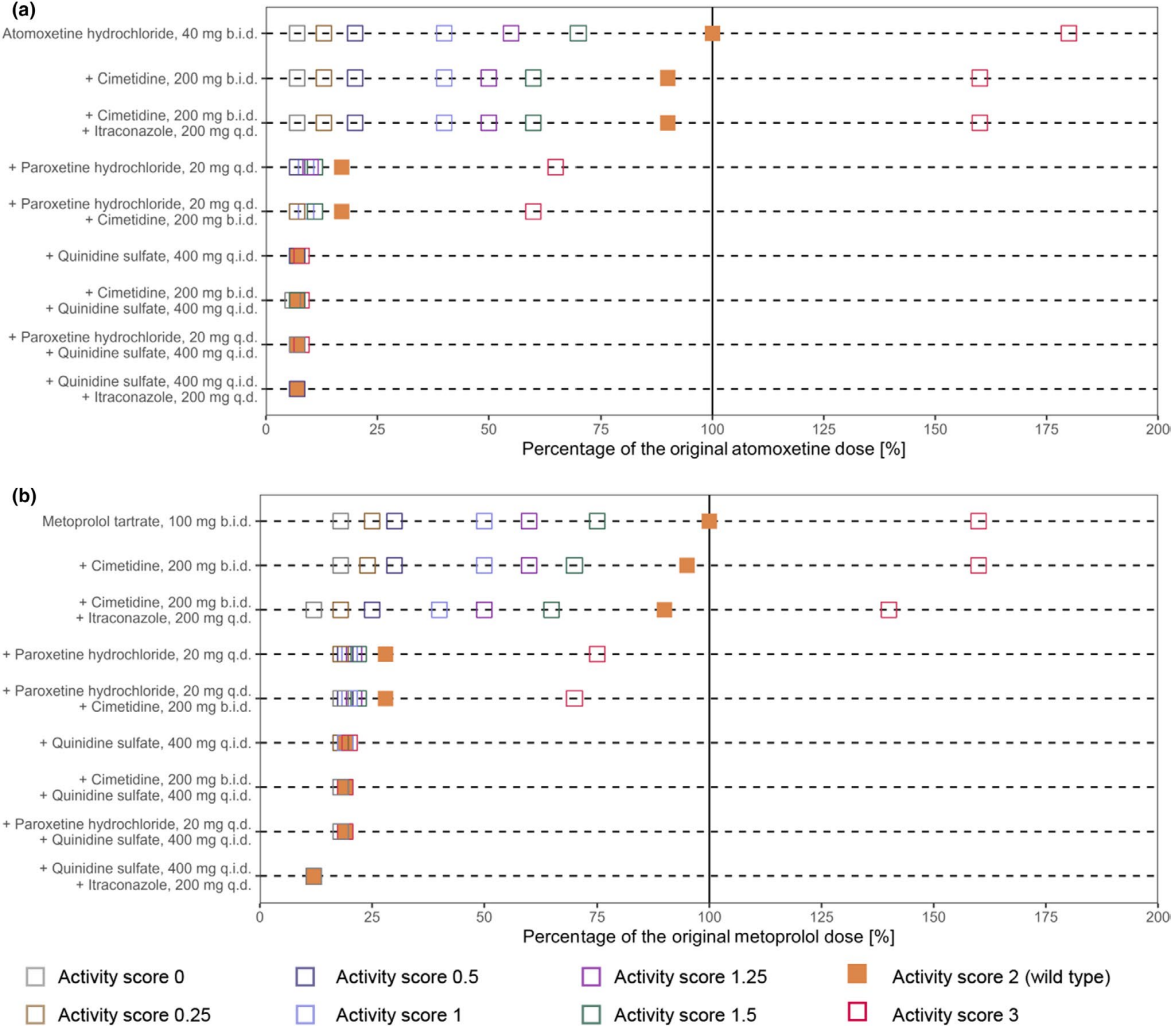
Overview of model‐based dose adaptations for (**a**) atomoxetine and (**b**) metoprolol within single and multiple D(D)GI scenarios based on the exposure matching principle, where points and squares show the percentage of the original dose that match the PBPK simulated monotherapy AUC_ss_ for activity score 2. Colored symbols depict dose reductions for the different activity scores. AUC_ss_, area under the concentration–time curve during steady state; b.i.d., twice a day; D(D)GI, drug(−drug)–gene interaction; PBPK, physiologically‐based pharmacokinetic; q.d., once daily; q.i.d., four times a day.

The final network was applied to simulate D(D)GI scenarios involving the CYP2D6 victim drugs atomoxetine and metoprolol with various combinations of strong and weak inhibitors of CYP2D6 and CYP3A4 across different CYP2D6 activity scores. Paroxetine and quinidine were selected as CYP2D6 perpetrators and itraconazole as CYP3A4 inhibitor. Cimetidine was selected as a weak inhibitor of both CYP2D6 and CYP3A4. Moreover, activity scores of 0, 0.25, 0.5, 1, 1.25, 1.5, 2 (wild‐type) and 3 were assumed for simulated D(D)GI scenarios. Model exposure simulations revealed that co‐administration of the perpetrator drugs may result in AUC_ss_ increases of up to 14.8‐fold and 8.5‐fold as well as reductions of up to 0.5‐fold and 0.6‐fold of the reference AUC_ss_ for atomoxetine and metoprolol, respectively. Fold changes in AUC_ss_ for the different scenarios compared with the reference AUC_ss_ are presented in **Figure** **4**. All simulated D(D)GI scenarios including administration protocols of the respective victim and perpetrator drugs are listed in **Figure** **5**. Simulations to match the respective model‐simulated monotherapy AUC_ss_ for the CYP2D6 activity score 2 yielded a dose range of 2.4–72 mg (6%–180% of the original dose) for atomoxetine and 12–160 mg (12%–160% of the original dose) for metoprolol (**Figure** **5**). As shown in **Figures** **S68** **and** **S69**, dose adjustments resulted in simulated exposures well within the bioequivalence criteria (80%–125%) compared with the reference exposure.

## DISCUSSION

In this study, we present a newly established DD(G)I network centered around the highly polymorphic CYP2D6 enzyme, prone to DDIs, DGIs, and DDGIs. The presented network was built based on various previously published models and compound‐specific DD(G)I networks, combining and expanding them for a total of 32 distinct drug combinations involving 23 different compound models, also including 18 metabolites. The research presented in this study introduces a comprehensive CYP2D6 DDGI network that significantly advances beyond prior efforts. Specifically compared with the previously published quinidine DDGI modeling study by Feick *et al*.,^18^ the CYP2D6 network expands the scope to include eight additional CYP2D6 drugs—three victims and five perpetrators—encompassing a broader spectrum of interaction mechanisms, such as CYP2D6 down‐regulation by bupropion and mechanism‐based inactivation by paroxetine. The comprehensive coverage of CYP2D6 victim and perpetrator drugs, interaction scenarios, and interaction mechanisms within the developed network allows for a more nuanced understanding of CYP2D6 interactions. The good predictive performance of the network was evaluated using established graphical and quantitative measures. Subsequently, model‐based dose adjustments for simulated clinically untested D(D)GI scenarios were performed for the CYP2D6 substrates atomoxetine and metoprolol.

Previous studies have outlined the potential of PBPK DDGI networks in clinical applications and how these models may be used to generate model‐guided dose adaptations even in highly complex situations. For instance, Türk *et al*. provided dose adaptations covering the combined effect of genetic variants in two pharmacogenes (*CYP2C8* and *SLCO1B1*) and co‐administration of perpetrator drugs, such as gemfibrozil and itraconazole.^37^ Similarly, Wojtyniak and colleagues presented a DD(G)I network centered around simvastatin highlighting the combinatorial complexities occurring from DDGIs.^10^ The presented network was established based on a large number of CYP2D6 victim and perpetrator drugs as well as observed clinical data to provide a comprehensive foundation for the prediction of clinically untested CYP2D6 DD(G)I and even multiple DD(G)I scenarios with more than one perpetrator. To demonstrate the multitude of combinatorial possibilities of interactions, the effect of several CYP2D6 and CYP3A4 perpetrators on the exposure of the sensitive CYP2D6 substrate atomoxetine and the moderately sensitive CYP2D6 substrate metoprolol was predicted for different CYP2D6 activity scores. In a next step, model‐based dose adjustments were performed to demonstrate the translation of the model predictions into patient‐relevant information for clinical application. However, the applicability of this approach for patient‐individual dose adaptations is constrained by the extensive interindividual variability in the CYP2D6 enzyme activity, even when accounting for CYP2D6 activity scores.^21^ Like the population approach, individual predictions using PBPK models could be substantially improved with the integration of patient‐specific PK measurements and individual PK data.^38^ Here, hybrid population PBPK approaches may increase the confidence in individual PBPK model predictions, especially when used for clinical decision making.^38^ Our presented network can serve as a basis for such hybrid approaches.

The dose adjustments performed for atomoxetine and metoprolol resulted in substantial increases or decreases in the respective dose, highlighting the need for individual dose adaptations. Model simulations indicate that to match the exposure of atomoxetine in poor metabolizers (activity score 0) with the reference exposure (activity score 2, no co‐medication), a dose reduction to 6% of the original atomoxetine dose is required. Additionally, simulations reveal that co‐administrations of strong CYP2D6 inhibitors quinidine and paroxetine either alone or in combination with other CYP2D6 or CYP3A4 inhibitors resulted in a significant reduction in CYP2D6 activity across all activity scores above 0. This results in atomoxetine exposures similar to those simulated in poor metabolizers (activity score 0). Consequently, similarly extensive dose reductions are necessary to match their exposure levels to the reference group. The observed phenoconversion is consistent with the FDA‐approved label for STRATTERA®, stating that atomoxetine blood levels are increased ~10‐fold in poor metabolizers or those taking strong CYP2D6 inhibitors.^39^ Accordingly, the label recommends a dose reduction to 40% of the target dose for these individuals, with an optional subsequent dose escalation to 100% if symptoms fail to improve after four weeks and the initial dose is well‐tolerated. This recommendation is likely based on the observation of only modest differences in the frequency of ADRs between normal and poor metabolizers of CYP2D6 alongside the generally well‐tolerated atomoxetine doses. Additionally, while the label recommendations use traditional CYP2D6 phenotype categories, dose adjustments derived from our model simulations utilize the more detailed activity score categories.^8^ The broad normal metabolizer category used as the reference in label dosing contrasts with the specific activity score of 2 used in model‐based adjustments, explaining the variance in recommended dose reductions for poor metabolizers and patients on strong CYP2D6 inhibitors. For metoprolol, the Dutch Pharmacogenetics Working Group (DPWG) provides dose adjustments based on CYP2D6 phenotypes.^40^ Model‐derived dose recommendations for metoprolol in DGI scenarios have been found to be largely consistent with DPWG recommendations.^34^ The current study extends these recommendations to DDGI scenarios, incorporating the isolated and combined effects of strong CYP2D6 inhibitors paroxetine and quinidine, the effect of strong CYP3A4 inhibitor itraconazole, as well as the effect of weak CYP2D6 and CYP3A4 inhibitor cimetidine on the PK of metoprolol. Here, the most significant effect was observed with the co‐administration of quinidine and itraconazole, necessitating a reduction of the metoprolol dose to 12% of the original dose. While the presented dose adjustments were performed based on AUC_ss_, dose adjustments using PBPK models can also be performed for other PK parameters, such as trough concentration (*C*_trough_) or *C*_max_, when appropriate for the drug of interest.

In the future, the network can be expanded by developing novel PBPK models of victims or perpetrators within the OSP framework enabling the prediction of an even broader range of clinically relevant DD(G)I scenarios. The PBPK approach facilitates the prediction of concomitant medications and generation of individual optimizations, as highlighted in this work. While PBPK models are versatile and beneficial for clinical applications, such as dose optimizations, their adoption in clinical practice remains limited. This is chiefly due to a lack of clinical acceptance caused by an apparent lack of usability by the target user group or accountability issues.^5^ Here, our network can serve as the foundation for PBPK‐driven clinical decision support systems (CDSS) for clinicians and patients. These CDSS aim to generate optimal model‐based doses for an individual patient based on the underlying victim and perpetrator models of the relevant medication as well as patient‐specific genetic and demographic input data. Additionally, CDSS can provide a more accessible way to leverage the potential of PBPK networks outside of dedicated modeling software, consequently lowering the barriers of access and improving clinical utility.^5^

In total, the presented CYP2D6 network was built and evaluated based on 85 DGI, 121 DDI, and 42 DDGI scenarios demonstrating good predictive performance for all interaction types. Moreover, the developed network shows good coverage of the *FDA Table of Substrates, Inhibitors and Inducers* for the CYP2D6 enzyme with three strong inhibitors, two weak inhibitors, three sensitive substrates and one moderately sensitive substrate included.^41^ Paroxetine, a strong clinical index inhibitor of CYP2D6 is featured while desipramine and dextromethorphan provide two sensitive clinical index substrates of CYP2D6.^15^ However, due to the absence of compatible PBPK models in the published literature, no moderate CYP2D6 inhibitors listed by the FDA are currently represented in the network. Once such models become available, our network can be expanded accordingly. This modularity is essential for incorporating new data and models, enhancing the network's utility and accuracy over time. Additionally, good coverage of the *FDA Table of Substrates, Inhibitors and Inducers* ensures regulatory compliance and applicability to a broad range of clinical scenarios. Hence, our network may aid clinical decision‐making, especially in the context of MID3, where previously published PBPK DD(G)I networks have shown to be of considerable interest complementing clinical studies. For instance, extrapolations from moderate inhibitors to weak or strong inhibitors using a CYP3A4 DDI network have been successfully applied to support and accelerate clinical development.^42^ Here, PBPK interaction networks offer a robust framework for simulating and predicting drug interactions *in silico*, potentially reducing the need for early‐phase clinical trials.^5^

Most models within the network were utilized without modifications to their original configurations. However, PBPK models for atomoxetine, bupropion, cimetidine, and fluvoxamine were extended to include additional interaction parameters that mechanistically describe DDIs mediated by these drugs. All interaction parameters implemented in the respective models were sourced from the published literature. For instance, the bupropion model was modified to reflect its unique interaction mechanism, a mix of competitive inhibition and down‐regulation of CYP2D6 caused by bupropion and its metabolites hydroxybupropion, erythrohydrobupropion, and threohydrobupropion as described by Sager *et al*.^27^ These model extensions were necessary to accurately capture the interactions between bupropion and the CYP2D6 victim drugs atomoxetine and desipramine.^28^, ^43^ In the case of metoprolol, the model was refined to include a CYP3A4‐mediated clearance pathway, replacing a previously implemented non‐specific pathway. This adjustment aligns with *in vitro* evidence suggesting that metoprolol's metabolism is partially mediated by CYP3A4.^35^ Although CYP2D6 is generally considered non‐inducible by prototypical CYP inducers, such as carbamazepine and rifampicin, Bennett *et al*. have reported an increase of metoprolol AUC after rifampicin co‐administration by ~30% indicating additional non‐CYP2D6‐mediated metabolism.^44^

Further adaptations of the network models may increase their predictive performance to cover various additional DDGI scenarios. This includes incorporating *in vitro* interaction parameters for metabolites currently not included in the respective models, as metabolites often contribute to the apparent interactions caused by their parent drug. For instance, Sauer and colleagues reported an additive effect of CYP3A4 inhibition caused by atomoxetine and its metabolites N‐desmethylatomoxetine and 4‐hydroxyatomoxetine, which are not included in the current atomoxetine model.^32^ In addition, models can be refined once new insights into the PK of a drug emerge. For instance, predicted desipramine profiles show a trend toward underprediction in DDI scenarios, although all interaction PK ratios were within the limits proposed by Guest *et al*.^19^ This discrepancy may have been caused by possible transport processes that were not included in the desipramine model but may be targeted by perpetrators. For example, experiments with rats indicate that P‐gp inhibitors can increase desipramine brain concentrations.^45^ However, since no broad knowledge and especially quantifying parameters on the potential transport of desipramine by P‐gp were available in the literature, P‐gp‐mediated transport was not included in the model. Should further data indicating relevant transport by P‐gp or other transporters *in vivo* become available, the desipramine model can be refined accordingly. Overall, these adaptations showcase the mechanistic flexibility of PBPK models and their adaptability to describe complex DDGI scenarios.

Although a large amount of data was used for network development and evaluation, the generalizability of the network predictions may be limited to some extent caused by the design of the clinical studies used. For instance, most studies were conducted with European or American subjects, while data on Asian subjects were only available for the victim drugs atomoxetine, paroxetine, and risperidone. Hence, the predictive performance may vary for ethnicities not represented in the respective study cohorts. Moreover, as study participants typically were healthy men aged around 30, the predictive performance may differ for women and older patients. Finally, the predictive performance of the presented network in various other vulnerable patient populations, such as pediatric patients or patients with renal or hepatic impairment, remains unknown. Here, previous examples highlight how PBPK DD(G)I models can be adapted to cover these scenarios.^46^, ^47^, ^48^ Additional studies covering a wider range of demographic characteristics of study participants would be of interest to extend and evaluate the developed network, increasing its clinical utility in predicting real‐world scenarios.

In conclusion, this work presents a comprehensive whole‐body PBPK DDGI network that can describe and predict the simultaneous effects of CYP2D6 activities and concomitant administrations of various perpetrator drugs on the PK of victim drugs. Overall, the developed network not only provides a valuable basis for the realization of PBPK MIPD for CYP2D6 victim drugs, but also represents a well‐suited foundation for applications within MID3 due to the broad coverage of CYP2D6 victim and perpetrator drugs. The modular nature of PBPK models supports this broad applicability of the network by facilitating future extensions through the inclusion of additional perpetrator or victim drugs. Therefore, all model files will be made publicly available (http://models.clinicalpharmacy.me).

## FUNDING

This work is part of the Horizon 2020 INSPIRATION (Qualified Open Systems Pharmacology Modeling Network of Drug–Drug‐Gene‐Interactions) project. The INSPIRATION project (grant 643272, FKZ 031 L0241) is supported by the German Federal Ministry of Education and Research and ZonMW (9003035202) under the framework of ERACoSysMed. Matthias Schwab was supported in parts by the Robert Bosch Stiftung Stuttgart, Germany, and the Deutsche Forschungsgemeinschaft (DFG) under Germany's Excellence Strategy‐EXC 2180–390900677. Thorsten Lehr, Jesse J. Swen and Maaike van der Lee were supported by the European Union Horizon 2021 SafePolyMed (grant 101057639).

## CONFLICT OF INTEREST

Denise Feick and Donato Teutonico are employees of Sanofi and may hold shares and/or stock options in the company. Annika Schneider and Juri Solodenko are employees of Bayer AG. Sebastian Frechen was an employee of Bayer AG. Juri Solodenko and Annika Schneider use Open Systems Pharmacology software, tools, or models in their professional roles. Donato Teutonico, Juri Solodenko and Thorsten Lehr are members of the Open Systems Pharmacology Management Team. Sebastian Frechen was a member of the Open Systems Pharmacology Sounding Board. All other authors declared no competing interest for this work.

## AUTHOR CONTRIBUTIONS

All authors wrote the manuscript. S.R., H.L.H.L., D.F., D.S., and T.L. designed the research. S.R., H.L.H.L., and D.F. performed the research. S.R., H.L.H.L., D.F., and D.S. analyzed the data.

## Supporting information


Data S1.
