# Supplementary material for: A Comprehensive CYP2D6 Drug–Drug–Gene Interaction Network for Application in Precision Dosing and Drug Development
Source: Clin Pharmacol Ther. 2025 Feb 14;117(6):1718–31. doi: 10.1002/cpt.3604 (PMC12087690; doi:10.1002/cpt.3604)
Supplement: Supplementary file 1 — Data S1. [file CPT-117-1718-s001.pdf]

Clinical Pharmacology & Therapeutics

A Comprehensive CYP2D6 Drug-Drug-Gene Interaction Network for Application in Precision Dosing and Drug Development

Supplement S1 - Model Information and Evaluation

Simeon Rüdesheim[1,2*], Helena Leonie Hanae Loer[1*], Denise Feick[1,3], Fatima Zahra Marok[1], Laura Maria Fuhr[1], Dominik Selzer[1], Donato Teutonico[4], Annika R. P. Schneider[5], Juri Solodenko[5], Sebastian Frechen[5], Maaike van der Lee[6], Dirk Jan A. R. Moes[6], Jesse J. Swen[6], Matthias Schwab[2,7,8], and Thorsten Lehr[1]

[*] Authors contributed equally
[1] Clinical Pharmacy, Saarland University, Saarbrücken, Germany
[2] Dr. Margarete Fischer-Bosch-Institute of Clinical Pharmacology, Stuttgart, Germany
[3] Drug Metabolism and Pharmacokinetics, Sanofi R&D, Frankfurt am Main, Germany
[4] Translational Medicine & Early Development, Sanofi R&D, Vitry-sur-Seine, France
[5] Bayer AG, Pharmaceuticals, Research & Development, Model-informed drug development, Leverkusen, Germany
[6] Department of Clinical Pharmacy & Toxicology, Leiden University Medical Center, Leiden, The Netherlands
[7] Departments of Clinical Pharmacology, Pharmacy and Biochemistry, University of Tübingen, Tübingen, Germany
[8] Cluster of Excellence iFIT (EXC2180) "Image-guided and Functionally Instructed Tumor Therapies", University of Tübingen, Tübingen, Germany

Corresponding Author:

Prof. Dr. Thorsten Lehr, Clinical Pharmacy, Saarland University, Campus C4 3, 66123 Saarbrücken, Germany, Phone: +49 681 302 70255, Email: thorsten.lehr@mx.uni-saarland.de

Contents

S1 Desipramine PBPK Model Development **2**
S1.1 PBPK Model Building 2
S1.2 PBPK Model Evaluation 2

S2 Desipramine Base Model Results **3**
S2.1 Clinical Studies 4
S2.2 Drug-Dependent Parameters 5
S2.3 Plasma Concentration-Time Profiles (Semilogarithmic Representation) 6
S2.4 Plasma Concentration-Time Profiles (Linear Representation) 8
S2.5 Predicted Compared to Observed Concentrations 10
S2.6 Mean Relative Deviation of Plasma Concentration Predictions 11
S2.7 Predicted Compared to Observed AUC_{last} and C_{max} Values 12
S2.8 Geometric Mean Fold Errors of Predicted AUC_{last} and C_{max} Values 13
S2.9 Sensitivity Analysis 15

S3 Desipramine DGI Model Results **17**
S3.1 Clinical Studies 17
S3.2 Plasma Concentration-Time Profiles (Semilogarithmic Representation) 18
S3.3 Plasma Concentration-Time Profiles (Linear Representation) 20
S3.4 DGI AUC_{last} and C_{max} Ratios 22

S4 DD(G)I Network Development **24**
S4.1 System-Dependent Parameters 24
S4.2 Types of Interaction 28
S4.3 Published PBPK DDI Models 29

S5 DGI Model Evaluation **35**
S5.1 DGI AUC_{last} and C_{max} Ratios 35
S5.2 Geometric Mean Fold Errors of Predicted DGI AUC_{last} and C_{max} Ratios 36

S6 DDI Model Evaluation **39**
S6.1 Alprazolam 39
S6.2 Atomoxetine 43
S6.3 (*E*)-Clomiphene 48
S6.4 Desipramine 68
S6.5 Dextromethorphan 73
S6.6 Digoxin 78
S6.7 Metoprolol 82
S6.8 Mexiletine 89
S6.9 Midazolam 93
S6.10 Paroxetine 97
S6.11 Quinidine 101
S6.12 Risperidone 106
S6.13 DDI AUC_{last} and C_{max} Ratios 111

S7 DDGI Model Evaluation **112**
S7.1 DDGI AUC_{last} and C_{max} Ratios 112
S7.2 Geometric Mean Fold Errors of Predicted DDGI AUC_{last} and C_{max} Ratios 113

S8 Model-Informed Dose Adaptations **115**

References **117**

S1 Desipramine PBPK Model Development

S1.1 PBPK Model Building

Physiologically based pharmacokinetic (PBPK) model development for the desipramine parent-metabolite model was initiated with an extensive literature search for physicochemical parameters as well as information on absorption, distribution, metabolism and excretion processes. Moreover, plasma concentration-time profiles of desipramine and 2-hydroxydesipramine after intravenous and oral administrations of desipramine, as well as important meta data such as information on study subjects and administration protocols were collected. Plasma concentration-time profiles were split into a training dataset for model development and a test dataset for model evaluation. Studies for the training dataset were selected to include various routes of administration (i.e., intravenous and oral) and a wide dosing range. To determine appropriate quantitative structure-activity relationship (QSAR) techniques for computing partition coefficients and cellular permeabilities, parameter optimizations were conducted. Additionally, using the Monte Carlo algorithm, model parameter values that were not available in the literature, such as relevant catalytic and transport rate constants (k_{cat}) and intestinal permeability, were optimized by fitting model simulations to all studies of the training dataset.

For drug-gene interaction (DGI) modeling, the cytochrome P450 (CYP) 2D6 Michaelis-Menten constant (K_M) values for desipramine 2-hydroxylation were kept constant for all modeled phenotypes. CYP2D6 k_{cat} values were adjusted separately for the different phenotypes: CYP2D6 poor metabolizers were assumed to show no CYP2D6 activity (k_{cat} = 0 1/min) whereas the k_{cat} value for normal metabolizers (NMs) was optimized. Of note, the “fast NM” phenotype is singular to the studies conducted by Brøsen et al. and does not directly correspond to any traditional phenotype category. Rather, the authors grouped NM individuals with a particularly low sparteine metabolic ratio within the NM category into a category separate to the broad NM category. Modeling of the corresponding CYP2D6 metabolism was complicated by the fact that no CYP2D6 genotypes or activity scores (ASs) were reported for any of the respective study cohorts. To reflect the higher activity of the fast NMs compared to NMs, fast NM was assumed to correspond to an AS of 2, with the respective CYP2D6 k_{cat} value used for fast NMs.

S1.2 PBPK Model Evaluation

Model performance of the desipramine PBPK model was evaluated graphically by comparison of population predicted (1000 individuals) and observed desipramine and 2-hydroxydesipramine plasma concentration-time profiles. Additionally, predicted plasma concentrations for mean individuals, area under the plasma concentration-time curve calculated from the first to the last concentration measurement (AUC_{last}) and maximum plasma concentration (C_{max}) values were compared to observed values in goodness-of-fit plots. Mean relative deviations (MRDs) for all predicted concentration time-profiles as well as geometric mean fold errors (GMFEs) for all predicted AUC_{last} and C_{max} values were calculated as previously described [1].

Sensitivity of the parent-metabolite model to single model parameters was calculated, determined as relative change of $AUC_{0\text{-}24h}$ at steady state according to Equation S1. A relative perturbation of 1000% (variation range 10.0, maximum number of 2 steps) was applied. Parameters were included into the analysis if (i) they have been optimized, (ii) they are associated with optimized parameters or (iii) they could have a strong impact due to their use in the calculation of permeabilities or partition coefficients. Parameters were considered sensitive, if their sensitivity value was equal or greater than 0.5.

$$S = \frac{\Delta AUC}{AUC} \cdot \frac{p}{\Delta p} \tag{S1}$$

S = sensitivity of the AUC to the examined model parameter, ΔAUC = change of the AUC, AUC = simulated AUC with the original parameter value, Δp = change of the examined parameter value and p = original parameter value.

DGI prediction performance was evaluated by comparing population predicted (1000 individuals) and observed plasma concentration-time profiles of the different phenotypes. Predicted DGI AUC_{last} and C_{max} ratios were calculated relative to normal metabolizers as previously described [1] with subsequent comparison to the corresponding observed ratios in goodness-of-fit plots. The limits proposed by Guest et al. were applied to determine prediction accuracy (including 20% variability) [2]. Quantitative assessment was conducted by calculating GMFE values for all predicted DGI AUC_{last} and C_{max} ratios as previously described [1].

S2 Desipramine Base Model Results

Whole-body PBPK models for desipramine and its metabolite 2-hydroxydesipramine were developed in PK-Sim® using a total of 39 plasma concentration-time profiles of desipramine (28 profiles) and 2-hydroxydesipramine (11 profiles) from 20 clinical studies. Here, plasma concentration-time profiles were reported after intravenous administrations of 50 mg desipramine hydrochloride as well as single and multiple oral administrations of 25–100 mg desipramine hydrochloride. Comprehensive information on clinical studies used for model building and evaluation is given in Table S1. The main metabolic pathway of desipramine, its 2-hydroxylation to its main metabolite 2-hydroxydesipramine, was incorporated via CYP2D6. Residual metabolism and 2-hydroxydesipramine clearance was implemented via an unspecific hepatic clearance process. Additionally, passive glomerular filtration was incorporated in the model for both compounds. All relevant drug-dependent parameters for desipramine and 2-hydroxydesipramine are listed in Table S2. Information on the expression and localization of relevant proteins is provided in Tables S8–S10. Population predictions of all modeled plasma concentration-time profiles of desipramine and 2-hydroxydesipramine in semilogarithmic and linear representations are shown in Sections S2.3–S2.4.

Figures S5–S6 display the good descriptive and predictive performance of the desipramine parent-metabolite model. Overall, regarding the predicted plasma concentrations, AUC_{last} and C_{max} values, 96%, 100% and 100% of the desipramine training dataset, 91%, 100% and 100% of the desipramine test dataset, 100%, 100% and 100% of the 2-hydroxydesipramine training dataset as well as 90%, 100% and 100% of the 2-hydroxydesipramine test dataset were within two-fold of the corresponding observed values, respectively. Values for MRD, as well as AUC_{last} and C_{max} values of all profiles are provided in Tables S3–S4.

A local sensitivity analysis using a multiple dose simulation of 50 mg desipramine hydrochloride revealed that the desipramine model is sensitive to perturbation of the CYP2D6 k_{cat} and lipophilicity of desipramine (both optimized), as well as the fraction unbound of desipramine and CYP2D6 K_M (both implemented as fixed literature values). The 2-hydroxydesipramine model is sensitive to changes of the fraction unbound of 2-hydroxydesipramine (fixed value from the literature) and its unspecific hepatic clearance process (optimized). Table S5 provides a list of parameters evaluated during the local sensitivity analysis with its results visualized in Figure S7.

S2.1 Clinical Studies

Table S1: Clinical study data used for desipramine model development

Dose [mg][a]	Route	n	Population[b]	Fem. [%]	Age [years]	Weight [kg]	BMI [kg/m²]	Phenotype/AS	Molecule	Dataset	Reference
Intravenous											
50 (44)	s.d. iv 60 min inf	3	European [3]	33	29 (24–35)	-	-	PM	DES	training	Brøsen 1988 [4]
50 (44)	s.d. iv 60 min inf	4	European [3]	75	29.75 (22–37)	-	-	NM	DES	training	Brøsen 1988 [4]
50 (44)	s.d. iv 60 min inf	4	European [3]	50	23.75 (22–27)	-	-	fast NM	DES	training	Brøsen 1988 [4]
Oral											
25 (22)	s.d. po	6	European [3]	21	(27–51)	-	-	PM	DES	training	Spina 1987 [5]
25 (22)	s.d. po	8	European [3]	21	(27–51)	-	-	NM	DES	training	Spina 1987 [5]
50 (44)	s.d. po	13	European [3]	0	26 (20–56)	84 (69–93)	-	-	DES	training	Aarnoutse 2005 [6]
50 (44)	s.d. po	6	American [7]	0	44 (35–53)	78.2 (60.3–92.8)	-	-	DES	test	Bergstrom 1992 [8]
50 (44)	s.d. po	26	American [7]	4	33.6±7.2	80.9±11.2	26.4±2.8	-	DES, OHD	training	Boni 2009 [9]
50 (44)	s.d. po	17	American [7]	53	31.5±10.8	-	24.6±3.19	-	DES	training	Harris 2007 [10]
50 (44)	s.d. po	20	European [3]	55	58.3±9.9	84.3±28.5	29.3±10.1	-	DES, OHD	test	Hynes 2015 [11]
50 (44)	s.d. po	24	European [3]	-	(18–45)	-	-	-	DES	training	Madani 2002 [12]
50 (44)	s.d. po	20	American [7]	15	35.0±7.7	79.9±9.8	-	-	DES, OHD	test	Nichols 2009 [13]
50 (44)	s.d. po	38	American [7]	-	(18–45)	above 60	(18–30)	-	DES	test	Nichols 2013 [14]
50 (44)	s.d. po	20	American [7]	-	(18–55)	above 50	(18–30)	-	DES, OHD	test	Patroneva 2008 [15]
50 (44)	s.d. po	15	American [7]	0	-	-	-	-	DES	test	Reese 2008 [16]
50 (44)	s.d. po	22	American [7]	50	(26–55)	-	(18.8–30.4)	-	DES	test	Sauer 2004 [17]
50 (44)	s.d. po	16	American [7]	56	42 (21–63)	71.4	-	-	DES	test	Skinner 2003 [18]
50 (44)	q.d. po	6	American [7]	0	30.4±5.2	73.3±8.5	-	-	DES	test	Alderman 1997 [19]
100 (88)	s.d. po	6	European [3]	83	26.8 (24–33)	-	-	AS = 2	DES, OHD	test	Bergmann 2001 [20]
100 (88)	s.d. po	6	European [3]	33	36.7 (22–54)	-	-	AS = 2.5	DES, OHD	test	Bergmann 2001 [20]
100 (88)	s.d. po	6	European [3]	50	28.8 (23–34)	-	-	PM	DES	training	Brøsen 1986 [21]
100 (88)	s.d. po	6	European [3]	50	28.3 (21–36)	-	-	NM	DES, OHD	training	Brøsen 1986 [21]
100 (88)	s.d. po	6	European [3]	50	24.1 (21–30)	-	-	fast NM	DES, OHD	training	Brøsen 1986 [21]
100 (88)	s.d. po	8	European [3]	0	(22–24)	-	-	PM	DES	test	Brøsen 1993 [22]
100 (88)	s.d. po	5	European [3]	0	(22–24)	-	-	NM	DES, OHD	test	Brøsen 1993 [22]
100 (88)	s.d. po	4	European [3]	0	(22–24)	-	-	fast NM	DES, OHD	test	Brøsen 1993 [22]
100 (88)	s.d. po	6	European [3]	50	27.7 (23–38)	-	-	-	DES, OHD	test	Brøsen 1989 [23]
100 (88)	s.d. po	6	European [3]	0	(24–38)	(67–81)	-	-	DES	test	Spina 1995 [24]

AS: CYP2D6 activity score, BMI: body mass index, DES: desipramine, fem: females, inf: infusion, iv: intravenous, n: number of study participants, NM: CYP2D6 normal metabolizer, OHD: 2-hydroxydesipramine, PM: CYP2D6 poor metabolizer, po: oral, q.d.: once daily, -: not available. Values are given as mean (range). Respective doses of desipramine base were calculated and incorporated in simulations. [a] Dose given as desipramine hydrochloride (desipramine base). [b] Population used in simulations.

S2.2 Drug-Dependent Parameters

Table S2: Drug-dependent parameters of the desipramine model

	Desipramine			2-Hydroxydesipramine			
**Parameter**	**Value**	**Source**	**Literature**	**Value**	**Source**	**Literature**	**Description**
MW [g/mol]	266.4	Lit.	266.4 [25]	282.4	Lit.	282.4 [25]	Molecular weight of desipramine base
pK_a (base 1)	2.84	Lit.	2.84 [26]	4.51	Lit.	4.51 [26]	Acid dissociation constant
pK_a (base 2)	10.02	Lit.	10.02 [27]	9.90	Lit.	9.90 [26]	Acid dissociation constant
pK_a (acid)	-	-	-	10.63	Lit.	10.63 [26]	Acid dissociation constant
Solubility (pH 6.5) [mg/L]	214.29	Lit.	214.29 [26]	282.39	Lit.	282.39 [26]	Solubility
Lipophilicity	3.52	Opt.	3.90, 4.90 [26, 27]	2.33	Opt.	3.17 [26]	Lipophilicity
$f_{u,p}$ [%]	14	Lit.	14[a] [28]	12	Lit.	12[a] [28]	Fraction unbound plasma
CYP2D6 (DES $\rightarrow$ OHD) K_M [µmol/L]	0.73	Lit.	6.1 × 0.12[b] [29, 30]				Michaelis-Menten constant
CYP2D6 (DES $\rightarrow$ OHD) k_{cat} [1/min]	5.03[d]	Opt.	-	-	-	-	Catalytic rate constant (NM)
CYP2D6 (DES $\rightarrow$ OHD) k_{cat} [1/min]	9.86	Opt.	-	-	-	-	Catalytic rate constant (AS = 2)
CYP2D6 (DES $\rightarrow$ OHD) k_{cat} [1/min]	28.88	Opt.	-	-	-	-	Catalytic rate constant (AS = 2.5)
CYP2D6 (DES $\rightarrow$ OHD) k_{cat} [1/min]	0	Opt.	-				Catalytic rate constant (PM)
CL_{hep} [1/min]	0.54	Opt.	-	9.93	Opt.	-	Hepatic metabolic clearance
GFR fraction	1	Asm.	-	1	Asm.	-	Fraction of filtered drug in the urine
EHC continuous fraction	1	Asm.	-	1	Asm.	-	Fraction of bile continually released
Partition coefficients	Diverse	Calc.	R & R [31, 32]	Diverse	Calc.	R & R [31, 32]	Cell to plasma partition coefficients
Cellular perm. [cm/min]	0.27	Calc.	PK-Sim [33]	0.01	Calc.	PK-Sim [33]	Permeability into the cellular space
Intestinal perm. [cm/min]	$1.14 \cdot 10^{-5}$	Opt.	$6.44 \cdot 10^{-4}$	$3.19 \cdot 10^{-5}$	Calc	$3.19 \cdot 10^{-5}$ (calc.)	Transcellular intestinal permeability
Formulation	Solution	Asm.	-	-	-	-	Formulation used in predictions

AS: CYP2D6 activity score, asm.: assumed, calc.: calculated, CYP: cytochrome P450, DES: desipramine, EHC: enterohepatic circulation, GFR: glomerular filtration rate, lit.: literature, NM: CYP2D6 normal metabolizer, OHD: 2-hydroxydesipramine, opt.: optimized, PK-Sim: PK-Sim calculation method, PM: CYP2D6 poor metabolizer, R & R: Rodgers & Rowland calculation method, -: not implemented/not available. [a] Calculated with $f_{u,p}$ predictor [28]. [b] Reported K_M values adjusted for fraction unbound in the incubation (fu_{inc}) = 12% (calculated) [30]. [d] Also assumed for studies where no information about CYP2D6 polymorphism was provided.

S2.3 Plasma Concentration-Time Profiles (Semilogarithmic Representation)

Profiles from DGI studies in semilogarithmic representation can be found in Subsection S3.2 of the separate Section S3 "Desipramine DGI Model Results".

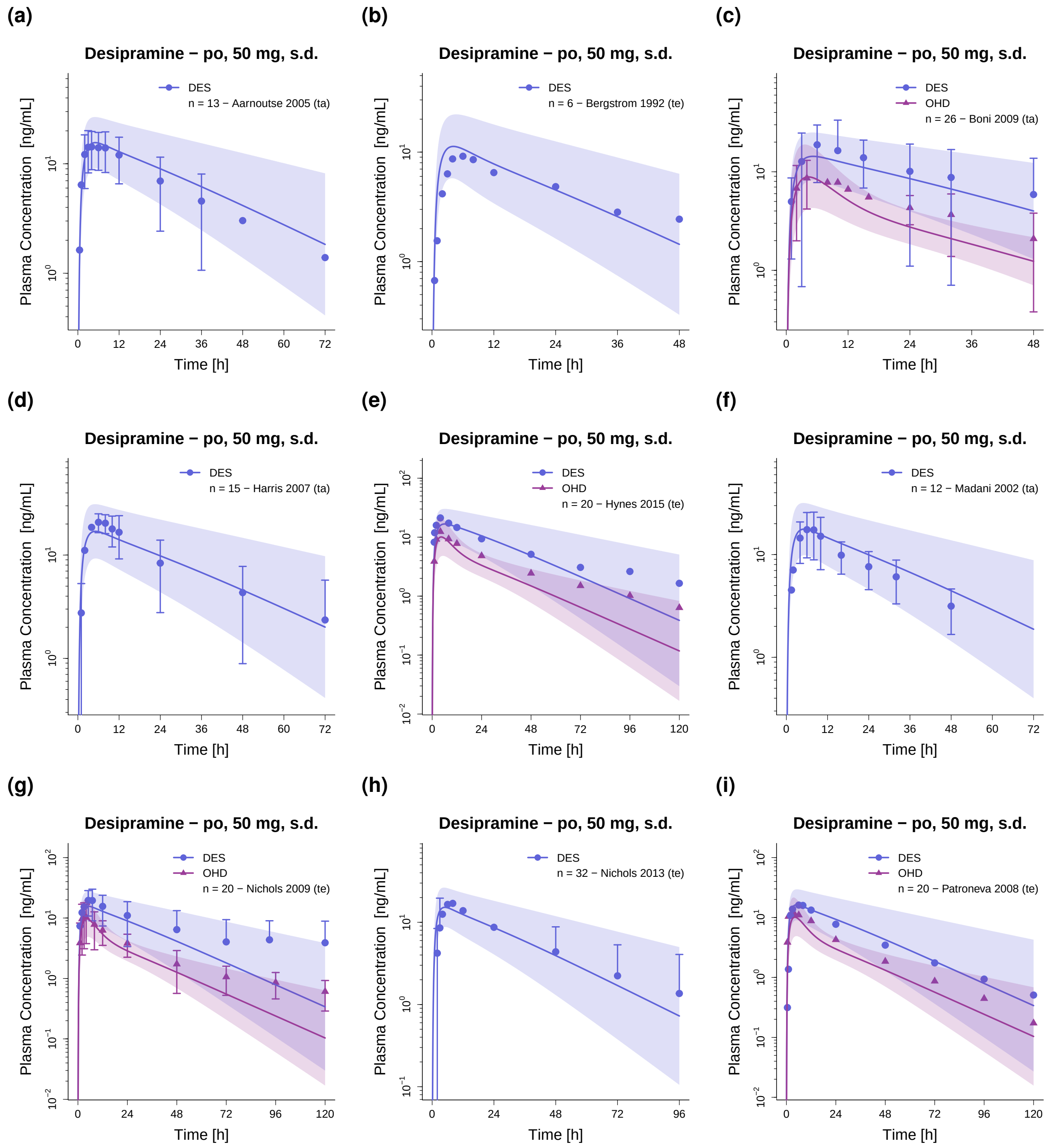

Figure S1: Predicted compared to observed plasma concentration-time profiles of desipramine and 2-hydroxydesipramine. Population predicted (1000 individuals) geometric means are shown as lines, corresponding geometric standard deviations as shaded areas and observed data as dots/triangles (± standard deviation, if reported) [6, 8–15]. DES: desipramine n: number of study participants, OHD: 2-hydroxydesipramine, po: oral, s.d.: single dose, ta: training dataset, te: test dataset.

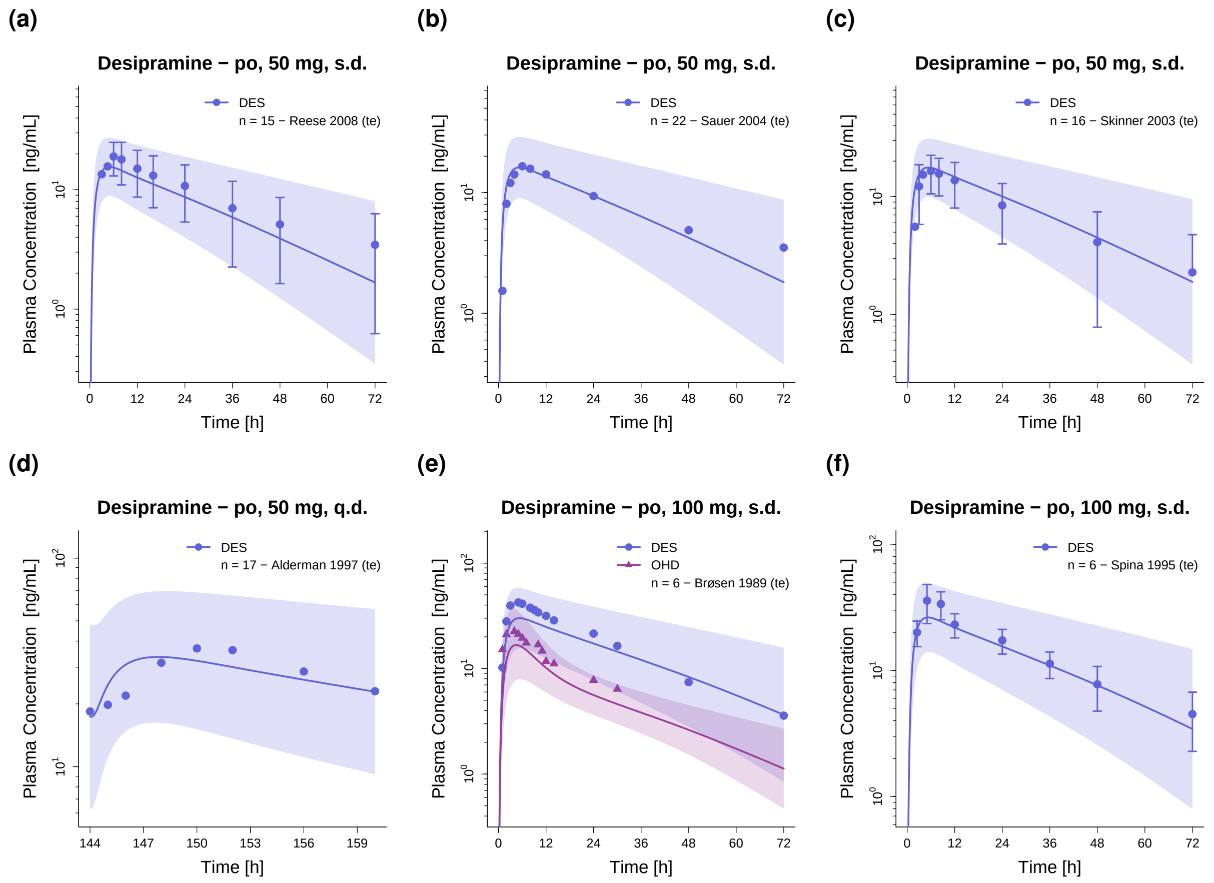

Figure S2: Predicted compared to observed plasma concentration-time profiles of desipramine and 2-hydroxydesipramine. Population predicted (1000 individuals) geometric means are shown as lines, corresponding geometric standard deviations as shaded areas and observed data as dots/triangles (± standard deviation, if reported) [16–19, 23, 24]. DES: desipramine n: number of study participants, OHD: 2-hydroxydesipramine, po: oral, q.d.: once daily, s.d.: single dose, te: test dataset.

S2.4 Plasma Concentration-Time Profiles (Linear Representation)

Profiles from DGI studies in semilogarithmic representation can be found in Subsection S3.3 of the separate Section S3 "Desipramine DGI Model Results".

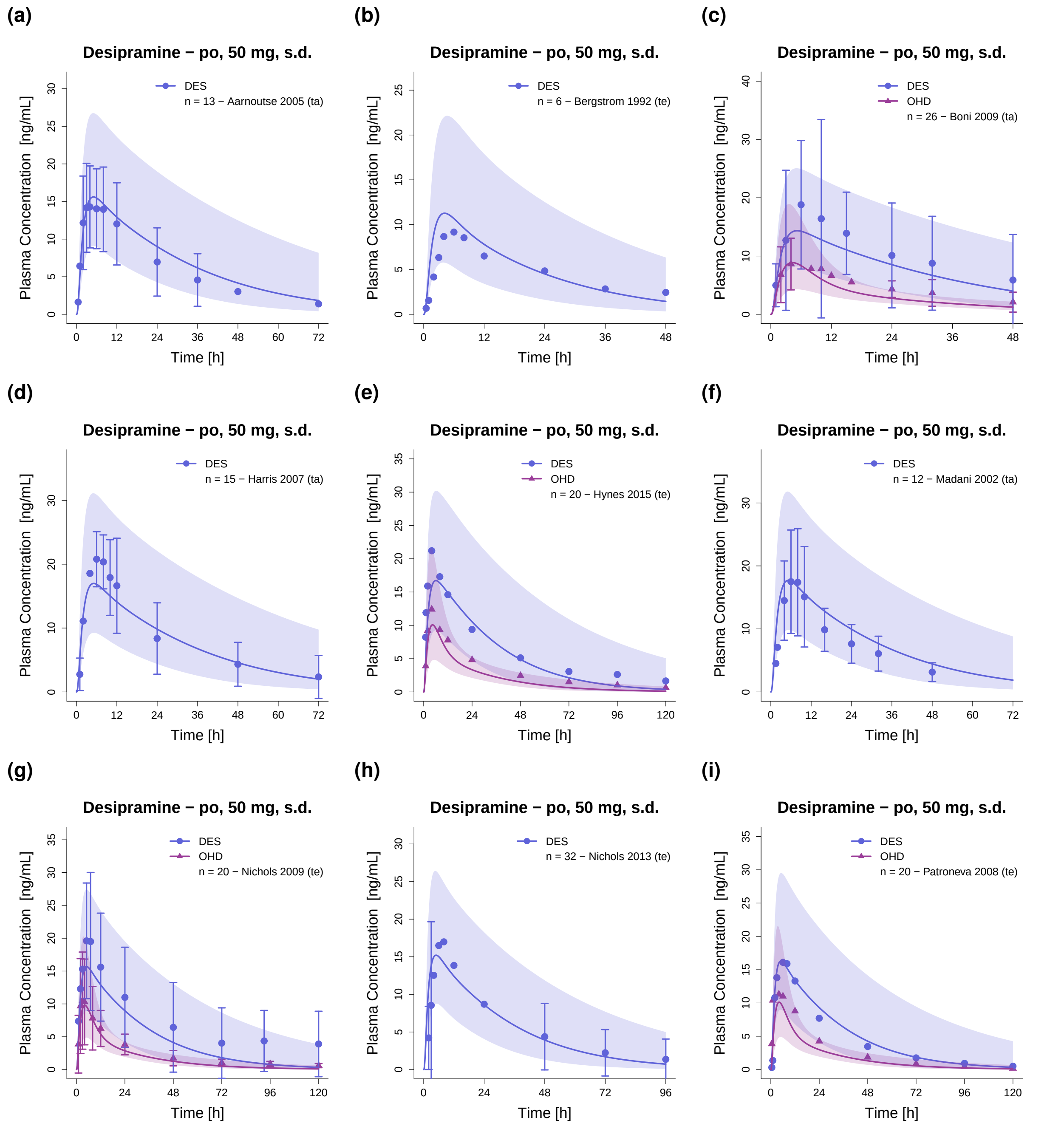

Figure S3: Predicted compared to observed plasma concentration-time profiles of desipramine and 2-hydroxydesipramine. Population predicted (1000 individuals) geometric means are shown as lines, corresponding geometric standard deviations as shaded areas and observed data as dots/triangles (± standard deviation, if reported) [6, 8–15]. DES: desipramine n: number of study participants, OHD: 2-hydroxydesipramine, po: oral, s.d.: single dose, ta: training dataset, te: test dataset.

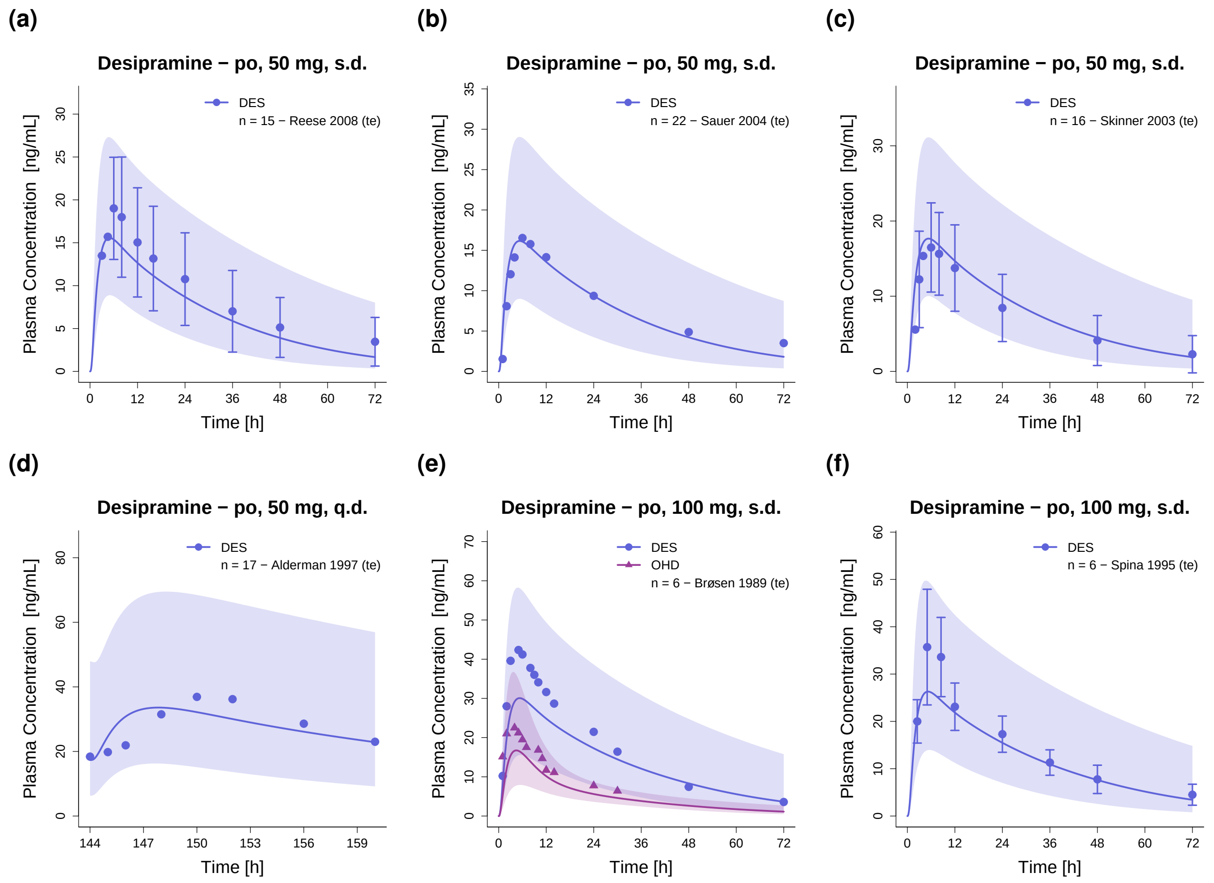

Figure S4: Predicted compared to observed plasma concentration-time profiles of desipramine and 2-hydroxydesipramine. Population predicted (1000 individuals) geometric means are shown as lines, corresponding geometric standard deviations as shaded areas and observed data as dots/triangles (± standard deviation, if reported) [16–19, 23, 24]. DES: desipramine n: number of study participants, OHD: 2-hydroxydesipramine, po: oral, q.d.: once daily, s.d.: single dose, te: test dataset.

S2.5 Predicted Compared to Observed Concentrations

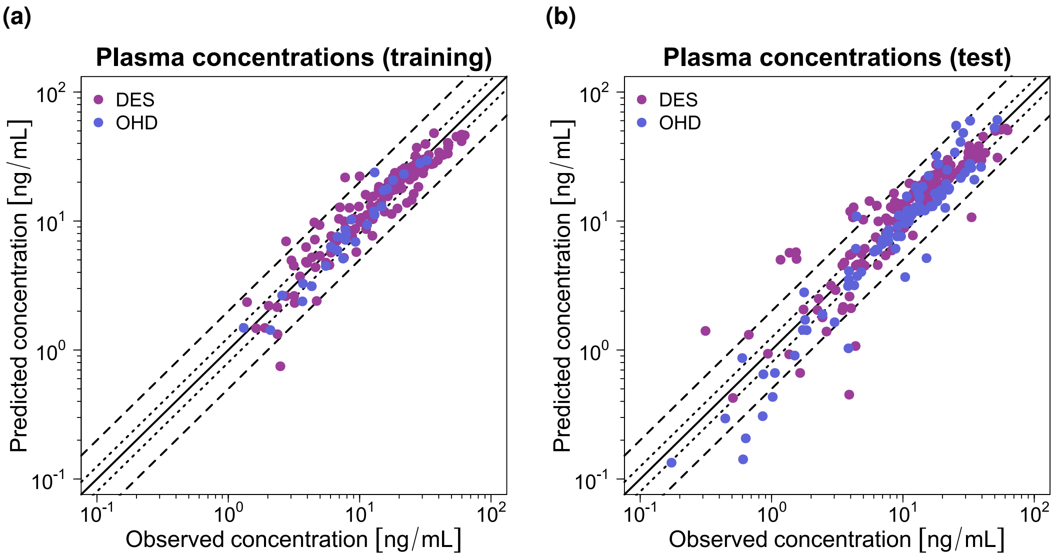

Figure S5: Goodness-of-fit plots comparing predicted and observed plasma concentration values of desipramine and 2-hydroxydesipramine. The solid line marks the line of identity. Dotted lines indicate 1.25-fold, dashed lines indicate 2-fold deviation. DES: desipramine, OHD: 2-hydroxydesipramine.

S2.6 Mean Relative Deviation of Plasma Concentration Predictions

Table S3: MRD values of desipramine plasma concentration predictions

Dose [mg][a]	Route	MRD DES	MRD OHD	Phenotype/AS	Dataset	Reference
Intravenous						
50 (44)	s.d. iv 60 min inf	1.20	-	PM	training	Brøsen 1988 [4]
50 (44)	s.d. iv 60 min inf	1.16	-	NM	training	Brøsen 1988 [4]
50 (44)	s.d. iv 60 min inf	1.72	-	fast NM	training	Brøsen 1988 [4]
Oral						
25 (22)	s.d. po	1.11	-	PM	training	Spina 1987 [5]
25 (22)	s.d. po	1.23	-	NM	training	Spina 1987 [5]
50 (44)	s.d. po	1.37	-	-	training	Aarnoutse 2005 [6]
50 (44)	s.d. po	1.84	-	-	test	Bergstrom 1992 [8]
50 (44)	s.d. po	1.14	1.28	-	training	Boni 2009 [9]
50 (44)	s.d. po	1.45	-	-	training	Harris 2007 [10]
50 (44)	s.d. po	1.50	1.63	-	test	Hynes 2015 [11]
50 (44)	s.d. po	1.44	-	-	training	Madani 2002 [12]
50 (44)	s.d. po	2.26	1.76	-	test	Nichols 2009 [13]
50 (44)	s.d. po	1.56	-	-	test	Nichols 2013 [14]
50 (44)	s.d. po	1.85	1.80	-	test	Patroneva 2008 [15]
50 (44)	s.d. po	1.21	-	-	test	Reese 2008 [16]
50 (44)	s.d. po	1.63	-	-	test	Sauer 2004 [17]
50 (44)	s.d. po	1.55	-	-	test	Skinner 2003 [18]
50 (44)	q.d. po	1.15	-	-	test	Alderman 1997 [19]
100 (88)	s.d. po	1.13	1.29	AS = 2	test	Bergmann 2001 [20]
100 (88)	s.d. po	1.09	1.46	AS = 2.5	test	Bergmann 2001 [20]
100 (88)	s.d. po	1.34	-	PM	training	Brøsen 1986 [21]
100 (88)	s.d. po	1.39	1.22	NM	training	Brøsen 1986 [21]
100 (88)	s.d. po	1.44	1.24	fast NM	training	Brøsen 1986 [21]
100 (88)	s.d. po	1.58	-	PM	test	Brøsen 1993 [22]
100 (88)	s.d. po	1.54	1.31	NM	test	Brøsen 1993 [22]
100 (88)	s.d. po	1.95	1.60	fast NM	test	Brøsen 1993 [22]
100 (88)	s.d. po	1.22	1.45	-	test	Brøsen 1989 [23]
100 (88)	s.d. po	1.20	-	-	test	Spina 1995 [24]
**Mean DES MRD training dataset (range):**		**1.33 (1.11–1.72), 12/12 with MRD $\leq$ 2**				
**Mean DES MRD test dataset (range):**		**1.52 (1.09–2.26), 15/16 with MRD $\leq$ 2**				
**Overall DES MRD (range):**		**1.44 (1.09–2.26), 27/28 with MRD $\leq$ 2**				
**Mean OHD MRD training dataset (range):**		**1.24 (1.22–1.28), 3/3 with MRD $\leq$ 2**				
**Mean OHD MRD test dataset (range):**		**1.54 (1.29–1.80), 8/8 with MRD $\leq$ 2**				
**Overall OHD MRD (range):**		**1.46 (1.22–1.80), 11/11 with MRD $\leq$ 2**				
**Overall MRD training dataset (range):**		**1.32 (1.11–1.72), 15/15 with MRD $\leq$ 2**				
**Overall MRD test dataset (range):**		**1.52 (1.09–2.26), 23/24 with MRD $\leq$ 2**				
**Overall MRD (range):**		**1.44 (1.09–2.26), 38/39 with MRD $\leq$ 2**				

AS: CYP2D6 activity score, DES: desipramine, inf: infusion, iv: intravenous, MRD: mean relative deviation, NM: CYP2D6 normal metabolizer, OHD: 2-hydroxydesipramine, PM: CYP2D6 poor metabolizer, po: oral, q.d.: once daily, s.d.: single dose. Respective doses of desipramine base were calculated and incorporated in simulations. [a] Dose given as desipramine hydrochloride (desipramine base).

S2.7 Predicted Compared to Observed AUC_{last} and C_{max} Values

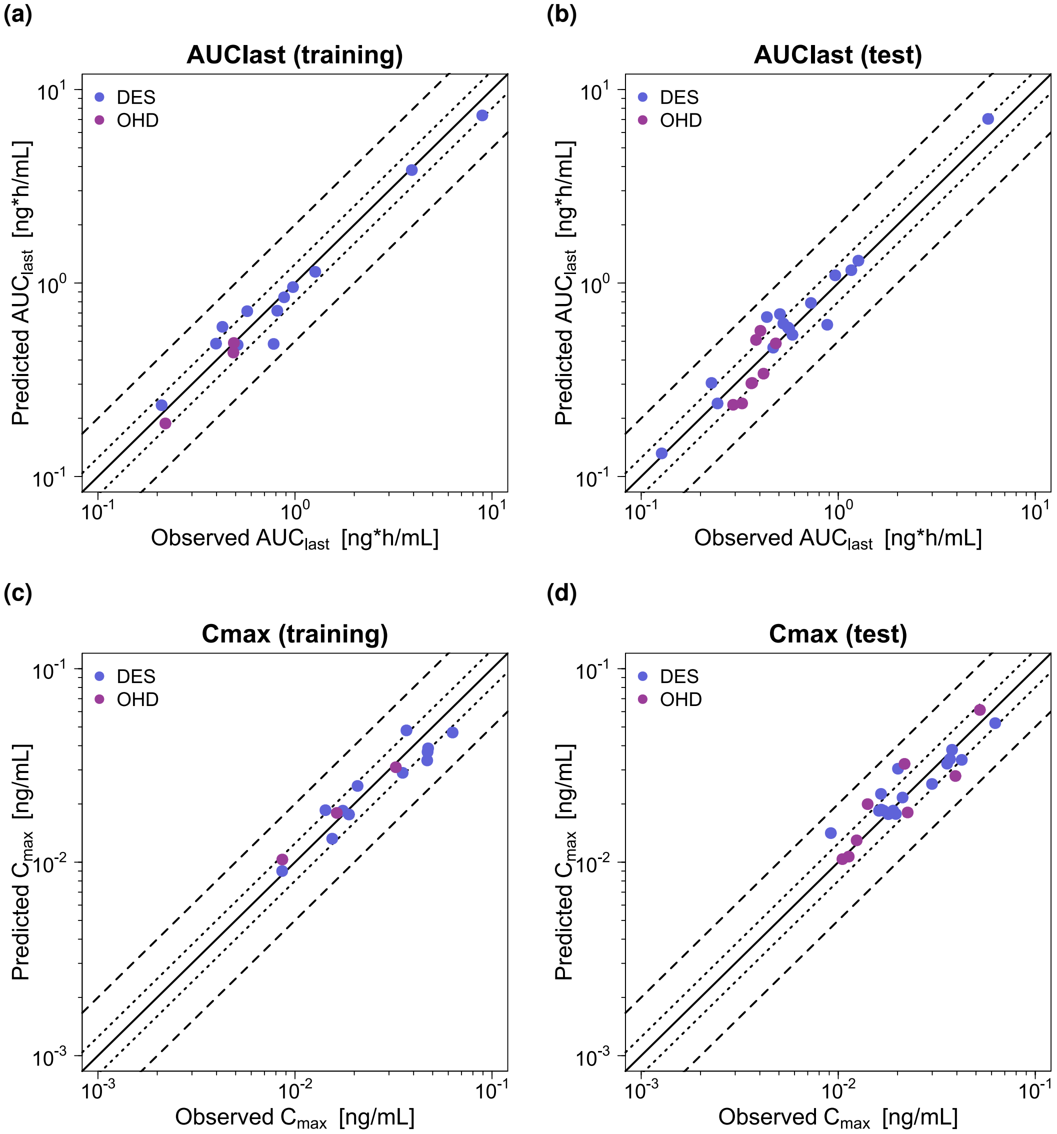

Figure S6: Goodness-of-fit plots comparing predicted and observed AUC_{last} and C_{max} values of desipramine and 2-hydroxydesipramine. The solid line marks the line of identity. Dotted lines indicate 1.25-fold, dashed lines indicate 2-fold deviation. AUC_{last}: area under the plasma concentration-time curve calculated between the first and last concentration measurement, C_{max}: maximum plasma concentration, DES: desipramine, OHD: 2-hydroxydesipramine.

S2.8 Geometric Mean Fold Errors of Predicted AUC_{last} and C_{max} Values

Table S4: Predicted and observed desipramine AUC_{last} and C_{max} values

			AUC_{last}			C_{max}						
**Dose [mg][a]**	**Route**	**t_{last} [h]**	**Pred [ng*h/mL]**	**Obs [ng*h/mL]**	**Pred/Obs**	**Pred [ng/mL]**	**Obs [ng/mL]**	**Pred/Obs**	**Phenotype/AS**	**Molecule**	**Dataset**	**Reference**
Desipramine												
50 (44)	s.d. iv 60 min inf	432	3836.62	3918.10	0.98	38.77	47.40	0.82	PM	DES	training	Brøsen 1988 [4]
50 (44)	s.d. iv 60 min inf	72	844.06	880.83	0.96	48.00	36.80	1.30	NM	DES	training	Brøsen 1988 [4]
50 (44)	s.d. iv 60 min inf	72	484.37	779.40	0.62	37.17	47.10	0.79	fast NM	DES	training	Brøsen 1988 [4]
25 (22)	s.d. po	96	952.76	976.94	0.98	13.23	15.46	0.86	PM	DES	training	Spina 1987 [5]
25 (22)	s.d. po	48	233.63	210.49	1.11	9.00	8.61	1.05	NM	DES	training	Spina 1987 [5]
50 (44)	s.d. po	72	593.82	428.44	1.39	18.57	14.28	1.30	-	DES	training	Aarnoutse 2005 [6]
50 (44)	s.d. po	48	304.54	227.47	1.34	14.14	9.17	1.54	-	DES	test	Bergstrom 1992 [8]
50 (44)	s.d. po	48	479.07	511.64	0.94	17.65	18.80	0.94	-	DES	training	Boni 2009 [9]
50 (44)	s.d. po	72	715.30	572.38	1.25	24.78	20.78	1.19	-	DES	training	Harris 2007 [10]
50 (44)	s.d. po	120	788.64	726.71	1.09	21.59	21.20	1.02	-	DES	test	Hynes 2015 [11]
50 (44)	s.d. po	48	486.19	397.47	1.22	18.46	17.50	1.05	-	DES	training	Madani 2002 [12]
50 (44)	s.d. po	120	608.65	879.27	0.69	17.75	19.60	0.91	-	DES	test	Nichols 2009 [13]
50 (44)	s.d. po	96	589.25	555.75	1.06	18.46	16.98	1.09	-	DES	test	Nichols 2013 [14]
50 (44)	s.d. po	120	617.53	526.28	1.17	18.43	16.10	1.14	-	DES	test	Patroneva 2008 [15]
50 (44)	s.d. po	72	540.00	585.45	0.92	18.48	19.01	0.97	-	DES	test	Reese 2008 [16]
50 (44)	s.d. po	72	581.05	563.27	1.03	18.69	16.53	1.13	-	DES	test	Sauer 2004 [17]
50 (44)	s.d. po	72	691.11	505.75	1.37	22.54	16.48	1.37	-	DES	test	Skinner 2003 [18]
50 (44)	q.d. po	24	463.40	467.65	0.99	34.09	36.90	0.92	-	DES	test	Alderman 1997 [19]
100 (88)	s.d. po	14	238.61	243.94	0.98	25.37	29.92	0.85	AS = 2	DES	test	Bergmann 2001 [20]
100 (88)	s.d. po	14	131.70	127.07	1.04	17.71	17.96	0.99	AS = 2.5	DES	test	Bergmann 2001 [20]
100 (88)	s.d. po	408	7352.29	8926.66	0.82	46.79	63.10	0.74	PM	DES	training	Brøsen 1986 [21]
100 (88)	s.d. po	72	1142.66	1267.39	0.90	33.58	46.90	0.72	NM	DES	training	Brøsen 1986 [21]
100 (88)	s.d. po	72	719.20	813.04	0.88	28.96	35.20	0.82	fast NM	DES	training	Brøsen 1986 [21]
100 (88)	s.d. po	240	7052.91	5765.12	1.22	52.33	62.60	0.84	PM	DES	test	Brøsen 1993 [22]
100 (88)	s.d. po	72	1305.38	1265.24	1.03	38.11	37.80	1.01	NM	DES	test	Brøsen 1993 [22]
100 (88)	s.d. po	48	667.02	434.27	1.54	30.42	20.10	1.51	fast NM	DES	test	Brøsen 1993 [22]
100 (88)	s.d. po	72	1165.41	1164.85	1.00	33.79	42.35	0.80	-	DES	test	Brøsen 1989 [23]
100 (88)	s.d. po	72	1096.07	965.43	1.14	32.29	35.70	0.90	-	DES	test	Spina 1995 [24]
**Mean DES GMFE training dataset (range):**			**1.18 (1.02–1.61), 12/12 with GMFE $\leq$ 2**			**1.22 (1.05–1.40), 12/12 with GMFE $\leq$ 2**						
**Mean DES GMFE test dataset (range):**			**1.16 (1.00–1.54), 16/16 with GMFE $\leq$ 2**			**1.17 (1.01–1.54), 16/16 with GMFE $\leq$ 2**						
**Overall DES GMFE (range):**			**1.17 (1.00–1.61), 28/28 with GMFE $\leq$ 2**			**1.19 (1.01–1.54), 28/28 with GMFE $\leq$ 2**						

AS: CYP2D6 activity score, AUC_{last}: area under the plasma concentration-time curve calculated between the first and last concentration measurement, C_{max}: maximum plasma concentration, DES: desipramine, GMFE: geometric mean fold error, inf: infusion, iv: intravenous, obs: observed, NM: CYP2D6 normal metabolizer, OHD: 2-hydroxydesipramine, PM: CYP2D6 poor metabolizer, po: oral, q.d.: once daily, s.d.: single dose, t_{last}: time of the last concentration measurement, -: not available. Respective doses of desipramine base were calculated and incorporated in simulations. [a] Dose given as desipramine hydrochloride (desipramine base).

Table S4: Predicted and observed desipramine AUC_{last} and C_{max} values *(continued)*

			AUC_{last}			C_{max}						
**Dose [mg][a]**	**Route**	**t_{last} [h]**	**Pred [ng*h/mL]**	**Obs [ng*h/mL]**	**Pred/Obs**	**Pred [ng/mL]**	**Obs [ng/mL]**	**Pred/Obs**	**Phenotype/AS**	**Molecule**	**Dataset**	**Reference**
2-Hydroxydesipramine												
50 (44)	s.d. po	48	188.12	219.93	0.86	10.32	8.63	1.20	-	OHD	training	Boni 2009 [9]
50 (44)	s.d. po	120	304.64	365.80	0.83	12.97	12.40	1.05	-	OHD	test	Hynes 2015 [11]
50 (44)	s.d. po	120	234.84	292.76	0.80	10.37	10.50	0.99	-	OHD	test	Nichols 2009 [13]
50 (44)	s.d. po	120	238.64	325.03	0.73	10.69	11.30	0.95	-	OHD	test	Patroneva 2008 [15]
100 (88)	s.d. po	24	340.07	417.57	0.81	27.89	39.33	0.71	AS = 2	OHD	test	Bergmann 2001 [20]
100 (88)	s.d. po	24	508.29	382.58	1.33	61.22	52.34	1.17	AS = 2.5	OHD	test	Bergmann 2001 [20]
100 (88)	s.d. po	72	437.29	487.03	0.90	17.98	16.30	1.10	NM	OHD	training	Brøsen 1986 [21]
100 (88)	s.d. po	48	490.01	489.54	1.00	30.96	32.50	0.95	fast NM	OHD	training	Brøsen 1986 [21]
100 (88)	s.d. po	72	487.49	482.02	1.01	19.96	14.10	1.42	NM	OHD	test	Brøsen 1993 [22]
100 (88)	s.d. po	72	566.50	402.70	1.41	32.22	21.70	1.48	fast NM	OHD	test	Brøsen 1993 [22]
100 (88)	s.d. po	30	303.09	362.43	0.84	18.07	22.50	0.80	-	OHD	test	Brøsen 1989 [23]
**Mean OHD GMFE training dataset (range):**			**1.09 (1.00–1.17), 3/3 with GMFE ≤ 2**			**1.12 (1.05–1.20), 3/3 with GMFE ≤ 2**						
**Mean OHD GMFE test dataset (range):**			**1.25 (1.01–1.41), 8/8 with GMFE ≤ 2**			**1.23 (1.01–1.48), 8/8 with GMFE ≤ 2**						
**Overall OHD GMFE (range):**			**1.21 (1.00–1.41), 11/11 with GMFE ≤ 2**			**1.20 (1.01–1.48), 11/11 with GMFE ≤ 2**						
**Mean GMFE training dataset (range):**			**1.16 (1.00–1.61), 15/15 with GMFE ≤ 2**			**1.20 (1.05–1.40), 15/15 with GMFE ≤ 2**						
**Mean GMFE test dataset (range):**			**1.19 (1.00–1.54), 24/24 with GMFE ≤ 2**			**1.19 (1.01–1.54), 24/24 with GMFE ≤ 2**						
**Overall GMFE (range):**			**1.18 (1.00–1.61), 39/39 with GMFE ≤ 2**			**1.19 (1.01–1.54), 39/39 with GMFE ≤ 2**						

AS: CYP2D6 activity score, AUC_{last}: area under the plasma concentration-time curve calculated between the first and last concentration measurement, C_{max}: maximum plasma concentration, DES: desipramine, GMFE: geometric mean fold error, inf: infusion, iv: intravenous, obs: observed, NM: CYP2D6 normal metabolizer, OHD: 2-hydroxydesipramine, PM: CYP2D6 poor metabolizer, po: oral, q.d.: once daily, s.d.: single dose, t_{last}: time of the last concentration measurement, -: not available. Respective doses of desipramine base were calculated and incorporated in simulations. [a] Dose given as desipramine hydrochloride (desipramine base).

S2.9 Sensitivity Analysis

Table S5: Parameters evaluated during desipramine and 2-hydroxydesipramine sensitivity analysis

	Desipramine		2-Hydroxydesipramine	
**Parameter**	**Value**	**Source**	**Value**	**Source**
pK_a (base 1)	2.84	Literature	4.51	Literature
pK_a (base 2)	10.02	Literature	9.90	Literature
pK_a (acid)	-	-	10.63	Literature
Solubility (pH 6.5) [mg/L]	214.29	Literature	282.39	Literature
Lipophilicity	3.52	Optimized	2.33	Optimized
$f_{u,p}$ [%]	14	Literature	12	Literature
CYP2D6 (DES $\rightarrow$ OHD) K_M [µmol/L]	0.73	Literature	-	-
CYP2D6 (DES $\rightarrow$ OHD) k_{cat} [1/min]	5.03	Optimized	-	-
CL_{hep} [1/min]	0.54	Optimized	9.93	Optimized
GFR fraction	1	Assumed	1	Assumed
Intestinal permeability [cm/min]	$1.14 \cdot 10^{-5}$	Optimized	-	-

CL_{hep}: hepatic metabolic clearance, CYP: cytochrome P450, DES: desipramine, $f_{u,p}$: fraction unbound plasma, k_{cat}: catalytic or transport rate constant, K_i: concentration for 50% inhibition (competitive), K_M: Michaelis-Menten constant, OHD: 2-hydroxydesipramine, pK_a: acid dissociation constant, -: parameter not included.

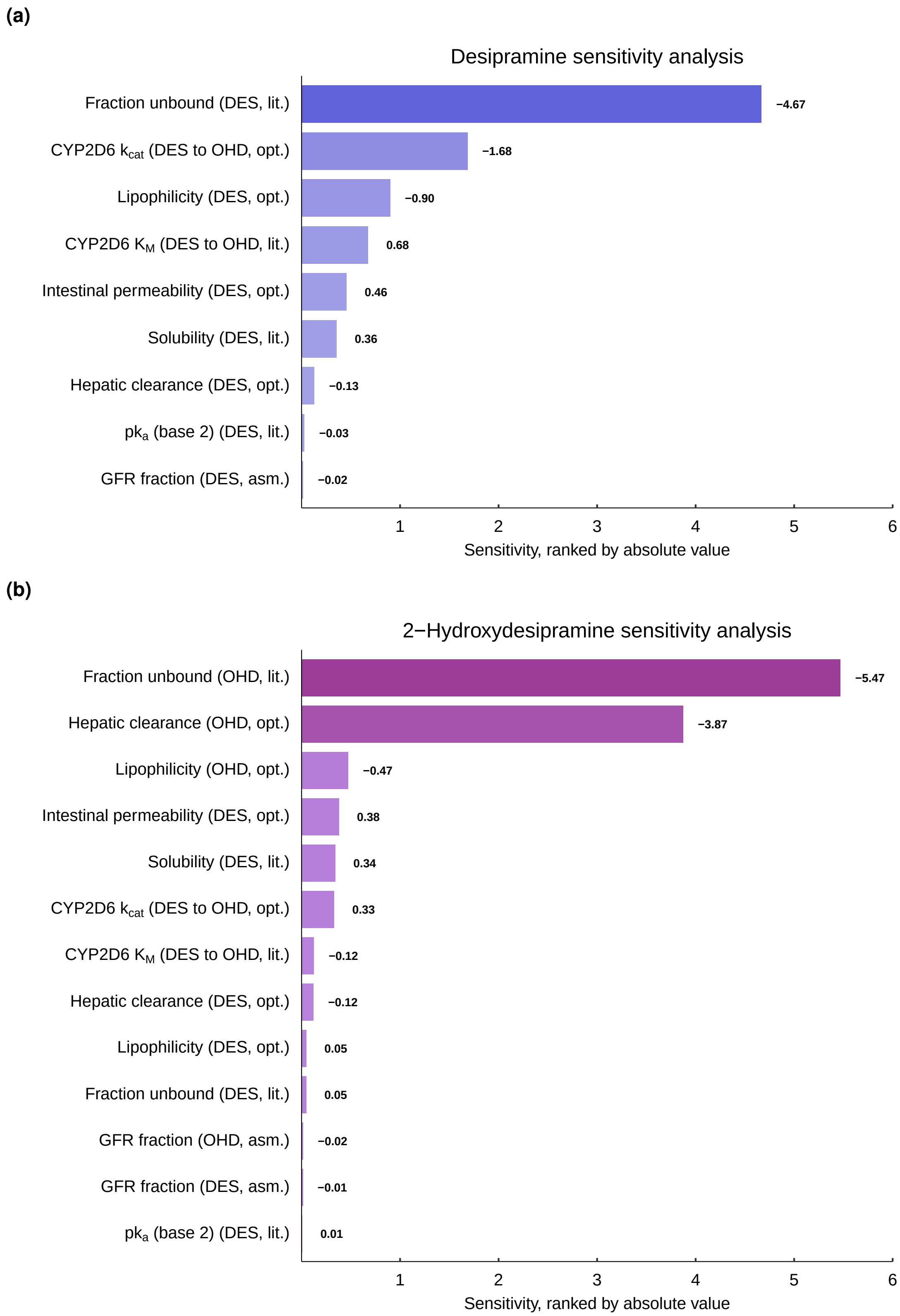

Figure S7: Local sensitivity analysis results of the desipramine PBPK model ((a) desipramine and (b) 2-hydroxydesipramine), determined as relative change of $AUC_{0\text{-}24h}$ at steady state in a once daily regimen of 50 mg desipramine hydrochloride. Asm.: assumed, CYP: cytochrome P450, DES: desipramine, k_{cat}: catalytic or transport rate constant, K_M: Michaelis-Menten constant, lit.: literature value, OHD: 2-hydroxydesipramine, opt.: optimized, pK_a: acid dissociation constant.

S3 Desipramine DGI Model Results

Desipramine DGI modeling was performed using five DGI studies providing a total of thirteen desipramine and six 2-hydroxydesipramine plasma-concentration-time profiles. Comprehensive information on the DGI studies used is given in Table S6.

Predicted and observed plasma concentration-time profiles of desipramine and 2-hydroxydesipramine are shown in semilogarithmic and linear representations in Sections S3.2–S3.3. Overall, predicted AUC_{last} and C_{max} ratios were in good agreement with observed DGI ratios, highlighting the good performance of the desipramine DGI model with 8/11 DGI AUC_{last} and 8/11 C_{max} ratios within the prediction success limits proposed by Guest et al. [2] as depicted in Figure S12. All predicted and observed DGI AUC_{last} and C_{max} ratios are listed in Table S7.

S3.1 Clinical Studies

Table S6: Clinical study data used for desipramine DGI model development

Dose [mg][a]	Route	n	Population[b]	Fem. [%]	Age [years]	Weight [kg]	BMI [kg/m²]	Phenotype/AS	Molecule	Reference
Intravenous										
50 (44)	s.d. iv 60 min inf	3	European [3]	33	29 (24–35)	-	-	PM	DES	Brøsen 1988 [4]
50 (44)	s.d. iv 60 min inf	4	European [3]	75	29.75 (22–37)	-	-	NM	DES	Brøsen 1988 [4]
50 (44)	s.d. iv 60 min inf	4	European [3]	50	23.75 (22–27)	-	-	fast NM	DES	Brøsen 1988 [4]
Oral										
25 (22)	s.d. po	6	European [3]	21	(27–51)	-	-	PM	DES	Spina 1987 [5]
25 (22)	s.d. po	8	European [3]	21	(27–51)	-	-	NM	DES	Spina 1987 [5]
100 (88)	s.d. po	6	European [3]	83	26.8 (24–33)	-	-	AS = 2	DES, OHD	Bergmann 2001 [20]
100 (88)	s.d. po	6	European [3]	33	36.7 (22–54)	-	-	AS = 2.5	DES, OHD	Bergmann 2001 [20]
100 (88)	s.d. po	6	European [3]	50	28.8 (23–34)	-	-	PM	DES	Brøsen 1986 [21]
100 (88)	s.d. po	6	European [3]	50	28.3 (21–36)	-	-	NM	DES, OHD	Brøsen 1986 [21]
100 (88)	s.d. po	6	European [3]	50	24.1 (21–30)	-	-	fast NM	DES, OHD	Brøsen 1986 [21]
100 (88)	s.d. po	8	European [3]	0	(22–24)	-	-	PM	DES	Brøsen 1993 [22]
100 (88)	s.d. po	5	European [3]	0	(22–24)	-	-	NM	DES, OHD	Brøsen 1993 [22]
100 (88)	s.d. po	4	European [3]	0	(22–24)	-	-	fast NM	DES, OHD	Brøsen 1993 [22]

AS: CYP2D6 activity score, BMI: body mass index, calc: calculated, DES: desipramine, DGI: drug-gene interaction, fem: females, inf: infusion, iv: intravenous, n: number of study participants, NM: CYP2D6 normal metabolizer, OHD: 2-hydroxydesipramine, PM: CYP2D6 poor metabolizer, po: oral, -: not available. Values are given as mean (range). Respective doses of desipramine base were calculated and incorporated in simulations. [a] Dose given as desipramine hydrochloride (desipramine base). [b] Population used in simulations.

S3.2 Plasma Concentration-Time Profiles (Semilogarithmic Representation)

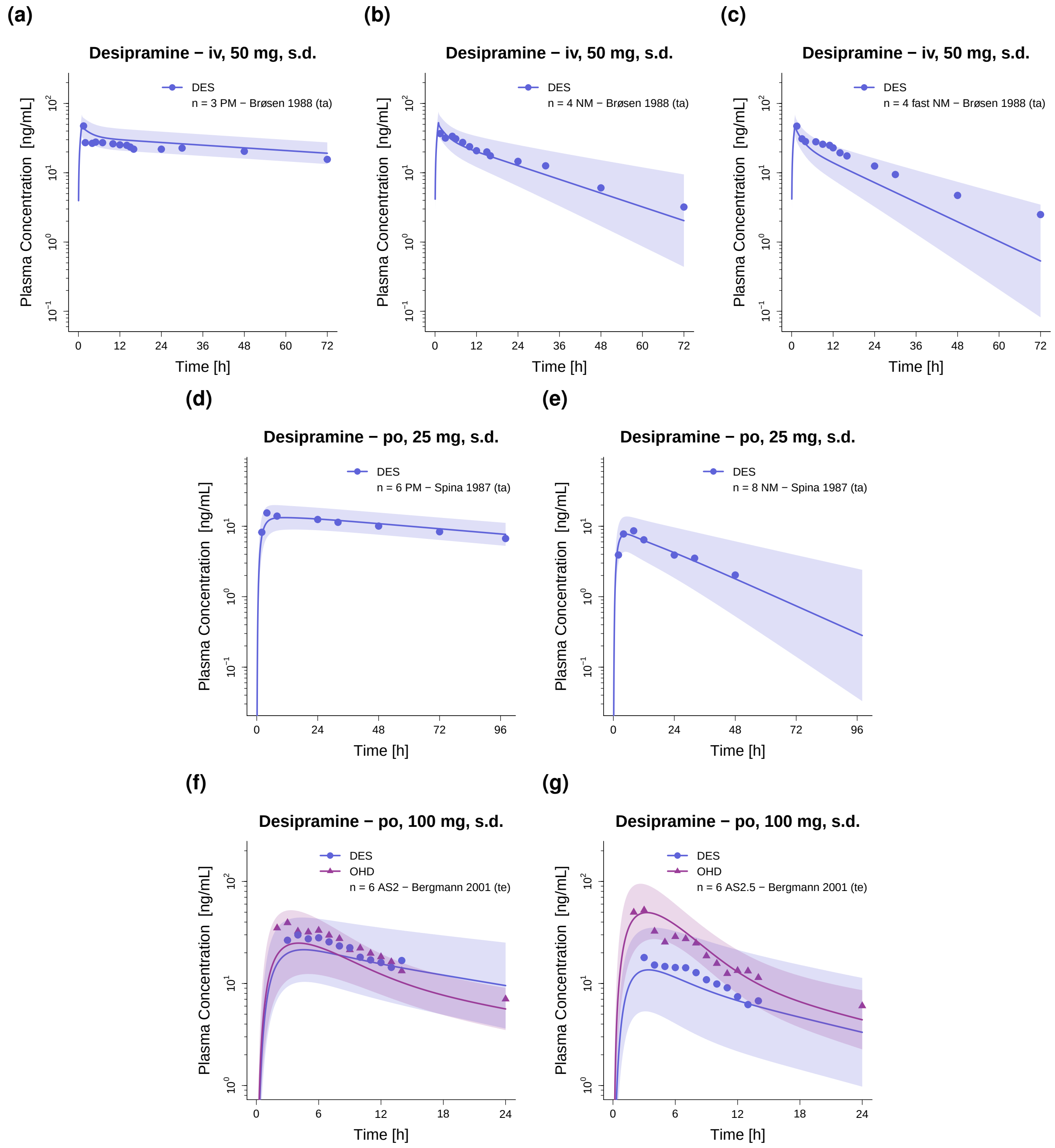

Figure S8: Predicted compared to observed plasma concentration-time profiles of desipramine and 2-hydroxydesipramine at varying CYP2D6 activity levels. Population predicted (1000 individuals) geometric means are shown as lines, corresponding geometric standard deviations as shaded areas and observed data as dots/triangles [4, 5, 20]. AS: CYP2D6 activity score, DES: desipramine, iv: intravenous, n: number of study participants, NM: CYP2D6 normal metabolizer, OHD: 2-hydroxydesipramine, PM: CYP2D6 poor metabolizer, po: oral, s.d.: single dose, ta: training dataset, te: test dataset.

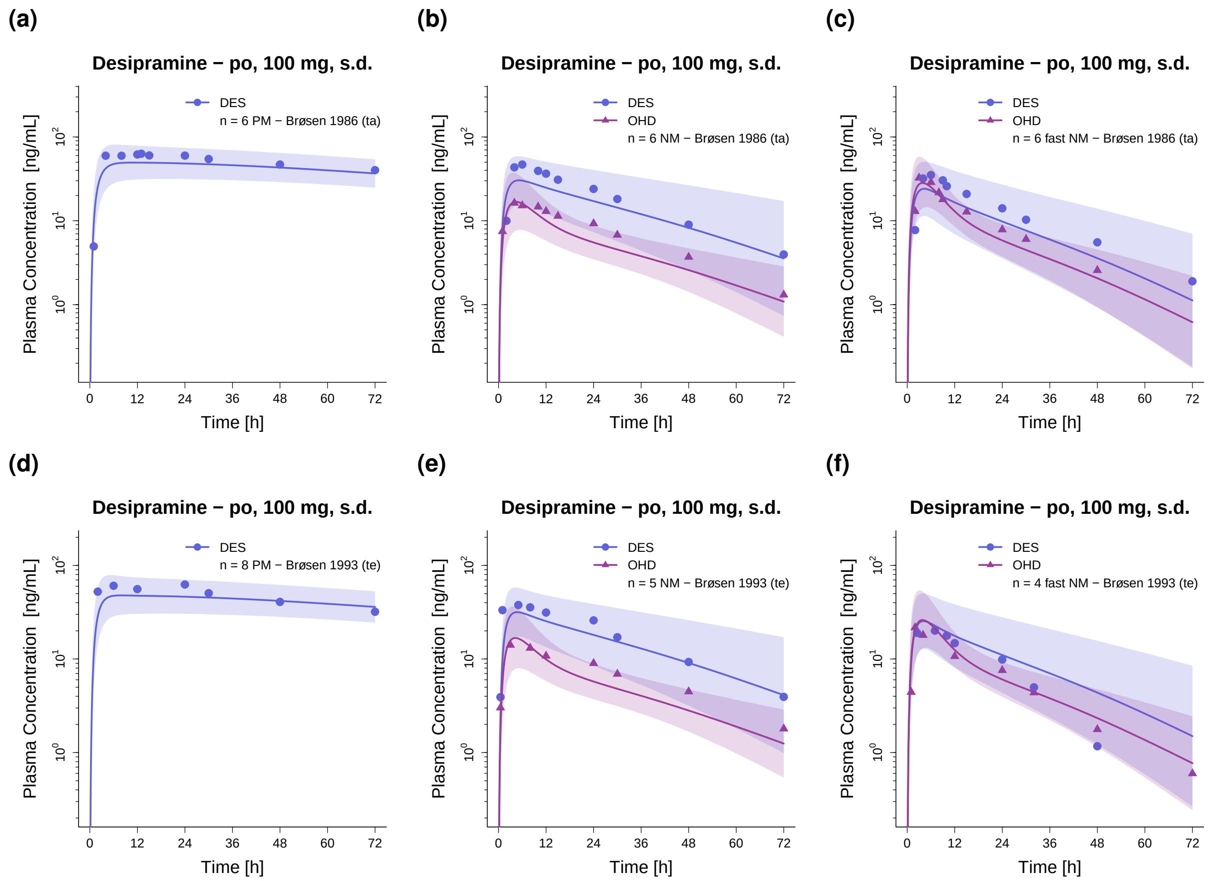

Figure S9: Predicted compared to observed plasma concentration-time profiles of desipramine and 2-hydroxydesipramine at varying CYP2D6 activity levels. Population predicted (1000 individuals) geometric means are shown as lines, corresponding geometric standard deviations as shaded areas and observed data as dots/triangles [21, 22]. AS: CYP2D6 activity score, DES: desipramine n: number of study participants, NM: CYP2D6 normal metabolizer, OHD: 2-hydroxydesipramine, PM: CYP2D6 poor metabolizer, po: oral, s.d.: single dose, ta: training dataset, te: test dataset.

S3.3 Plasma Concentration-Time Profiles (Linear Representation)

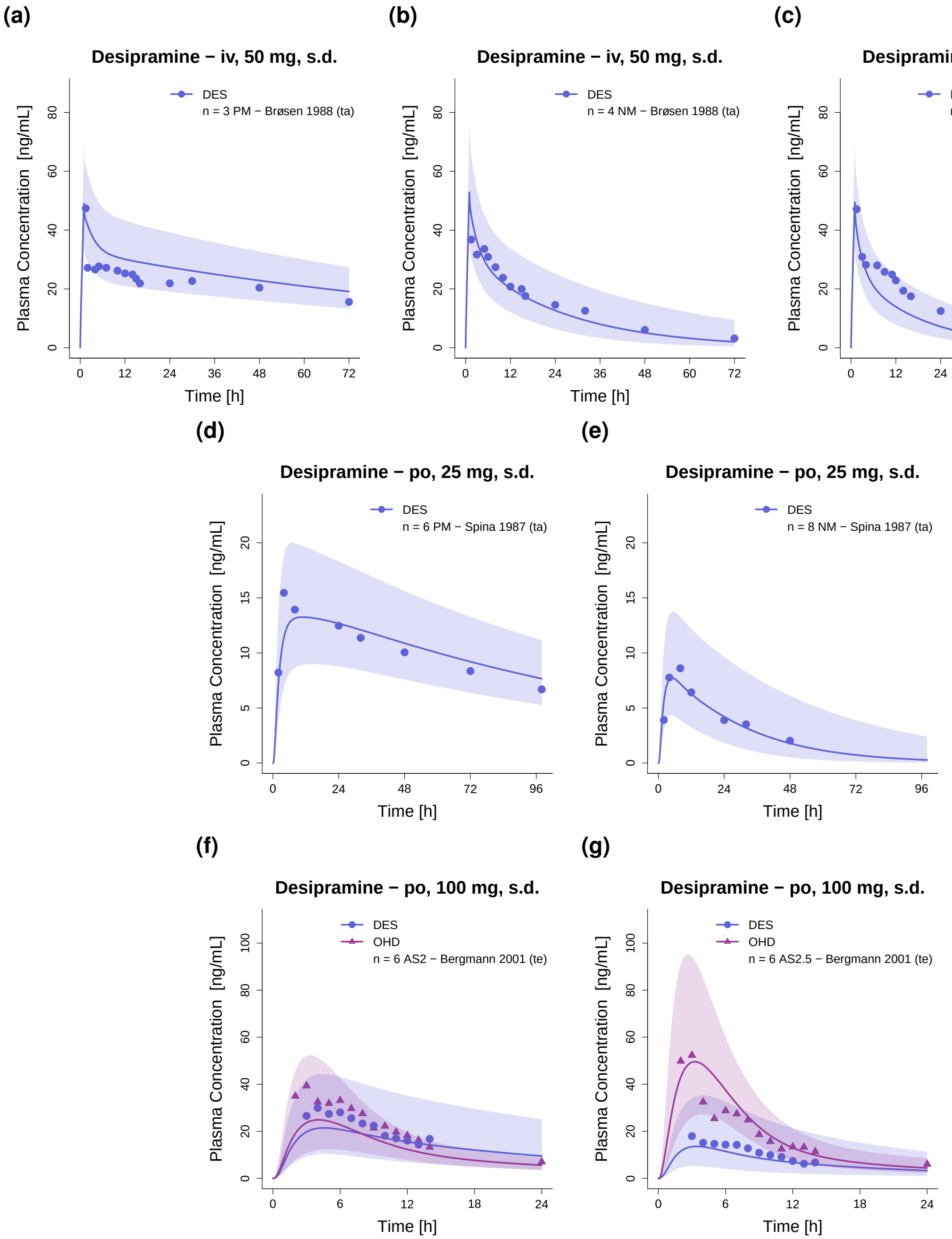

Figure S10: Predicted compared to observed plasma concentration-time profiles of desipramine and 2-hydroxydesipramine at varying CYP2D6 activity levels. Population predicted (1000 individuals) geometric means are shown as lines, corresponding geometric standard deviations as shaded areas and observed data as dots/triangles [4, 5, 20]. AS: CYP2D6 activity score, DES: desipramine, iv: intravenous, n: number of study participants, NM: CYP2D6 normal metabolizer, OHD: 2-hydroxydesipramine, PM: CYP2D6 poor metabolizer, po: oral, s.d.: single dose, ta: training dataset, te: test dataset.

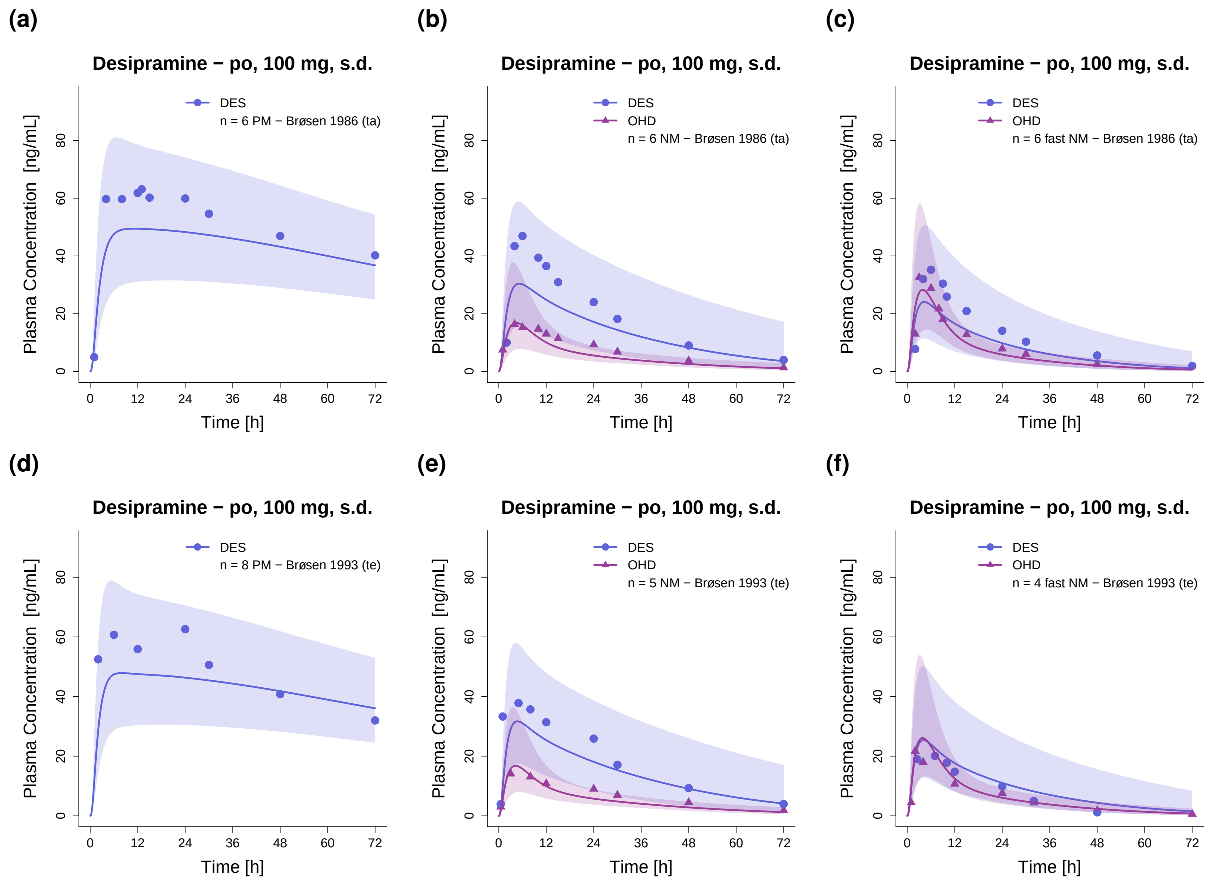

Figure S11: Predicted compared to observed plasma concentration-time profiles of desipramine and 2-hydroxydesipramine at varying CYP2D6 activity levels. Population predicted (1000 individuals) geometric means are shown as lines, corresponding geometric standard deviations as shaded areas and observed data as dots/triangles [21, 22]. AS: CYP2D6 activity score, DES: desipramine n: number of study participants, NM: CYP2D6 normal metabolizer, OHD: 2-hydroxydesipramine, PM: CYP2D6 poor metabolizer, po: oral, s.d.: single dose, ta: training dataset, te: test dataset.

S3.4 DGI AUC_{last} and C_{max} Ratios

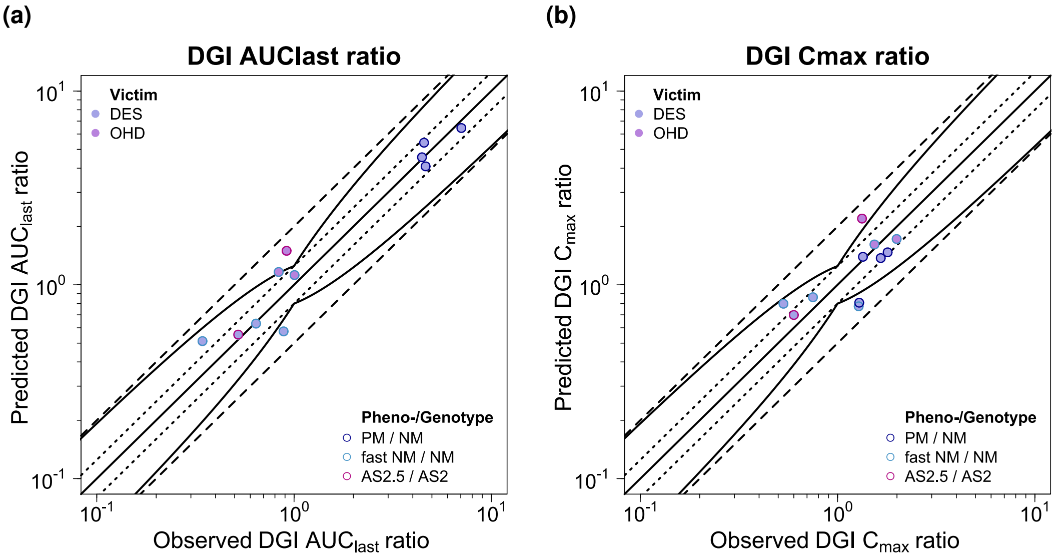

Figure S12: Goodness-of-fit plots comparing predicted and observed DGI AUC_{last} and C_{max} ratios for victim drug desipramine. The solid line marks the line of identity. Dotted lines indicate 1.25-fold, dashed lines indicate 2-fold deviation. Prediction success limits proposed by Guest et al. [2] are shown as curved lines (including 20% variability). AS: CYP2D6 activity score, AUC_{last}: area under the plasma concentration-time curve calculated between the first and last concentration measurement, C_{max}: maximum plasma concentration, DES: desipramine, DGI: drug-gene interaction, NM: CYP2D6 normal metabolizer, OHD: 2-hydroxydesipramine, PM: CYP2D6 poor metabolizer.

S3.4.1 Geometric Mean Fold Errors of Predicted DGI AUC_{last} and C_{max} Ratios

Table S7: Predicted and observed DGI AUC_{last} and C_{max} ratios involving **desipramine** as victim drug

			**DGI AUC_{last} ratio**			**DGI C_{max} ratio**					
**Dose [mg][a]**	**Route**	**t_{last} [h]**	**Pred**	**Obs**	**Pred/Obs**	**Pred**	**Obs**	**Pred/Obs**	**Phenotype/AS**	**Molecule**	**Reference**
Intravenous											
50 (44)	s.d. iv 60 min inf	432 / 72	4.55	4.45	1.02	0.81	1.29	0.63	PM / NM	DES	Brøsen 1988 [4]
50 (44)	s.d. iv 60 min inf	72 / 72	0.57	0.88	0.65	0.77	1.28	0.60	fast NM / NM	DES	Brøsen 1988 [4]
**Mean GMFE (range):**			**1.28 (1.02–1.54), 2/2 with GMFE $\leq$ 2**			**1.62 (1.59–1.65), 2/2 with GMFE $\leq$ 2**					
Oral											
25 (22)	s.d. po	96 / 48	4.08	4.64	0.88	1.47	1.80	0.82	PM / NM	DES	Spina 1987 [5]
100 (88)	s.d. po	14 / 14	0.55	0.52	1.06	0.70	0.60	1.16	AS2.5 / AS2	DES	Bergmann 2001 [20]
100 (88)	s.d. po	24 / 24	1.49	0.92	1.63	2.19	1.33	1.65	AS2.5 / AS2	OHD	Bergmann 2001 [20]
100 (88)	s.d. po	408 / 72	6.43	7.04	0.91	1.39	1.35	1.04	PM / NM	DES	Brøsen 1986 [21]
100 (88)	s.d. po	72 / 72	0.63	0.64	0.98	0.86	0.75	1.15	fast NM / NM	DES	Brøsen 1986 [21]
100 (88)	s.d. po	48 / 72	1.12	1.01	1.11	1.72	1.99	0.86	fast NM / NM	OHD	Brøsen 1986 [21]
100 (88)	s.d. po	240 / 72	5.40	4.56	1.19	1.37	1.66	0.83	PM / NM	DES	Brøsen 1993 [22]
100 (88)	s.d. po	48 / 72	0.51	0.34	1.49	0.80	0.53	1.50	fast NM / NM	DES	Brøsen 1993 [22]
100 (88)	s.d. po	72 / 72	1.16	0.84	1.39	1.61	1.54	1.05	fast NM / NM	OHD	Brøsen 1993 [22]
**Mean GMFE (range):**			**1.24 (1.02–1.63), 9/9 with GMFE $\leq$ 2**			**1.24 (1.04–1.65), 9/9 with GMFE $\leq$ 2**					
**Overall GMFE (range):**			**1.24 (1.02–1.63), 11/11 with GMFE $\leq$ 2**			**1.31 (1.04–1.65), 11/11 with GMFE $\leq$ 2**					

AS: CYP2D6 activity score, AUC_{last}: area under the plasma concentration-time curve calculated between the first and last concentration measurement, C_{max}: maximum plasma concentration, DES: desipramine, DGI: drug-gene interaction, GMFE: geometric mean fold error, inf: infusion, iv: intravenous, NM: CYP2D6 normal metabolizer, obs: observed, OHD: 2-hydroxydesipramine, PM: CYP2D6 poor metabolizer, po: oral, pred: predicted, s.d.: single dose, t_{last}: time of the last concentration measurement. Respective doses of desipramine base were calculated and incorporated in simulations. [a] Dose given as desipramine hydrochloride (desipramine base).

S4 DD(G)I Network Development

S4.1 System-Dependent Parameters

Table S8: Relevant enzymes, transporters and binding proteins

			Reference concentration [µmol/L]	
**Protein**	**Relevant model(s)**	**Highest expression**	**Mean[a]**	**GSD**
Enzymes				
AADAC	KET, RIF	Liver [34]	1.00[b] [35]	1.40[c]
CYP1A2	FLV, MEX	Liver [36]	1.80 [37]	1.63 [38]
CYP2B6	BUP, CBZ, CLO	Liver [34]	1.56 [37]	1.56 [38]
CYP2C8	CBZ	Liver [36]	2.56 [37]	2.05 [38]
CYP2C19	ATO, BUP, OME	Liver [36]	0.76 [37]	1.80 [38]
CYP2D6	ATO, DES, DEX, CLO, FLV, MET, MEX, PAR, RIS	Liver [36]	0.40 [37]	2.49 [38]
CYP3A4	ALP, CBZ, CLA, CLO, DEX, ERY, ITR, KET, MET, MID, OME, PAR, QUI, RIS, VER	Liver [36]	4.32 [37]	1.18 liver, 1.46 int. [38]
EPHX1	CBZ	Liver [34]	1.00[b] [35]	1.40[c]
FMO3	KET	Liver [39]	1.00[b] [35]	1.40[c]
HSD11B1	BUP	Liver [39]	1.00[b] [35]	1.40[c]
UGT1A4	KET, MID	Liver [34]	2.32[d] [40, 41]	1.51 [40]
UGT2B7	CBZ, BUP	Kidney [42]	2.78 [43]	1.60 [38]
UGT2B15	DEX	Liver [34]	2.48[d] [40, 41]	1.26 [40]
Transporters				
MATE1	CIM	Kidney [44, 45]	0.13[e] [41, 46]	1.53 [46]
OAT3	CIM	Kidney [47]	0.09[e] [41, 46]	1.53 [46]
OATP1B1	ERY, RIF	Liver [47]	0.07[f] [48]	1.54 [48]
OCT1	CIM	Liver [39]	0.16[f] [48, 49]	1.50 [49]
P-gp	DIG, KET, QUI, RIF, RIS, VER	Duodenum mucusa, Upper jejunum mucosa, Lower jejunum mucosa, Upper ileum mucosa Lower ileum mucosa [47]	1.41[g] [50]	1.60 [48]
Binding proteins				
ATP1A2	DIG	Brain [39]	0.48[g] [50]	1.40[c]
BP[h]	BUP	Brain [42]	1.00[b] [35]	1.40[c]
GABRG2	MID	Brain [39]	1.04 [51]	1.40[c]

AADAC: arylacetamide deacetylase, ALP: alprazolam, ATO: atomoxetine, ATP1A2: ATPase Na^+/K^+ transporting subunit alpha 2, BP: binding partner, BUP: bupropion, CBZ: carbamazepine, CIM: cimetidine, CLA: clarithromycin, CLO: *E*-clomiphene, CYP: cytochrome P450, DES: desipramine, DEX: dextromethorphan, DIG: digoxin, EPHX: epoxide hydrolase, ERY: erythromycin, FLV: fluvoxamine, FMO: flavin-containing monooxygenase, GABRG: gamma-aminobutyric acid (GABA) A receptor gamma, GSD: geometric standard deviation, HSD: hydroxysteroid dehydrogenase, int: intestine, ITR: itraconazole, KET: ketoconazole, MATE: multidrug and toxin extrusion protein, MET: metoprolol, MEX: mexiletine, MID: midazolam, OAT: organic anion transporter, OATP: organic anion transporting polypeptide, OCT: organic cation transporter, OME: omeprazole, PAR: paroxetine, P-gp: P-glycoprotein, QUI: quinidine, RIF: rifampicin, RIS: risperidone, UGT: uridine 5'-diphospho-glucuronosyltransferase, VER: verapamil. [a] In the tissue of highest expression. [b] If no information was available, the mean reference concentration was set to 1.00 µmol/L and the catalytic rate constant was optimized according to [35]. [c] A moderate variability of 35% CV was assumed (= 1.40 GSD). [d] Protein per mg microsomal protein x 40 mg microsomal protein per g liver [41]. [e] Transporter per mg membrane protein x 26.2 mg human kidney microsomal protein per g kidney [41]. [f] Transporter per mg membrane protein x 37.0 mg membrane protein per g liver [48]. [g] Optimized by Hanke et al. [50]. [h] Binding partner representing various neurotransmitter transporters (therapeutic target).

Table S9: Expression data of relevant enzymes

	AADAC	CYP1A2	CYP2B6	CYP2C8	CYP2C19	CYP2D6	CYP3A4
Properties							
**Localization**	Intracellular	Intracellular	Intracellular	Intracellular	Intracellular	Intracellular	Intracellular
**Half-life liver/intestine [h][a]**	36/23	39/23	32/23	23/23	26/23	51/23	36/23 [52, 53]
Relative expression in various organs and tissues [%]							
**Data source**	RT-PCR [34]	RT-PCR [36]	RT-PCR [34]	RT-PCR [36]	RT-PCR [36, 54]	RT-PCR [36]	RT-PCR [36]
**Blood Cells**	0	0	0	0	0	0	0
**Plasma**	0	0	0	0	0	0	0
**Bone**	0	0	0	0	0	0	0
**Brain**	0	0	0	0	0	1	0
**Fat**	0	0	0	0	0	0	0
**Gonads**	0	0	1	1	0	77	0
**Heart**	0	0	0	0	0	0	0
**Kidney**	0	0	10	0	0	2	1
**Liver Periportal**	100	100	100	100	100	100	100
**Liver Pericentral**	100	100	100	100	100	100	100
**Lung**	3	0	60	0	0	2	0
**Muscle**	0	0	0	0	0	0	0
**Pancreas**	15	0	0	0	0	0	0
**Skin**	0	0	0	0	0	0	0
**Spleen**	0	0	0	0	0	0	0
**Duodenum mucosa**	25	0	7	0	2	9	7
**Upper jejunum musoca**	25	0	7	0	1	9	7
**Lower jejunum mucosa**	25	0	7	0	1	9	7
**Upper ileum mucosa**	25	0	7	0	1	9	7
**Lower ileum mucosa**	25	0	7	0	1	9	7
**Colon ascendens mucosa**	0	0	0	0	0	0	0
**Colon transversum mucosa**	0	0	0	0	0	0	0
**Colon descendens mucosa**	0	0	0	0	0	0	0
**Colon sigmoid mucosa**	0	0	0	0	0	0	0
**Stomach non-mucosal tissue**	8	0	0	0	0	0	0
**Small intestine non-mucosal tissue**	25	0	7	0	1	9	7
**Large intestine non-mucosal tissue**	0	0	0	0	0	0	0

AADAC: arylacetamide deacetylase, Array: microarray expression profile, CYP: cyrochrome P450, EPHX: epoxide hydrolase, EST: expressed sequence tag, FMO: flavin-containing monooxygenase, HSD: hydroxysteroid dehydrogenase, RT-PCR: reverse transcription-polymerase chain reaction measured expression profile, UGT: uridine 5'-diphospho-glucuronosyltransferase. [a] Information from PK-Sim® expression database.

Table S9: Expression data of relevant enzymes (*continued*)

	EPHX1	FMO3	HSD11B1	UGT1A4	UGT2B7	UGT2B15
Properties						
**Localization**	Intracellular	Intracellular	Intracellular	Intracellular	Intracellular	Intracellular
**Half-life liver/intestine [h][a]**	36/23	36/23	36/23	36/23	36/23	36/23
Relative expression in various organs and tissues [%]						
**Data source**	RT-PCR [34]	Array [39]	Array [39]	RT-PCR [34]	EST [42]	RT-PCR [34]
**Blood Cells**	1	0	0	0	0	0
**Plasma**	1	0	0	0	0	0
**Bone**	2	1	4	0	0	0
**Brain**	4	0	9	0	8	0
**Fat**	0	0	0	0	0	0
**Gonads**	18	0	21	0	13	0
**Heart**	12	0	0	0	0	0
**Kidney**	15	0	7	0	100	0
**Liver Periportal**	100	100	100	100	23	100
**Liver Pericentral**	100	100	100	100	23	100
**Lung**	14	2	8	0	0	0
**Muscle**	36	1	6	0	0	0
**Pancreas**	10	0	7	0	0	2
**Skin**	0	0	30	0	3	0
**Spleen**	6	0	10	0	0	0
**Duodenum mucosa**	6	0	9	0	4	0
**Upper jejunum musoca**	6	0	9	0	4	0
**Lower jejunum mucosa**	6	0	9	0	4	0
**Upper ileum mucosa**	6	0	9	0	4	0
**Lower ileum mucosa**	6	0	9	0	4	0
**Colon ascendens mucosa**	4	0	4	0	0	0
**Colon transversum mucosa**	4	0	4	0	0	0
**Colon descendens mucosa**	4	0	4	0	0	0
**Colon sigmoid mucosa**	4	0	4	0	0	0
**Stomach non-mucosal tissue**	5	1	4	0	13	3
**Small intestine non-mucosal tissue**	6	0	9	0	4	0
**Large intestine non-mucosal tissue**	4	0	4	0	0	0

AADAC: arylacetamide deacetylase, Array: microarray expression profile, CYP: cyrochrome P450, EPHX: epoxide hydrolase, EST: expressed sequence tag, FMO: flavin-containing monooxygenase, HSD: hydroxysteroid dehydrogenase, RT-PCR: reverse transcription-polymerase chain reaction measured expression profile, UGT: uridine 5'-diphospho-glucuronosyltransferase. [a] Information from PK-Sim® expression database.

Table S10: Expression data of relevant transporters and binding proteins

	MATE1	OAT3	OATP1B1	OCT1	P-gp	ATP1A2	BP[a]	GABRG2
Properties								
**Localization**	Cell membrane	Cell membrane	Cell membrane	Cell membrane	Cell membrane	Interstitial	Interstitial	Interstitial
**Direction**	Efflux	Influx	Influx	Influx	Efflux	n.a.	n.a.	n.a.
**Half-life liver/intestine [h][b]**	n.a./n.a.	n.a./n.a.	36/23	36/23	36/23	36/23	36/23	36/23
Relative expression in various organs and tissues [%]								
**Data source**	[44, 45]	RT-PCR [47]	RT-PCR [47]	Array [39]	RT-PCR [47, 50]	Array [39]	EST [42]	[51]
**Blood Cells**	0	0	0	0	0	0	0	0
**Plasma**	0	0	0	0	0	0	0	0
**Bone**	0	0	0	2	2	1	0	0
**Brain**	0	0	0	1 (BBB)	8 (BBB)	100	100	100
**Fat**	0	0	0	0	0	0	0	0
**Gonads**	0	0	1	0	2	5	28	0
**Heart**	0	0	0	1	4	32	0	0
**Kidney**	100 (apical)	100 (basolateral)	0	3 (basolateral)	71 (apical)	2	42	0
**Liver Periportal**	0	0	100 (basolateral)	100 (basolateral)	19 (apical)	2	0	0
**Liver Pericentral**	0	0	100 (basolateral)	100 (basolateral)	19 (apical)	2	0	0
**Lung**	0	0	0	1	7	3	0	0
**Muscle**	0	0	0	4	1	70	12	0
**Pancreas**	0	0	0	1	1	1	0	0
**Skin**	0	0	0	1	0	4	0	0
**Spleen**	0	0	0	0	7	1	0	0
**Duodenum mucosa**	0	0	0	2 (apical)	100 (apical)	5	18	0
**Upper jejunum musoca**	0	0	0	2 (apical)	100 (apical)	5	18	0
**Lower jejunum mucosa**	0	0	0	2 (apical)	100 (apical)	5	18	0
**Upper ileum mucosa**	0	0	0	2 (apical)	100 (apical)	5	18	0
**Lower ileum mucosa**	0	0	0	2 (apical)	100 (apical)	5	18	0
**Colon ascendens mucosa**	0	0	0	0	40 (apical)	8	0	0
**Colon transversum mucosa**	0	0	0	0	40 (apical)	8	0	0
**Colon descendens mucosa**	0	0	0	0	40 (apical)	8	0	0
**Colon sigmoid mucosa**	0	0	0	0	40 (apical)	8	0	0
**Stomach non-mucosal tissue**	0	0	0	1	3	3	0	0
**Small intestine non-mucosal tissue**	0	0	0	2	28	5	18	0
**Large intestine non-mucosal tissue**	0	0	0	3	11	8	0	0

Array: microarray expression profile, ATP1A2: ATPase Na^+/K^+ transporting subunit alpha 2, BBB: blood-brain barrier, BP: binding partner, EST: expressed sequence tag, GABRG: gamma-aminobutyric acid (GABA) A receptor gamma, MATE: multidrug and toxin extrusion, protein, n.a.: not applicable, OAT: organic anion transporter, OATP: organic anion transporting polypeptide, OCT: organic cation transporter, P-gp: P-glycoprotein, RT-PCR: reverse transcription-polymerase chain reaction measured expression profile. [a] Binding partner representing various neurotransmitter transporters (therapeutic target). [b] Information from PK-Sim® expression database.

S4.2 Types of Interaction

S4.2.1 Competitive Inhibition

$$K_{M,app} = K_M * (1 + \frac{[I]}{K_i}) \tag{S2}$$

$$v = \frac{v_{max} * [S]}{K_{M,app} + [S]} = \frac{k_{cat} * [E] * [S]}{K_{M,app} + [S]} \tag{S3}$$

$K_{M,app}$ = Michaelis-Menten constant in the presence of inhibitor, K_M = Michaelis-Menten constant, $[I]$ = free inhibitor concentration, K_i = dissociation constant of the inhibitor-enzyme/transporter complex, v = reaction velocity, $[S]$ = free substrate concentration, k_{cat} = catalytic or transport rate constant and $[E]$ = enzyme concentration.

S4.2.2 Non-Competitive Inhibition

$$v_{max,app} = \frac{v_{max}}{1 + \frac{[I]}{K_i}} \tag{S4}$$

$$v = \frac{v_{max,app} \cdot [S]}{K_M + [S]} \tag{S5}$$

$v_{max,app}$ = maximum reaction velocity in the presence of inhibitor, v_{max} = maximum reaction velocity, $[I]$ = free inhibitor concentration, K_i = dissociation constant of the inhibitor-enzyme/transporter complex, v = reaction velocity, $[S]$ = free substrate concentration and K_M = Michaelis-Menten constant.

S4.2.3 Mechanism-Based Inactivation

$$\frac{d[E]}{dt} = k_{deg} * E_0 - \frac{k_{deg} + k_{inact} * [I]}{K_I + [I]} * [E] \tag{S6}$$

$\frac{d[E]}{dt}$ = enzyme turnover, k_{deg} = degradation rate constant, E_0 = enzyme concentration at time 0, $[I]$ = free mechanism-based inactivator concentration, k_{inact} = maximum inactivation rate constant, K_I = concentration for half-maximal inactivation and $[E]$ = enzyme concentration.

S4.2.4 Induction

$$\frac{d[E]}{dt} = k_{deg} * E_0 * \frac{1 + (E_{max} * [Ind])}{EC50 + [Ind]} \tag{S7}$$

$\frac{d[E]}{dt}$ = enzyme turnover, k_{deg} = degradation rate constant, E_0 = enzyme concentration at time 0, E_{max} = maximal induction effect *in vivo*, $[Ind]$ = free inducer concentration and $EC50$ = concentration for half maximal induction *in vivo*.

S4.2.5 Down Regulation

$$\frac{d[E]}{dt} = k_{deg} * E_0 * \frac{1 + (E_{max} * [Ind])}{EC50 + [Ind]} \tag{S8}$$

$\frac{d[E]}{dt}$ = enzyme turnover, k_{deg} = degradation rate constant, E_0 = enzyme concentration at time 0, E_{max} = maximal induction effect *in vivo*, $[Ind]$ = free inducer concentration and $EC50$ = concentration for half maximal induction *in vivo*.

S4.3 Published PBPK DDI Models

Interaction parameters added to the original model during the network development process are indicated by citing the corresponding references.

Table S11: Published perpetrator models and included relevant interaction constants

Model (PK-Sim® Version)	Mechanism	Parameter	Value	Publication	Model repository
Atomoxetine (V11)				Rüdesheim et al. 2022 [1]	Atomoxetine-Model
Atomoxetine	Competitive inhibition	CYP3A4 K_i [µmol/L]	34.00	[17]	
	Competitive inhibition	CYP2D6 K_i [µmol/L]	3.60	[17]	
Bupropion (V11)				Marok et al. 2021 [55]	Bupropion-DDGI-Model
Bupropion	Down regulation	CYP2D6 E_{max}	-0.63	[56, 57]	
	Down regulation	CYP2D6 EC_{50}	0.61	[56, 57]	
	Competitive inhibition	CYP2D6 K_i	21	[16]	
Hydroxybupropion	Down regulation	CYP2D6 E_{max}	-0.96	[56, 57]	
	Down regulation	CYP2D6 EC_{50}	1.78	[56, 57]	
	Competitive inhibition	CYP2D6 EC_i	13.30	[16]	
Erythrohydrobupropion	Down regulation	CYP2D6 E_{max}	-0.23	[56, 57]	
	Down regulation	CYP2D6 EC_{50}	2.30	[56, 57]	
	Competitive inhibition	CYP2D6 EC_i	1.70	[16]	
Threohydrobupropion	Down regulation	CYP2D6 E_{max}	-0.53	[56, 57]	
	Down regulation	CYP2D6 EC_{50}	0.10	[56, 57]	
	Competitive inhibition	CYP2D6 EC_i	5.40	[16]	
Carbamazepine (V11)				Fuhr et al. 2021 [58]	Carbamazepine-Model (OSP, v1.0)[a]
Carbamazepine	Induction	CYP3A4 E_{max}	6.00		
	Induction	CYP3A4 EC_{50} [µmol/L]	20.00		
Carbamazepine-10,11-epoxide	-	-	-		
Cimetidine (V11)				Hanke et al. 2020 [59]	Cimetidine-Model (OSP, v1.1)[a]
Cimetidine	Competitive inhibition	CYP3A4 K_i [µmol/L]	268.00		
	Competitive inhibition	CYP2D6 K_i [µmol/L]	38.00	[60]	
Clarithromycin (V11)				Hanke et al. 2018 [50]	Clarithromycin-Model (OSP, v1.2)[a]
Clarithromycin	Mechanism-based inactivation	CYP3A4 KI	6.04		
	Mechanism-based inactivation	CYP3A4 k_{inact} [µmol/L]	0.04		

CYP: cytochrome P450, EC_{50}: concentration for half maximal induction, E_{max}: maximal induction effect, K_i: dissociation constant of the inhibitor-enzyme/transporter (competitive) and inhibitor-enzyme/transporter(-substrate) complex (non-competitive), KI: concentration for 50% inactivation (mechanism-based inactivation), k_{inact}: maximum inactivation rate (mechanism-based inactivation), OSP: Open Systems Pharmacology, P-gp: P-glycoprotein. If not otherwise indicated, interaction constants were adopted from the respective published models. Hyperlinks refer to the respective model repositories. [a] Open Systems Pharmacology model repository (https://github.com/Open-Systems-Pharmacology). [b] modification by Feick et al. [66].

Table S11: Published perpetrator models and included relevant interaction constants *(continued)*

Model (PK-Sim® Version)	Mechanism	Parameter	Value	Publication	Model repository
Erythromycin (V11)					Erythromycin-Model (OSP, v1.3)[a]
Erythromycin	Mechanism-based inactivation	CYP3A4 KI	7.60		
	Mechanism-based inactivation	CYP3A4 k_{inact} [µmol/L]	0.03		
Fluvoxamine (V11)				Britz et al. 2019 [61]	Fluvoxamine-Model (OSP, v1.2)[a]
Fluvoxamine	Competitive inhibition	CYP3A4 K_i [µmol/L]	1.60		
	Competitive inhibition	CYP2D6 K_i [µmol/L]	8.2	[62]	
	Competitive inhibition	CYP2C19 K_i [nmol/L]	3.60	[63]	
Itraconazole (V11)				Hanke et al. 2018 [50]	Itraconazole-Model (OSP, v1.3)[a]
Itraconazole	Competitive inhibition	CYP3A4 K_i [nmol/L]	1.30		
	Competitive inhibition	P-gp K_i [nmol/L]	8.00		
Hydroxy-itraconazole	Competitive inhibition	CYP3A4 K_i [nmol/L]	14.40		
Keto-itraconazole	Competitive inhibition	CYP3A4 K_i [nmol/L]	5.12		
N-Desalkyl-itraconazole	Competitive inhibition	CYP3A4 K_i [nmol/L]	0.32		
Ketoconazole (V11)				Marok et al. 2023 [64]	Ketoconazole-DDI-Model
Ketoconazole	Competitive inhibition	CYP3A4 K_i [µmol/L]	0.008		
	Competitive inhibition	P-gp K_i [µmol/L]	0.035		
N-Deacetylketoconazole	Competitive inhibition	CYP3A4 K_i [µmol/L]	0.022		
	Competitive inhibition	P-gp K_i [µmol/L]	0.119		
N-Deacetyl-N-Hydroxyketoconazole	Competitive inhibition	CYP3A4 K_i [µmol/L]	0.022		
	Competitive inhibition	P-gp K_i [µmol/L]	0.119		
Omeprazole (V11)				Kanacher et al. 2020 [54]	Omeprazole-Model (OSP, v1.1)[a]
R-Omeprazole	Competitive inhibition	CYP3A4 K_i [µmol/L]	44.50[b]		
S-Omeprazole	Competitive inhibition	CYP3A4 K_i [µmol/L]	46.60[b]		
Paroxetine (V11)				Rüdesheim et al. 2022 [1]	Paroxetine-Model
Paroxetine	Mechanism-based inactivation	CYP2D6 KI [µmol/L]	0.17		
	Mechanism-based inactivation	CYP2D6 k_{inact} [1/min]	0.17		
	Mechanism-based inactivation	CYP3A4 KI [µmol/L]	4.48		
	Mechanism-based inactivation	CYP3A4 k_{inact} [1/min]	0.01		

CYP: cytochrome P450, EC_{50}: concentration for half maximal induction, E_{max}: maximal induction effect, K_i: dissociation constant of the inhibitor-enzyme/transporter (competitive) and inhibitor-enzyme/transporter(-substrate) complex (non-competitive), KI: concentration for 50% inactivation (mechanism-based inactivation), k_{inact}: maximum inactivation rate (mechanism-based inactivation), OSP: Open Systems Pharmacology, P-gp: P-glycoprotein. If not otherwise indicated, interaction constants were adopted from the respective published models. Hyperlinks refer to the respective model repositories. [a] Open Systems Pharmacology model repository (https://github.com/Open-Systems-Pharmacology). [b] modification by Feick et al. [66].

Table S11: Published perpetrator models and included relevant interaction constants *(continued)*

**Model (PK-Sim® Version)**	**Mechanism**	**Parameter**	**Value**	**Publication**	**Model repository**
Quinidine (V11)				Feick et al. 2023 [66]	Quinidine-Model
Quinidine	Competitive inhibition	CYP2D6 K_i [µmol/L]	0.017		
	Competitive inhibition	P-gp K_i [µmol/L]	0.10		
3-Hydroxyquinidine	Competitive inhibition	CYP2D6 K_i [µmol/L]	2.30		
Rifampicin (V11)				Hanke et al. 2018 [50]	Rifampicin-Model (OSP, v1.2)[a]
Rifampicin	Induction	CYP3A4 E_{max}	9.00		
	Induction	CYP3A4 EC_{50} [µmol/L]	0.34		
	Competitive inhibition	CYP3A4 K_i [µmol/L]	18.50		
	Induction	P-gp E_{max}	2.50		
	Induction	P-gp EC_{50} [µmol/L]	0.34		
	Competitive inhibition	P-gp K_i [µmol/L]	169.00		
Verapamil (V11)				Hanke et al. 2020a [67]	Verapamil-Norverapamil-Model
R-Verapamil	Mechanism-based inactivation	CYP3A4 KI [µmol/L]	27.63		
	Mechanism-based inactivation	CYP3A4 k_{inact} [1/min]	0.038		
	Non-competitive inhibition	P-gp K_i [µmol/L]	0.038		
S-Verapamil	Mechanism-based inactivation	CYP3A4 KI [µmol/L]	3.85		
	Mechanism-based inactivation	CYP3A4 k_{inact} [1/min]	0.034		
	Non-competitive inhibition	P-gp K_i [µmol/L]	0.038		
R-Norverapamil	Mechanism-based inactivation	CYP3A4 KI [µmol/L]	6.10		
	Mechanism-based inactivation	CYP3A4 k_{inact} [1/min]	0.048		
	Non-competitive inhibition	P-gp K_i [µmol/L]	0.038		
S-Norverapamil	Mechanism-based inactivation	CYP3A4 KI [µmol/L]	2.90		
	Mechanism-based inactivation	CYP3A4 k_{inact} [1/min]	0.080		
	Non-competitive inhibition	P-gp K_i [µmol/L]	0.038		

CYP: cytochrome P450, EC_{50}: concentration for half maximal induction, E_{max}: maximal induction effect, K_i: dissociation constant of the inhibitor-enzyme/transporter (competitive) and inhibitor-enzyme/transporter(-substrate) complex (non-competitive), KI: concentration for 50% inactivation (mechanism-based inactivation), k_{inact}: maximum inactivation rate (mechanism-based inactivation), OSP: Open Systems Pharmacology, P-gp: P-glycoprotein. If not otherwise indicated, interaction constants were adopted from the respective published models. Hyperlinks refer to the respective model repositories. [a] Open Systems Pharmacology model repository (https://github.com/Open-Systems-Pharmacology). [b] modification by Feick et al. [66].

Table S12: Published victim models and affected metabolism and transport pathways

Model (PK-Sim® Version)	Mechanism	Parameter	Value	Publication	Model repository
Alprazolam (V11)					Alprazolam-Model (OSP, v1.1)[a]
Alprazolam	Metabolism to 4-OHA	CYP3A4 K_M [µmol/L]	704.00		
	Metabolism to 4-OHA	CYP3A4 k_{cat} [1/min]	13.73		
	Metabolism to α-OHA	CYP3A4 K_M [µmol/L]	269.00		
	Metabolism to α-OHA	CYP3A4 k_{cat} [1/min]	0.81		
Atomoxetine (V11)				Rüdesheim et al. 2022 [1]	Atomoxetine-Model
Atomoxetine	Metabolism	CYP2D6 K_M [µmol/L]	2.30		
	Metabolism	CYP2D6 k_{cat} [1/min] (NM)	37.44		
(E)-Clomiphene (V11)				Kovar et al. 2022 [68]	E-clomiphene-Model
(E)-clomiphene	Metabolism to OHC	CYP2D6 K_M [µmol/L]	0.13		
	Metabolism to OHC	CYP2D6 k_{cat} [1/min] (NM)	306.38		
	Metabolism to NDC	CYP2D6 K_M [µmol/L]	0.78		
	Metabolism to NDC	CYP2D6 k_{cat} [1/min] (NM)	121.36		
	Metabolism	CYP2D6 K_M [µmol/L]	0.03		
	Metabolism	CYP2D6 k_{cat} [1/min] (NM)	130.35		
	Metabolism to NDC	CYP3A4 K_M [µmol/L]	0.78		
	Metabolism to NDC	CYP3A4 k_{cat} [1/min]	44.95		
(*E*)-4-hydroxyclomiphene	Metabolism	CYP2D6 K_M [µmol/L]	3.60		
	Metabolism	CYP2D6 k_{cat} [1/min] (NM)	8.55.17		
	Metabolism to HDC	CYP3A4 K_M [µmol/L]	3.40		
	Metabolism to HDC	CYP3A4 k_{cat} [1/min]	19.52		
(*E*)-N-desethylclomiphene	Metabolism to HDC	CYP2D6 K_M [µmol/L]	0.49		
	Metabolism to HDC	CYP2D6 k_{cat} [1/min] (NM)	64.52		
	Metabolism	CYP2D6 K_M [µmol/L]	0.97		
	Metabolism	CYP2D6 k_{cat} [1/min] (NM)	5.84		
	Metabolism	CYP3A4 K_M [µmol/L]	0.97		
	Metabolism	CYP3A4 k_{cat} [1/min]	0.78		
(*E*)-4-OH-DE-clomiphene	Metabolism	CYP2D6 K_M [µmol/L]	8.86		
	Metabolism	CYP2D6 k_{cat} [1/min] (NM)	211.71		

α-OHA: α-hydroxyalprazolam, CL: clearance, CYP: cytochrome P450, DXT: dextrorphan, HDC/(*E*)-4-OH-DE-clomiphene:(*E*)-4-hydroxy-N-desethylclomiphene, k_{cat}: catalytic or transport rate constant, K_M: Michaelis-Menten constant, NDC: (*E*)-N-desethylclomiphene, NM: CYP2D6 normal metabolizer, 4-OHA: 4-hydroxyalprazolam, OHC: (*E*)-4-hydroxyclomiphene, OHM: α-hydroxymetoprolol, OHQ: 3-hydroxyquinidine, OHR: 9-hydroxyrisperidone, OSP: Open Systems Pharmacology, P-gp: P-glycoprotein. If not otherwise indicated, process parameters were adopted from the respective published models. CYP2D6 NM kcat values were adjusted according to the respective publications to account for varying CYP2D6 activity levels. Hyperlinks refer to the respective model repositories. [a] Open Systems Pharmacology model repository (https://github.com/Open-Systems-Pharmacology). [b] Replacement of original unspecific hepatic metabolic clearance process.

Table S12: Published victim models and affected metabolism and transport pathways *(continued)*

Model (PK-Sim® Version)	Mechanism	Parameter	Value	Publication	Model repository
Dextromethorphan (V11)				Rüdesheim et al. 2022 [69]	Dextromethorphan-Model
Dextromethorphan	Metabolism to DXT	CYP2D6 K_M [µmol/L]	4.65		
	Metabolism to DXT	CYP2D6 k_{kat} [1/min] (NM)	90.89		
	Metabolism	CYP4A4 K_M [µmol/L]	176.80		
	Metabolism	CYP3A4 k_{kat} [1/min]	5.65		
Dextrorphan	Metabolism	CYP4A4 K_M [µmol/L]	910.00		
	Metabolism	CYP3A4 k_{kat} [1/min]	7.41		
Dextrorphan-O-glucuronide	-	-	-		
Digoxin (V11)				Hanke et al. 2018 [50]	Digoxin-Model (OSP)[a]
Digoxin	Transport	P-gp K_M [µmol/L]	177.00		
	Transport	P-gp k_{cat} [1/min]	71.16		
Metoprolol (V11)				Rüdesheim et al. 2020 [70]	Metoprolol-Model
R-Metoprolol	Metabolism to OHM	CYP2D6 K_M [µmol/L]	10.08		
	Metabolism to OHM	CYP2D6 k_{cat} [1/min] (NM)	6.02		
	Metabolism	CYP2D6 K_M [µmol/L]	8.82		
	Metabolism	CYP2D6 k_{cat} [1/min] (NM)	9.87		
	Metabolism	CYP3A4 CL [1/min]	0.02[b]		
S-Metoprolol	Metabolism to OHM	CYP2D6 K_M [µmol/L]	10.75		
	Metabolism to OHM	CYP2D6 k_{cat} [1/min] (NM)	8.27		
	Metabolism	CYP2D6 K_M [µmol/L]	12.43		
	Metabolism	CYP2D6 k_{cat} [1/min] (NM)	10.37		
	Metabolism	CYP3A4 CL [1/min]	0.02[b]		
α-Hydroxymetoprolol	-	-	-		
Mexiletine (V11)				Kanacher et al. 2020 [54]	Mexiletine-Model (OSP, v1.1)[a]
Mexiletine	Metabolism	CYP2D6 CL [1/min] (NM)	0.46		
Midazolam (V11)				Hanke et al. 2018 [50]	Midazolam-Model (OSP, v1.1)[a]
Midazolam	Metabolism	CYP3A4 K_M [µmol/L]	4.00		
	Metabolism	CYP3A4 k_{cat} [1/min]	8.76		

α-OHA: α-hydroxyalprazolam, CL: clearance, CYP: cytochrome P450, DXT: dextrorphan, HDC/(*E*)-4-OH-DE-clomiphene:(*E*)-4-hydroxy-N-desethylclomiphene, k_{cat}: catalytic or transport rate constant, K_M: Michaelis-Menten constant, NDC: (*E*)-N-desethylclomiphene, NM: CYP2D6 normal metabolizer, 4-OHA: 4-hydroxyalprazolam, OHC: (*E*)-4-hydroxyclomiphene, OHM: α-hydroxymetoprolol, OHQ: 3-hydroxyquinidine, OHR: 9-hydroxyrisperidone, OSP: Open Systems Pharmacology, P-gp: P-glycoprotein. If not otherwise indicated, process parameters were adopted from the respective published models. CYP2D6 NM kcat values were adjusted according to the respective publications to account for varying CYP2D6 activity levels. Hyperlinks refer to the respective model repositories. [a] Open Systems Pharmacology model repository (https://github.com/Open-Systems-Pharmacology). [b] Replacement of original unspecific hepatic metabolic clearance process.

Table S12: Published victim models and affected metabolism and transport pathways *(continued)*

Model (PK-Sim® Version)	Mechanism	Parameter	Value	Publication	Model repository
Paroxetine (V11)				Rüdesheim et al. 2022 [1]	Paroxetine-Model
Paroxetine	Metabolism	CYP2D6 K_M [µmol/L]	0.03		
	Metabolism	CYP2D6 k_{cat} [1/min] (NM)	1.37		
	Metabolism	CYP3A4 K_M [µmol/L]	4.70		
	Metabolism	CYP3A4 k_{cat} [1/min]	1.01		
Quinidine (V11)				Feick et al. 2023 [66]	Quinidine-Model
Quinidine	Metabolism to OHQ	CYP3A4 K_M [µmol/L]	51.80		
	Metabolism to OHQ	CYP3A4 k_{cat} [1/min]	2.21		
	Metabolism	CYP3A4 K_M [µmol/L]	65.03		
	Metabolism	CYP3A4 k_{cat} [1/min]	3.84		
	Transport	P-gp K_M [µmol/L]	0.23		
	Transport	P-gp k_{cat} [1/min]	0.77		
3-Hydroxyquinidine	Metabolism	CYP3A4 CL [1/min]	0.08		
Risperidone (V11)				Rüdesheim et al. 2022 [1]	Risperidone-Model
Risperidone	Metabolism to OHR	CYP2D6 K_M [µmol/L]	1.10		
	Metabolism to OHR	CYP2D6 k_{cat} [1/min] (NM)	1.04		
	Metabolism	CYP2D6 K_M [µmol/L]	1.10		
	Metabolism	CYP2D6 k_{cat} [1/min] (NM)	0.63		
	Metabolism to OHR	CYP3A4 K_M [µmol/L]	61.00		
	Metabolism to OHR	CYP3A4 k_{cat} [1/min]	0.70		
	Metabolism	CYP3A4 K_M [µmol/L]	61.00		
	Metabolism	CYP3A4 k_{cat} [1/min]	0.15		
	Transport	P-gp K_M [µmol/L]	26.30		
	Transport	P-gp k_{cat} [1/min]	12.72		
9-Hydroxyrisperidone	Transport	P-gp K_M [µmol/L]	149.60		
	Transport	P-gp k_{cat} [1/min]	$5.70 \cdot 10^{-3}$		

α-OHA: α-hydroxyalprazolam, CL: clearance, CYP: cytochrome P450, DXT: dextrorphan, HDC/(*E*)-4-OH-DE-clomiphene:(*E*)-4-hydroxy-N-desethylclomiphene, k_{cat}: catalytic or transport rate constant, K_M: Michaelis-Menten constant, NDC: (*E*)-N-desethylclomiphene, NM: CYP2D6 normal metabolizer, 4-OHA: 4-hydroxyalprazolam, OHC: (*E*)-4-hydroxyclomiphene, OHM: α-hydroxymetoprolol, OHQ: 3-hydroxyquinidine, OHR: 9-hydroxyrisperidone, OSP: Open Systems Pharmacology, P-gp: P-glycoprotein. If not otherwise indicated, process parameters were adopted from the respective published models. CYP2D6 NM kcat values were adjusted according to the respective publications to account for varying CYP2D6 activity levels. Hyperlinks refer to the respective model repositories. [a] Open Systems Pharmacology model repository (https://github.com/Open-Systems-Pharmacology). [b] Replacement of original unspecific hepatic metabolic clearance process.

S5 DGI Model Evaluation

S5.1 DGI AUC_{last} and C_{max} Ratios

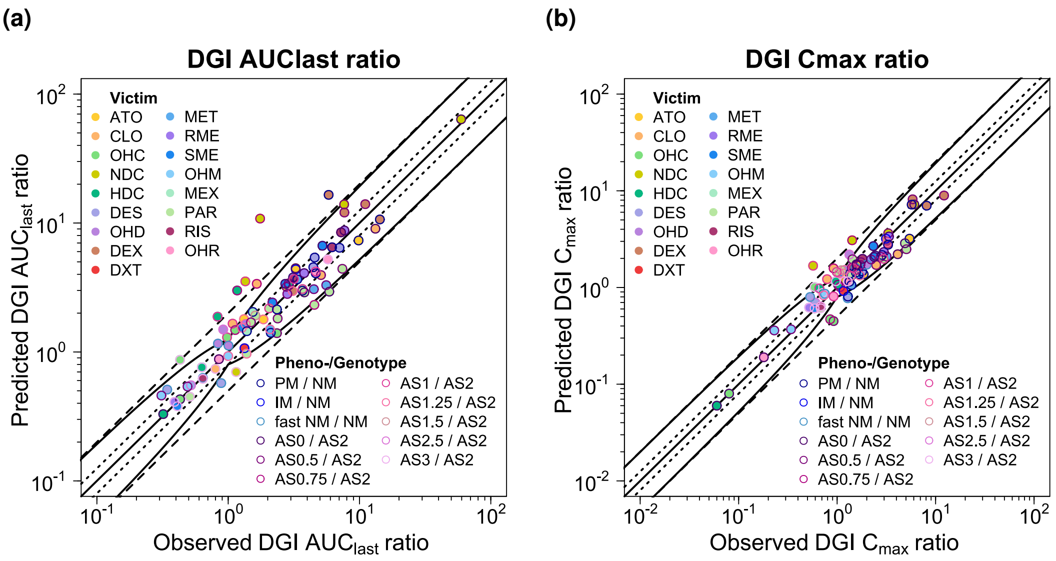

Figure S13: Goodness-of-fit plots comparing predicted and observed DGI AUC_{last} and C_{max} ratios. The solid line marks the line of identity. Dotted lines indicate 1.25-fold, dashed lines indicate 2-fold deviation. Prediction success limits proposed by Guest et al. [2] are shown as curved lines (including 20% variability). AS: CYP2D6 activity score, ATO: atomoxetine, AUC_{last}: area under the plasma concentration-time curve calculated between the first and last concentration measurement, CLO: (*E*)-clomiphene, C_{max}: maximum plasma concentration, DES: desipramine, DEX: dextromethorphan, DGI: drug-gene interaction, DXT: dextrorphan, HDC: (*E*)-4-hydroxy-N-desethylclomiphene, IM: CYP2D6 intermediate metabolizer, MET: metoprolol, MEX: mexiletine, NDC: (*E*)-N-desethylclomiphene, NM: CYP2D6 normal metabolizer, OHC: (*E*)-4-hydroxyclomiphene, OHD: 2-hydroxydesipramine, OHM: α-hydroxymetoprolol, OHR: 9-hydroxyrisperidone, PAR: paroxetine, PM: CYP2D6 poor metabolizer, RIS: risperidone, RME: R-metoprolol, SME: S-metoprolol.

S5.2 Geometric Mean Fold Errors of Predicted DGI AUC_{last} and C_{max} Ratios

Table S13: Predicted and observed DGI AUC_{last} and C_{max} ratios

		DGI AUC_{last} ratio			DGI C_{max} ratio					
**Drug administration**	**t_{last} [h]**	**Pred**	**Obs**	**Pred/Obs**	**Pred**	**Obs**	**Pred/Obs**	**Phenotype/AS**	**Molecule**	**Reference**
Atomoxetine										
20 mg s.d. po	24 / 24	4.47	3.26	1.37	1.71	1.57	0.86	AS0 / AS2	ATO	Jung 2020 [71]
20 mg s.d. po	24 / 24	1.79	1.86	0.96	1.17	1.21	1.09	AS1.25 / AS2	ATO	Jung 2020 [71]
20 mg s.d. po	24 / 24	3.20	2.78	1.15	1.71	1.61	1.06	AS0.5 / AS2	ATO	Kim 2018 [72]
20 mg b.i.d. po	216 / 72	7.33	9.84	0.74	3.20	5.43	0.59	PM / NM	ATO	Sauer 2003 [73]
25 mg s.d. po	48 / 48	2.99	3.68	0.81	1.37	1.86	0.74	PM / NM	ATO	Todor 2016 [74]
40 mg s.d. po	24 / 24	3.41	2.89	1.18	1.83	1.74	1.05	AS0.5 / AS2	ATO	Byeon 2015 [75]
40 mg s.d. po	24 / 24	1.80	1.32	1.36	1.40	1.15	1.22	AS1.25 / AS2	ATO	Byeon 2015 [75]
**Mean GMFE (range):**		**1.24 (1.04–1.37), 7/7 with GMFE $\leq$ 2**			**1.22 (1.03–1.70), 7/7 with GMFE $\leq$ 2**					
(E)-Clomiphene										
42 mg s.d. po	168 / 72	9.04	13.21	0.68	2.21	4.12	0.54	AS0 / AS2	CLO	Mürdter 2016 [76]
42 mg s.d. po	168 / 168	0.43	0.43	1.01	0.08	0.08	0.99	AS0 / AS2	OHC	Mürdter 2016 [76]
42 mg s.d. po	168 / 72	63.77	59.41	1.07	7.23	6.07	1.19	AS0 / AS2	NDC	Mürdter 2016 [76]
42 mg s.d. po	168 / 168	0.33	0.32	1.03	0.06	0.06	1.04	AS0 / AS2	HDC	Mürdter 2016 [76]
42 mg s.d. po	168 / 72	3.95	5.09	0.78	1.71	2.49	0.69	AS0.5 / AS2	CLO	Mürdter 2016 [76]
42 mg s.d. po	168 / 168	1.40	2.35	0.59	0.45	0.92	0.48	AS0.5 / AS2	OHC	Mürdter 2016 [76]
42 mg s.d. po	168 / 72	13.95	7.64	1.83	3.64	3.30	1.10	AS0.5 / AS2	NDC	Mürdter 2016 [76]
42 mg s.d. po	168 / 168	3.29	2.90	1.13	1.07	1.02	1.05	AS0.5 / AS2	HDC	Mürdter 2016 [76]
42 mg s.d. po	168 / 72	3.39	1.65	2.05	1.58	0.92	1.72	AS0.75 / AS2	CLO	Mürdter 2016 [76]
42 mg s.d. po	168 / 168	1.48	1.13	1.31	0.47	0.85	0.55	AS0.75 / AS2	OHC	Mürdter 2016 [76]
42 mg s.d. po	168 / 72	10.83	1.75	6.19	3.08	1.42	2.17	AS0.75 / AS2	NDC	Mürdter 2016 [76]
42 mg s.d. po	168 / 168	3.00	1.17	2.57	1.01	1.07	0.94	AS0.75 / AS2	HDC	Mürdter 2016 [76]
42 mg s.d. po	72 / 72	1.66	1.08	1.54	1.22	0.79	1.55	AS1 / AS2	CLO	Mürdter 2016 [76]
42 mg s.d. po	168 / 168	1.30	0.98	1.32	0.61	0.59	1.03	AS1 / AS2	OHC	Mürdter 2016 [76]
42 mg s.d. po	72 / 168	3.53	1.35	2.61	1.68	0.57	2.92	AS1 / AS2	NDC	Mürdter 2016 [76]
42 mg s.d. po	168 / 168	1.88	0.83	2.25	0.98	0.66	1.49	AS1 / AS2	HDC	Mürdter 2016 [76]
42 mg s.d. po	72 / 72	0.74	0.80	0.93	0.88	0.71	1.23	AS3 / AS2	CLO	Mürdter 2016 [76]
42 mg s.d. po	168 / 168	0.87	0.43	2.00	1.01	0.59	1.72	AS3 / AS2	OHC	Mürdter 2016 [76]
42 mg s.d. po	72 / 72	0.70	1.15	0.61	1.17	1.19	0.98	AS3 / As2	NDC	Mürdter 2016 [76]
42 mg s.d. po	168 / 168	0.76	0.63	1.20	1.16	0.97	1.19	AS3 / AS2	HDC	Mürdter 2016 [76]
**Mean GMFE (range):**		**1.83 (1.03–6.19), 14/20 with GMFE $\leq$ 2**			**1.46 (1.02–2.95), 17/20 with GMFE $\leq$ 2**					
Desipramine										
50 mg s.d. iv	432 / 72	4.55	4.45	1.02	0.81	1.29	0.63	PM / NM	DES	Brøsen 1988 [4]
50 mg s.d. iv	72 / 72	0.57	0.88	0.65	0.77	1.28	0.60	fast NM / NM	DES	Brøsen 1988 [4]
25 mg s.d. po	96 / 48	4.08	4.64	0.88	1.47	1.80	0.82	PM / NM	DES	Spina 1987 [5]
100 mg s.d. po	240 / 72	5.40	4.56	1.19	1.37	1.66	0.83	PM / NM	DES	Brøsen 1993 [22]
100 mg s.d. po	48 / 72	0.51	0.34	1.49	0.80	0.53	1.50	fast NM / NM	DES	Brøsen 1993 [22]
100 mg s.d. po	72 / 72	1.16	0.84	1.39	1.61	1.54	1.05	fast NM / NM	OHD	Brøsen 1993 [22]

AS: CYP2D6 activity score, ATO: atomoxetine, AUC_{last}: area under the plasma concentration-time curve calculated between the first and last concentration measurement, b.i.d.: twice daily, CLO: (*E*)-clomiphene, C_{max}: maximum plasma concentration, DES: desipramine, DEX: dextromethorphan, DGI: drug-gene interaction, DXT: dextrorphan, GMFE: geometric mean fold error, HDC: (*E*)-4-hydroxy-N-desethylclomiphene, IM: CYP2D6 intermediate metabolizer, iv: intravenous, MET: metoprolol, MEX: mexiletine, NDC: (*E*)-N-desethylclomiphene, NM: CYP2D6 normal metabolizer, obs: observed, OHC: (*E*)-4-hydroxyclomiphene, OHD: 2-hydroxydesipramine, OHM: α-hydroxymetoprolol, OHR: 9-hydroxyrisperidone, PAR: paroxetine, PM: CYP2D6 poor metabolizer, RIS: risperidone, RME: R-metoprolol, SME: S-metoprolol, po: oral, pred: predicted, q.d.: once daily, s.d.: single dose, t_{last}: time of the last concentration measurement. If perpetrator or victim drugs were applied in form of salts, the respective dose of base was calculated and incorporated in simulations.

Table S13: Predicted and observed DGI AUC_{last} and C_{max} ratios *(continued)*

		DGI AUC_{last} ratio			DGI C_{max} ratio					
**Drug administration**	**t_{last} [h]**	**Pred**	**Obs**	**Pred/Obs**	**Pred**	**Obs**	**Pred/Obs**	**Phenotype/AS**	**Molecule**	**Reference**
100 mg s.d. po	14 / 14	0.55	0.52	1.06	0.70	0.60	1.16	AS2.5 / AS2	DES	Bergmann 2001 [20]
100 mg s.d. po	24 / 24	1.49	0.92	1.63	2.19	1.33	1.65	AS2.5 / AS2	OHD	Bergmann 2001 [20]
100 mg s.d. po	408 / 72	6.43	7.04	0.91	1.39	1.35	1.04	PM / NM	DES	Brøsen 1986 [21]
100 mg s.d. po	72 / 72	0.63	0.64	0.98	0.86	0.75	1.15	fast NM / NM	DES	Brøsen 1986 [21]
100 mg s.d. po	48 / 72	1.12	1.01	1.11	1.72	1.99	0.86	fast NM / NM	OHD	Brøsen 1986 [21]
**Mean GMFE (range):**		**1.24 (1.02–1.63), 11/11 with GMFE $\leq$ 2**			**1.31 (1.04–1.65), 11/11 with GMFE $\leq$ 2**					
Dextromethorphan										
5 mg s.d. po	24 / 24	4.37	3.27	1.34	3.20	3.13	1.34	IM / NM	DEX	Storelli 2018 [77]
5 mg s.d. po	24 / 24	1.07	1.33	0.80	0.92	1.16	0.80	IM / NM	DXT	Storelli 2018 [77]
15 mg s.d. po	24 / 12	14.01	11.06	1.27	8.94	12.17	0.73	AS0.5 / AS2	DEX	Qiu 2016 [78]
15 mg s.d. po	24 / 12	2.97	3.10	0.96	2.94	3.23	0.91	AS1.25 / AS2	DEX	Qiu 2016 [78]
30 mg s.d. po	24 / 24	12.07	7.67	1.57	8.17	5.84	1.40	AS0.5 / AS2	DEX	Yamazaki 2017 [79]
30 mg s.d. po	168 / 48	16.55	5.82	2.84	7.20	5.71	1.26	PM / NM	DEX	Capon 1996 [80]
30 mg s.d. po	12 / 12	10.67	14.29	0.75	7.02	8.11	0.87	PM / NM	DEX	Gorski 2004 [81]
**Mean GMFE (range):**		**1.52 (1.04–2.84), 6/7 with GMFE $\leq$ 2**			**1.22 (1.02–1.40), 7/7 with GMFE $\leq$ 2**					
Metoprolol										
20 mg s.d. iv	8 / 8	1.41	2.11	0.67	1.06	1.40	0.76	PM / NM	MET	Leemann 1993 [82]
100 mg s.d. po	24 / 12	0.46	0.31	1.46	0.36	0.23	1.56	AS0.5 / AS2	MET	Bae 2014 [83]
100 mg s.d. po	24 / 24	3.302	5.58	1.69	2.08	2.77	0.75	AS0.5 / AS2	OHM	Bae 2014 [83]
100 mg s.d. po	24 / 12	3.07	4.49	0.68	2.09	3.18	0.66	AS0.5 / AS2	MET	Jin 2008 [84]
100 mg s.d. po	24 / 24	0.54	0.49	1.09	0.37	0.34	1.06	AS0.5 / AS2	OHM	Jin 2008 [84]
100 mg s.d. po	24 / 12	1.50	2.08	0.72	1.33	1.70	0.78	AS1.25 / AS2	MET	Jin 2008 [84]
100 mg s.d. po	24 / 24	0.93	1.00	0.92	0.83	0.73	1.14	AS1.25 / AS2	OHM	Jin 2008 [84]
100 mg s.d. po	48 / 24	3.66	3.36	1.09	2.17	2.86	0.76	PM / NM	MET	Hamelin 2000 [85]
100 mg s.d. po	48 / 12	3.79	3.12	1.22	2.14	2.44	0.88	AS0 / AS2	MET	Sharma 2005 [86]
100 mg s.d. po	48 / 12	4.09	3.79	1.08	2.33	2.98	0.78	AS0 / AS2	RME	Sharma 2005 [86]
100 mg s.d. po	48 / 12	3.38	2.72	1.25	2.07	2.38	0.87	AS0 / AS2	SME	Sharma 2005 [86]
100 mg s.d. po	12 / 12	2.82	2.82	1.00	1.95	2.21	0.89	AS0.5 / AS2	RME	Huang 1999 [87]
100 mg s.d. po	12 / 12	2.42	2.17	1.11	1.71	1.72	0.99	AS0.5 / AS2	SME	Huang 1999 [87]
100 mg s.d. po	12 / 12	1.65	1.36	1.22	1.52	1.19	1.28	AS1.25 / AS2	RME	Huang 1999 [87]
100 mg s.d. po	12 / 12	1.54	1.27	1.21	1.41	1.05	1.34	AS1.25 / AS2	SME	Huang 1999 [87]
100 mg s.d. po	24 / 24	8.76	7.67	1.14	3.37	3.36	1.00	AS0 / AS2	RME	Seeringer 2008 [88]
100 mg s.d. po	24 / 24	6.67	5.22	1.28	2.68	2.33	1.15	AS0 / AS2	SME	Seeringer 2008 [88]
100 mg s.d. po	10 / 24	0.41	0.39	1.04	0.62	0.52	1.18	AS3 / AS2	RME	Seeringer 2008 [88]
100 mg s.d. po	10 / 24	0.38	0.41	0.93	0.61	0.59	1.02	AS3 / AS2	SME	Seeringer 2008 [88]
**Mean GMFE (range):**		**1.23 (1.00–1.69), 19/19 with GMFE $\leq$ 2**			**1.22 (1.00–1.57), 19/19 with GMFE $\leq$ 2**					

AS: CYP2D6 activity score, ATO: atomoxetine, AUC_{last}: area under the plasma concentration-time curve calculated between the first and last concentration measurement, b.i.d.: twice daily, CLO: (*E*)-clomiphene, C_{max}: maximum plasma concentration, DES: desipramine, DEX: dextromethorphan, DGI: drug-gene interaction, DXT: dextrorphan, GMFE: geometric mean fold error, HDC: (*E*)-4-hydroxy-N-desethylclomiphene, IM: CYP2D6 intermediate metabolizer, iv: intravenous, MET: metoprolol, MEX: mexiletine, NDC: (*E*)-N-desethylclomiphene, NM: CYP2D6 normal metabolizer, obs: observed, OHC: (*E*)-4-hydroxyclomiphene, OHD: 2-hydroxydesipramine, OHM: α-hydroxymetoprolol, OHR: 9-hydroxyrisperidone, PAR: paroxetine, PM: CYP2D6 poor metabolizer, RIS: risperidone, RME: R-metoprolol, SME: S-metoprolol, po: oral, pred: predicted, q.d.: once daily, s.d.: single dose, t_{last}: time of the last concentration measurement. If perpetrator or victim drugs were applied in form of salts, the respective dose of base was calculated and incorporated in simulations.

Table S13: Predicted and observed DGI AUC_{last} and C_{max} ratios *(continued)*

Drug administration	t_{last} [h]	DGI AUC_{last} ratio			DGI C_{max} ratio			Phenotype/AS	Molecule	Reference
		**Pred**	**Obs**	**Pred/Obs**	**Pred**	**Obs**	**Pred/Obs**			
Mexiletine										
100 mg b.i.d. po	12 / 12	2.05	1.54	1.32	1.33	1.40	0.96	PM / NM	MEX	Labbé 2000 [89]
200 mg s.d. po	48 / 48	1.45	1.39	1.05	1.15	1.33	0.86	PM / NM	MEX	Abolfathi 1993 [90]
**Mean GMFE (range):**		**1.19 (1.05–1.33), 2/2 with GMFE $\leq$ 2**			**1.11 (1.05–1.16), 2/2 with GMFE $\leq$ 2**					
Paroxetine										
25 mg s.d. po	96 / 48	4.42	7.40	0.60	2.49	5.05	0.49	AS0.5 / AS2	PAR	Chen 2015 [91]
25 mg s.d. po	72 / 48	3.08	3.68	0.84	1.98	2.96	0.67	AS1 / AS2	PAR	Chen 2015 [91]
25 mg s.d. po	72 / 48	1.92	1.61	1.19	1.51	1.10	1.37	AS1.5 / AS2	PAR	Chen 2015 [91]
30 mg q.d. po	96 / 96	2.14	2.35	0.91	1.431	1.55	0.92	PM / NM	PAR	Sindrup 1992 [92]
40 mg s.d. po	144 / 144	2.90	3.80	0.76	1.74	1.57	1.11	AS0 / AS2	PAR	Mürdter 2016 [76]
40 mg s.d. po	144 / 144	1.82	2.38	0.76	1.60	1.41	1.13	AS0.5 / AS2	PAR	Mürdter 2016 [76]
40 mg s.d. po	144 / 144	2.32	4.53	0.51	1.52	1.57	0.97	AS0.75 / AS2	PAR	Mürdter 2016 [76]
40 mg s.d. po	144 / 144	2.18	2.05	1.06	1.45	0.99	1.46	AS1 / AS2	PAR	Mürdter 2016 [76]
40 mg s.d. po	144 / 144	0.45	0.51	0.88	0.61	0.68	0.90	AS3 / AS2	PAR	Mürdter 2016 [76]
40 mg s.d. po	240 / 48	2.94	5.89	0.50	2.87	4.88	0.59	AS0 / AS2	PAR	Yoon 2000 [93]
40 mg s.d. po	192 / 48	1.70	1.49	1.14	1.96	1.43	1.37	AS0.5 / AS2	PAR	Yoon 2000 [93]
40 mg s.d. po	96 / 24	0.97	1.38	0.70	1.32	1.38	0.96	AS1.25 / AS2	PAR	Yoon 2000 [93]
**Mean GMFE (range):**		**1.33 (1.03–2.00), 12/12 with GMFE $\leq$ 2**			**1.35 (1.05–2.03), 11/12 with GMFE $\leq$ 2**					
Risperidone										
1 mg q.d. po	48 / 24	8.47	7.24	1.17	1.98	1.81	1.09	AS0 / AS2	RIS	Novalbos 2010 [94]
1 mg q.d. po	48 / 48	0.88	0.85	1.04	0.19	0.18	1.06	AS0 / AS2	OHR	Novalbos 2010 [94]
1 mg q.d. po	24 / 24	3.68	3.16	1.16	1.59	1.44	1.10	AS1 / AS2	RIS	Novalbos 2010 [94]
1 mg q.d. po	48 / 48	4.21	4.64	0.91	0.81	0.92	0.88	AS1 / AS2	OHR	Novalbos 2010 [94]
1 mg q.d. po	12 / 24	0.62	0.64	0.97	0.64	0.68	0.94	AS3 / AS2	RIS	Novalbos 2010 [94]
1 mg q.d. po	48 / 48	5.23	5.74	0.91	1.16	1.28	0.91	AS3 / AS2	OHR	Novalbos 2010 [94]
2 mg q.d. po	24 / 24	6.49	6.18	1.05	2.77	3.29	0.84	PM / NM	RIS	Bondolfi 2002 [95]
**Mean GMFE (range):**		**1.09 (1.03–1.17), 7/7 with GMFE $\leq$ 2**			**1.11 (1.06–1.19), 7/7 with GMFE $\leq$ 2**					
**Overall GMFE (range):**		**1.40 (1.00–6.19), 78/85 with GMFE $\leq$ 2**			**1.29 (1.00–2.95), 81/85 with GMFE $\leq$ 2**					

AS: CYP2D6 activity score, ATO: atomoxetine, AUC_{last}: area under the plasma concentration-time curve calculated between the first and last concentration measurement, b.i.d.: twice daily, CLO: (*E*)-clomiphene, C_{max}: maximum plasma concentration, DES: desipramine, DEX: dextromethorphan, DGI: drug-gene interaction, DXT: dextrorphan, GMFE: geometric mean fold error, HDC: (*E*)-4-hydroxy-N-desethylclomiphene, IM: CYP2D6 intermediate metabolizer, iv: intravenous, MET: metoprolol, MEX: mexiletine, NDC: (*E*)-N-desethylclomiphene, NM: CYP2D6 normal metabolizer, obs: observed, OHC: (*E*)-4-hydroxyclomiphene, OHD: 2-hydroxydesipramine, OHM: α-hydroxymetoprolol, OHR: 9-hydroxyrisperidone, PAR: paroxetine, PM: CYP2D6 poor metabolizer, RIS: risperidone, RME: R-metoprolol, SME: S-metoprolol, po: oral, pred: predicted, q.d.: once daily, s.d.: single dose, t_{last}: time of the last concentration measurement. If perpetrator or victim drugs were applied in form of salts, the respective dose of base was calculated and incorporated in simulations.

S6 DDI Model Evaluation

S6.1 Alprazolam

S6.1.1 Clinical Studies

Table S14: Clinical study data used for DDI model development with **alprazolam** as victim

Drug administration									
**Perpetrator**	**Alprazolam**	**n**	**Population[a]**	**Fem. [%]**	**Age [years]**	**Weight [kg]**	**BMI [kg/m²]**	**Molecule**	**Reference**
Paroxetine									
20 mg q.d. po	1 mg q.d. po	22	European [3]	64	26 ± 4	64 ± 9	168 ± 7[b]	ALP, PAR	Calvo 2004 [96]

ALP: alprazolam, BMI: body mass index, DDI: drug-drug interaction, fem: females, n: number of study participants, PAR: paroxetine, po: oral, q.d.: once daily. Values are given as mean (range). If perpetrator or victim drugs were applied in form of salts, the respective dose of base was calculated and incorporated in simulations. [a] Population used in simulations. [b] Height of subjects [cm].

S6.1.2 Plasma Concentration-Time Profiles (Semilogarithmic Representation)

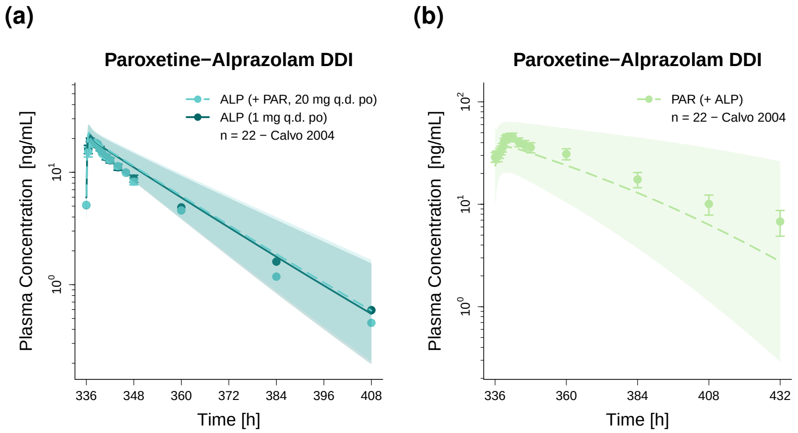

Figure S14: Predicted compared to observed plasma concentration-time profiles of alprazolam alone (solid line) and after pretreatment and/or concomitant administration (dashed line) of (a) paroxetine (semilogarithmic representation). Population predicted (1000 individuals) geometric means are shown as lines, corresponding geometric standard deviations as shaded areas and observed data as dots (± standard deviation, if reported) [96]. ALP: alprazolam, DDI: drug-drug interaction, n: number of study participants, PAR: paroxetine, po: oral, q.d.: once daily.

S6.1.3 Plasma Concentration-Time Profiles (Linear Representation)

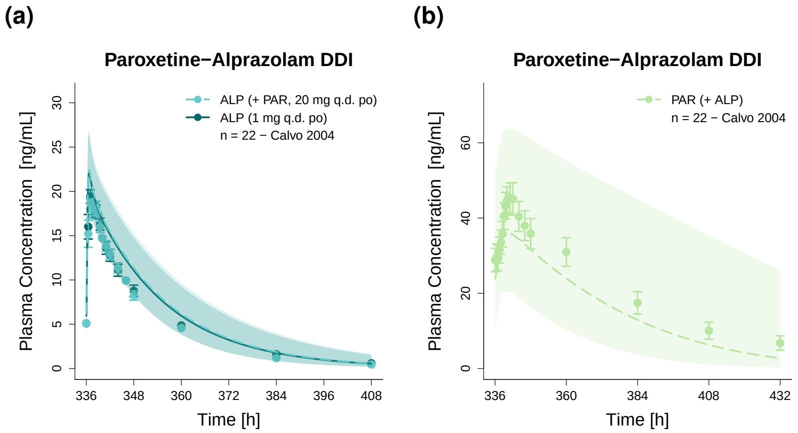

Figure S15: Predicted compared to observed plasma concentration-time profiles of (a) alprazolam alone (solid line) and after pretreatment (dashed line) with paroxetine (linear representation). Population predicted (1000 individuals) geometric means are shown as lines, corresponding geometric standard deviations as shaded areas and observed data as dots (± standard deviation, if reported) [96]. ALP: alprazolam, DDI: drug-drug interaction, n: number of study participants, PAR: paroxetine, po: oral, q.d.: once daily.

S6.1.4 DDI AUC_{last} and C_{max} Ratios

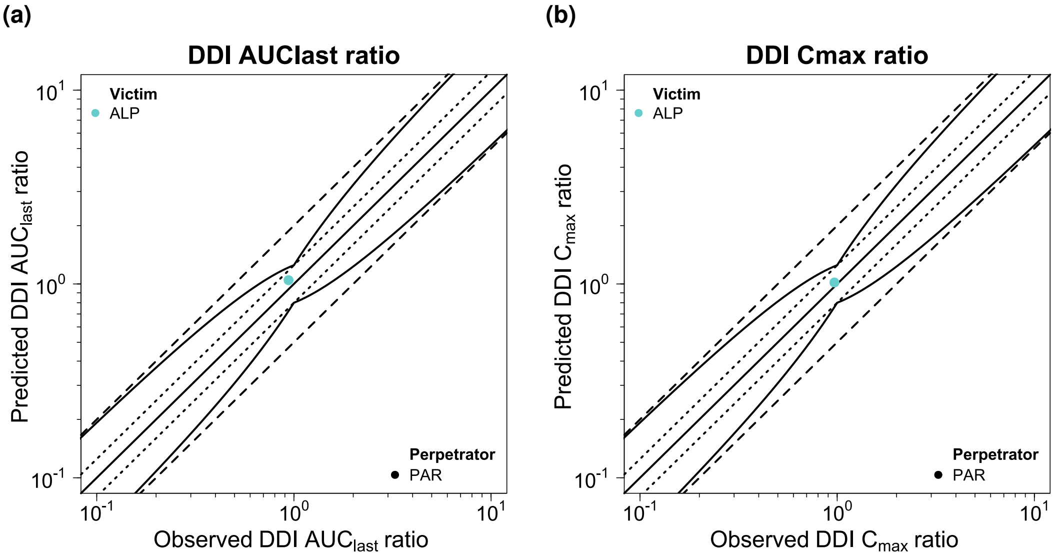

Figure S16: Goodness-of-fit plots comparing predicted and observed DDI AUC_{last} and C_{max} ratios for victim drug alprazolam. The solid line marks the line of identity. Dotted lines indicate 1.25-fold, dashed lines indicate 2-fold deviation. Prediction success limits proposed by Guest et al. [2] are shown as curved lines (including 20% variability). ALP: alprazolam, AUC_{last}: area under the plasma concentration-time curve calculated between the first and last concentration measurement, C_{max}: maximum plasma concentration, DDI: drug-drug interaction, PAR: paroxetine.

S6.1.5 Geometric Mean Fold Errors of Predicted DDI AUC_{last} and C_{max} Ratios

Table S15: Predicted and observed DDI AUC_{last} and C_{max} ratios involving **alprazolam** as victim drug

Drug administration			DDI AUC_{last} ratio			DDI C_{max} ratio				
**Perpetrator**	**Alprazolam**	**t_{last} [h]**	**Pred**	**Obs**	**Pred/Obs**	**Pred**	**Obs**	**Pred/Obs**	**Molecule**	**Reference**
Paroxetine										
20 mg q.d. po	1 mg q.d. po	72	1.05	0.94	1.11	1.02	0.97	1.05	ALP	Calvo 2004 [96]
**Overall GMFE (range):**			**1.11 (-), 1/1 with GMFE $\leq$ 2**			**1.05 (-), 1/1 with GMFE $\leq$ 2**				

ALP: alprazolam, AUC_{last}: area under the plasma concentration-time curve calculated between the first and last concentration measurement, C_{max}: maximum plasma concentration, DDI: drug-drug interaction, GMFE: geometric mean fold error, obs: observed, PAR: paroxetine, po: oral, pred: predicted, q.d.: once daily, t_{last}: time of the last concentration measurement. If perpetrator or victim drugs were applied in form of salts, the respective dose of base was calculated and incorporated in simulations.

S6.2 Atomoxetine

S6.2.1 Clinical Studies

Table S16: Clinical study data used for DD(G)I model development with **atomoxetine** as victim

Drug administration										
**Perpetrator**	**Atomoxetine**	**n**	**Population[a]**	**Fem. [%]**	**Age [years]**	**Weight [kg]**	**BMI [kg/m²]**	**Phenotype/AS**	**Molecule**	**Reference**
Bupropion										
150/300 mg q.d. po	25 mg s.d. po	2	European [3]	-	(18–55)	-	-	PM	ATO	Todor 2016 [74]
150/300 mg q.d. po	25 mg s.d. po	18	European [3]	-	(18–55)	-	-	NM	ATO	Todor 2016 [74]
Fluvoxamine										
50/100 mg q.d. po	25 mg s.d. po	18	European [3]	-	(18–55)	-	(19–25)	NM	ATO	Todor 2017 [97]
Paroxetine										
20 mg q.d. po	20 mg s.d. po	7	Asian [98]	8	22.7±1.9	-	21.4±1.6	AS = 0	ATO	Jung 2020 [71]
20 mg q.d. po	20 mg s.d. po	9	Asian [98]	8	23.2±1.4	-	21.3±1.3	AS = 1.25	ATO	Jung 2020 [71]
20 mg q.d. po	20 mg s.d. po	10	Asian [98]	8	22±1.8	-	21.8±1.8	AS = 2	ATO	Jung 2020 [71]
20 mg b.i.d./q.d. po	25 mg s.d. po	22	European [3]	32	25.3±2.3	-	24.1±3.1	-	ATO	Todor 2015 [99]
20 mg q.d. po	20 mg b.i.d. po	22	American [7]	23	38 (20–49)	-	23.8 (fem.)<br>24.4 (male)	NM	ATO, PAR	Belle 2002 [100]

AS: CYP2D6 activity score, ATO: atomoxetine, b.i.d.: twice daily, BMI: body mass index, DD(G)I: drug-drug(-gene) interaction, fem: females, n: number of study participants, NM: CYP2D6 normal metabolizer, PAR: paroxetine, PM: CYP2D6 poor metabolizer, po: oral, q.d.: once daily, s.d: single dose, -: not available. Values are given as mean (range). If perpetrator or victim drugs were applied in form of salts, the respective dose of base was calculated and incorporated in simulations. [a] Population used in simulations.

S6.2.2 Plasma Concentration-Time Profiles (Semilogarithmic Representation)

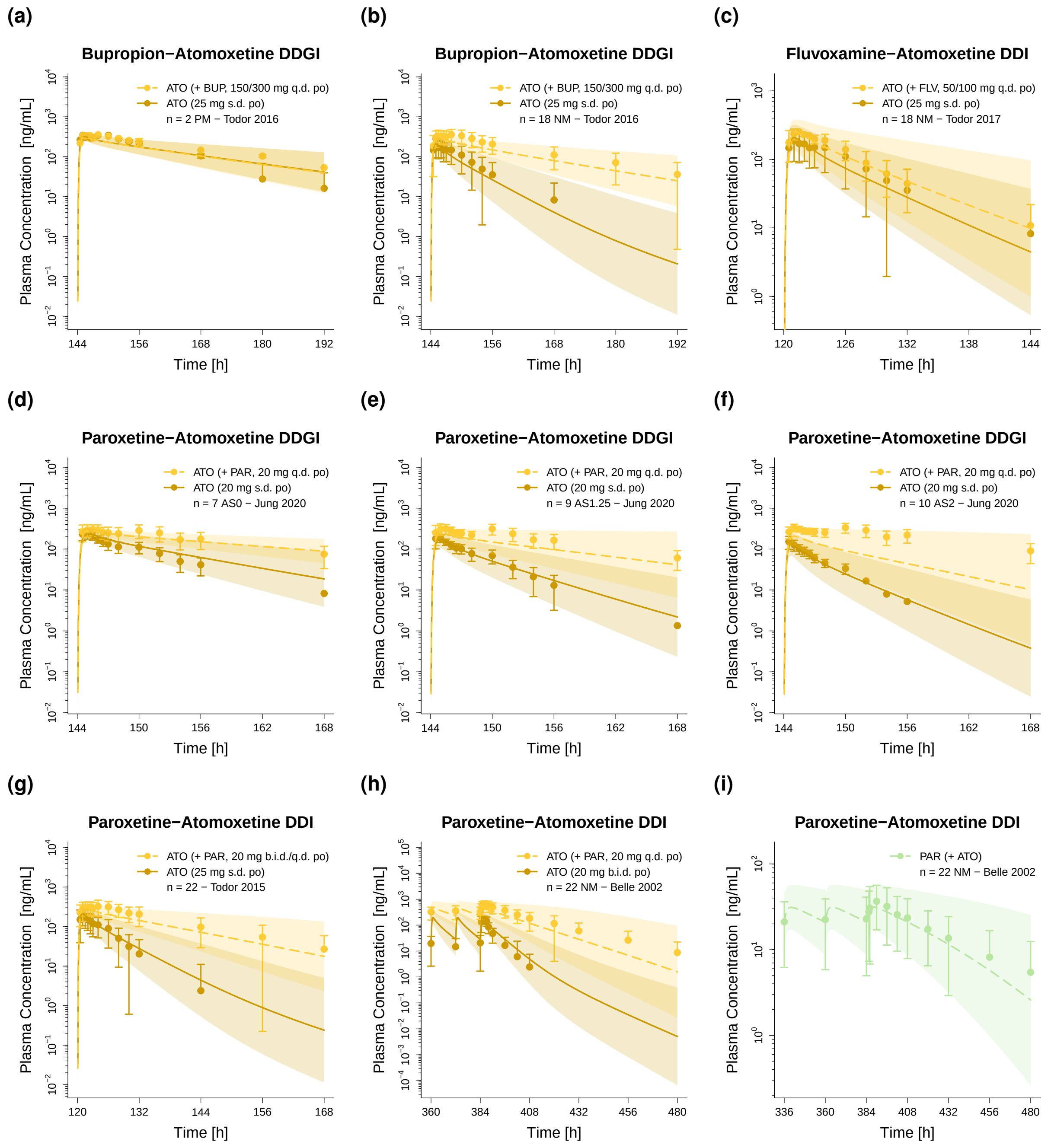

Figure S17: Predicted compared to observed plasma concentration-time profiles of atomoxetine alone (solid line) and after pretreatment and/or concomitant administration (dashed line) of (a–b) bupropion, (c) fluvoxamine and (d–h) paroxetine (semilogarithmic representation). Population predicted (1000 individuals) geometric means are shown as lines, corresponding geometric standard deviations as shaded areas and observed data as dots (± standard deviation, if reported) [71, 74, 97, 99, 100]. AS: CYP2D6 activity score, b.i.d.: twice daily, BUP: bupropion, DD(G)I: drug-drug(-gene) interaction, FLV: fluvoxamine, n: number of study participants, NM: CYP2D6 normal metabolizer, PAR: paroxetine, PM: CYP2D6 poor metabolizer, po: oral, q.d.: once daily, s.d.: single dose.

S6.2.3 Plasma Concentration-Time Profiles (Linear Representation)

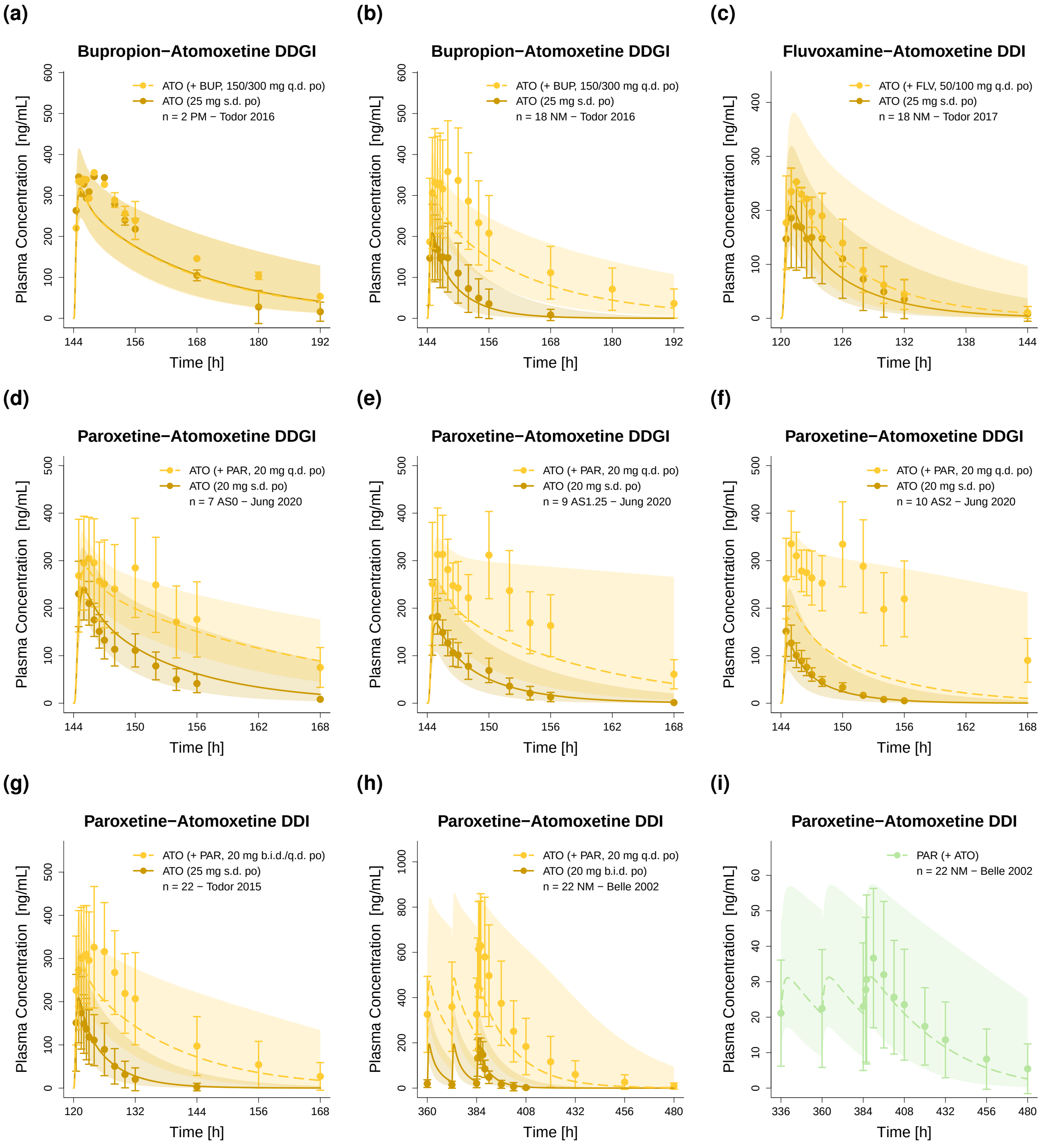

Figure S18: Predicted compared to observed plasma concentration-time profiles of atomoxetine alone (solid line) and after pretreatment and/or concomitant administration (dashed line) of (a–b) bupropion, (c) fluvoxamine and (d–h) paroxetine (linear representation). Population predicted (1000 individuals) geometric means are shown as lines, corresponding geometric standard deviations as shaded areas and observed data as dots (± standard deviation, if reported) [71, 74, 97, 99, 100]. AS: CYP2D6 activity score, b.i.d.: twice daily, BUP: bupropion, DD(G)I: drug-drug(-gene) interaction, FLV: fluvoxamine, n: number of study participants, NM: CYP2D6 normal metabolizer, PAR: paroxetine, PM: CYP2D6 poor metabolizer, po: oral, q.d.: once daily, s.d.: single dose.

S6.2.4 DDI AUC_{last} and C_{max} Ratios

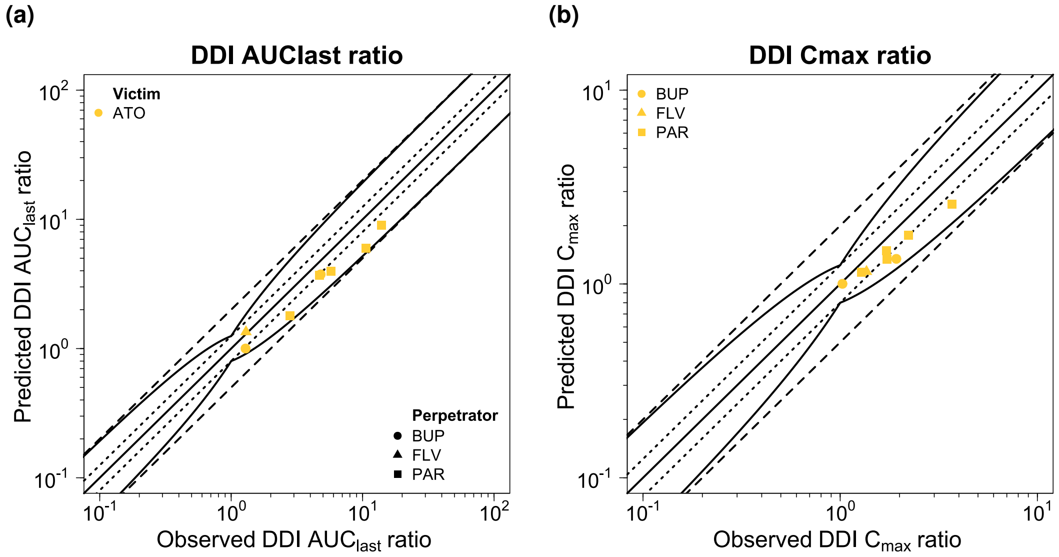

Figure S19: Goodness-of-fit plots comparing predicted and observed DDI AUC_{last} and C_{max} ratios for victim drug atomoxetine. The solid line marks the line of identity. Dotted lines indicate 1.25-fold, dashed lines indicate 2-fold deviation. Prediction success limits proposed by Guest et al. [2] are shown as curved lines (including 20% variability). ATO: atomoxetine, AUC_{last}: area under the plasma concentration-time curve calculated between the first and last concentration measurement, BUP: bupropion, C_{max}: maximum plasma concentration, FLV: fluvoxamine, PAR: paroxetine.

S6.2.5 Geometric Mean Fold Errors of Predicted DDI AUC_{last} and C_{max} Ratios

Table S17: Predicted and observed DDI AUC_{last} and C_{max} ratios involving **atomoxetine** as victim drug

Drug administration			DDI AUC_{last} ratio			DDI C_{max} ratio					
**Perpetrator**	**Atomoxetine**	**t_{last} [h]**	**Pred**	**Obs**	**Pred/Obs**	**Pred**	**Obs**	**Pred/Obs**	**Phenotype/AS**	**Molecule**	**Reference**
Bupropion											
150/300 mg q.d. po	25 mg s.d. po	48	1.00	1.29	0.78	1.00	1.03	0.98	PM	ATO	Todor 2016 [74]
150/300 mg q.d. po	25 mg s.d. po	48	3.75	4.80	0.78	1.35	1.93	0.70	NM	ATO	Todor 2016 [74]
**Mean GMFE (range):**			**1.32 (1.28–1.36), 2/2 with GMFE $\leq$ 2**			**1.19 (1.02–1.6), 2/2 with GMFE $\leq$ 2**					
Fluvoxamine											
50/100 mg q.d. po	25 mg s.d. po	24	1.35	1.30	1.04	1.16	1.36	0.85	NM	ATO	Todor 2017 [97]
**Mean GMFE (range):**			**1.04 (-), 1/1 with GMFE $\leq$ 2**			**1.17 (-), 1/1 with GMFE $\leq$ 2**					
Paroxetine											
20 mg q.d. po	20 mg s.d. po	24	1.80	2.80	0.64	1.15	1.28	0.90	AS = 0	ATO	Jung 2020 [71]
20 mg q.d. po	20 mg s.d. po	24	3.69	4.70	0.79	1.49	1.71	0.87	AS = 1.25	ATO	Jung 2020 [71]
20 mg q.d. po	20 mg s.d. po	24	5.98	10.57	0.57	1.78	2.21	0.87	AS = 2	ATO	Jung 2020 [71]
20 mg b.i.d./q.d. po	25 mg s.d. po	48	3.96	5.73	0.69	1.34	1.73	0.78	-	ATO	Todor 2015 [99]
20 mg q.d. po	20 mg b.i.d. po	96	9.04	13.87	0.65	2.58	3.70	0.70	NM	ATO	Belle 2002 [100]
**Mean GMFE (range):**			**1.52 (1.27–1.77), 5/5 with GMFE $\leq$ 2**			**1.25 (1.12–1.43), 5/5 with GMFE $\leq$ 2**					
**Overall GMFE (range):**			**1.41 (1.04–1.77), 8/8 with GMFE $\leq$ 2**			**1.22 (1.02–1.43), 8/8 with GMFE $\leq$ 2**					

AS: CYP2D6 activity score, ATO: atomoxetine, AUC_{last}: area under the plasma concentration-time curve calculated between the first and last concentration measurement, b.i.d.: twice daily, C_{max}: maximum plasma concentration, DDI: drug-drug interaction, GMFE: geometric mean fold error, NM: CYP2D6 normal metabolizer, obs: observed, PM: CYP2D6 poor metabolizer, po: oral, pred: predicted, q.d.: once daily, s.d.: single dose, t_{last}: time of the last concentration measurement. If perpetrator or victim drugs were applied in form of salts, the respective dose of base was calculated and incorporated in simulations.

S6.3 (*E*)-Clomiphene

S6.3.1 Clinical Studies

Table S18: Clinical study data used for DD(G)I model development with **(*E*)-clomiphene** as victim

Drug administration										
Perpetrator	***(E)-Clomiphene***	**n**	**Population[a]**	**Fem. [%]**	**Age [years]**	**Weight [kg]**	**BMI [kg/m²]**	**AS**	**Molecule**	**Reference**
Clarithromycin										
500 mg b.i.d po	42 mg s.d. po	5	European [3]	100	25.0 (22–29)	61.1 (50.0–70.0)	21.4 (20.6–22.9)	AS = 0	CLO, OHC, NDC, HDC	Mürdter 2016 [76]
500 mg b.i.d po	42 mg s.d. po	4	European [3]	100	24.3 (21–30)	59.3 (55.5–64.0)	21.1 (20.3–22.0)	AS = 0.5	CLO, OHC, NDC, HDC	Mürdter 2016 [76]
500 mg b.i.d po	42 mg s.d. po	2	European [3]	100	25.5 (23–28)	68.8 (63.5–74.0)	23.6 (22.5–24.7)	AS = 1	CLO, OHC, NDC, HDC	Mürdter 2016 [76]
500 mg b.i.d po	42 mg s.d. po	3	European [3]	100	32.3 (26–43)	56.5 (48.0–63.5)	21.3 (18.8–24.2)	AS = 2	CLO, OHC, NDC, HDC	Mürdter 2016 [76]
500 mg b.i.d po	42 mg s.d. po	3	European [3]	100	25.7 (22–28)	61.7 (54.0–73.0)	22.6 (20.3–23.8)	AS = 3	CLO, OHC, NDC, HDC	Mürdter 2016 [76]
Paroxetine										
40 mg q.d. po	42 mg s.d. po	4	European [3]	100	24.8 (22–29)	60.5 (50.0–70.0)	21.5 (20.6–22.9)	AS = 0	CLO, OHC, NDC, HDC	Mürdter 2016 [76]
40 mg q.d. po	42 mg s.d. po	4	European [3]	100	24.3 (21–30)	59.3 (55.5–64.0)	21.1 (20.3–22.0)	AS = 0.5	CLO, OHC, NDC, HDC	Mürdter 2016 [76]
40 mg q.d. po	42 mg s.d. po	2	European [3]	100	25.5 (23–28)	68.8 (63.5–74.0)	23.6 (22.5–24.7)	AS = 1	CLO, OHC, NDC, HDC	Mürdter 2016 [76]
40 mg q.d. po	42 mg s.d. po	3	European [3]	100	32.3 (26–43)	56.5 (48.0–63.5)	21.3 (18.8–24.2)	AS = 2	CLO, OHC, NDC, HDC	Mürdter 2016 [76]
40 mg q.d. po	42 mg s.d. po	3	European [3]	100	25.7 (22–28)	61.7 (54.0–73.0)	22.6 (20.3–23.8)	AS = 3	CLO, OHC, NDC, HDC	Mürdter 2016 [76]

AS: CYP2D6 activity score, b.i.d.: twice daily, BMI: body mass index, CLO: (*E*)-clomiphene, DD(G)I: drug-drug(-gene) interaction, fem: females, HDC: (*E*)-4-hydroxy-N-desethylclomiphene, n: number of study participants, NDC: (*E*)-N-desethylclomiphene, OHC: (*E*)-4-hydroxyclomiphene, po: oral, q.d.: once daily, s.d: single dose. Values are given as mean (range). If perpetrator or victim drugs were applied in form of salts, the respective dose of base was calculated and incorporated in simulations. [a] Population used in simulations.

S6.3.2 Plasma Concentration-Time Profiles (Semilogarithmic Representation)

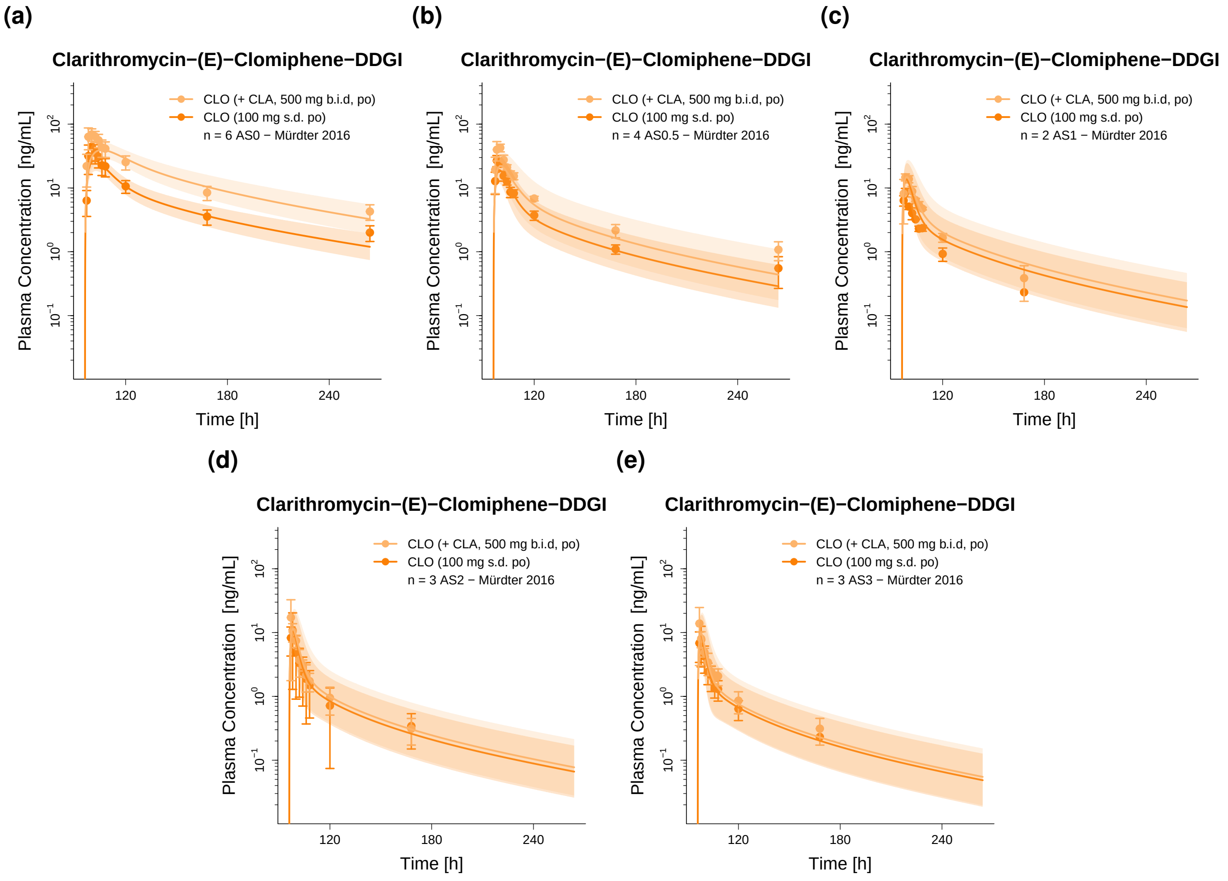

Figure S20: Predicted compared to observed plasma concentration-time profiles of (*E*)-clomiphene alone (solid line) and after pretreatment and concomitant administration (dashed line) of clarithromycin (semilogarithmic representation). Population predicted (1000 individuals) geometric means are shown as lines, corresponding geometric standard deviations as shaded areas and observed data as dots (± standard deviation, if reported) [76]. AS: CYP2D6 activity score, b.i.d.: twice daily, CLA: clarithromycin, CLO: (*E*)-clomiphene, DDGI: drug-drug-gene interaction, n: number of study participants, po: oral, s.d.: single dose.

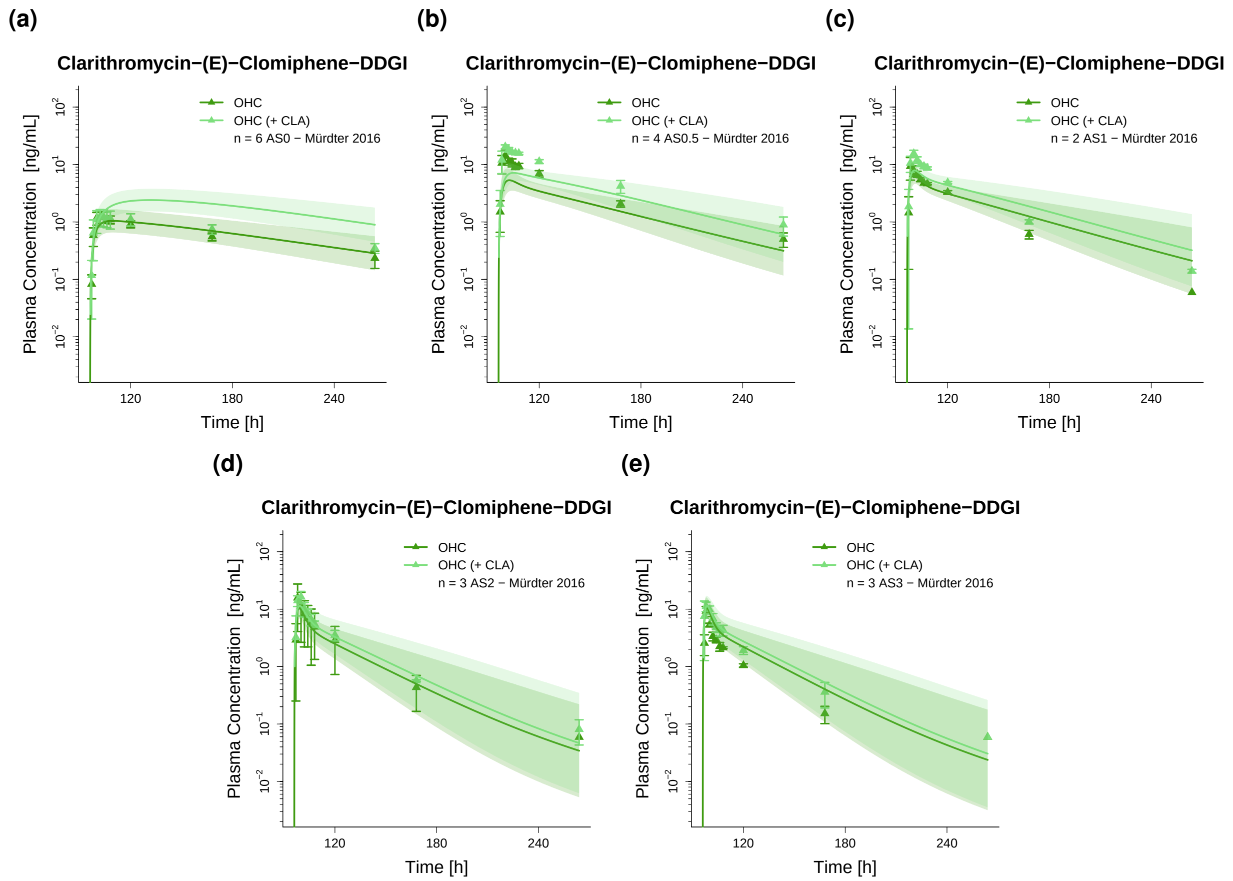

Figure S21: Predicted compared to observed plasma concentration-time profiles of (*E*)-4-hydroxyclomiphene alone (solid line) and after pretreatment and concomitant administration (dashed line) of clarithromycin (semilogarithmic representation). Population predicted (1000 individuals) geometric means are shown as lines, corresponding geometric standard deviations as shaded areas and observed data as triangles (± standard deviation, if reported) [76]. AS: CYP2D6 activity score, CLA: clarithromycin, DDGI: drug-drug-gene interaction, n: number of study participants, OHC: (*E*)-4-hydroxyclomiphene.

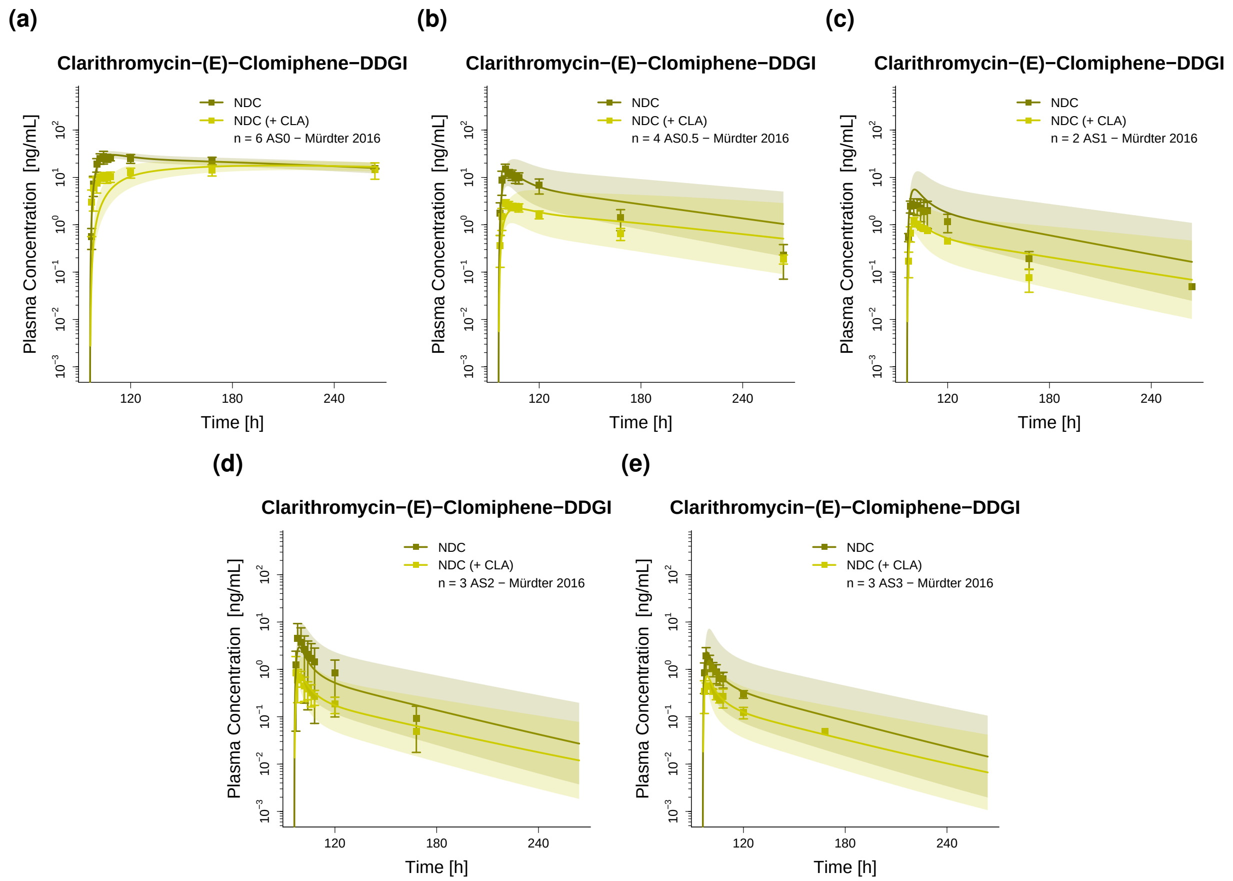

Figure S22: Predicted compared to observed plasma concentration-time profiles of (*E*)-N-desethylclomiphene alone (solid line) and after pretreatment and concomitant administration (dashed line) of clarithromycin (semilogarithmic representation). Population predicted (1000 individuals) geometric means are shown as lines, corresponding geometric standard deviations as shaded areas and observed data as triangles (± standard deviation, if reported) [76]. AS: CYP2D6 activity score, CLA: clarithromycin, DDGI: drug-drug-gene interaction, n: number of study participants, NDC: (*E*)-N-desethylclomiphene.

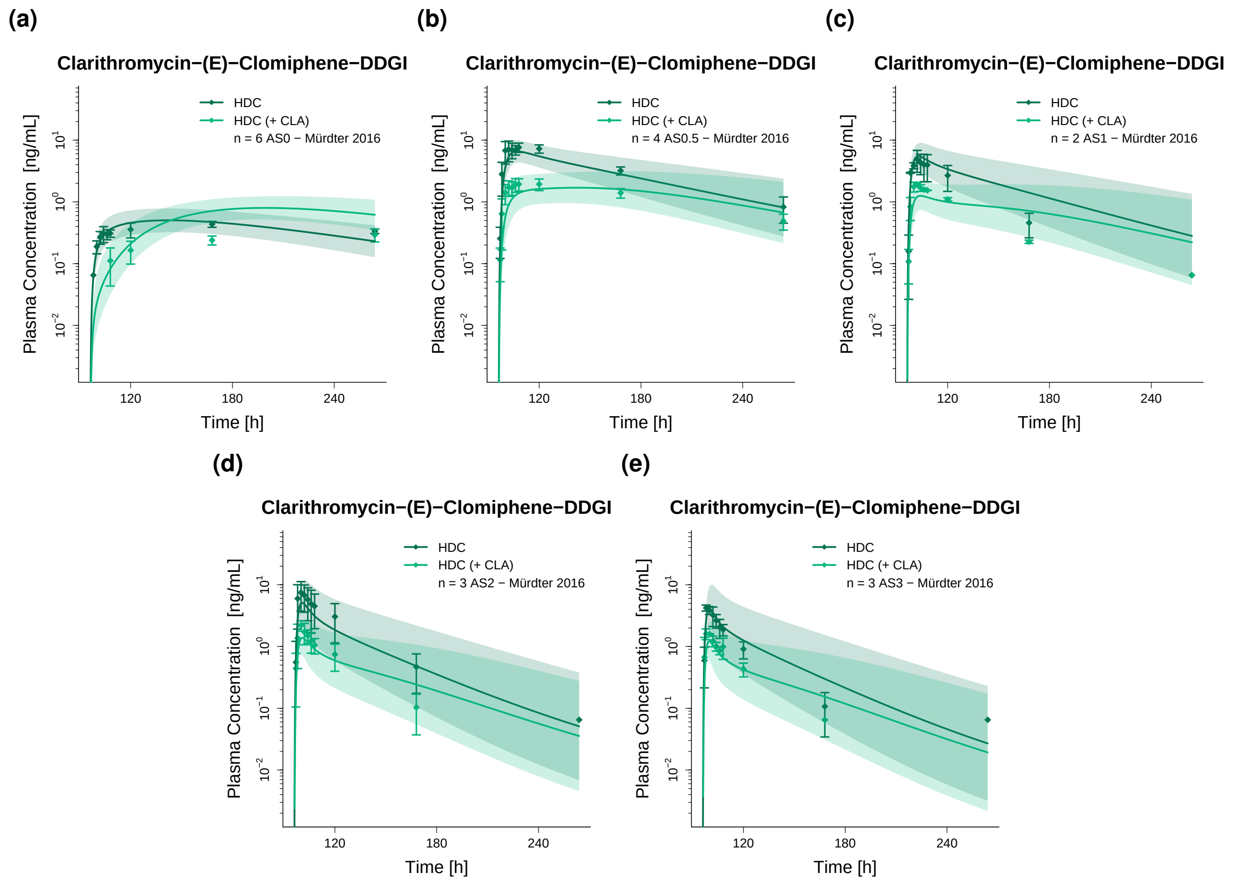

Figure S23: Predicted compared to observed plasma concentration-time profiles of (*E*)-4-hydroxy-N-desethylclomiphene alone (solid line) and after pretreatment and concomitant administration (dashed line) of clarithromycin (semilogarithmic representation). Population predicted (1000 individuals) geometric means are shown as lines, corresponding geometric standard deviations as shaded areas and observed data as triangles (± standard deviation, if reported) [76]. AS: CYP2D6 activity score, CLA: clarithromycin, DDGI: drug-drug-gene interaction, HDC: (*E*)-4-hydroxy-N-desethylclomiphene, n: number of study participants.

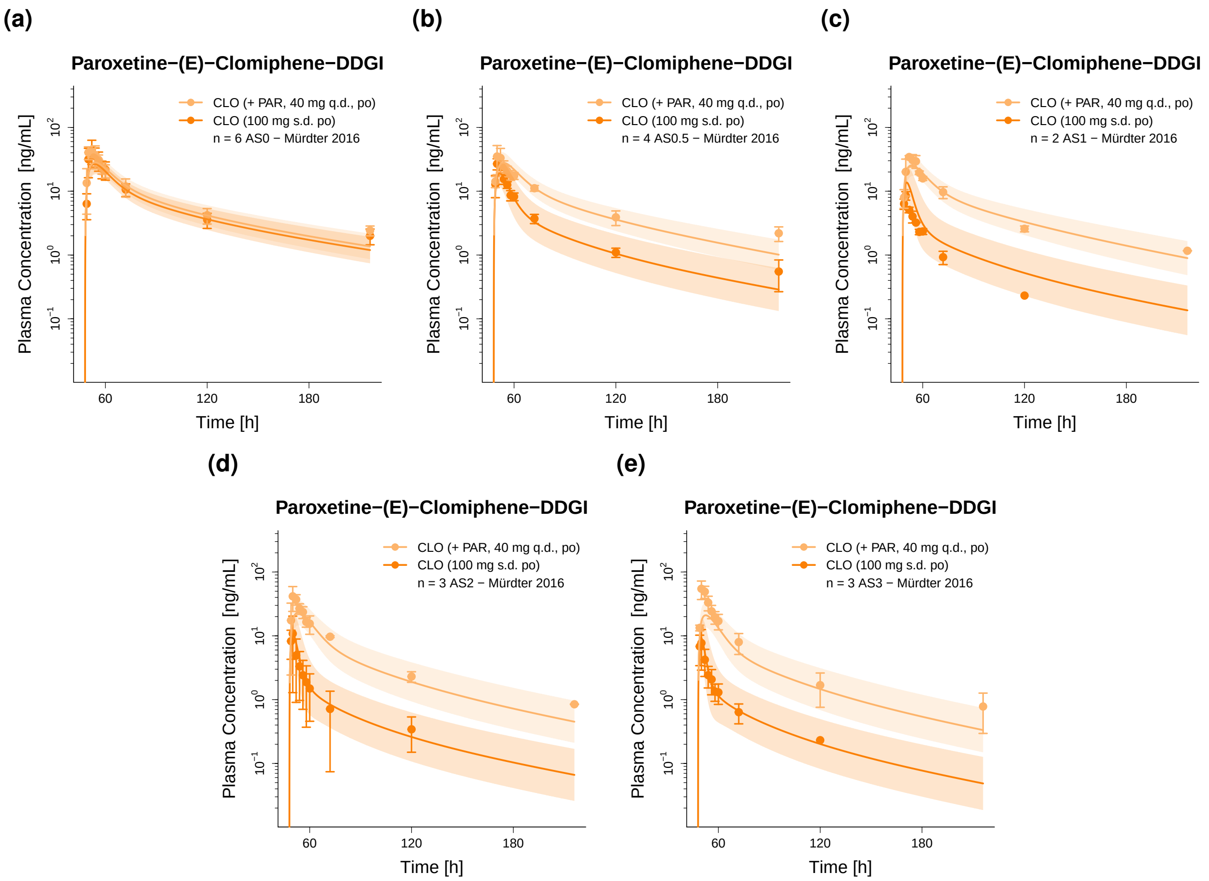

Figure S24: Predicted compared to observed plasma concentration-time profiles of (*E*)-clomiphene alone (solid line) and after pretreatment and concomitant administration (dashed line) of paroxetine (semilogarithmic representation). Population predicted (1000 individuals) geometric means are shown as lines, corresponding geometric standard deviations as shaded areas and observed data as dots (± standard deviation, if reported) [76]. AS: CYP2D6 activity score, CLO: (*E*)-clomiphene, DDGI: drug-drug-gene interaction, n: number of study participants, PAR: paroxetine, po: oral, q.d.: once daily, s.d.: single dose.

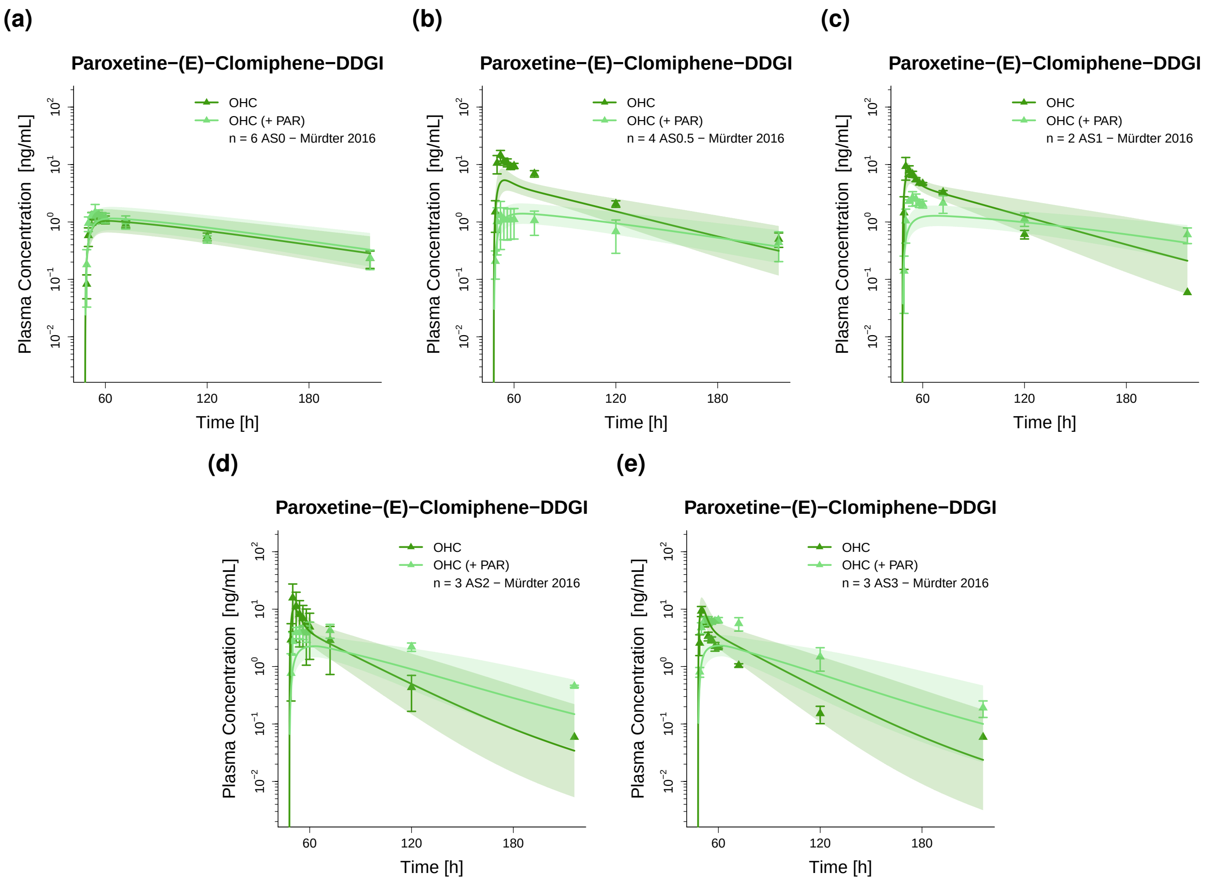

Figure S25: Predicted compared to observed plasma concentration-time profiles of (*E*)-4-hydroxyclomiphene alone (solid line) and after pretreatment and concomitant administration (dashed line) of paroxetine (semilogarithmic representation). Population predicted (1000 individuals) geometric means are shown as lines, corresponding geometric standard deviations as shaded areas and observed data as triangles (± standard deviation, if reported) [76]. AS: CYP2D6 activity score, DDGI: drug-drug-gene interaction, n: number of study participants, OHC: (*E*)-4-hydroxyclomiphene, PAR: paroxetine.

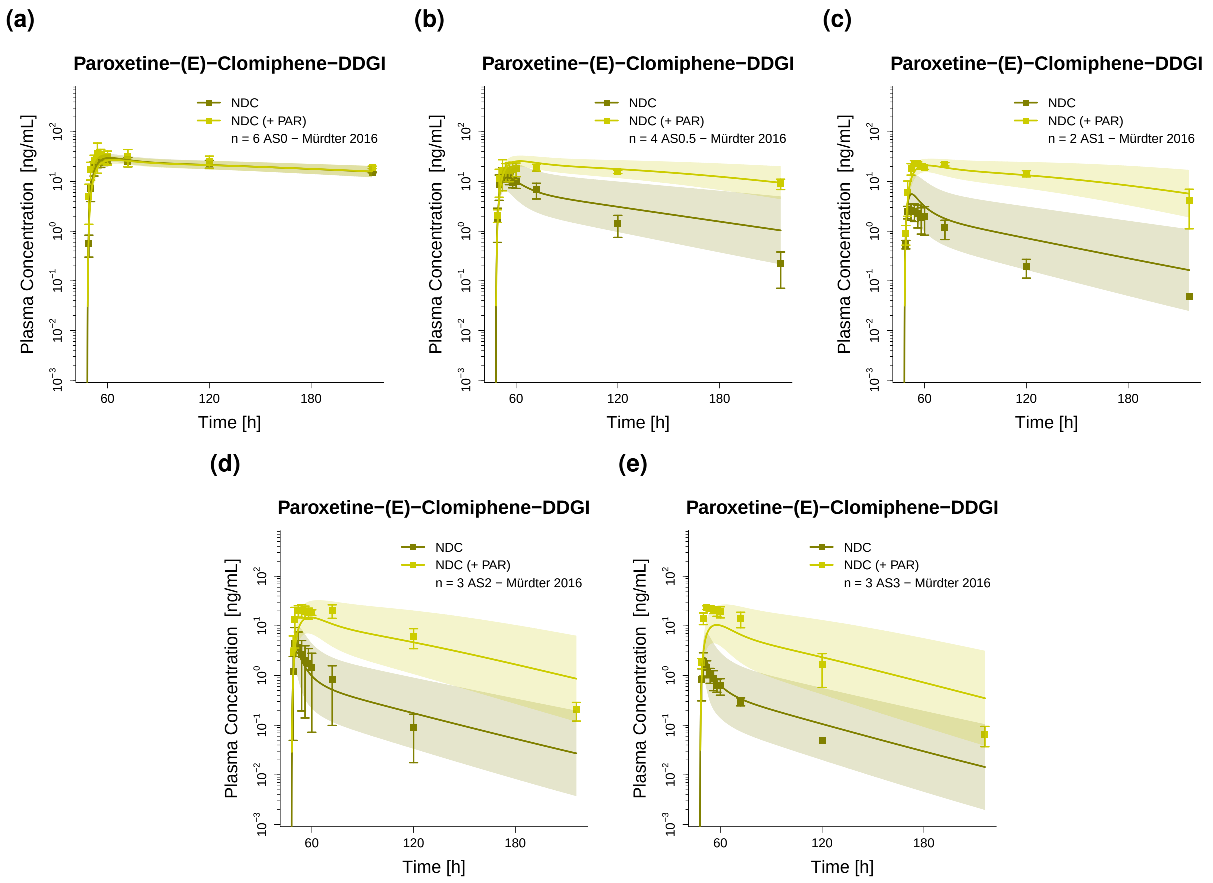

Figure S26: Predicted compared to observed plasma concentration-time profiles of (*E*)-N-desethylclomiphene alone (solid line) and after pretreatment and concomitant administration (dashed line) of paroxetine (semilogarithmic representation). Population predicted (1000 individuals) geometric means are shown as lines, corresponding geometric standard deviations as shaded areas and observed data as triangles (± standard deviation, if reported) [76]. AS: CYP2D6 activity score, DDGI: drug-drug-gene interaction, n: number of study participants, NDC: (*E*)-N-desethylclomiphene, PAR: paroxetine.

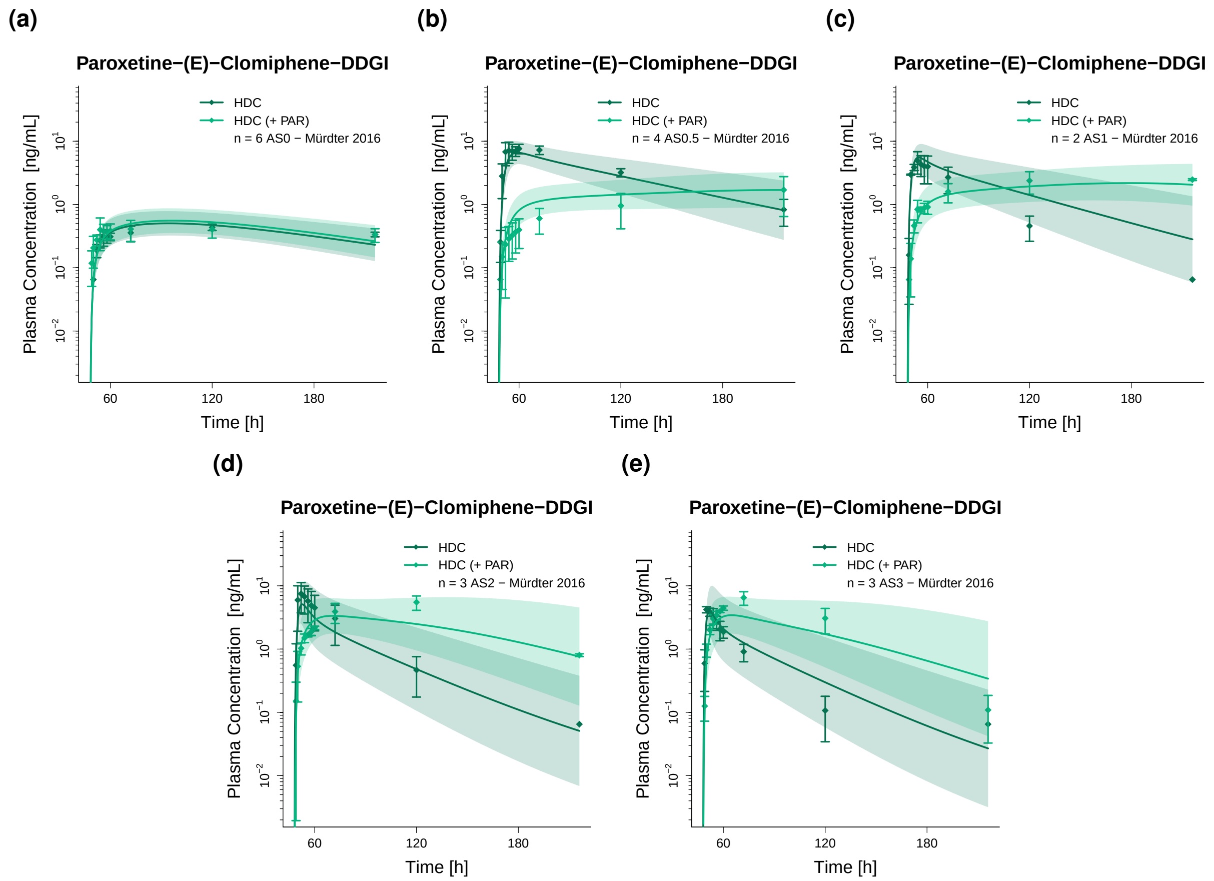

Figure S27: Predicted compared to observed plasma concentration-time profiles of (*E*)-4-hydroxy-N-desethylclomiphene alone (solid line) and after pretreatment and concomitant administration (dashed line) of paroxetine (semilogarithmic representation). Population predicted (1000 individuals) geometric means are shown as lines, corresponding geometric standard deviations as shaded areas and observed data as triangles (± standard deviation, if reported) [76]. AS: CYP2D6 activity score, DDGI: drug-drug-gene interaction, HDC: (*E*)-4-hydroxy-N-desethylclomiphene, n: number of study participants, PAR: paroxetine.

S6.3.3 Plasma Concentration-Time Profiles (Linear Representation)

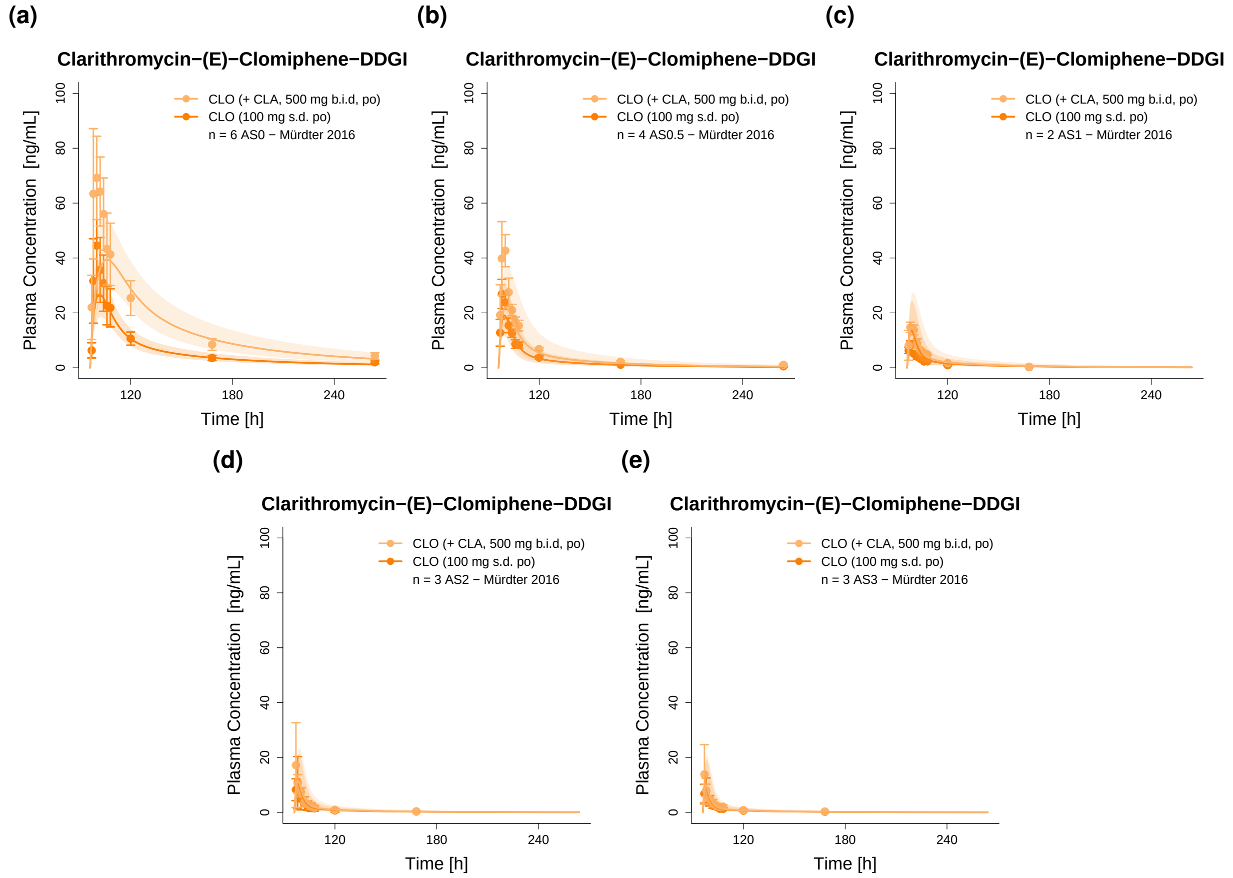

Figure S28: Predicted compared to observed plasma concentration-time profiles of (*E*)-clomiphene alone (solid line) and after pretreatment and concomitant administration (dashed line) of clarithromycin (linear representation). Population predicted (1000 individuals) geometric means are shown as lines, corresponding geometric standard deviations as shaded areas and observed data as dots (± standard deviation, if reported) [76]. AS: CYP2D6 activity score, b.i.d.: twice daily, CLA: clarithromycin, CLO: (*E*)-clomiphene, DDGI: drug-drug-gene interaction, n: number of study participants, po: oral, s.d.: single dose.

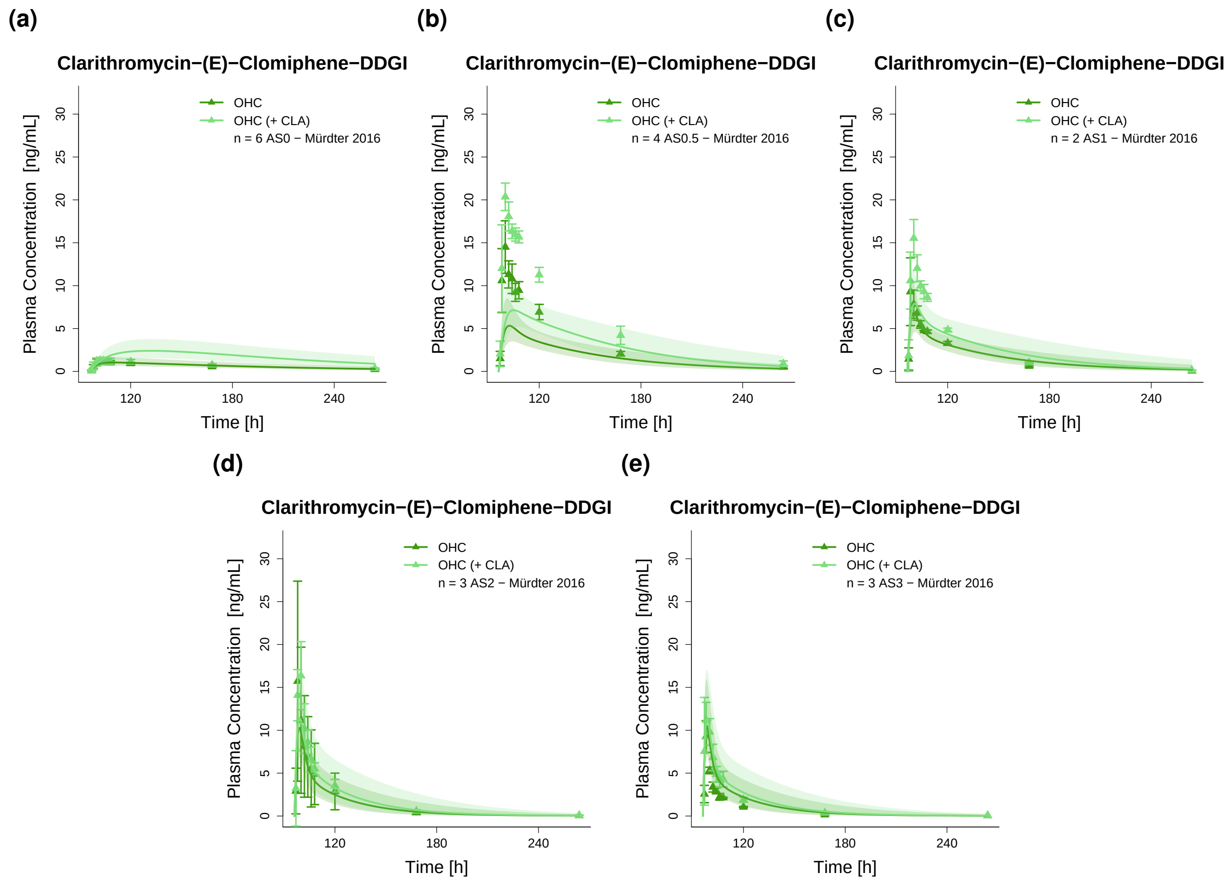

Figure S29: Predicted compared to observed plasma concentration-time profiles of (*E*)-4-hydroxyclomiphene alone (solid line) and after pretreatment and concomitant administration (dashed line) of clarithromycin (linear representation). Population predicted (1000 individuals) geometric means are shown as lines, corresponding geometric standard deviations as shaded areas and observed data as triangles (± standard deviation, if reported) [76]. AS: CYP2D6 activity score, CLA: clarithromycin, DDGI: drug-drug-gene interaction, n: number of study participants, OHC: (*E*)-4-hydroxyclomiphene.

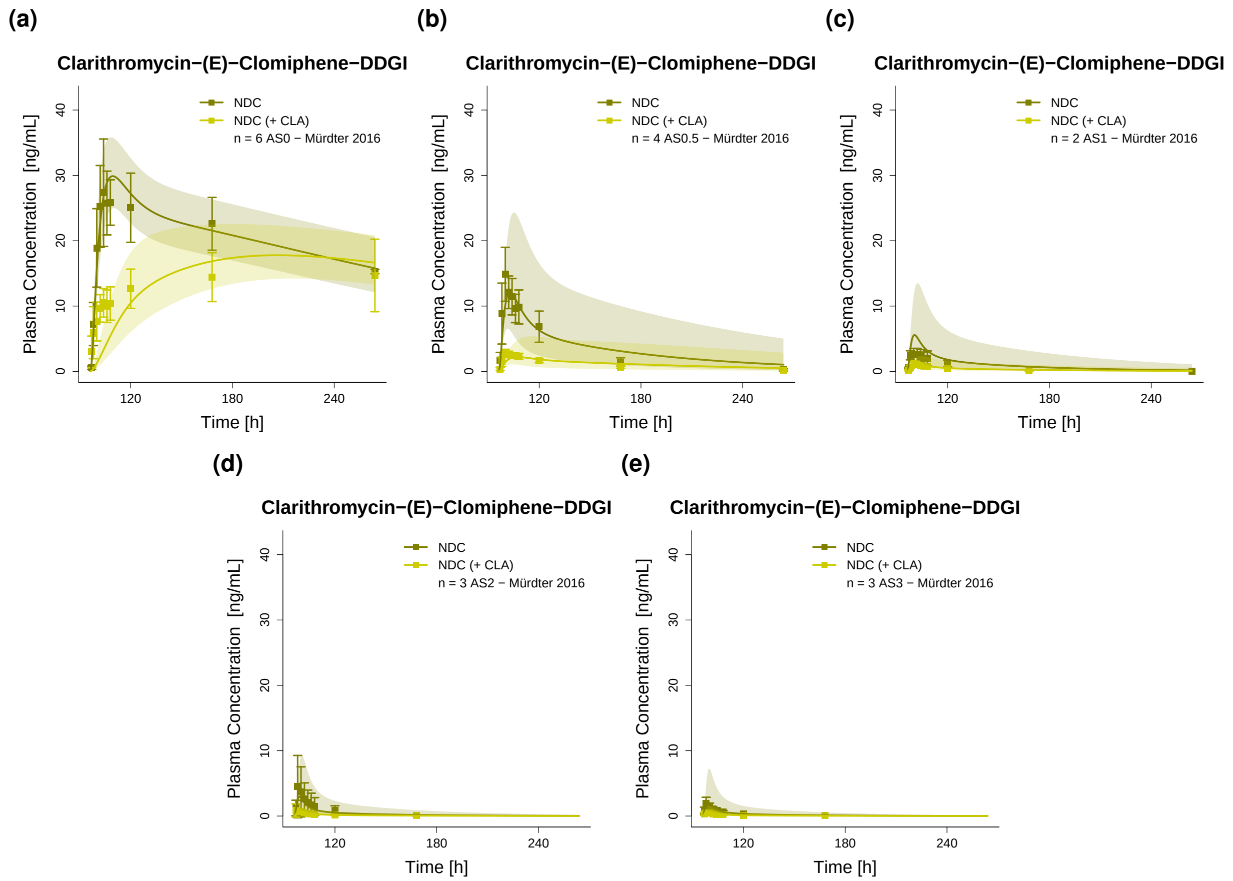

Figure S30: Predicted compared to observed plasma concentration-time profiles of (*E*)-N-desethylclomiphene alone (solid line) and after pretreatment and concomitant administration (dashed line) of clarithromycin (linear representation). Population predicted (1000 individuals) geometric means are shown as lines, corresponding geometric standard deviations as shaded areas and observed data as triangles (± standard deviation, if reported) [76]. AS: CYP2D6 activity score, CLA: clarithromycin, DDGI: drug-drug-gene interaction, n: number of study participants, NDC: (*E*)-N-desethylclomiphene.

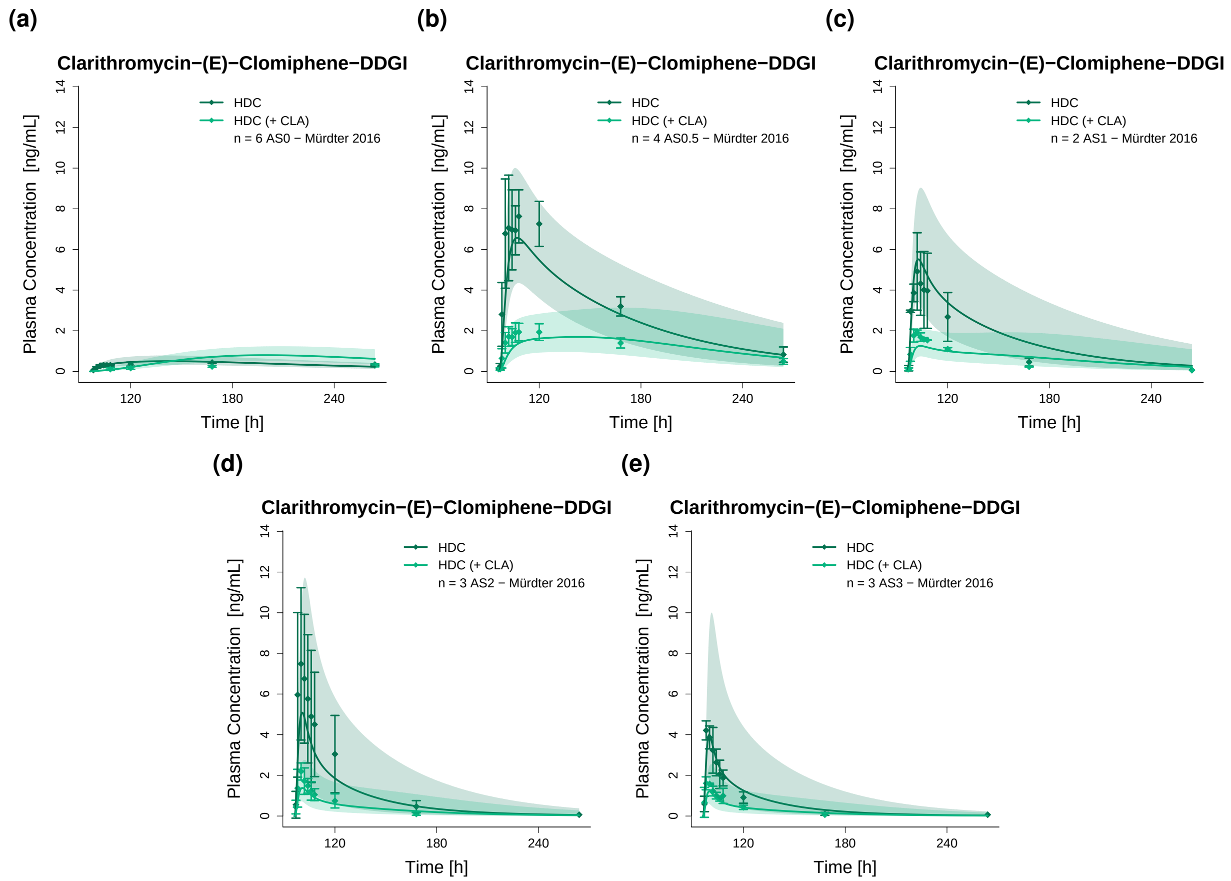

Figure S31: Predicted compared to observed plasma concentration-time profiles of (*E*)-4-hydroxy-N-desethylclomiphene alone (solid line) and after pretreatment and concomitant administration (dashed line) of clarithromycin (linear representation). Population predicted (1000 individuals) geometric means are shown as lines, corresponding geometric standard deviations as shaded areas and observed data as triangles (± standard deviation, if reported) [76]. AS: CYP2D6 activity score, CLA: clarithromycin, DDGI: drug-drug-gene interaction, HDC: (*E*)-4-hydroxy-N-desethylclomiphene, n: number of study participants.

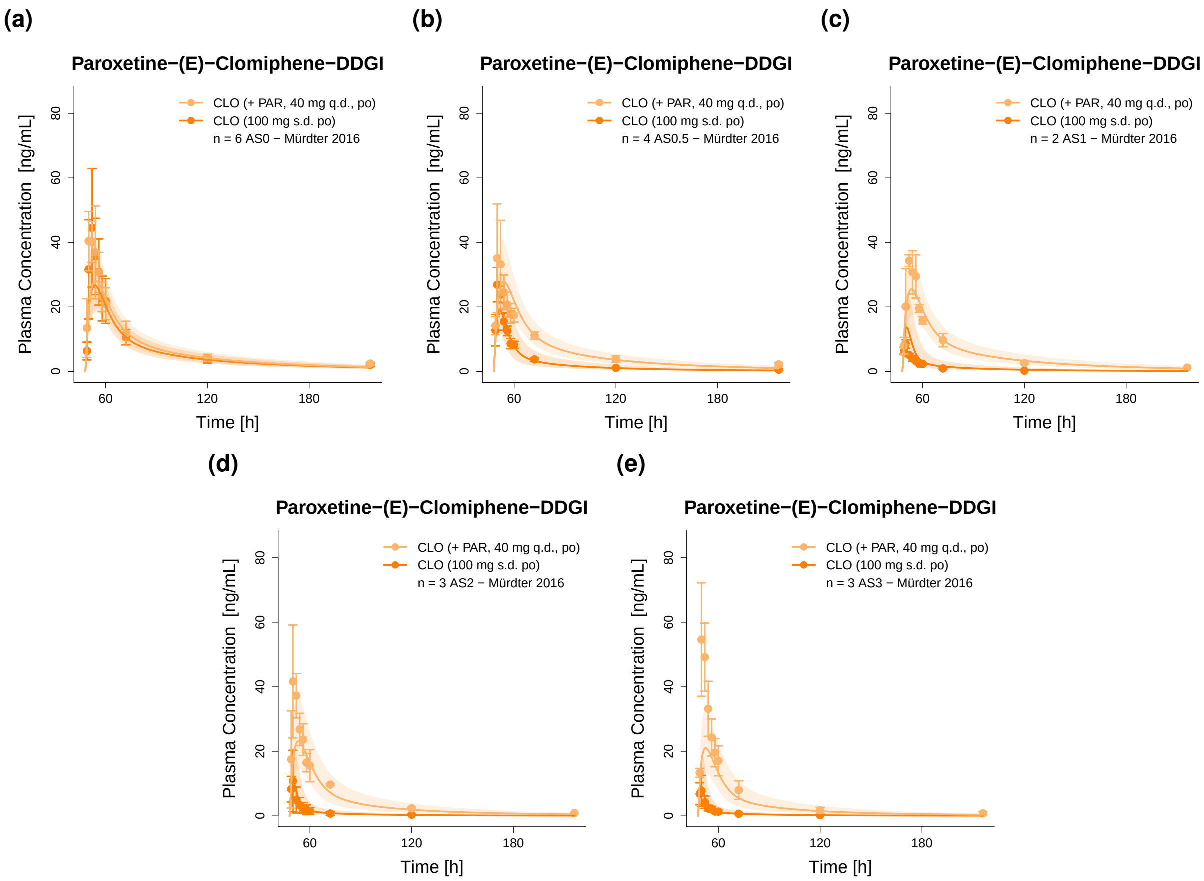

Figure S32: Predicted compared to observed plasma concentration-time profiles of (*E*)-clomiphene alone (solid line) and after pretreatment and concomitant administration (dashed line) of paroxetine (linear representation). Population predicted (1000 individuals) geometric means are shown as lines, corresponding geometric standard deviations as shaded areas and observed data as dots (± standard deviation, if reported) [76]. AS: CYP2D6 activity score, CLO: (*E*)-clomiphene, DDGI: drug-drug-gene interaction, n: number of study participants, PAR: paroxetine, po: oral, q.d.: once daily, s.d.: single dose.

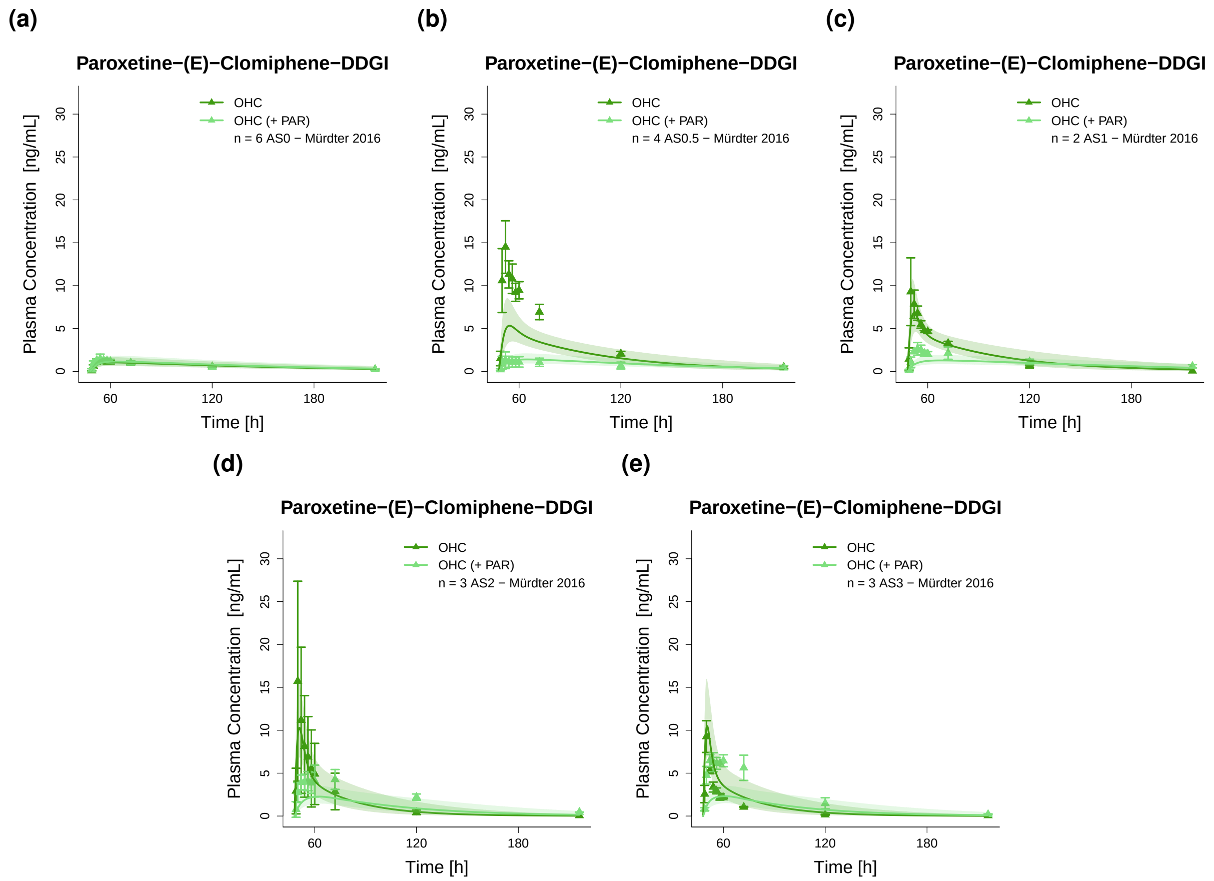

Figure S33: Predicted compared to observed plasma concentration-time profiles of (*E*)-4-hydroxyclomiphene alone (solid line) and after pretreatment and concomitant administration (dashed line) of paroxetine (linear representation). Population predicted (1000 individuals) geometric means are shown as lines, corresponding geometric standard deviations as shaded areas and observed data as triangles (± standard deviation, if reported) [76]. AS: CYP2D6 activity score, DDGI: drug-drug-gene interaction, n: number of study participants, OHC: (*E*)-4-hydroxyclomiphene, PAR: paroxetine.

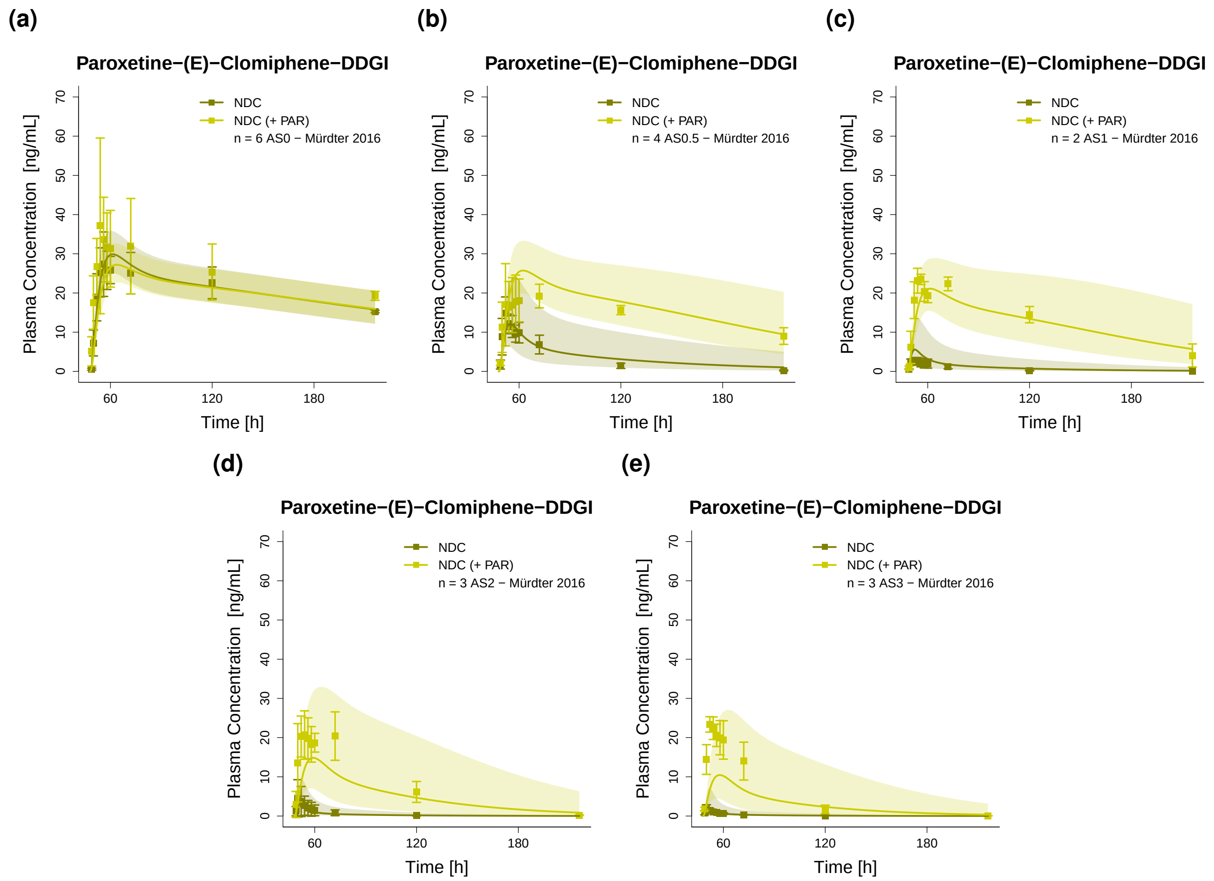

Figure S34: Predicted compared to observed plasma concentration-time profiles of (*E*)-N-desethylclomiphene alone (solid line) and after pretreatment and concomitant administration (dashed line) of paroxetine (linear representation). Population predicted (1000 individuals) geometric means are shown as lines, corresponding geometric standard deviations as shaded areas and observed data as triangles (± standard deviation, if reported) [76]. AS: CYP2D6 activity score, DDGI: drug-drug-gene interaction, n: number of study participants, NDC: (*E*)-N-desethylclomiphene, PAR: paroxetine.

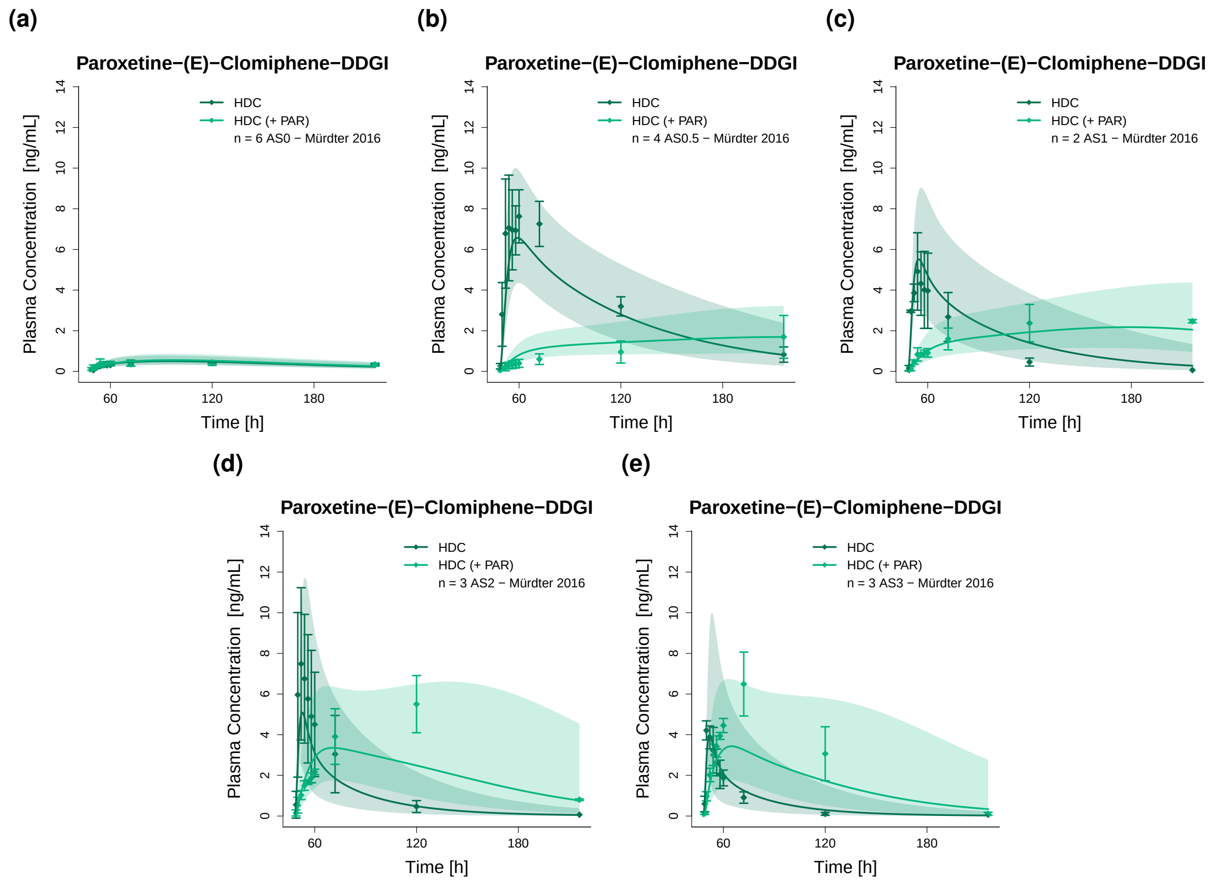

Figure S35: Predicted compared to observed plasma concentration-time profiles of (*E*)-4-hydroxy-N-desethylclomiphene alone (solid line) and after pretreatment and concomitant administration (dashed line) of paroxetine (linear representation). Population predicted (1000 individuals) geometric means are shown as lines, corresponding geometric standard deviations as shaded areas and observed data as triangles (± standard deviation, if reported) [76]. AS: CYP2D6 activity score, DDGI: drug-drug-gene interaction, HDC: (*E*)-4-hydroxy-N-desethylclomiphene, n: number of study participants, PAR: paroxetine.

S6.3.4 DDI AUC_{last} and C_{max} Ratios

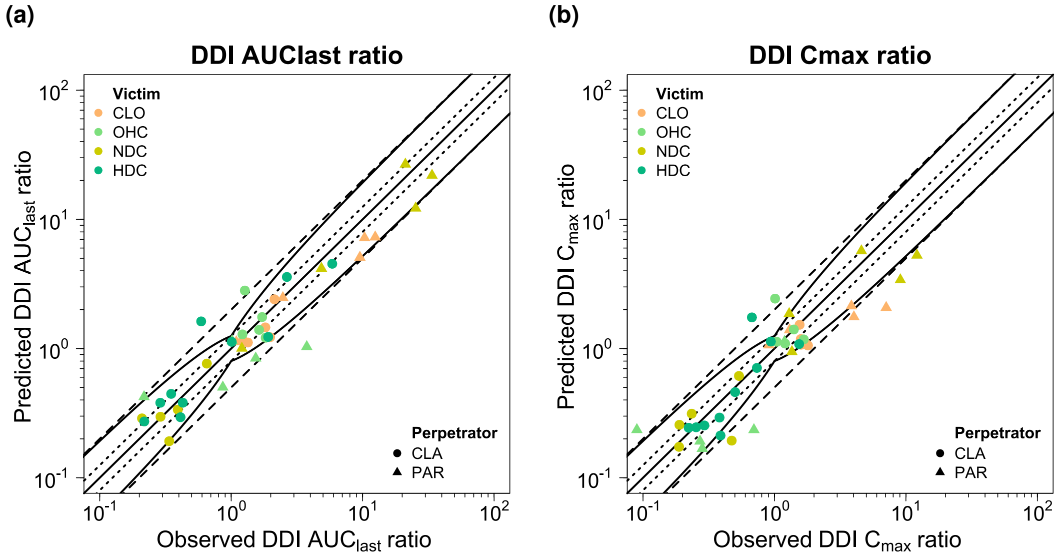

Figure S36: Goodness-of-fit plots comparing predicted and observed DDI AUC_{last} and C_{max} ratios for victim drug clomiphene. The solid line marks the line of identity. Dotted lines indicate 1.25-fold, dashed lines indicate 2-fold deviation. Prediction success limits proposed by Guest et al. [2] are shown as curved lines (including 20% variability). AUC_{last}: area under the plasma concentration-time curve calculated between the first and last concentration measurement, CLO: (*E*)-clomiphene, CLA: clarithromycin, C_{max}: maximum plasma concentration, HDC: (*E*)-4-hydroxy-N-desethylclomiphene, NDC: (*E*)-N-desethylclomiphene, OHC: (*E*)-4-hydroxyclomiphene, PAR: paroxetine.

S6.3.5 Geometric Mean Fold Errors of Predicted DDI AUC_{last} and C_{max} Ratios

Table S19: Predicted and observed DDI AUC_{last} and C_{max} ratios involving **(*E*)-clomiphene** as victim drug

Drug administration			DDI AUC_{last} ratio			DDI C_{max} ratio					
**Perpetrator**	**(E)-Clomiphene**	**t_{last} [h]**	**Pred**	**Obs**	**Pred/Obs**	**Pred**	**Obs**	**Pred/Obs**	**AS**	**Molecule**	**Reference**
Clarithromycin											
500 mg b.i.d po	42 mg s.d. po	168	2.41	2.13	1.13	1.53	1.55	0.98	AS = 0	CLO	Mürdter 2016 [76]
500 mg b.i.d po	42 mg s.d. po	168	2.81	1.27	2.21	2.44	1.01	2.41	AS = 0	OHC	Mürdter 2016 [76]
500 mg b.i.d po	42 mg s.d. po	168	0.76	0.65	1.17	0.61	0.54	1.14	AS = 0	NDC	Mürdter 2016 [76]
500 mg b.i.d po	42 mg s.d. po	168	1.62	0.59	2.75	1.74	0.67	2.59	AS = 0	HDC	Mürdter 2016 [76]
500 mg b.i.d po	42 mg s.d. po	168	1.46	1.82	0.80	1.18	1.59	0.75	AS = 0.5	CLO	Mürdter 2016 [76]
500 mg b.i.d po	42 mg s.d. po	168	1.75	1.72	1.02	1.41	1.40	1.00	AS = 0.5	OHC	Mürdter 2016 [76]
500 mg b.i.d po	42 mg s.d. po	168	0.30	0.29	1.02	0.17	0.19	0.92	AS = 0.5	NDC	Mürdter 2016 [76]
500 mg b.i.d po	42 mg s.d. po	168	0.45	0.35	1.28	0.25	0.25	0.97	AS = 0.5	HDC	Mürdter 2016 [76]
500 mg b.i.d po	42 mg s.d. po	72	1.22	1.98	0.62	1.10	1.73	0.63	AS = 1	CLO	Mürdter 2016 [76]
500 mg b.i.d po	42 mg s.d. po	168	1.40	1.63	0.86	1.18	1.67	0.70	AS = 1	OHC	Mürdter 2016 [76]
500 mg b.i.d po	42 mg s.d. po	72	0.19	0.34	0.57	0.19	0.47	0.41	AS = 1	NDC	Mürdter 2016 [76]
500 mg b.i.d po	42 mg s.d. po	168	0.38	0.43	0.89	0.21	0.39	0.55	AS = 1	HDC	Mürdter 2016 [76]
500 mg b.i.d po	42 mg s.d. po	72	1.15	1.23	0.94	1.07	1.59	0.67	AS = 2	CLO	Mürdter 2016 [76]
500 mg b.i.d po	42 mg s.d. po	168	1.29	1.22	1.06	1.13	1.04	1.09	AS = 2	OHC	Mürdter 2016 [76]
500 mg b.i.d po	42 mg s.d. po	72	0.29	0.21	1.38	0.26	0.19	1.36	AS = 2	NDC	Mürdter 2016 [76]
500 mg b.i.d po	42 mg s.d. po	72	0.27	0.22	1.25	0.25	0.29	0.87	AS = 2	HDC	Mürdter 2016 [76]
500 mg b.i.d po	42 mg s.d. po	72	1.34	1.06	0.83	1.06	1.80	0.59	AS = 3	CLO	Mürdter 2016 [76]
500 mg b.i.d po	42 mg s.d. po	168	1.21	1.83	0.66	1.09	1.20	0.91	AS = 3	OHC	Mürdter 2016 [76]
500 mg b.i.d po	42 mg s.d. po	72	0.34	0.39	0.86	0.31	0.23	1.34	AS = 3	NDC	Mürdter 2016 [76]
500 mg b.i.d po	42 mg s.d. po	72	0.29	0.41	0.72	0.29	0.38	0.77	AS = 3	HDC	Mürdter 2016 [76]
**Mean GMFE (range):**			**1.43 (1.08–2.34), 19/20 with GMFE $\leq$ 2**			**1.54 (1.06–2.55), 18/20 with GMFE $\leq$ 2**					

AUC_{last}: area under the plasma concentration-time curve calculated between the first and last concentration measurement, AS: CYP2D6 activity score, b.i.d.: twice daily, CLO: (*E*)-clomiphene, C_{max}: maximum plasma concentration, DDI: drug-drug interaction, GMFE: geometric mean fold error, HDC: (*E*)-4-hydroxy-N-desethylclomiphene, NDC: (*E*)-N-desethylclomiphene, obs: observed, OHC: (*E*)-4-hydroxyclomiphene, po: oral, pred: predicted, q.d.: once daily, s.d: single dose, t_{last}: time of the last concentration measurement. If perpetrator or victim drugs were applied in form of salts, the respective dose of base was calculated and incorporated in simulations.

Table S19: Predicted and observed DDI AUC_{last} and C_{max} ratios involving ***(E)*-clomiphene** as victim drug *(continued)*

Drug administration			DDI AUC_{last} ratio			DDI C_{max} ratio					
**Perpetrator**	**(E)-Clomiphene**	**t_{last} [h]**	**Pred**	**Obs**	**Pred/Obs**	**Pred**	**Obs**	**Pred/Obs**	**AS**	**Molecule**	**Reference**
Paroxetine											
40 mg q.d. po	42 mg s.d. po	168	1.13	1.09	1.03	1.06	0.91	1.17	AS = 0	CLO	Mürdter 2016 [76]
40 mg q.d. po	42 mg s.d. po	168	1.15	1.02	1.13	1.13	1.19	0.95	AS = 0	OHC	Mürdter 2016 [76]
40 mg q.d. po	42 mg s.d. po	168	1.00	1.21	0.83	0.94	1.36	0.70	AS = 0	NDC	Mürdter 2016 [76]
40 mg q.d. po	42 mg s.d. po	168	1.13	1.01	1.12	1.13	0.94	1.21	AS = 0	HDC	Mürdter 2016 [76]
40 mg q.d. po	42 mg s.d. po	168	2.47	2.47	1.00	1.39	1.30	1.07	AS = 0.5	CLO	Mürdter 2016 [76]
40 mg q.d. po	42 mg s.d. po	168	0.42	0.22	1.95	0.24	0.09	2.62	AS = 0.5	OHC	Mürdter 2016 [76]
40 mg q.d. po	42 mg s.d. po	168	4.17	4.86	0.86	1.86	1.29	1.44	AS = 0.5	NDC	Mürdter 2016 [76]
40 mg q.d. po	42 mg s.d. po	168	0.38	0.29	1.32	0.24	0.22	1.10	AS = 0.5	HDC	Mürdter 2016 [76]
40 mg q.d. po	42 mg s.d. po	168	5.08	9.53	0.53	1.76	4.02	0.44	AS = 1	CLO	Mürdter 2016 [76]
40 mg q.d. po	42 mg s.d. po	168	0.50	0.86	0.58	0.17	0.28	0.60	AS = 1	OHC	Mürdter 2016 [76]
40 mg q.d. po	42 mg s.d. po	168	12.21	25.32	0.48	3.40	9.07	0.37	AS = 1	NDC	Mürdter 2016 [76]
40 mg q.d. po	42 mg s.d. po	168	1.23	1.91	0.64	0.46	0.50	0.92	AS = 1	HDC	Mürdter 2016 [76]
40 mg q.d. po	42 mg s.d. po	168	7.19	10.35	0.69	2.13	3.85	0.55	AS = 2	CLO	Mürdter 2016 [76]
40 mg q.d. po	42 mg s.d. po	168	0.84	1.54	0.55	0.19	0.27	0.71	AS = 2	OHC	Mürdter 2016 [76]
40 mg q.d. po	42 mg s.d. po	168	26.72	21.14	1.26	5.70	4.59	1.24	AS = 2	NDC	Mürdter 2016 [76]
40 mg q.d. po	42 mg s.d. po	168	3.58	2.65	1.35	0.71	0.74	0.96	AS = 2	HDC	Mürdter 2016 [76]
40 mg q.d. po	42 mg s.d. po	168	7.29	12.47	0.59	2.08	7.08	0.29	AS = 3	CLO	Mürdter 2016 [76]
40 mg q.d. po	42 mg s.d. po	168	1.03	3.76	0.27	0.24	0.70	0.34	AS = 3	OHC	Mürdter 2016 [76]
40 mg q.d. po	42 mg s.d. po	168	21.91	33.72	0.65	5.28	12.14	0.43	AS = 3	NDC	Mürdter 2016 [76]
40 mg q.d. po	42 mg s.d. po	168	4.53	5.87	0.77	1.08	1.54	0.70	AS = 3	HDC	Mürdter 2016 [76]
**Mean GMFE (range):**			**1.56 (1.00–3.64), 18/20 with GMFE $\leq$ 2**			**1.72 (1.04–3.41), 15/20 with GMFE $\leq$ 2**					
**Overall GMFE (range):**			**1.49 (1.00–3.64), 37/40 with GMFE $\leq$ 2**			**1.63 (1.04–3.41), 33/40 with GMFE $\leq$ 2**					

AUC_{last}: area under the plasma concentration-time curve calculated between the first and last concentration measurement, AS: CYP2D6 activity score, b.i.d.: twice daily, CLO: (*E*)-clomiphene, C_{max}: maximum plasma concentration, DDI: drug-drug interaction, GMFE: geometric mean fold error, HDC: (*E*)-4-hydroxy-N-desethylclomiphene, NDC: (*E*)-N-desethylclomiphene, obs: observed, OHC: (*E*)-4-hydroxyclomiphene, po: oral, pred: predicted, q.d.: once daily, s.d: single dose, t_{last}: time of the last concentration measurement. If perpetrator or victim drugs were applied in form of salts, the respective dose of base was calculated and incorporated in simulations.

S6.4 Desipramine

S6.4.1 Clinical Studies

Table S20: Clinical study data used for DD(G)I model development with **desipramine** as victim

Drug administration										
**Perpetrator**	**Desipramine**	**n**	**Population**[a]	**Fem. [%]**	**Age [years]**	**Weight [kg]**	**BMI [kg/m²]**	**Phenotype**	**Molecule**	**Reference**
Atomoxetine										
40/60 mg b.i.d. po	50 mg s.d. po	22	American [7]	50	(26–55)	-	(18.8–30.4)	-	DES	Sauer 2004 [17]
Bupropion										
150 mg q.d./b.i.d. po	50 mg s.d. po	15	American [7]	0	-	-	-	-	DES	Reese 2008 [16]
Paroxetine										
20 mg q.d. po	50 mg s.d. po	20	American [7]	15	35.0±7.7	79.9±9.8	-	-	DES, OHD	Nichols 2009 [13]
20 mg q.d. po	100 mg s.d. po	8	European [3]	0	(22–24)	-	-	PM	DES	Brøsen 1993 [22]
20 mg q.d. po	100 mg s.d. po	5	European [3]	0	(22–24)	-	-	NM	DES	Brøsen 1993 [22]
20 mg q.d. po	100 mg s.d. po	4	European [3]	0	(22–24)	-	-	fast NM	DES	Brøsen 1993 [22]
20/30 mg q.d. po	50 mg q.d. po	6	American [7]	0	30.4±5.2	73.3±8.5	-	-	DES	Alderman 1997 [19]
Quinidine										
200 mg q.d. po	100 mg s.d. po	6	European [3]	50	27.7 (23–38)	-	-	-	DES, OHD, QUI, OHQ	Brøsen 1989 [23]

b.i.d.: twice daily, BMI: body mass index, DD(G)I: drug-drug(-gene) interaction, DES: desipramine, fem: females, n: number of study participants, NM: CYP2D6 normal metabolizer, OHD: 2-hydroxydesipramine, OHQ: 3-hydroxyquinidine, PM: CYP2D6 poor metabolizer, po: oral, q.d.: once daily, QUI: quinidine, s.d: single dose, -: not available. Values are given as mean (range). If perpetrator or victim drugs were applied in form of salts, the respective dose of base was calculated and incorporated in simulations. [a] Population used in simulations.

S6.4.2 Plasma Concentration-Time Profiles (Semilogarithmic Representation)

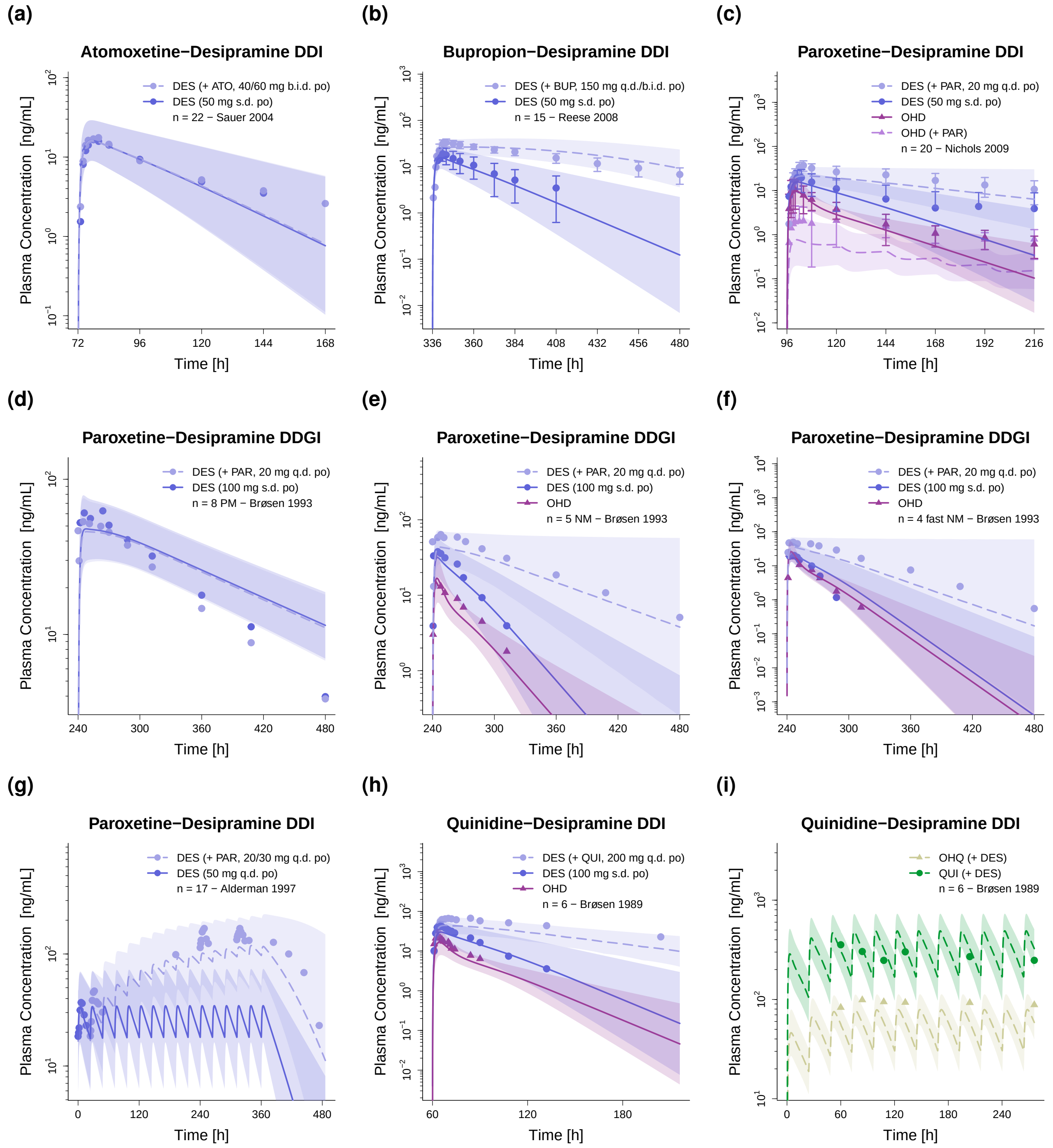

Figure S37: Predicted compared to observed plasma concentration-time profiles of desipramine alone (solid line) and after pretreatment and/or concomitant administration (dashed line) of (a) atomoxetine, (b) bupropion, (c–g) paroxetine and (h) quinidine (semilogarithmic representation). Population predicted (1000 individuals) geometric means are shown as lines, corresponding geometric standard deviations as shaded areas and observed data as dots/triangles (± standard deviation, if reported) [13, 16, 17, 19, 22, 23]. ATO: atomoxetine, b.i.d.: twice daily, BUP: bupropion, DD(G)I: drug-drug(-gene) interaction, DES: desipramine, n: number of study participants, NM: CYP2D6 normal metabolizer, OHD: 2-hydroxydesipramine, OHQ: 3-hydroxydesipramine, PAR: paroxetine, PM: CYP2D6 poor metabolizer, po: oral, q.d.: once daily, QUI: quinidine, s.d.: single dose.

S6.4.3 Plasma Concentration-Time Profiles (Linear Representation)

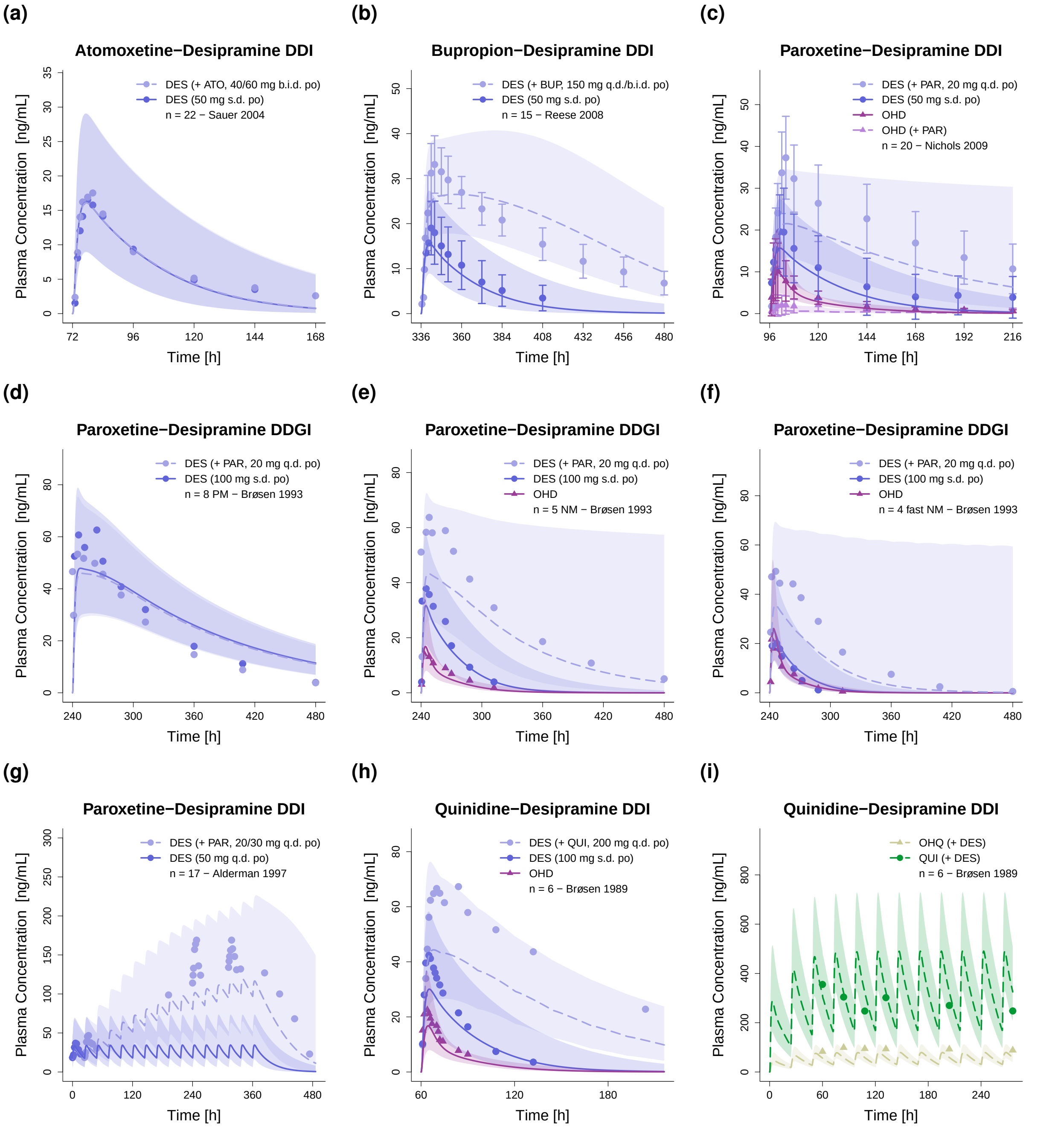

Figure S38: Predicted compared to observed plasma concentration-time profiles of desipramine alone (solid line) and after pretreatment and/or concomitant administration (dashed line) of (a) atomoxetine, (b) bupropion, (c–g) paroxetine and (h) quinidine (linear representation). Population predicted (1000 individuals) geometric means are shown as lines, corresponding geometric standard deviations as shaded areas and observed data as dots/triangles (± standard deviation, if reported) [13, 16, 17, 19, 22, 23]. ATO: atomoxetine, b.i.d.: twice daily, BUP: bupropion, DD(G)I: drug-drug(-gene) interaction, DES: desipramine, n: number of study participants, NM: CYP2D6 normal metabolizer, OHD: 2-hydroxydesipramine, OHQ: 3-hydroxydesipramine, PAR: paroxetine, PM: CYP2D6 poor metabolizer, po: oral, q.d.: once daily, QUI: quinidine, s.d.: single dose.

S6.4.4 DDI AUC_{last} and C_{max} Ratios

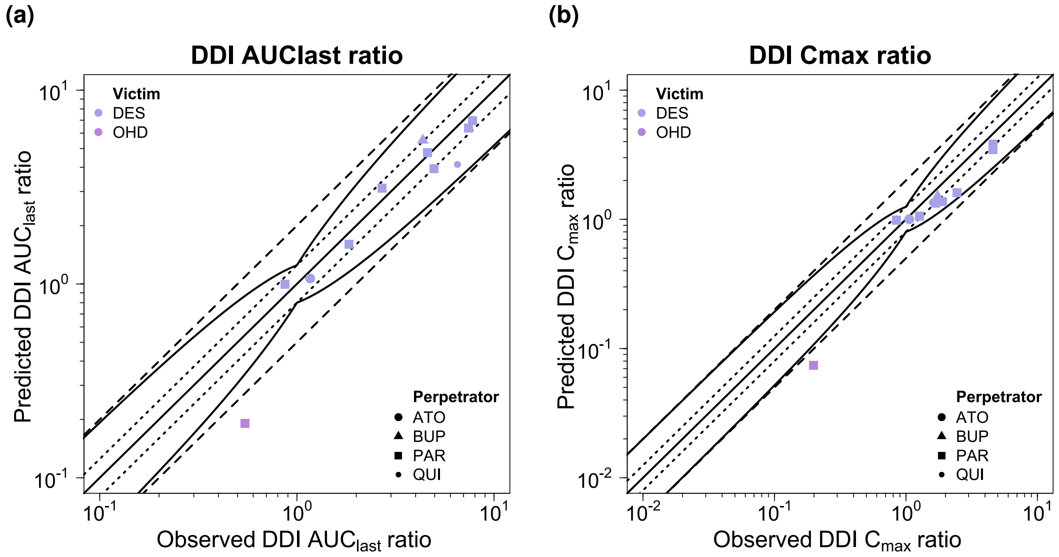

Figure S39: Goodness-of-fit plots comparing predicted and observed DDI AUC_{last} and C_{max} ratios for victim drug desipramine. The solid line marks the line of identity. Dotted lines indicate 1.25-fold, dashed lines indicate 2-fold deviation. Prediction success limits proposed by Guest et al. [2] are shown as curved lines (including 20% variability). ATO: atomoxetine, BUP: bupropion, AUC_{last}: area under the plasma concentration-time curve calculated between the first and last concentration measurement, C_{max}: maximum plasma concentration, DES: desipramine, DDI: drug-drug interaction, OHD: 2-hydroxydesipramine, PAR: paroxetine, QUI: quinidine.

S6.4.5 Geometric Mean Fold Errors of Predicted DDI AUC_{last} and C_{max} Ratios

Table S21: Predicted and observed DDI AUC_{last} and C_{max} ratios involving **desipramine** as victim drug

**Drug administration**			**DDI AUC_{last} ratio**			**DDI C_{max} ratio**					
**Perpetrator**	**Desipramine**	**t_{last} [h]**	**Pred**	**Obs**	**Pred/Obs**	**Pred**	**Obs**	**Pred/Obs**	**Phenotype**	**Molecule**	**Reference**
Atomoxetine											
40/60 mg b.i.d. po	50 mg s.d. po	96	1.07	1.17	0.91	1.00	1.06	0.95	-	DES	Sauer 2004 [17]
**Mean GMFE (range):**			**1.09 (-), 1/1 with GMFE ≤ 2**			**1.06 (-), 1/1 with GMFE ≤ 2**					
Bupropion											
150 mg q.d./b.i.d. po	50 mg s.d. po	168	5.52	4.35	1.27	1.51	1.74	0.87	-	DES	Reese 2008 [16]
**Mean GMFE (range):**			**1.27 (-), 1/1 with GMFE ≤ 2**			**1.15 (-), 1/1 with GMFE ≤ 2**					
Paroxetine											
20 mg q.d. po	50 mg s.d. po	120	3.13	2.71	1.16	1.37	1.90	0.72	-	DES	Nichols 2009 [13]
20 mg q.d. po	50 mg s.d. po	120	0.19	0.55	0.35	0.07	0.20	0.37	-	OHD	Nichols 2009 [13]
20 mg q.d. po	100 mg s.d. po	239	1.00	0.87	1.15	0.99	0.85	1.16	PM	DES	Brøsen 1993 [22]
20 mg q.d. po	100 mg s.d. po	234	4.76	4.60	1.04	1.33	1.90	0.79	NM	DES	Brøsen 1993 [22]
20 mg q.d. po	100 mg s.d. po	233	6.96	7.76	0.90	1.61	2.45	0.65	fast NM	DES	Brøsen 1993 [22]
20/30 mg q.d. po	50 mg q.d. po (D1)	24	1.60	1.84	0.87	1.06	1.27	0.84	-	DES	Alderman 1997 [19]
20/30 mg q.d. po	50 mg q.d. po (D10)	16	3.93	4.96	0.79	3.48	4.58	0.76	-	DES	Alderman 1997 [19]
20/30 mg q.d. po	50 mg q.d. po (D13)	16	6.37	7.40	0.86	3.83	4.58	0.84	-	DES	Alderman 1997 [19]
**Mean GMFE (range):**			**1.37 (1.04–2.86), 7/8 with GMFE ≤ 2**			**1.47 (1.16–2.68), 7/8 with GMFE ≤ 2**					
Quinidine											
200 mg q.d. po	100 mg s.d. po	213	4.14	6.52	0.63	1.35	1.59	0.85	-	DES	Brøsen 1989 [23]
**Mean GMFE (range):**			**1.58 (-), 1/1 with GMFE ≤ 2**			**1.18 (-), 1/1 with GMFE ≤ 2**					
**Overall GMFE (range):**			**1.35 (1.04–2.86), 10/11 with GMFE ≤ 2**			**1.38 (1.06–2.68), 10/11 with GMFE ≤ 2**					

AUC_{last}: area under the plasma concentration-time curve calculated between the first and last concentration measurement, b.i.d.: twice daily, C_{max}: maximum plasma concentration, D: day, DDI: drug-drug interaction, DES: desipramine, GMFE: geometric mean fold error, NM: CYP2D6 normal metabolizer, obs: observed, OHD: 2-hydroxydesipramine, PM: CYP2D6 poor metabolizer, po: oral, pred: predicted, q.d.: once daily, s.d.: single dose, t_{last}: time of the last concentration measurement. If perpetrator or victim drugs were applied in form of salts, the respective dose of base was calculated and incorporated in simulations.

S6.5 Dextromethorphan

S6.5.1 Clinical Studies

Table S22: Clinical study data used for DD(G)I model development with **dextromethorphan** as victim

Drug administration										
**Perpetrator**	**Dextromethorphan**	**n**	**Population[a]**	**Fem. [%]**	**Age [years]**	**Weight [kg]**	**BMI [kg/m²]**	**Phenotype**	**Molecule**	**Reference**
Paroxetine										
20 mg b.i.d. po	5 mg s.d. po	16	European [3]	75	24 (21–27)	-	22 (19–26)	IM	DEX, DXT, PAR	Storelli 2018 [77]
20 mg b.i.d. po	5 mg s.d. po	17	European [3]	53	27 (18–42)	-	23 (19–26)	NM	DEX, DXT, PAR	Storelli 2018 [77]
30 mg q.d. po	30 mg b.i.d. po[b]	13	American [7]	14.3	33.5 (23–50)	73.3	25.1	NM	DEX	Schoedel 2012 [101]
Quinidine										
50 mg s.d. po	30 mg s.d. po	6	European [3]	33.3	22.4 (20–26)	70 (49–86)	-	NM	DEX, DTT	Capon 1996 [80]
100 mg s.d. po	30 mg s.d. po	5	American [7]	80	26.4 (20–31)	-	-	NM	DEX, DXT, DXG	Schadel 1995 [102]

b.i.d.: twice daily, BMI: body mass index, DD(G)I: drug-drug(-gene) interaction, DEX: dextromethorphan, DTT: total dextrorphan, DXG: dextrorphan-O-glucuronide, DXT: dextrorphan, fem: females, IM: CYP2D6 intermediate metabolizer, n: number of study participants, NM: CYP2D6 normal metabolizer, PAR: paroxetine, po: oral, s.d: single dose, -: not available. Values are given as mean (range). If perpetrator or victim drugs were applied in form of salts, the respective dose of base was calculated and incorporated in simulations. [a] Population used in simulations. [b]: plus quinidine (30 mg b.i.d. po)

S6.5.2 Plasma Concentration-Time Profiles (Semilogarithmic Representation)

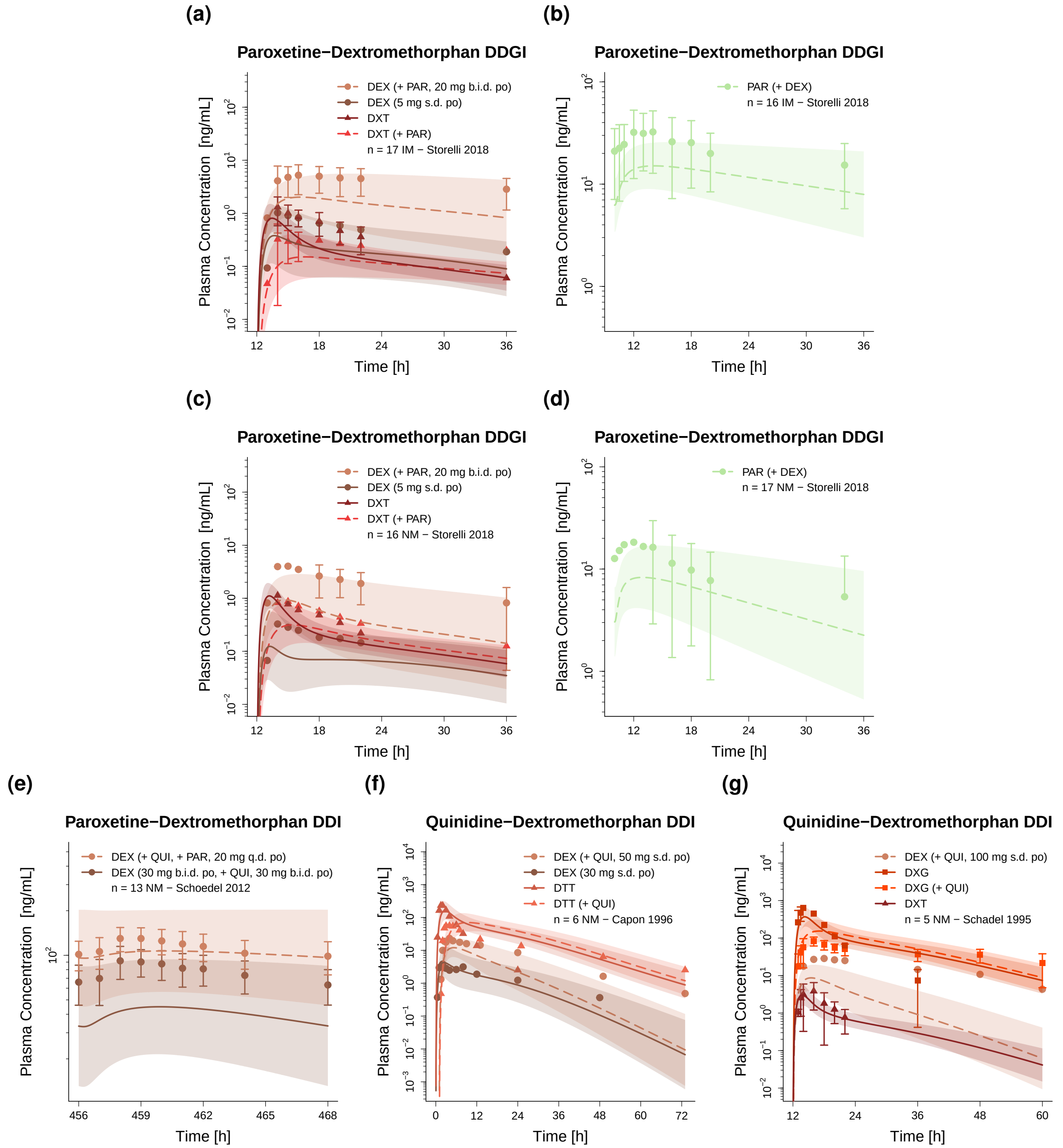

Figure S40: Predicted compared to observed plasma concentration-time profiles of dextromethorphan alone (solid line) and after pretreatment and/or concomitant administration (dashed line) of (a,c,e) paroxetine and (f–g) quinidine (semilogarithmic representation). Population predicted (1000 individuals) geometric means are shown as lines, corresponding geometric standard deviations as shaded areas and observed data as dots/triangles/squares (± standard deviation, if reported) [77, 80, 101, 102]. b.i.d.: twice daily, DD(G)I: drug-drug(-gene) interaction, DEX: dextromethorphan, DTT: total dextrorphan, DXG: dextrorphan-O-glucuronide, DXT: dextrorphan, IM: CYP2D6 intermediate metabolizer, n: number of study participants, NM: CYP2D6 normal metabolizer, PAR: paroxetine, po: oral, q.d.: once daily, QUI: quinidine, s.d.: single dose.

S6.5.3 Plasma Concentration-Time Profiles (Linear Representation)

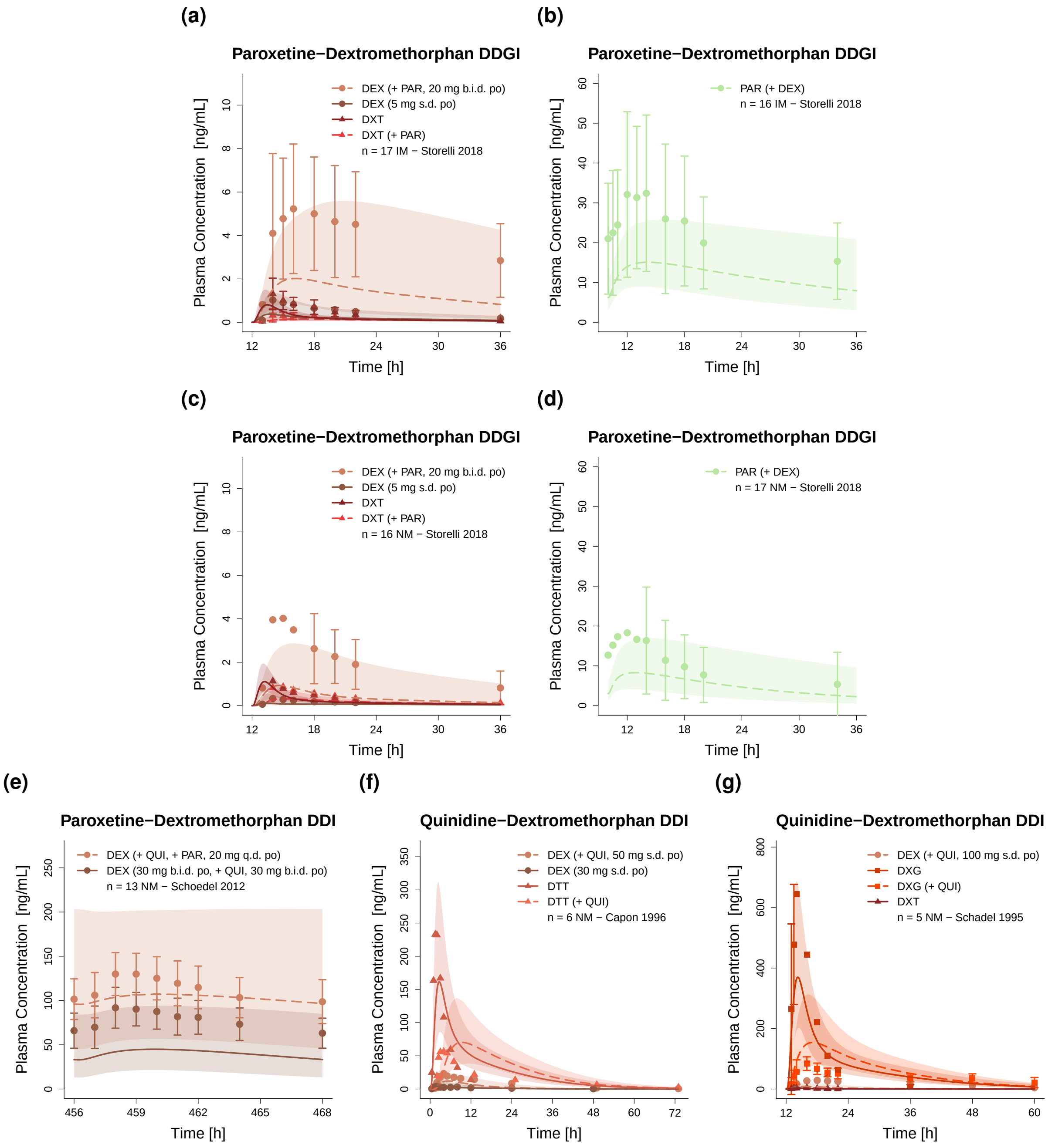

Figure S41: Predicted compared to observed plasma concentration-time profiles of dextromethorphan alone (solid line) and after pretreatment and/or concomitant administration (dashed line) of (a,c,e) paroxetine and (f–g) quinidine (linear representation). Population predicted (1000 individuals) geometric means are shown as lines, corresponding geometric standard deviations as shaded areas and observed data as dots/triangles/squares (± standard deviation, if reported) [77, 80, 101, 102]. b.i.d.: twice daily, DD(G)I: drug-drug(-gene) interaction, DEX: dextromethorphan, DTT: total dextrorphan, DXG: dextrorphan-O-glucuronide, DXT: dextrorphan, IM: CYP2D6 intermediate metabolizer, n: number of study participants, NM: CYP2D6 normal metabolizer, PAR: paroxetine, po: oral, q.d.: once daily, QUI: quinidine, s.d.: single dose.

S6.5.4 DDI AUC_{last} and C_{max} Ratios

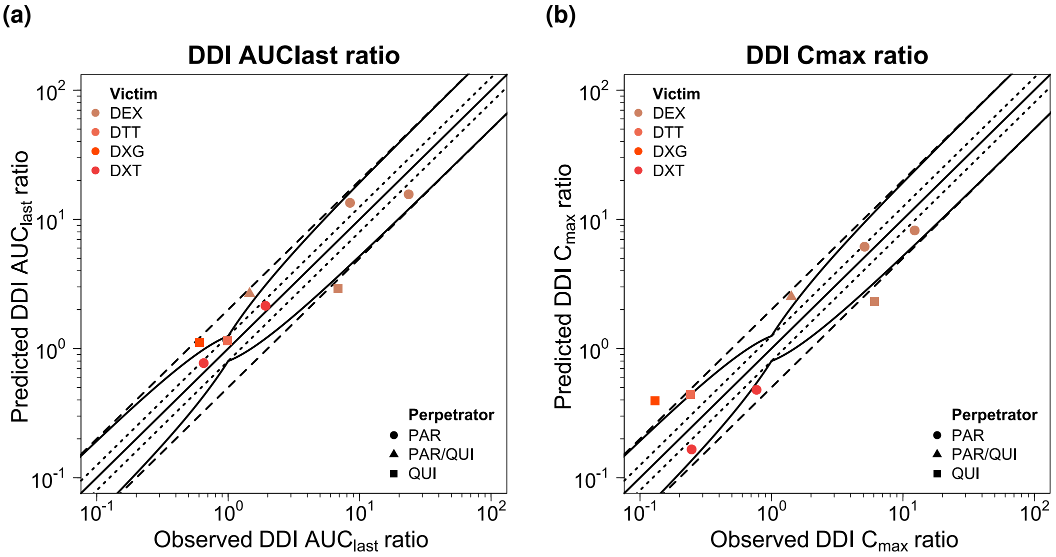

Figure S42: Goodness-of-fit plots comparing predicted and observed DDI AUC_{last} and C_{max} ratios for victim drug dextromethorphan. The solid line marks the line of identity. Dotted lines indicate 1.25-fold, dashed lines indicate 2-fold deviation. Prediction success limits proposed by Guest et al. [2] are shown as curved lines (including 20% variability). AUC_{last}: area under the plasma concentration-time curve calculated between the first and last concentration measurement, C_{max}: maximum plasma concentration, DEX: dextromethorphan, DTT: total dextrorphan, DXG: dextrorphan O-glucuronide, DXT: dextrorphan, PAR: paroxetine, QUI: quinidine.

S6.5.5 Geometric Mean Fold Errors of Predicted DDI AUC_{last} and C_{max} Ratios

Table S23: Predicted and observed DDI AUC_{last} and C_{max} ratios involving **dextromethorphan** as victim drug

**Drug administration**			**DDI AUC_{last} ratio**			**DDI C_{max} ratio**					
**Perpetrator**	**Dextromethorphan**	**t_{last} [h]**	**Pred**	**Obs**	**Pred/Obs**	**Pred**	**Obs**	**Pred/Obs**	**Phenotype**	**Molecule**	**Reference**
Paroxetine											
20 mg b.i.d. po	5 mg s.d. po	24	13.43	8.45	1.59	6.14	5.11	1.20	IM	DEX	Storelli 2018 [77]
20 mg b.i.d. po	5 mg s.d. po	24	0.77	0.65	1.18	0.17	0.25	0.67	IM	DXT	Storelli 2018 [77]
20 mg b.i.d. po	5 mg s.d. po	24	15.63	23.61	0.66	8.21	12.29	0.67	NM	DEX	Storelli 2018 [77]
20 mg b.i.d. po	5 mg s.d. po	24	2.14	1.92	1.11	0.48	0.77	0.62	NM	DXT	Storelli 2018 [77]
20 mg q.d. po	30 mg b.i.d. po[a]	12	2.66	1.45	1.83	2.51	1.42	1.77	NM	DEX	Schoedel 2012 [101]
**Mean GMFE (range):**			**1.52 (1.11–1.88), 5/5 with GMFE $\leq$ 2**			**1.61 (1.20–1.98), 5/5 with GMFE $\leq$ 2**					
Quinidine											
50 mg s.d. po	30 mg s.d. po	72	2.93	6.84	0.43	2.32	6.07	0.38	NM	DEX	Capon 1996 [80]
50 mg s.d. po	30 mg s.d. po	96	1.15	0.99	1.16	0.44	0.24	1.83	NM	DTT	Capon 1996 [80]
100 mg s.d. po	30 mg s.d. po	48	1.12	0.61	1.85	0.39	0.13	3.03	NM	DXG	Schadel 1995 [102]
**Mean GMFE (range):**			**1.78 (1.16–2.33), 2/3 with GMFE $\leq$ 2**			**2.49 (1.83–3.03), 1/3 with GMFE $\leq$ 2**					
**Overall GMFE (range):**			**1.62 (1.11–2.33), 7/8 with GMFE $\leq$ 2**			**1.94 (1.20–3.03), 6/8 with GMFE $\leq$ 2**					

AUC_{last}: area under the plasma concentration-time curve calculated between the first and last concentration measurement, b.i.d.: twice daily, C_{max}: maximum plasma concentration, DDI: drug-drug interaction, DEX: dextromethorphan, DTT: total dextrorphan, DXG: dextrorphan-O-glucuronide, DXT: dextrorphan, GMFE: geometric mean fold error, obs: observed, PAR: paroxetine, po: oral, pred: predicted, q.d.: once daily, s.d.: single dose, t_{last}: time of the last concentration measurement. If perpetrator or victim drugs were applied in form of salts, the respective dose of base was calculated and incorporated in simulations. [a]: plus quinidine (30 mg b.i.d. po)

S6.6 Digoxin

S6.6.1 Clinical Studies

Table S24: Clinical study data used for DDI model development with **digoxin** as victim

Drug administration									
**Perpetrator**	**Digoxin**	**n**	**Population[a]**	**Fem. [%]**	**Age [years]**	**Weight [kg]**	**BMI [kg/m²]**	**Molecule**	**Reference**
Quinidine									
200 mg b.i.d. po	10 µg/kg s.d. iv	6	European [3]	33	(21–28)	-	-	DIG	Steiness 1980 [103]
200 mg q.i.d. po	1 mg s.d. iv	7	European [3]	-	-	-	-	DIG	Ochs 1981 [104]

b.i.d.: twice daily, BMI: body mass index, DDI: drug-drug interaction, DIG: digoxin, fem: females, iv: intravenous, n: number of study participants, po: oral, q.i.d.: four times daily, s.d: single dose, -: not available. Values are given as mean (range). If perpetrator or victim drugs were applied in form of salts, the respective dose of base was calculated and incorporated in simulations. [a] Population used in simulations.

S6.6.2 Plasma Concentration-Time Profiles (Semilogarithmic Representation)

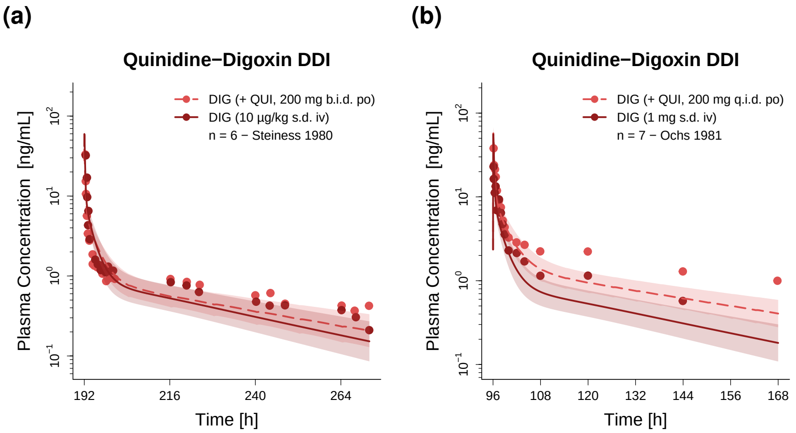

Figure S43: Predicted compared to observed plasma concentration-time profiles of digoxin alone (solid line) and after pretreatment and/or concomitant administration (dashed line) of (a–b) quinidine (semilogarithmic representation). Population predicted (1000 individuals) geometric means are shown as lines, corresponding geometric standard deviations as shaded areas and observed data as dots [103, 104]. b.i.d.: twice daily, DDI: drug-drug interaction, DIG: digoxin, iv: intravenous, n: number of study participants, po: oral, q.i.d.: four times daily, QUI: quinidine, s.d.: single dose.

S6.6.3 Plasma Concentration-Time Profiles (Linear Representation)

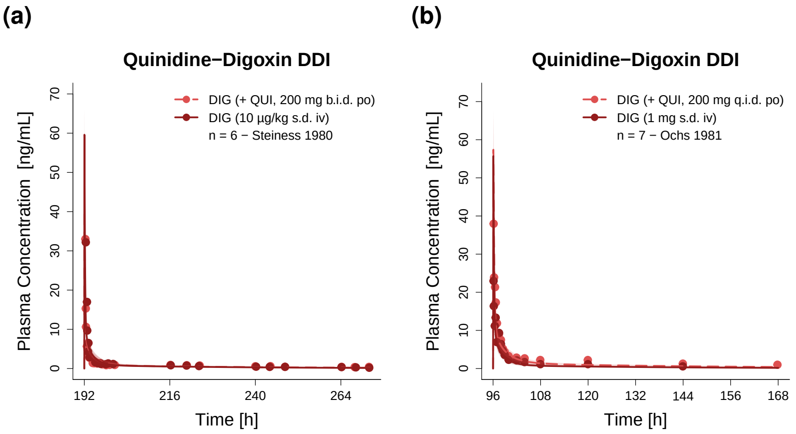

Figure S44: Predicted compared to observed plasma concentration-time profiles of digoxin alone (solid line) and after pretreatment and/or concomitant administration (dashed line) of (a–b) quinidine (linear representation). Population predicted (1000 individuals) geometric means are shown as lines, corresponding geometric standard deviations as shaded areas and observed data as dots [103, 104]. b.i.d.: twice daily, DDI: drug-drug interaction, DIG: digoxin, iv: intravenous, n: number of study participants, po: oral, q.i.d.: four times daily, QUI: quinidine, s.d.: single dose.

S6.6.4 DDI AUC_{last} and C_{max} Ratios

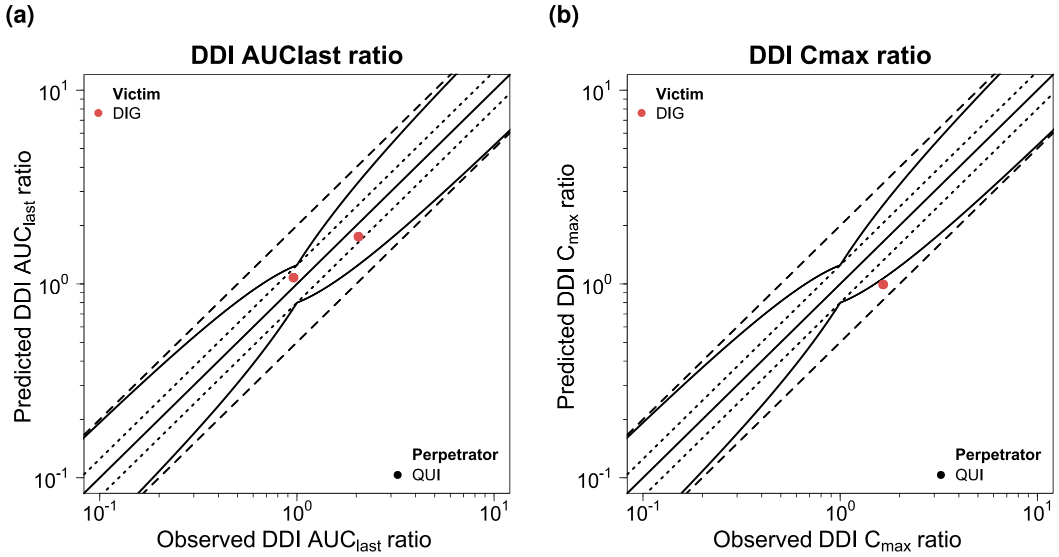

Figure S45: Goodness-of-fit plots comparing predicted and observed DDI AUC_{last} and C_{max} ratios for victim drug digoxin. The solid line marks the line of identity. Dotted lines indicate 1.25-fold, dashed lines indicate 2-fold deviation. Prediction success limits proposed by Guest et al. [2] are shown as curved lines (including 20% variability). AUC_{last}: area under the plasma concentration-time curve calculated between the first and last concentration measurement, C_{max}: maximum plasma concentration, DIG: digoxin, QUI: quinidine.

S6.6.5 Geometric Mean Fold Errors of Predicted DDI AUC_{last} and C_{max} Ratios

Table S25: Predicted and observed DDI AUC_{last} and C_{max} ratios involving **digoxin** as victim drug

Drug administration			DDI AUC_{last} ratio			DDI C_{max} ratio				
**Perpetrator**	**Digoxin**	**t_{last} [h]**	**Pred**	**Obs**	**Pred/Obs**	**Pred**	**Obs**	**Pred/Obs**	**Molecule**	**Reference**
Quinidine										
200 mg b.i.d. po	10 µg/kg s.d. iv	80	1.08	0.96	1.12	-	-	-	DIG	Steiness 1980 [103]
200 mg q.i.d. po	1 mg s.d. iv	72	1.75	2.05	0.85	0.99	1.65	0.60	DIG	Ochs 1981 [104]
**Mean GMFE (range):**			**1.15 (1.12–1.17), 2/2 with GMFE $\leq$ 2**			**1.66 (-), 1/1 with GMFE $\leq$ 2**				

AUC_{last}: area under the plasma concentration-time curve calculated between the first and last concentration measurement, b.i.d.: twice daily, C_{max}: maximum plasma concentration, DDI: drug-drug interaction, DIG: digoxin, GMFE: geometric mean fold error, obs: observed, po: oral, pred: predicted, q.i.d. four times daily, s.d.: single dose, t_{last}: time of the last concentration measurement, -: not available. If perpetrator or victim drugs were applied in form of salts, the respective dose of base was calculated and incorporated in simulations.

S6.7 Metoprolol

S6.7.1 Clinical Studies

Table S26: Clinical study data used for DD(G)I model development with **metoprolol** as victim

Drug administration										
**Perpetrator**	**Metoprolol**	**n**	**Population[a]**	**Fem. [%]**	**Age [years]**	**Weight [kg]**	**BMI [kg/m²]**	**Phenotype**	**Molecule**	**Reference**
Cimetidine										
800 mg q.d. po	100 mg s.d. po (IR)	12	European [3]	50	21.8 (19–34)	-	-	-	MET	Chellingsworth 1988 [105]
800 mg q.d. po	100 mg b.i.d. po (IR)	12	European [3]	0	23.2 (19–32)	-	-	-	MET	Chellingsworth 1988 [105]
800 mg q.d. po	100 mg b.i.d. po (IR)	12	European [3]	0	25 (20–33)	71 (63–84)	180 (171–191)[b]	-	MET	Toon 1988 [106]
200/400 mg q.i.d. po	100 mg b.i.d. po (IR)	6	European [3]	0	23.6±1.9	68.7±5.1	-	-	MET	Kirch 1982 [107]
Paroxetine										
10 mg b.i.d. po	50 mg s.d. po (IR)	10	American [7]	0	28±10	81.6±9.9	179.0±6.0[b]	NM	MET	Stout 2011 [108]
10 mg b.i.d. po	100 mg s.d. po (ER)	10	American [7]	0	28±10	81.6±9.9	179.0±6.0[b]	NM	MET	Stout 2011 [108]
20 mg q.d. po	100 mg s.d. po (IR)	12	American [7]	27	(21–45)	-	-	NM	RME, SME	Parker 2011 [109]
20 mg q.d. po	100 mg b.i.d. po (IR)	12	American [7]	27	(21–45)	-	-	NM	RME, SME	Parker 2011 [109]
20 mg q.d. po	200 mg s.d. po (ER)	12	American [7]	27	(21–45)	-	-	NM	RME, SME	Parker 2011 [109]
10 mg b.i.d. po	100 mg s.d. po (IR)	8	European [3]	0	(20–29)	-	-	-	RME, SME	Hemeryck 2000 [110]
Quinidine										
50 mg s.d. po	20 mg s.d. iv	3	European [3]	0	(22–34)	(58–80)	-	PM	MET	Leemann 1993 [82]
50 mg s.d. po	20 mg s.d. iv	4	European [3]	0	(22–34)	(58–80)	-	NM	MET	Leemann 1993 [82]
250 mg b.i.d. po	20 mg s.d. iv	3	European [3]	0	(22–34)	(58–80)	-	PM	MET	Leemann 1993 [82]
250 mg b.i.d. po	20 mg s.d. iv	4	European [3]	0	(22–34)	(58–80)	-	NM	MET	Leemann 1993 [82]
100 mg q.d. po	200 mg s.d. po	10	American [7]	0	28.9 (24–40)	85.2	-	NM	RME, SME	Johnson 1996 [111]
100 mg q.d. po	200 mg s.d. po	10	American [7]	0	28.5 (24–36)	82.2	-	NM	RME, SME	Johnson 1996 [111]
Rifampicin										
600 mg q.d. po	100 mg s.d. po	12	European [3]	0	28 (21–35)	71 (62–82)	-	-	MET	Bennett 1982 [112]

b.i.d.: twice daily, BMI: body mass index, DD(G)I: drug-drug(-gene) interaction, ER: extended release formulation, fem: females, IR: immediate release formulation, iv: intravenous, MET: metoprolol, n: number of study participants, NM: CYP2D6 normal metabolizer, PM: CYP2D6 poor metabolizer, po: oral, q.d.: once daily, q.i.d.: four times daily, RME: R-metoprolol, s.d: single dose, SME: S-metoprolol, -: not available. Values are given as mean (range). If perpetrator or victim drugs were applied in form of salts, the respective dose of base was calculated and incorporated in simulations.
[a] Population used in simulations. [b] Height of subjects [cm].

S6.7.2 Plasma Concentration-Time Profiles (Semilogarithmic Representation)

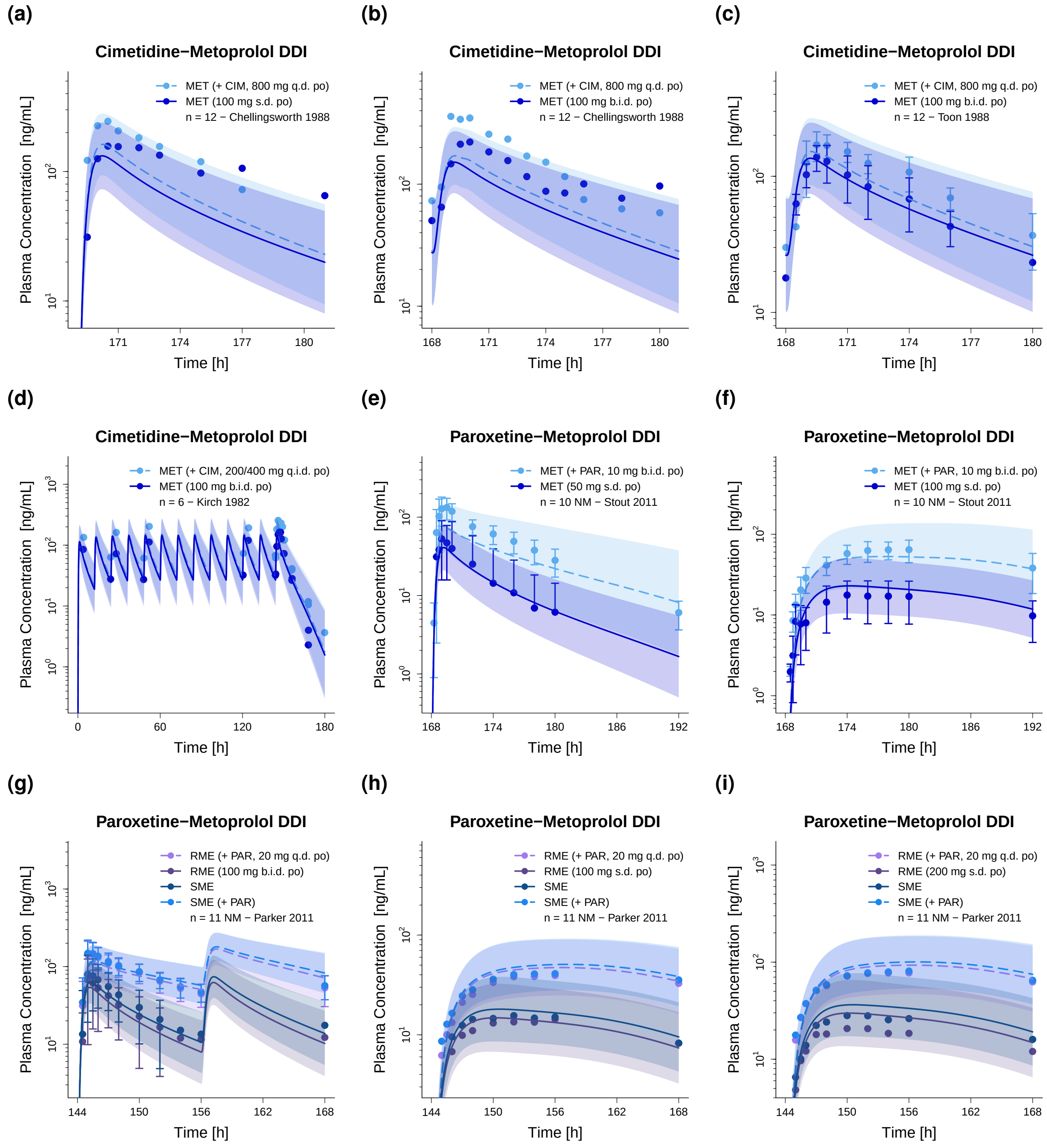

Figure S46: Predicted compared to observed plasma concentration-time profiles of metoprolol alone (solid line) and after pretreatment and/or concomitant administration (dashed line) of (a–d) cimetidine (e–i) paroxetine (semilogarithmic representation). Population predicted (1000 individuals) geometric means are shown as lines, corresponding geometric standard deviations as shaded areas and observed data as dots (± standard deviation, if reported) [105–109]. b.i.d.: twice daily, CIM: cimetidine, DDI: drug-drug interaction, MET: metoprolol, n: number of study participants, NM: CYP2D6 normal metabolizer, PAR: paroxetine, po: oral, q.d.: once daily, RME: R-metoprolol, s.d.: single dose, SME: S-metoprolol.

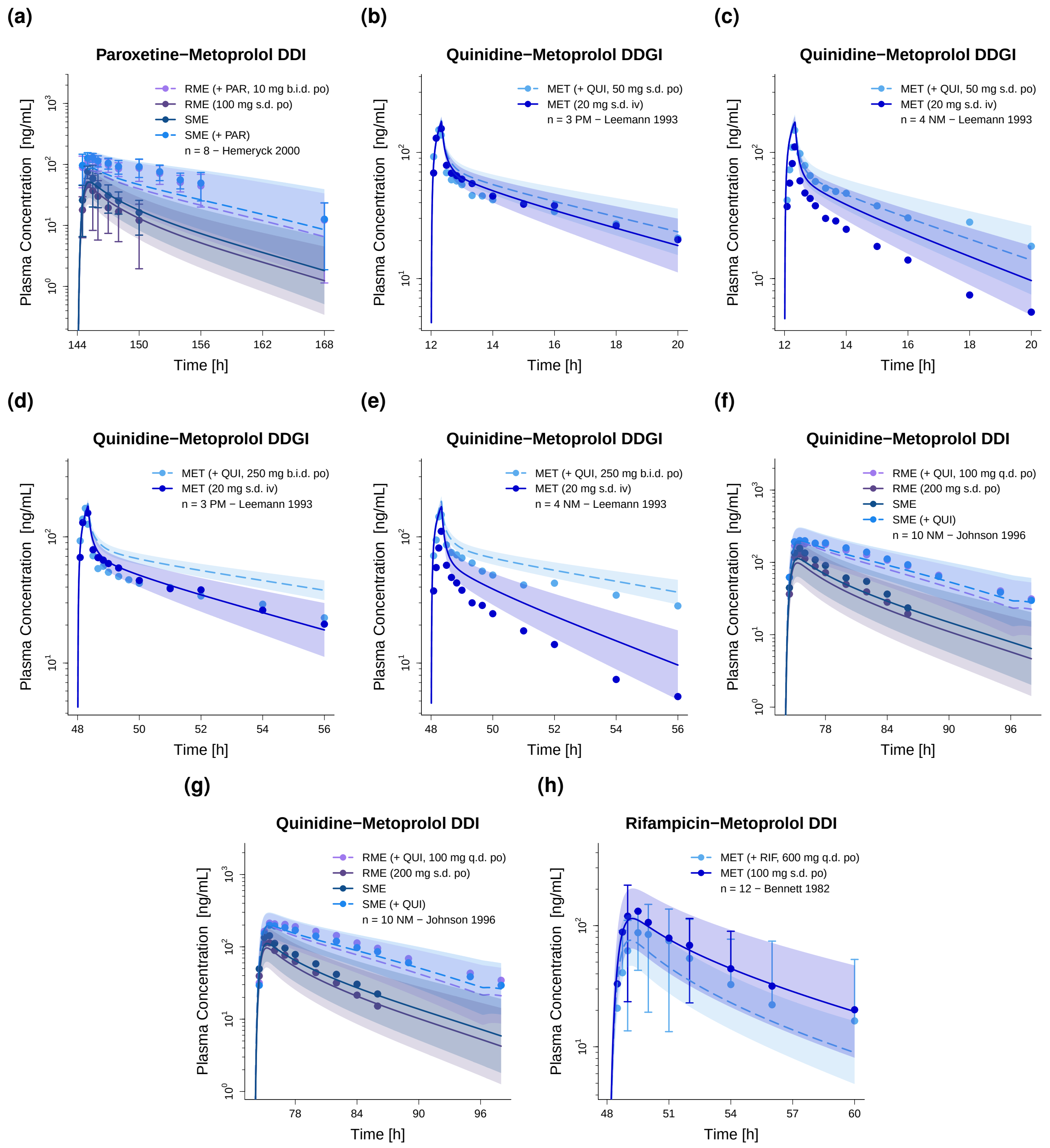

Figure S47: Predicted compared to observed plasma concentration-time profiles of metoprolol alone (solid line) and after pretreatment and/or concomitant administration (dashed line) of (a) paroxetine, (b–g) quinidine and (h) rifampicin (semilogarithmic representation). Population predicted (1000 individuals) geometric means are shown as lines, corresponding geometric standard deviations as shaded areas and observed data as dots (± standard deviation, if reported) [82, 110–112]. b.i.d.: twice daily, DD(G)I: drug-drug(-gene) interaction, iv: intravenous, MET: metoprolol, n: number of study participants, NM: CYP2D6 normal metabolizer, PAR: paroxetine, po: oral, q.d.: once daily, QUI: quinidine, RIF: rifampicin, RME: R-metoprolol, s.d.: single dose, SME: S-metoprolol.

S6.7.3 Plasma Concentration-Time Profiles (Linear Representation)

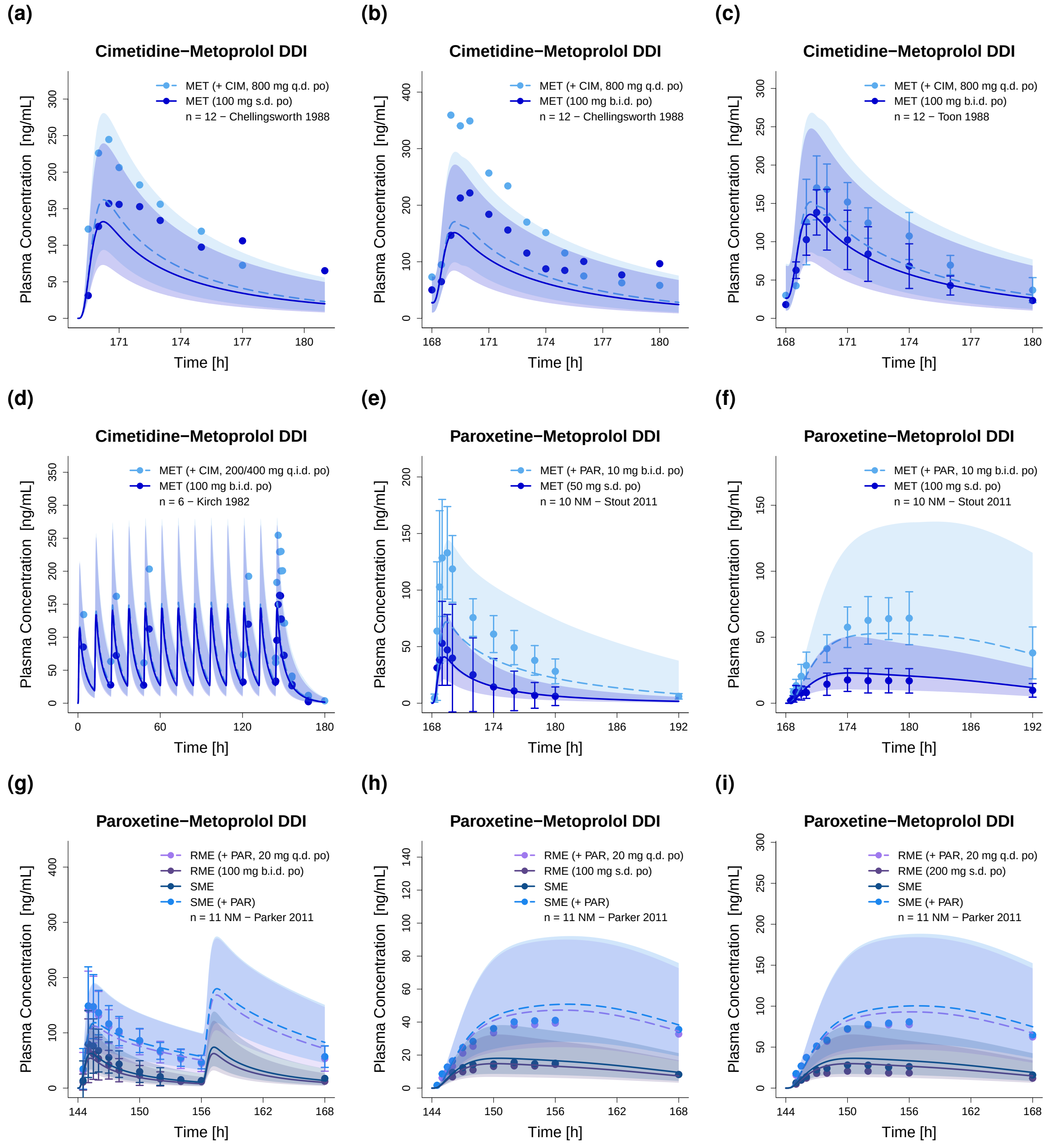

Figure S48: Predicted compared to observed plasma concentration-time profiles of metoprolol alone (solid line) and after pretreatment and/or concomitant administration (dashed line) of (a–d) cimetidine (e–i) paroxetine (linear representation). Population predicted (1000 individuals) geometric means are shown as lines, corresponding geometric standard deviations as shaded areas and observed data as dots (± standard deviation, if reported) [105–109]. b.i.d.: twice daily, CIM: cimetidine, DDI: drug-drug interaction, MET: metoprolol, n: number of study participants, NM: CYP2D6 normal metabolizer, PAR: paroxetine, po: oral, q.d.: once daily, RME: R-metoprolol, s.d.: single dose, SME: S-metoprolol.

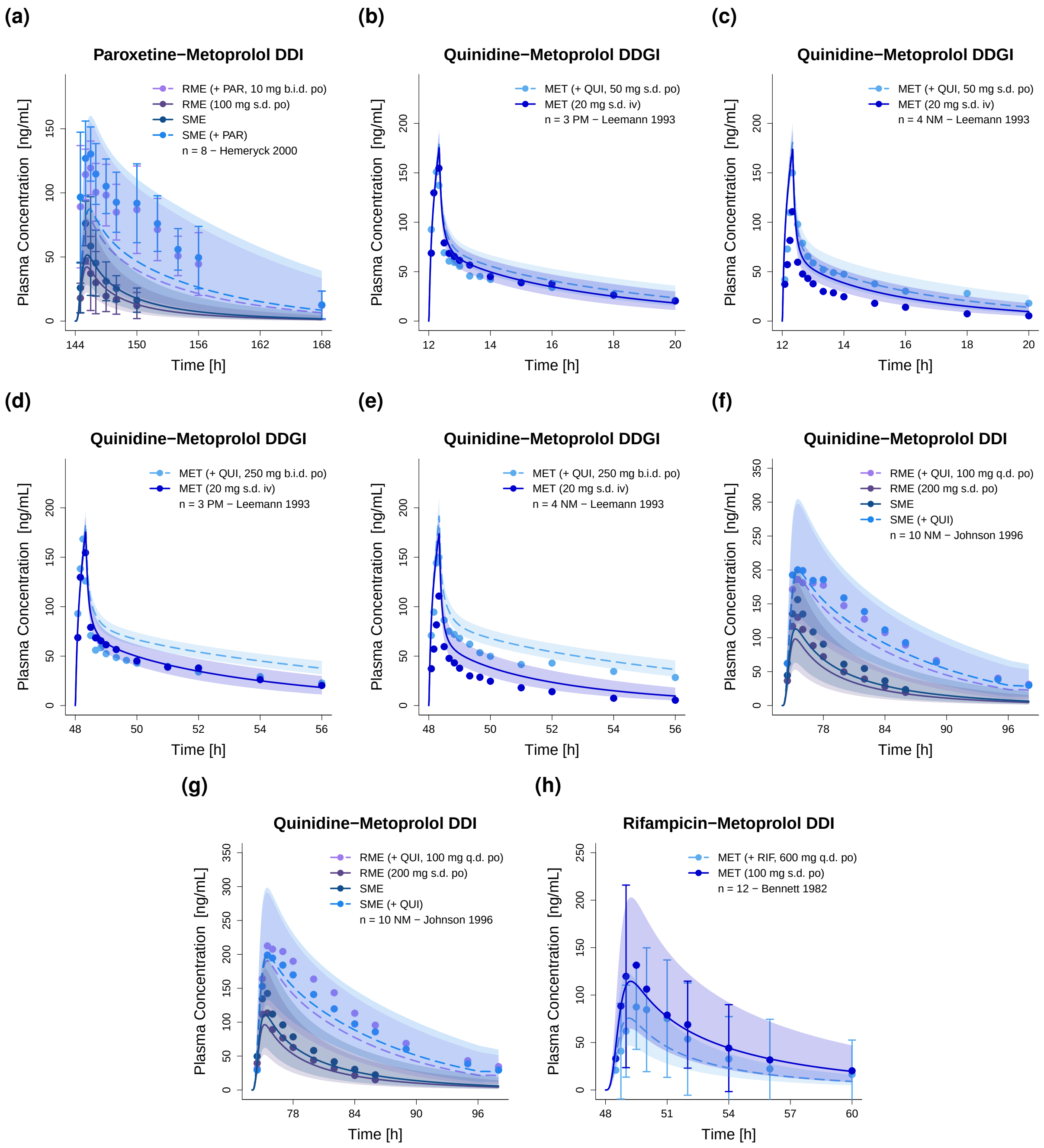

Figure S49: Predicted compared to observed plasma concentration-time profiles of metoprolol alone (solid line) and after pretreatment and/or concomitant administration (dashed line) of (a) paroxetine, (b–g) quinidine and (h) rifampicin (linear representation). Population predicted (1000 individuals) geometric means are shown as lines, corresponding geometric standard deviations as shaded areas and observed data as dots (± standard deviation, if reported) [82, 110–112]. b.i.d.: twice daily, DD(G)I: drug-drug(-gene) interaction, iv: intravenous, MET: metoprolol, n: number of study participants, NM: CYP2D6 normal metabolizer, PAR: paroxetine, po: oral, q.d.: once daily, QUI: quinidine, RIF: rifampicin, RME: R-metoprolol, s.d.: single dose, SME: S-metoprolol.

S6.7.4 DDI AUC_{last} and C_{max} Ratios

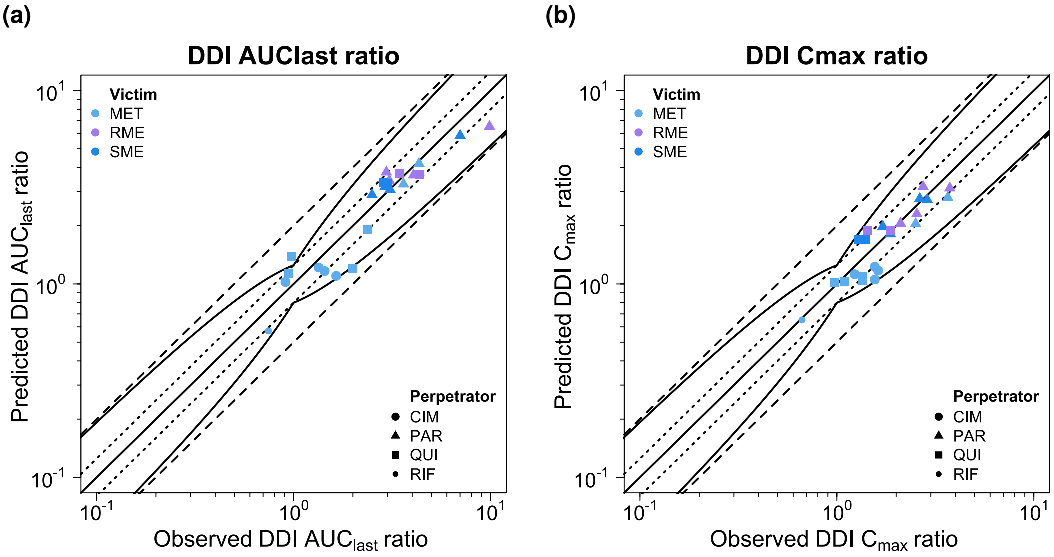

Figure S50: Goodness-of-fit plots comparing predicted and observed DDI AUC_{last} and C_{max} ratios for victim drug metoprolol. The solid line marks the line of identity. Dotted lines indicate 1.25-fold, dashed lines indicate 2-fold deviation. Prediction success limits proposed by Guest et al. [2] are shown as curved lines (including 20% variability). AUC_{last}: area under the plasma concentration-time curve calculated between the first and last concentration measurement, CIM: cimetidine, C_{max}: maximum plasma concentration, MET: metoprolol racemate, RME: (*R*-metoprolol, SME: (*S*-metoprolol, PAR: paroxetine, QUI: quinidine.

S6.7.5 Geometric Mean Fold Errors of Predicted DDI AUC_{last} and C_{max} Ratios

Table S27: Predicted and observed DDI AUC_{last} and C_{max} ratios involving **metoprolol** as victim drug

Drug administration			DDI AUC_{last} ratio			DDI C_{max} ratio					
**Perpetrator**	**Metoprolol**	**t_{last} [h]**	**Pred**	**Obs**	**Pred/Obs**	**Pred**	**Obs**	**Pred/Obs**	**Phenotype**	**Molecule**	**Reference**
Cimetidine											
800 mg q.d. po	100 mg s.d. po	12	1.02	0.91	1.13	1.23	1.56	0.79		MET	Chellingsworth 1988 [105]
800 mg q.d. po	100 mg b.i.d. po	12	1.22	1.34	0.91	1.17	1.62	0.72		MET	Chellingsworth 1988 [105]
800 mg q.d. po	100 mg b.i.d. po	12	1.16	1.44	0.81	1.12	1.23	0.91	-	MET	Toon 1988 [106]
200/400 mg q.i.d. po	100 mg b.i.d. po	36	1.10	1.64	0.67	1.05	1.56	0.68	-	MET	Kirch 1982 [107]
**Mean GMFE (range):**			**1.24 (1.10–1.49), 4/4 with GMFE $\leq$ 2**			**1.31 (1.10–1.48), 4/4 with GMFE $\leq$ 2**					
Paroxetine											
10 mg b.i.d. po	50 mg s.d. po	24	4.20	4.33	0.97	2.05	2.51	0.81	NM	MET	Stout 2011 [108]
10 mg b.i.d. po	100 mg s.d. po	24	3.28	3.62	0.91	2.79	3.65	0.76	NM	MET	Stout 2011 [108]
20 mg q.d. po	100 mg s.d. po	24	3.79	2.96	1.28	3.18	2.74	1.16	NM	RME	Parker 2011 [109]
20 mg q.d. po	100 mg s.d. po	24	3.18	2.91	1.09	2.76	2.64	1.05	NM	SME	Parker 2011 [109]
20 mg q.d. po	100 mg b.i.d. po	12	3.43	3.06	1.12	2.06	2.11	0.98	NM	RME	Parker 2011 [109]
20 mg q.d. po	100 mg b.i.d. po	12	2.89	2.50	1.16	1.81	1.87	0.97	NM	SME	Parker 2011 [109]
20 mg q.d. po	200 mg s.d. po	24	3.65	4.06	0.90	3.13	3.74	0.84	NM	RME	Parker 2011 [109]
20 mg q.d. po	200 mg s.d. po	24	3.08	3.09	1.00	2.72	2.88	0.95	NM	SME	Parker 2011 [109]
10 mg b.i.d. po	100 mg s.d. po	24	6.52	9.88	0.66	2.30	2.55	0.90	-	RME	Hemeryck 2000 [110]
10 mg b.i.d. po	100 mg s.d. po	24	5.85	7.01	0.83	1.98	1.71	1.16	-	SME	Hemeryck 2000 [110]
**Mean GMFE (range):**			**1.16 (1.00–1.51), 10/10 with GMFE $\leq$ 2**			**1.13 (1.02–1.31), 10/10 with GMFE $\leq$ 2**					
Quinidine											
50 mg s.d. po	20 mg s.d. iv	8	1.13	0.95	1.19	1.02	0.98	1.04	PM	MET	Leemann 1993 [82]
50 mg s.d. po	20 mg s.d. iv	8	1.20	2.00	0.60	1.04	1.35	0.77	NM	MET	Leemann 1993 [82]
250 mg b.i.d. po	20 mg s.d. iv	8	1.39	0.97	1.43	1.04	1.09	0.95	PM	MET	Leemann 1993 [82]
250 mg b.i.d. po	20 mg s.d. iv	8	1.91	2.38	0.80	1.09	1.35	0.81	NM	MET	Leemann 1993 [82]
100 mg q.d. po	200 mg s.d. po	24	3.71	3.44	1.08	1.88	1.43	1.32	NM	RME	Johnson 1996 [111]
100 mg q.d. po	200 mg s.d. po	24	3.33	2.87	1.16	1.69	1.28	1.32	NM	SME	Johnson 1996 [111]
100 mg q.d. po	200 mg s.d. po	24	3.69	4.35	0.85	1.89	1.87	1.01	NM	RME	Johnson 1996 [111]
100 mg q.d. po	200 mg s.d. po	24	3.31	2.99	1.11	1.69	1.40	1.21	NM	SME	Johnson 1996 [111]
**Mean GMFE (range):**			**1.27 (1.08–1.66), 8/8 with GMFE $\leq$ 2**			**1.19 (1.01–1.32), 8/8 with GMFE $\leq$ 2**					
Rifampicin											
600 mg q.d. po	100 mg s.d. po	12	0.57	0.74	0.77	0.65	0.66	0.98	-	MET	Bennett 1982 [112]
**Mean GMFE (range):**			**1.30 (-), 1/1 with GMFE $\leq$ 2**			**1.02 (-), 1/1 with GMFE $\leq$ 2**					
**Overall GMFE (range):**			**1.22 (1.00–1.66), 23/23 with GMFE $\leq$ 2**			**1.18 (1.01–1.48), 23/23 with GMFE $\leq$ 2**					

AUC_{last}: area under the plasma concentration-time curve calculated between the first and last concentration measurement, b.i.d.: twice daily, C_{max}: maximum plasma concentration, DDI: drug-drug interaction, GMFE: geometric mean fold error, iv: intravenous, MET: metoprolol, NM: CYP2D6 normal metabolizer, obs: observed, PM: CYP2D6 poor metabolizer, po: oral, pred: predicted, q.d.: once daily, RME: R-metoprolol, s.d.: single dose, SME: S-metoprolol, t_{last}: time of the last concentration measurement, -: not available. If perpetrator or victim drugs were applied in form of salts, the respective dose of base was calculated and incorporated in simulations.

S6.8 Mexiletine

S6.8.1 Clinical Studies

Table S28: Clinical study data used for DD(G)I model development with **mexiletine** as victim

Drug administration										
Perpetrator	Mexiletine	n	Population[a]	Fem. [%]	Age [years]	Weight [kg]	BMI [kg/m²]	Phenotype	Molecule	Reference
Quinidine										
50 mg q.i.d. po	200 mg s.d. po	4	American [7]	7	26	74	-	PM	MEX	Abolfathi 1993 [90]
50 mg q.i.d. po	200 mg s.d. po	10	American [7]	7	26	74	-	NM	MEX	Abolfathi 1993 [90]

BMI: body mass index, DD(G)I: drug-drug(-gene) interaction, fem: females, n: number of study participants, MEX: mexiletine, NM: CYP2D6 normal metabolizer, PM: CYP2D6 poor metabolizer, po: oral, q.i.d.: four times daily, s.d: single dose, -: not available. Values are given as mean (range). If perpetrator or victim drugs were applied in form of salts, the respective dose of base was calculated and incorporated in simulations. [a] Population used in simulations.

S6.8.2 Plasma Concentration-Time Profiles (Semilogarithmic Representation)

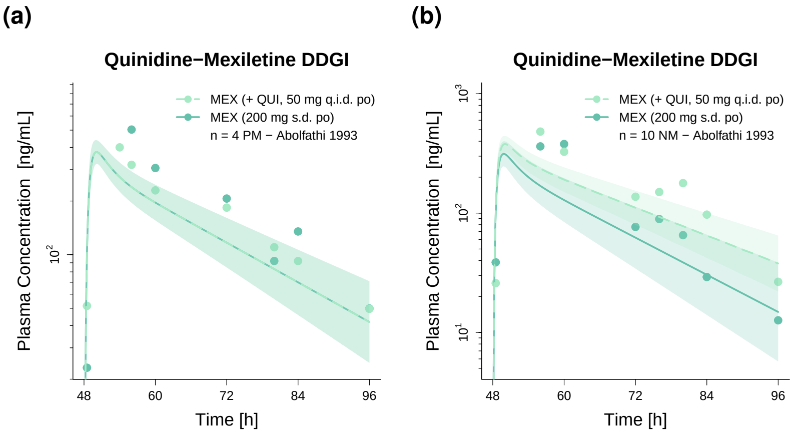

Figure S51: Predicted compared to observed plasma concentration-time profiles of mexiletine alone (solid line) and after pretreatment and/or concomitant administration (dashed line) of quinidine (semilogarithmic representation). Population predicted (1000 individuals) geometric means are shown as lines, corresponding geometric standard deviations as shaded areas and observed data as dots [90]. DDGI: drug-drug-gene interaction, MEX: mexiletine, n: number of study participants, NM: CYP2D6 normal metabolizer, PM: CYP2D6 poor metabolizer, po: oral, q.i.d.: four times daily, QUI: quinidine, s.d.: single dose.

S6.8.3 Plasma Concentration-Time Profiles (Linear Representation)

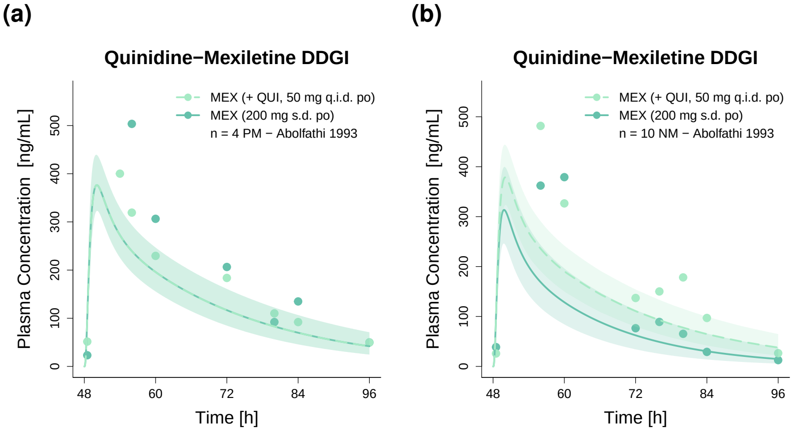

Figure S52: Predicted compared to observed plasma concentration-time profiles of mexiletine alone (solid line) and after pretreatment and/or concomitant administration (dashed line) of quinidine (linear representation). Population predicted (1000 individuals) geometric means are shown as lines, corresponding geometric standard deviations as shaded areas and observed data as dots [90]. DDGI: drug-drug-gene interaction, MEX: mexiletine, n: number of study participants, NM: CYP2D6 normal metabolizer, PM: CYP2D6 poor metabolizer, po: oral, q.i.d.: four times daily, QUI: quinidine, s.d.: single dose.

S6.8.4 DDI AUC_{last} and C_{max} Ratios

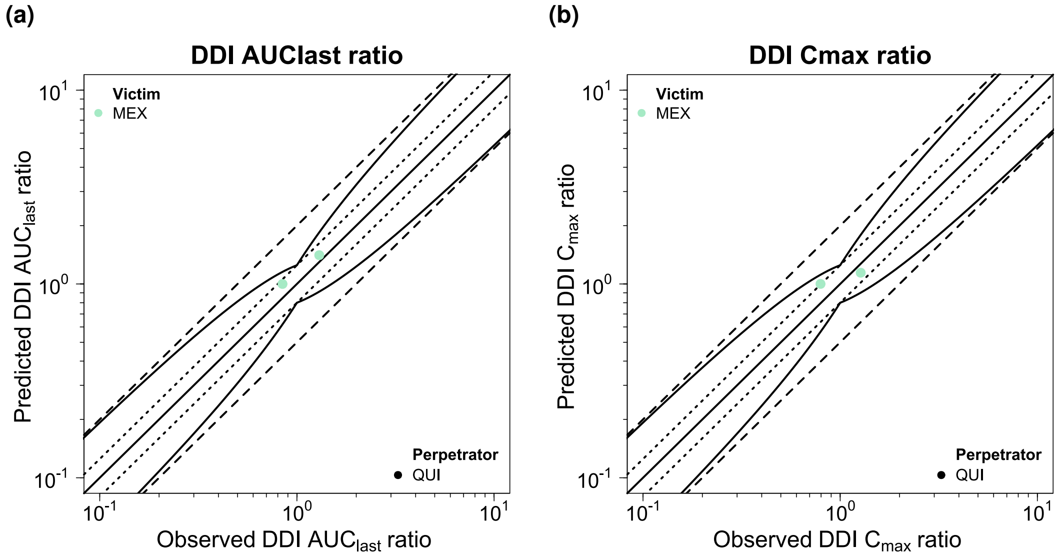

Figure S53: Goodness-of-fit plots comparing predicted and observed DDI AUC_{last} and C_{max} ratios for victim drug mexiletine. The solid line marks the line of identity. Dotted lines indicate 1.25-fold, dashed lines indicate 2-fold deviation. Prediction success limits proposed by Guest et al. [2] are shown as curved lines (including 20% variability). AUC_{last}: area under the plasma concentration-time curve calculated between the first and last concentration measurement, C_{max}: maximum plasma concentration, MEX: mexiletine, QUI: quinidine.

S6.8.5 Geometric Mean Fold Errors of Predicted DDI AUC_{last} and C_{max} Ratios

Table S29: Predicted and observed DDI AUC_{last} and C_{max} ratios involving **mexiletine** as victim drug

**Drug administration**			**DDI AUC_{last} ratio**			**DDI C_{max} ratio**					
**Perpetrator**	**Mexiletine**	**t_{last} [h]**	**Pred**	**Obs**	**Pred/Obs**	**Pred**	**Obs**	**Pred/Obs**	**Phenotype**	**Molecule**	**Reference**
Quinidine											
50 mg q.i.d. po	200 mg s.d. po	48	1.00	0.85	1.18	1.00	0.79	1.26	PM	MEX	Abolfathi 1993 [90]
50 mg q.i.d. po	200 mg s.d. po	48	1.41	1.30	1.08	1.14	1.27	0.90	NM	MEX	Abolfathi 1993 [90]
**Overall GMFE (range):**			**1.13 (1.08–1.18), 2/2 with GMFE $\leq$ 2**			**1.19 (1.11– 1.26), 2/2 with GMFE $\leq$ 2**					

AUC_{last}: area under the plasma concentration-time curve calculated between the first and last concentration measurement, DDI: drug-drug interaction, GMFE: geometric mean fold error, MEX: mexiletine, NM: CYP2D6 normal metabolizer, obs: observed, PM: CYP2D6 poor metabolizer, po: oral, pred: predicted, q.i.d. four times daily, q.d.: once daily, s.d.: single dose, t_{last}: time of the last concentration measurement. If perpetrator or victim drugs were applied in form of salts, the respective dose of base was calculated and incorporated in simulations.

S6.9 Midazolam

S6.9.1 Clinical Studies

Table S30: Clinical study data used for DDI model development with **midazolam** as victim

Drug administration										
**Perpetrator**	**Midazolam**	**n**	**Population[a]**	**Fem. [%]**	**Age [years]**	**Weight [kg]**	**BMI [kg/m²]**	**Phenotype**	**Molecule**	**Reference**
Atomoxetine										
60 mg b.i.d. po	5 mg s.d. po	8	American [7]	50	27 (20–35)	-	(18.1–25.1)	PM	MID	Sauer 2004 [17]

b.i.d.: twice daily, BMI: body mass index, DDI: drug-drug interaction, fem: females, n: number of study participants, PM: CYP2D6 poor metabolizer, po: oral, MID: midazolam -: not available. Values are given as mean (range). If perpetrator or victim drugs were applied in form of salts, the respective dose of base was calculated and incorporated in simulations. [a] Population used in simulations.

S6.9.2 Plasma Concentration-Time Profiles (Semilogarithmic Representation)

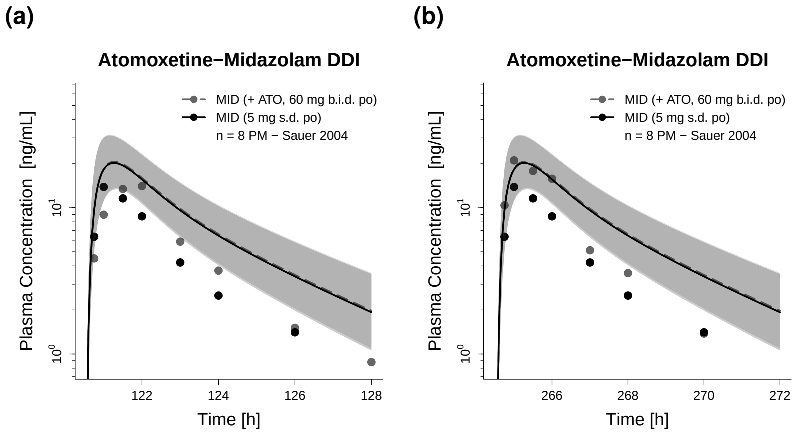

Figure S54: Predicted compared to observed plasma concentration-time profiles of midazolam alone (solid line) and after pretreatment and/or concomitant administration (dashed line) of atomoxetine (semilogarithmic representation). Population predicted (1000 individuals) geometric means are shown as lines, corresponding geometric standard deviations as shaded areas and observed data as dots [17]. ATO: atomoxetine, b.i.d.: twice daily, DDI: drug-drug interaction, MID: midazolam, n: number of study participants, PM: CYP2D6 poor metabolizer, po: oral, s.d.: single dose.

S6.9.3 Plasma Concentration-Time Profiles (Linear Representation)

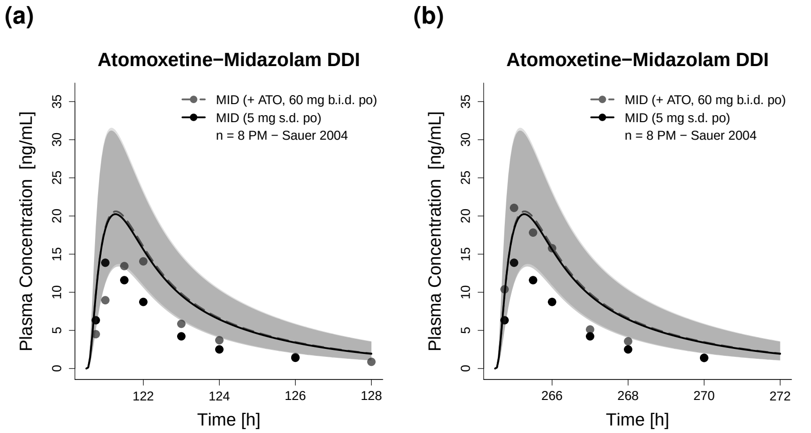

Figure S55: Predicted compared to observed plasma concentration-time profiles of midazolam alone (solid line) and after pretreatment and/or concomitant administration (dashed line) of atomoxetine (linear representation). Population predicted (1000 individuals) geometric means are shown as lines, corresponding geometric standard deviations as shaded areas and observed data as dots [17]. ATO: atomoxetine, b.i.d.: twice daily, DDI: drug-drug interaction, MID: midazolam, n: number of study participants, PM: CYP2D6 poor metabolizer, po: oral, s.d.: single dose.

S6.9.4 DDI AUC_{last} and C_{max} Ratios

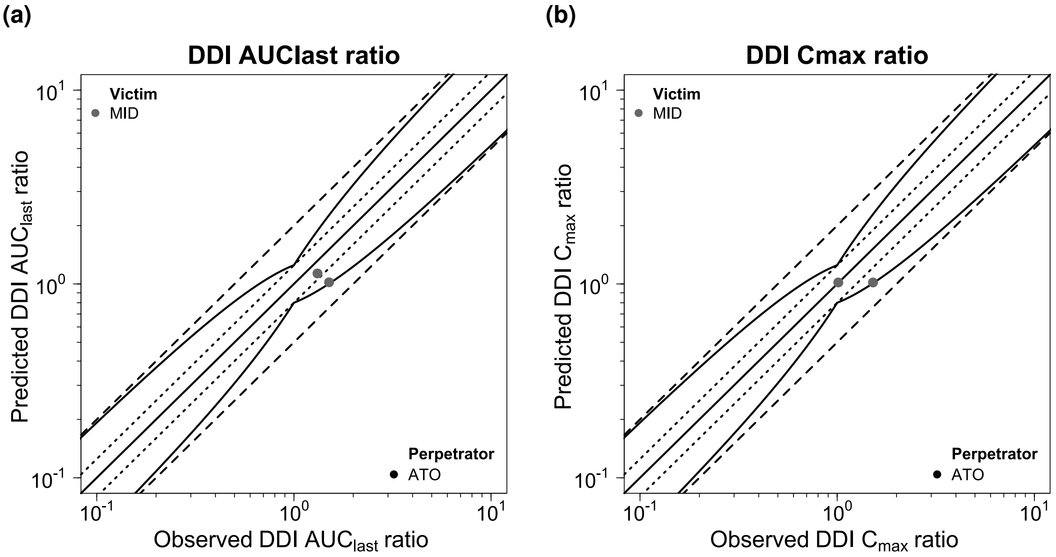

Figure S56: Goodness-of-fit plots comparing predicted and observed DDI AUC_{last} and C_{max} ratios for victim drug midazolam. The solid line marks the line of identity. Dotted lines indicate 1.25-fold, dashed lines indicate 2-fold deviation. Prediction success limits proposed by Guest et al. [2] are shown as curved lines (including 20% variability). ATO: atomoxetine, AUC_{last}: area under the plasma concentration-time curve calculated between the first and last concentration measurement, C_{max}: maximum plasma concentration, MID: midazolam.

S6.9.5 Geometric Mean Fold Errors of Predicted DDI AUC_{last} and C_{max} Ratios

Table S31: Predicted and observed DDI AUC_{last} and C_{max} ratios involving **midazolam** as victim drug

Drug administration			DDI AUC_{last} ratio			DDI C_{max} ratio					
**Perpetrator**	**Midazolam**	**t_{last} [h]**	**Pred**	**Obs**	**Pred/Obs**	**Pred**	**Obs**	**Pred/Obs**	**Phenotype**	**Molecule**	**Reference**
Atomoxetine											
60 mg b.i.d. po	5 mg s.d. po (D6)	5	1.13	1.32	0.86	1.02	1.01	1.00	PM	MID	Sauer 2004 [17]
60 mg b.i.d. po	5 mg s.d. po (D12)	5	1.02	1.51	0.68	1.02	1.52	0.67	PM	MID	Sauer 2004 [17]
**Overall GMFE (range):**			**1.39 (1.31–1.48), 2/2 with GMFE $\leq$ 2**			**1.36 (1.22–1.49), 2/2 with GMFE $\leq$ 2**					

AUC_{last}: area under the plasma concentration-time curve calculated between the first and last concentration measurement, b.i.d.: twice daily, C_{max}: maximum plasma concentration, D: day, DDI: drug-drug interaction, GMFE: geometric mean fold error, MID: midazolam, obs: observed, PM: CYP2D6 poor metabolizer, po: oral, pred: predicted, s.d.: single dose, t_{last}: time of the last concentration measurement. If perpetrator or victim drugs were applied in form of salts, the respective dose of base was calculated and incorporated in simulations.

S6.10 Paroxetine

S6.10.1 Clinical Studies

Table S32: Clinical study data used for DDI model development with **paroxetine** as victim

Drug administration										
**Perpetrator**	**Paroxetine**	**n**	**Population[a]**	**Fem. [%]**	**Age [years]**	**Weight [kg]**	**BMI [kg/m²]**	**Phenotype**	**Molecule**	**Reference**
Itraconazole										
100 mg b.i.d. po	20 mg s.d. po	13	Japanese [113]	77	24.2±3.5	57.3±7.2	-	-	PAR	Yasui-Furukori 2007 [114]
QUI/DEX										
30 mg b.i.d. po	20 mg q.d. po	14	American [7]	14.3	33.6 (19–55)	75.3	25.3	NM	PAR	Schoedel 2012 [101]

b.i.d.: twice daily, BMI: body mass index, DDI: drug-drug interaction, DEX: dextromethorphan, fem: females, n: number of study participants, NM: CYP2D6 normal metabolizer, PAR: paroxetine, po: oral, q.d.: once daily, QUI: quinidine, s.d: single dose, -: not available. Values are given as mean (range). If perpetrator or victim drugs were applied in form of salts, the respective dose of base was calculated and incorporated in simulations. [a] Population used in simulations.

S6.10.2 Plasma Concentration-Time Profiles (Semilogarithmic Representation)

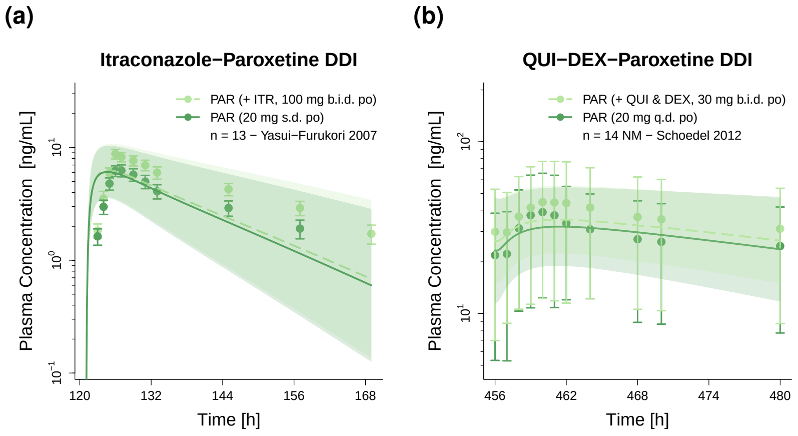

Figure S57: Predicted compared to observed plasma concentration-time profiles of paroxetine alone (solid line) and after pretreatment and/or concomitant administration (dashed line) of (a) itraconazole and (b) quinidine plus dextromethorphan (semilogarithmic representation). Population predicted (1000 individuals) geometric means are shown as lines, corresponding geometric standard deviations as shaded areas and observed data as dots (± standard deviation, if reported) [101, 114]. b.i.d.: twice daily, DDI: drug-drug interaction, DEX: dextromethorphan, ITR: itraconazole, n: number of study participants, PAR: paroxetine, po: oral, q.d.: once daily, QUI: quinidine, s.d.: single dose.

S6.10.3 Plasma Concentration-Time Profiles (Linear Representation)

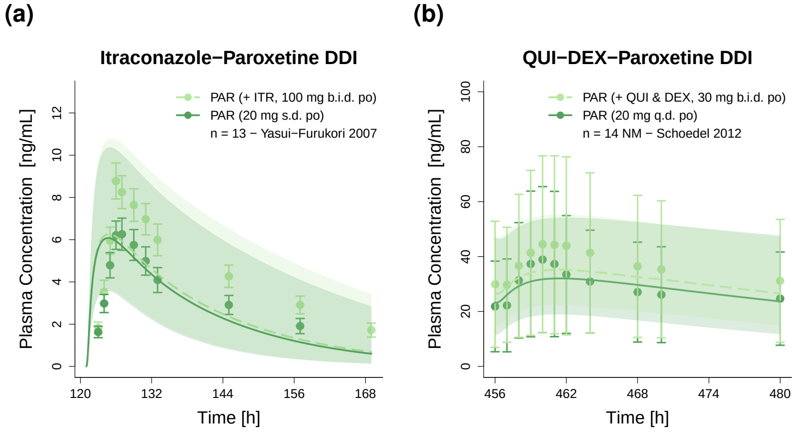

Figure S58: Predicted compared to observed plasma concentration-time profiles of paroxetine alone (solid line) and after pretreatment and/or concomitant administration (dashed line) of (a) itraconazole and (b) quinidine plus dextromethorphan (linear representation). Population predicted (1000 individuals) geometric means are shown as lines, corresponding geometric standard deviations as shaded areas and observed data as dots (± standard deviation, if reported) [101, 114]. b.i.d.: twice daily, DDI: drug-drug interaction, DEX: dextromethorphan, ITR: itraconazole, n: number of study participants, PAR: paroxetine, po: oral, q.d.: once daily, QUI: quinidine, s.d.: single dose.

S6.10.4 DDI AUC_{last} and C_{max} Ratios

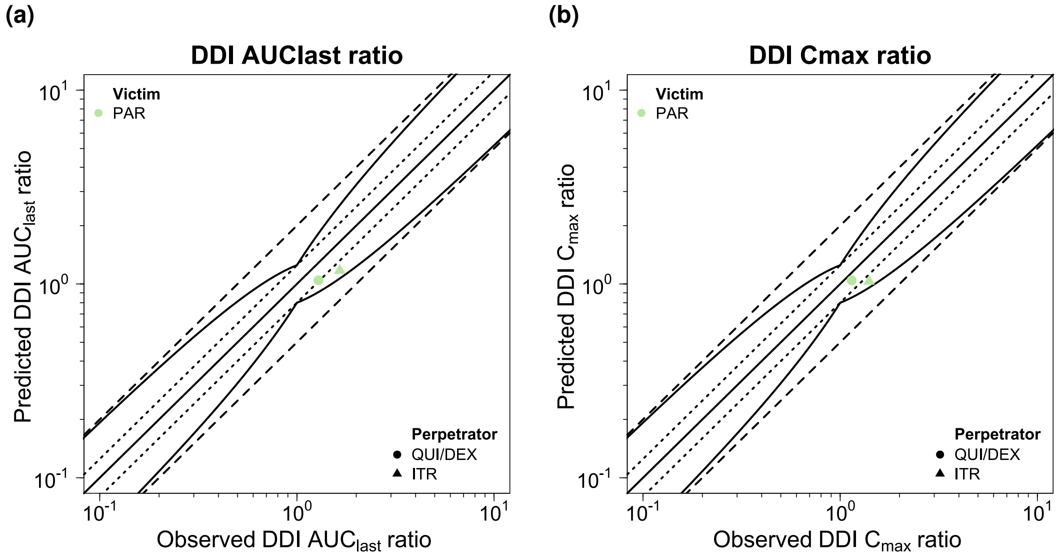

Figure S59: Goodness-of-fit plots comparing predicted and observed DDI AUC_{last} and C_{max} ratios for victim drug paroxetine. The solid line marks the line of identity. Dotted lines indicate 1.25-fold, dashed lines indicate 2-fold deviation. Prediction success limits proposed by Guest et al. [2] are shown as curved lines (including 20% variability). AUC_{last}: area under the plasma concentration-time curve calculated between the first and last concentration measurement, C_{max}: maximum plasma concentration, DEX: dextromethorphan, ITR: itraconazole, PAR: paroxetine, QUI: quinidine.

S6.10.5 Geometric Mean Fold Errors of Predicted DDI AUC_{last} and C_{max} Ratios

Table S33: Predicted and observed DDI AUC_{last} and C_{max} ratios involving **paroxetine** as victim drug

**Drug administration**			**DDI AUC_{last} ratio**			**DDI C_{max} ratio**					
**Perpetrator**	**Paroxetine**	**t_{last} [h]**	**Pred**	**Obs**	**Pred/Obs**	**Pred**	**Obs**	**Pred/Obs**	**Phenotype**	**Molecule**	**Reference**
Itraconazole											
100 mg b.i.d. po	20 mg s.d. po	36	1.17	1.65	0.81	1.03	1.40	0.74	-	PAR	Yasui-Furukori 2007 [114]
**Mean GMFE (range):**			**1.41 (-), 1/1 with GMFE $\leq$ 2**			**1.36 (-), 1/1 with GMFE $\leq$ 2**					
QUI/DEX											
30 mg b.i.d. po	20 mg q.d. po	24	1.04	1.29	0.81	1.04	1.14	0.91	NM	PAR	Schoedel 2012 [101]
**Mean GMFE:**			**1.23 (-), 1/1 with GMFE $\leq$ 2**			**1.10 (-), 1/1 with GMFE $\leq$ 2**					
**Overall GMFE (range):**			**1.32 (1.23–1.41), 2/2 with GMFE $\leq$ 2**			**1.23 (1.10–1.36), 2/2 with GMFE $\leq$ 2**					

AUC_{last}: area under the plasma concentration-time curve calculated between the first and last concentration measurement, b.i.d.: twice daily, C_{max}: maximum plasma concentration, DDI: drug-drug interaction, DEX: dextromethorphan, GMFE: geometric mean fold error, obs: observed, PAR: paroxetine, po: oral, pred: predicted, q.d.: once daily, QUI: quinidine, s.d.: single dose, t_{last}: time of the last concentration measurement. If perpetrator or victim drugs were applied in form of salts, the respective dose of base was calculated and incorporated in simulations.

S6.11 Quinidine

S6.11.1 Clinical Studies

Table S34: Clinical study data used for DDI model development with **quinidine** as victim

Drug administration									
**Perpetrator**	**Quinidine**	**n**	**Population[a]**	**Fem. [%]**	**Age [years]**	**Weight [kg]**	**BMI [kg/m²]**	**Molecule**	**Reference**
Carbamazepine									
200/400 mg b.i.d. po	200 mg s.d. po	10	European [3]	0	(21–26)	(62–85)	(19–26)	QUI, OHQ	Andreasen 2007 [115]
Cimetidine									
300 mg q.d. po	400 mg s.d. po	9	American [7]	0	(21–35)	-	-	QUI	Kolb 1984 [116]
300 mg q.i.d. po	400 mg s.d. po	9	American [7]	0	(21–35)	-	-	QUI	Hardy 1983 [117]
Erythromycin									
250 mg q.i.d. po	200 mg s.d. po	6	European [3]	0	(20–35)	-	-	QUI	Damkier 1999b [118]
Fluvoxamine									
100 mg q.d. po	200 mg s.d. po	6	American [7]	0	-	-	-	QUI, OHQ	Damkier 1999a [122]
Itraconazole									
200 mg q.d. po	100 mg s.d. po	9	European [3]	56	25 (21–32)	64 (41–80)	-	QUI	Kaukonen 1997 [120]
100 mg q.d. po	200 mg s.d. po	6	European [3]	0	(20–35)	-	-	QUI	Damkier 1999b [118]
Omeprazole									
40 mg q.d. po	400 mg s.d. po	8	European [3]	0	(22–29)	(60–94)	-	QUI, OHQ	Ching 1991 [121]
Rifampicin									
600 mg q.d. po	200 mg s.d. po	6	European [3]	0	-	-	-	QUI, OHQ	Damkier 1999 [119]
Verapamil									
80 mg t.i.d. po	400 mg s.d. po	6	European [3]	0	(23–34)	-	-	QUI	Edwards 1987 [123]
120 mg t.i.d. po	400 mg s.d. po	6	European [3]	0	(23–34)	-	-	QUI	Edwards 1987 [123]

b.i.d.: twice daily, BMI: body mass index, DDI: drug-drug interaction, fem: females, n: number of study participants, OHQ: 3-hydroxyquinidine, po: oral, q.d.: once daily, q.i.d.: four times daily, QUI: quinidine, s.d: single dose, t.i.d.: three times daily, -: not available. Values are given as mean (range). If perpetrator or victim drugs were applied in form of salts, the respective dose of base was calculated and incorporated in simulations. [a] Population used in simulations.

S6.11.2 Plasma Concentration-Time Profiles (Semilogarithmic Representation)

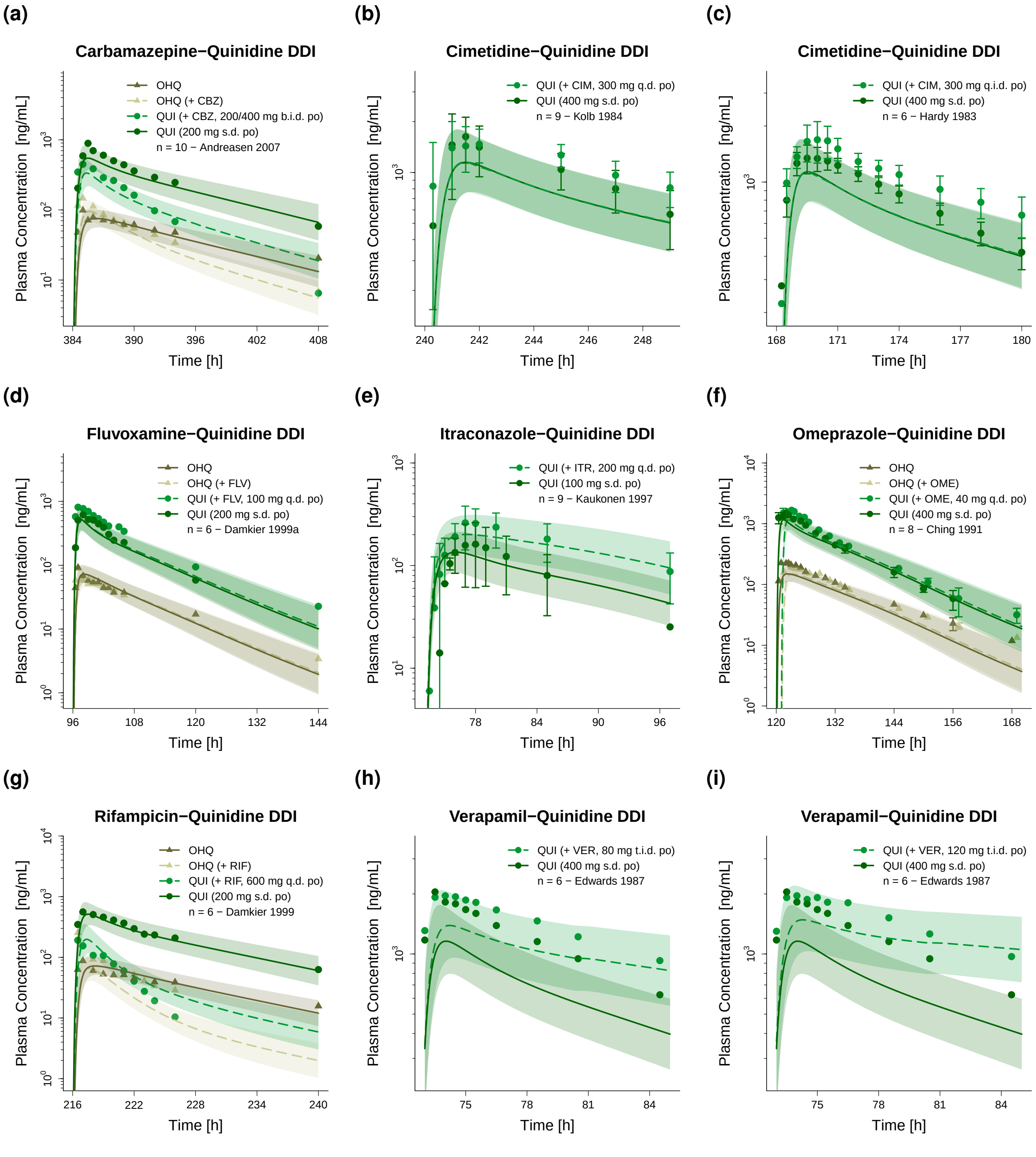

Figure S60: Predicted compared to observed plasma concentration-time profiles of quinidine alone (solid line) and after pretreatment and/or concomitant administration (dashed line) of (a) carbamazepine, (b–c) cimetidine, (d) fluvoxamine, (e) itraconazole, (f) omeprazole, (g) rifampicin and (h–i) verapamil (semilogarithmic representation). Population predicted (1000 individuals) geometric means are shown as lines, corresponding geometric standard deviations as shaded areas and observed data as dots/triangles (± standard deviation, if reported) [115–117, 119–123]. b.i.d.: twice daily, CBZ: carbamazepine, CIM: cimetidine, DDI: drug-drug interaction, FLV: fluvoxamine, ITR: itraconazole, n: number of study participants, OHQ; 3-hydroxyquinidine, OME: omeprazole, po: oral, q.d.: once daily, q.i.d.: four times daily, QUI: quinidine, RIF: rifampicin, s.d.: single dose, t.i.d.: three times daily, VER: verapamil.

S6.11.3 Plasma Concentration-Time Profiles (Linear Representation)

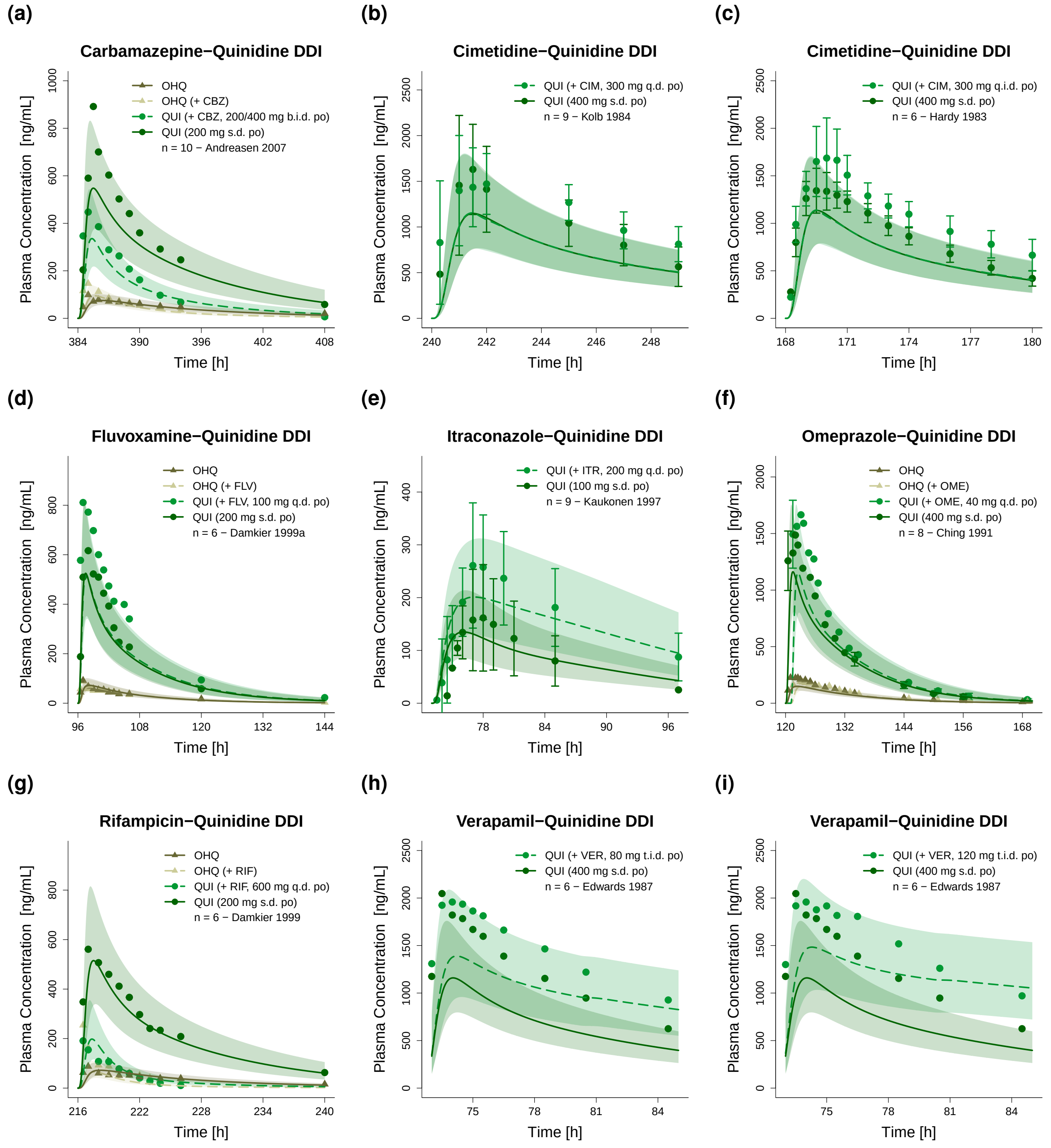

Figure S61: Predicted compared to observed plasma concentration-time profiles of quinidine alone (solid line) and after pretreatment and/or concomitant administration (dashed line) of (a) carbamazepine, (b–c) cimetidine, (d) fluvoxamine, (e) itraconazole, (f) omeprazole, (g) rifampicin and (h–i) verapamil (linear representation). Population predicted (1000 individuals) geometric means are shown as lines, corresponding geometric standard deviations as shaded areas and observed data as dots/triangles (± standard deviation, if reported) [115–117, 119–123]. b.i.d.: twice daily, CBZ: carbamazepine, CIM: cimetidine, DDI: drug-drug interaction, FLV: fluvoxamine, ITR: itraconazole, n: number of study participants, OHQ; 3-hydroxyquinidine, OME: omeprazole, po: oral, q.d.: once daily, q.i.d.: four times daily, QUI: quinidine, RIF: rifampicin, s.d.: single dose, t.i.d.: three times daily, VER: verapamil.

S6.11.4 DDI AUC_{last} and C_{max} Ratios

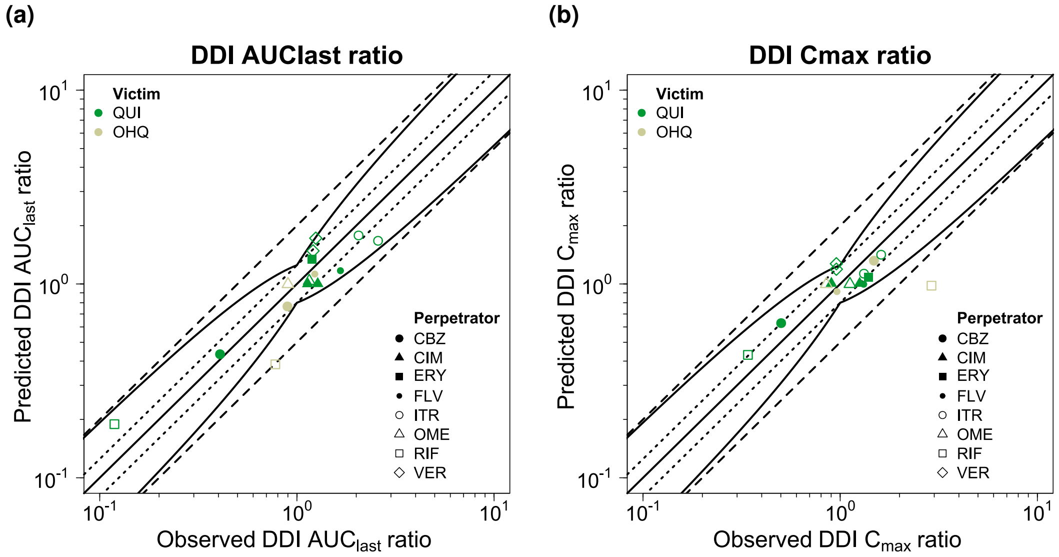

Figure S62: Goodness-of-fit plots comparing predicted and observed DDI AUC_{last} and C_{max} ratios for victim drug quinidine. The solid line marks the line of identity. Dotted lines indicate 1.25-fold, dashed lines indicate 2-fold deviation. Prediction success limits proposed by Guest et al. [2] are shown as curved lines (including 20% variability). AUC_{last}: area under the plasma concentration-time curve calculated between the first and last concentration measurement, CBZ: carbamazepine, CIM: cimetidine, C_{max}: maximum plasma concentration, ERY: erythromycin, FLV: fluvoxamine, ITR: itraconazole, OHQ: 3-hydroxyquinidine, OME: omeprazole, QUI: quinidine, RIF: rifampicin, VER: verapamil.

S6.11.5 Geometric Mean Fold Errors of Predicted DDI AUC_{last} and C_{max} Ratios

Table S35: Predicted and observed DDI AUC_{last} and C_{max} ratios involving **quinidine** as victim drug

Drug administration			DDI AUC_{last} ratio			DDI C_{max} ratio				
**Perpetrator**	**Quinidine**	**t_{last} [h]**	**Pred**	**Obs**	**Pred/Obs**	**Pred**	**Obs**	**Pred/Obs**	**Molecule**	**Reference**
Carbamazepine										
200/400 mg b.i.d. po	200 mg s.d. po	24	0.43	0.41	1.06	0.63	0.50	1.25	QUI	Andreasen 2007 [115]
200/400 mg b.i.d. po	200 mg s.d. po	24	0.77	0.90	0.86	1.32	1.48	0.89	OHQ	Andreasen 2007 [115]
**Mean GMFE (range):**			**1.12 (1.06–1.17), 2/2 with GMFE ≤ 2**			**1.19 (1.12–1.25), 2/2 with GMFE ≤ 2**				
Cimetidine										
300 mg q.d. po	400 mg s.d. po	9	1.00	1.13	0.88	1.01	0.90	1.11	QUI	Kolb 1984 [116]
300 mg q.i.d. po	400 mg s.d. po	12	1.00	1.28	0.79	1.01	1.26	0.80	QUI	Hardy 1983 [117]
**Mean GMFE (range):**			**1.20 (1.13–1.27), 2/2 with GMFE ≤ 2**			**1.18 (1.11–1.25), 2/2 with GMFE ≤ 2**				
Erythromycin										
250 mg q.i.d. po	200 mg s.d. po	inf	1.34	1.19	1.13	1.08	1.39	0.78	QUI	Damkier 1999b [118]
**Mean GMFE (range):**			**1.32 (-), 1/1 with GMFE ≤ 2**			**1.23 (-), 1/1 with GMFE ≤ 2**				
Fluvoxamine										
100 mg q.d. po	200 mg s.d. po	48	1.17	1.66	0.70	1.00	1.32	0.76	QUI	Damkier 1999a [122]
100 mg q.d. po	200 mg s.d. po	48	1.12	1.23	0.91	0.91	0.96	0.95	OHQ	Damkier 1999a [122]
**Mean GMFE (range):**			**1.26 (1.09–1.42), 2/2 with GMFE ≤ 2**			**1.19 (1.05–1.32), 2/2 with GMFE ≤ 2**				
Itraconazole										
200 mg q.d. po	100 mg s.d. po	24	1.78	2.05	0.87	1.42	1.61	0.88	QUI	Kaukonen 1997 [120]
100 mg q.d. po	200 mg s.d. po	inf	1.67	2.58	0.65	1.13	1.32	0.86	QUI	Damkier 1999b [118]
**Mean GMFE:**			**1.24 (1.15–1.32), 2/2 with GMFE ≤ 2**			**1.18 (1.14–1.23), 2/2 with GMFE ≤ 2**				
Omeprazole										
40 mg q.d. po	400 mg s.d. po	48	1.04	1.15	0.90	1.00	1.12	0.89	QUI	Ching 1991 [121]
40 mg q.d. po	400 mg s.d. po	48	1.00	0.90	1.11	1.00	0.85	1.17	OHQ	Ching 1991 [121]
**Mean GMFE (range):**			**1.11 (1.11–1.12), 2/2 with GMFE ≤ 2**			**1.15 (1.12–1.17), 2/2 with GMFE ≤ 2**				
Rifampicin										
600 mg q.d. po	200 mg s.d. po	10	0.19	0.12	1.59	0.43	0.34	1.26	QUI	Damkier 1999 [119]
600 mg q.d. po	200 mg s.d. po	10	0.38	0.78	0.50	0.98	2.90	0.34	OHQ	Damkier 1999 [119]
**Mean GMFE (range):**			**1.81 (1.59–2.02), 1/2 with GMFE ≤ 2**			**2.11 (1.26–2.96), 1/2 with GMFE ≤ 2**				
Verapamil										
80 mg t.i.d. po	400 mg s.d. po	12	1.48	1.21	1.23	1.19	0.96	1.25	QUI	Edwards 1987 [123]
120 mg t.i.d. po	400 mg s.d. po	12	1.72	1.25	1.38	1.27	0.96	1.33	QUI	Edwards 1987 [123]
**Mean GMFE (range):**			**1.30 (1.23–1.38), 2/2 with GMFE ≤ 2**			**1.29 (1.25–1.33), 2/2 with GMFE ≤ 2**				
**Overall GMFE (range):**			**1.29 (1.06–2.02), 14/15 with GMFE ≤ 2**			**1.32 (1.05–2.96), 14/15 with GMFE ≤ 2**				

AUC_{last}: area under the plasma concentration-time curve calculated between the first and last concentration measurement, b.i.d.: twice daily, C_{max}: maximum plasma concentration, DDI: drug-drug interaction, GMFE: geometric mean fold error, obs: observed, OHQ: 3-hydroxyquinidine, po: oral, pred: predicted, q.d.: once daily, q.i.d.: four times daily, QUI: quinidine, s.d.: single dose, t.i.d.: three times daily, t_{last}: time of the last concentration measurement. If perpetrator or victim drugs were applied in form of salts, the respective dose of base was calculated and incorporated in simulations.

S6.12 Risperidone

S6.12.1 Clinical Studies

Table S36: Clinical study data used for DDI model development with **risperidone** as victim

Drug administration										
**Perpetrator**	**Risperidone**	**n**	**Population[a]**	**Fem. [%]**	**Age [years]**	**Weight [kg]**	**BMI [kg/m²]**	**AS**	**Molecule**	**Reference**
Ketoconazole										
200 mg q.d. po	2 mg s.d. po	10	Asian [98]	0	33.3±8.1	64.1±5.8	165.8±7.3[b]	-	RIS, OHR	Mahatthanatrakul 2012 [124]
Rifampicin										
600 mg q.d. po	1 mg s.d po	10	Asian [98]	0	(23–38)	(65–80)	-	AS = 1.25	RIS, OHR	Kim 2018 [125]
600 mg q.d. po	4 mg s.d. po	10	Asian [98]	0	30.5±6.5	(55–76)	22.1±2.2	-	RIS	Mahatthanatrakul 2007 [126]
Verapamil										
80 mg t.i.d. po	1 mg s.d. po	12	Japanese [113]	0	24.0±2.0	64.8±6.2	-	AS = 1	RIS, OHR	Nakagami 2005 [127]

AS: CYP2D6 activity score, BMI: body mass index, DDI: drug-drug interaction, fem: females, n: number of study participants, OHR: 9-hydroxyrisperidone, po: oral, q.d.: once daily, RIS: risperidone, s.d: single dose, t.i.d.: three times daily, -: not available. Values are given as mean (range). If perpetrator or victim drugs were applied in form of salts, the respective dose of base was calculated and incorporated in simulations. [a] Population used in simulations. [b] Height of subjects [cm].

S6.12.2 Plasma Concentration-Time Profiles (Semilogarithmic Representation)

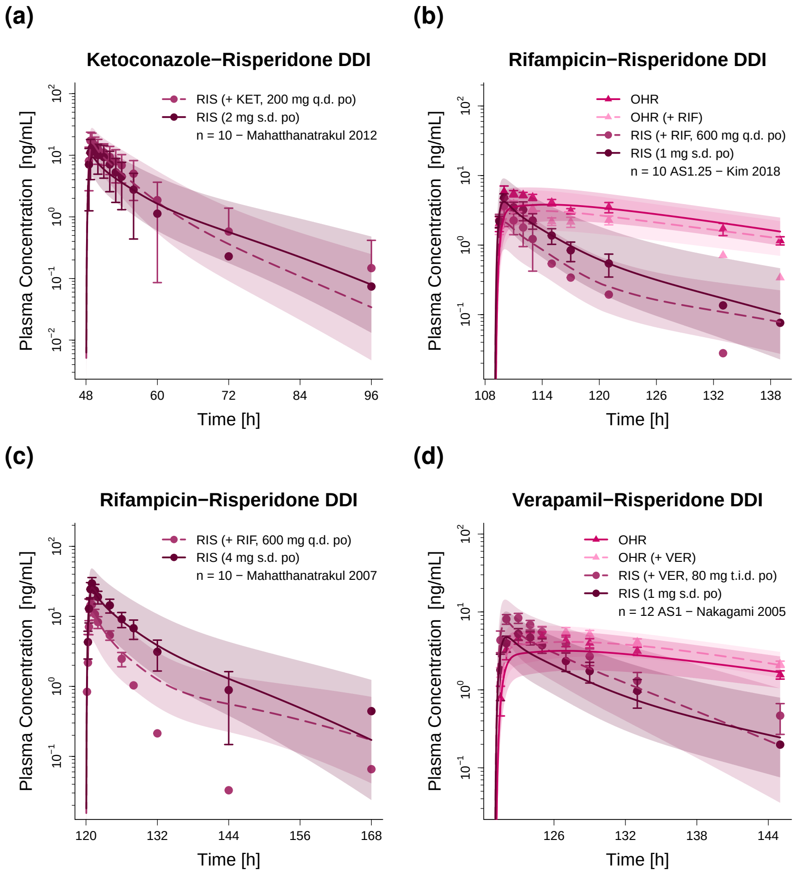

Figure S63: Predicted compared to observed plasma concentration-time profiles of risperidone alone (solid line) and after pretreatment and/or concomitant administration (dashed line) of (a) ketoconazole, (b–c) rifampicin and (d) verapamil (semilogarithmic representation). Population predicted (1000 individuals) geometric means are shown as lines, corresponding geometric standard deviations as shaded areas and observed data as dots/triangles (± standard deviation, if reported) [124–127]. AS: CYP2D6 activity score, DDI: drug-drug interaction, KET: ketoconazole, n: number of study participants, OHR: 9-hydroxyrisperidone, po: oral, q.d.: once daily, RIF: rifampicin, s.d.: single dose, t.i.d.: three times daily, VER: verapamil.

S6.12.3 Plasma Concentration-Time Profiles (Linear Representation)

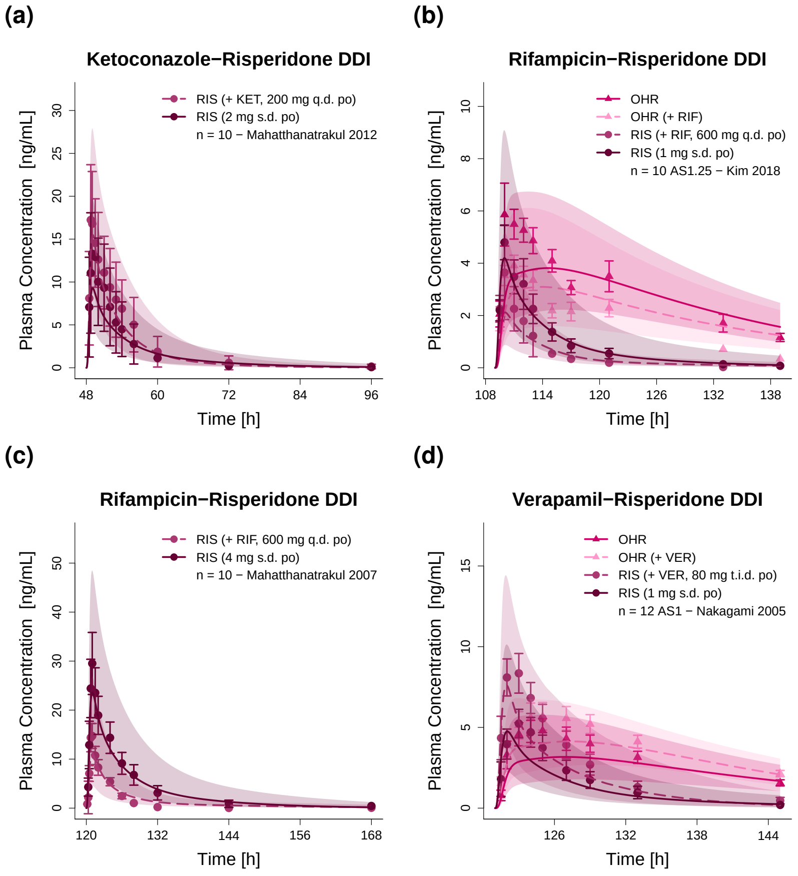

Figure S64: AS: CYP2D6 activity score, Predicted compared to observed plasma concentration-time profiles of risperidone alone (solid line) and after pretreatment and/or concomitant administration (dashed line) of (a) ketoconazole, (b–c) rifampicin and (d) verapamil (linear representation). Population predicted (1000 individuals) geometric means are shown as lines, corresponding geometric standard deviations as shaded areas and observed data as dots/triangles (± standard deviation, if reported) [124–127]. DDI: drug-drug interaction, KET: ketoconazole, n: number of study participants, OHR: 9-hydroxyrisperidone, po: oral, q.d.: once daily, RIF: rifampicin, s.d.: single dose, t.i.d.: three times daily, VER: verapamil.

S6.12.4 DDI AUC_{last} and C_{max} Ratios

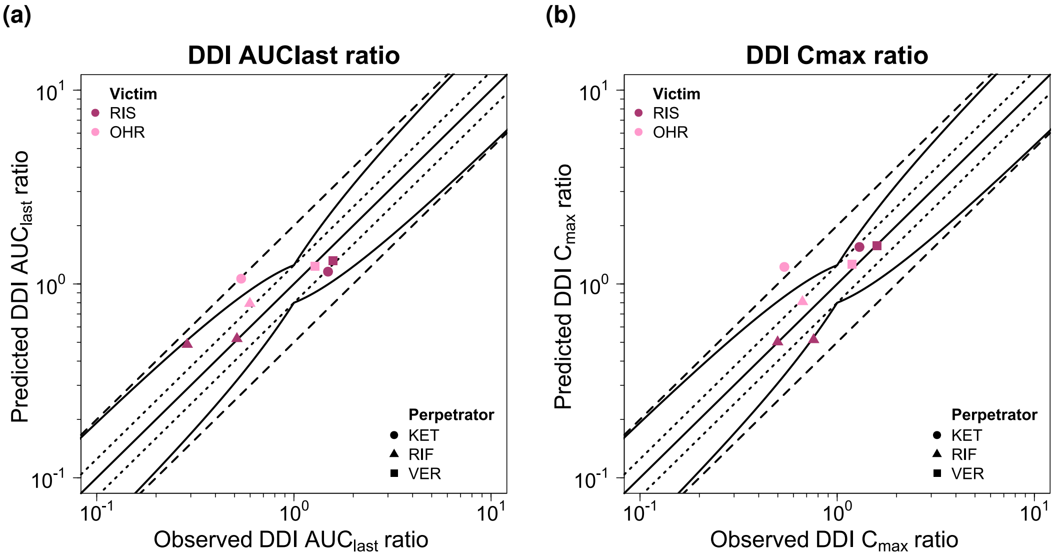

Figure S65: Goodness-of-fit plots comparing predicted and observed DDI AUC_{last} and C_{max} ratios for victim drug risperidone. The solid line marks the line of identity. Dotted lines indicate 1.25-fold, dashed lines indicate 2-fold deviation. Prediction success limits proposed by Guest et al. [2] are shown as curved lines (including 20% variability). AUC_{last}: area under the plasma concentration-time curve calculated between the first and last concentration measurement, C_{max}: maximum plasma concentration, KET: ketoconazole, OHR: 9-hydroxyrisperidone, RIF: rifampicin, RIS: risperidone, VER: verapamil.

S6.12.5 Geometric Mean Fold Errors of Predicted DDI AUC_{last} and C_{max} Ratios

Table S37: Predicted and observed DDI AUC_{last} and C_{max} ratios involving **risperidone** as victim drug

**Drug administration**			**DDI AUC_{last} ratio**			**DDI C_{max} ratio**					
**Perpetrator**	**Risperidone**	**t_{last} [h]**	**Pred**	**Obs**	**Pred/Obs**	**Pred**	**Obs**	**Pred/Obs**	**AS**	**Molecule**	**Reference**
Ketoconazole											
200 mg q.d. po	2 mg s.d. po	48	1.16	1.49	0.78	1.55	1.30	1.20	-	RIS	Mahatthanatrakul 2012 [124]
200 mg q.d. po	2 mg s.d. po	96	1.06	0.54	1.97	1.22	0.54	2.27	-	OHR	Mahatthanatrakul 2012 [124]
**Mean GMFE (range):**			**1.63 (1.29–1.97), 2/2 with GMFE $\leq$ 2**			**1.73 (1.20–2.27), 1/2 with GMFE $\leq$ 2**					
Rifampicin											
600 mg q.d. po	1 mg s.d po	24	0.52	0.51	1.02	0.52	0.76	0.68	AS = 1.25	RIS	Kim 2018 [125]
600 mg q.d. po	1 mg s.d po	30	0.79	0.60	1.33	0.81	0.67	1.21	AS = 1.25	OHR	Kim 2018 [125]
600 mg q.d. po	4 mg s.d. po	48	0.49	0.29	1.69	0.50	0.50	1.01		RIS	Mahatthanatrakul 2007 [126]
**Mean GMFE (range):**			**1.35 (1.02–1.69), 3/3 with GMFE $\leq$ 2**			**1.23 (1.01–1.47), 3/3 with GMFE $\leq$ 2**					
Verapamil											
80 mg t.i.d. po	1 mg s.d. po	24	1.32	1.58	0.83	1.58	1.59	0.99	AS = 1	RIS	Nakagami 2005 [127]
80 mg t.i.d. po	1 mg s.d. po	24	1.23	1.28	0.96	1.27	1.19	1.07	AS = 1	OHR	Nakagami 2005 [127]
**Mean GMFE (range):**			**1.12 (1.04–1.20), 2/2 with GMFE $\leq$ 2**			**1.04 (1.01–1.07), 2/2 with GMFE $\leq$ 2**					
**Overall GMFE (range):**			**1.36 (1.02–1.97), 7/7 with GMFE $\leq$ 2**			**1.32 (1.01–2.27), 6/7 with GMFE $\leq$ 2**					

AS: CYP2D6 activity score, AUC_{last}: area under the plasma concentration-time curve calculated between the first and last concentration measurement, C_{max}: maximum plasma concentration, DDI: drug-drug interaction, GMFE: geometric mean fold error, obs: observed, OHR: 9-hydroxyrisperidone, po: oral, pred: predicted, q.d.: once daily, RIS: risperidone, s.d.: single dose, t.i.d.: three times daily, t_{last}: time of the last concentration measurement. If perpetrator or victim drugs were applied in form of salts, the respective dose of base was calculated and incorporated in simulations.

S6.13 DDI AUC_{last} and C_{max} Ratios

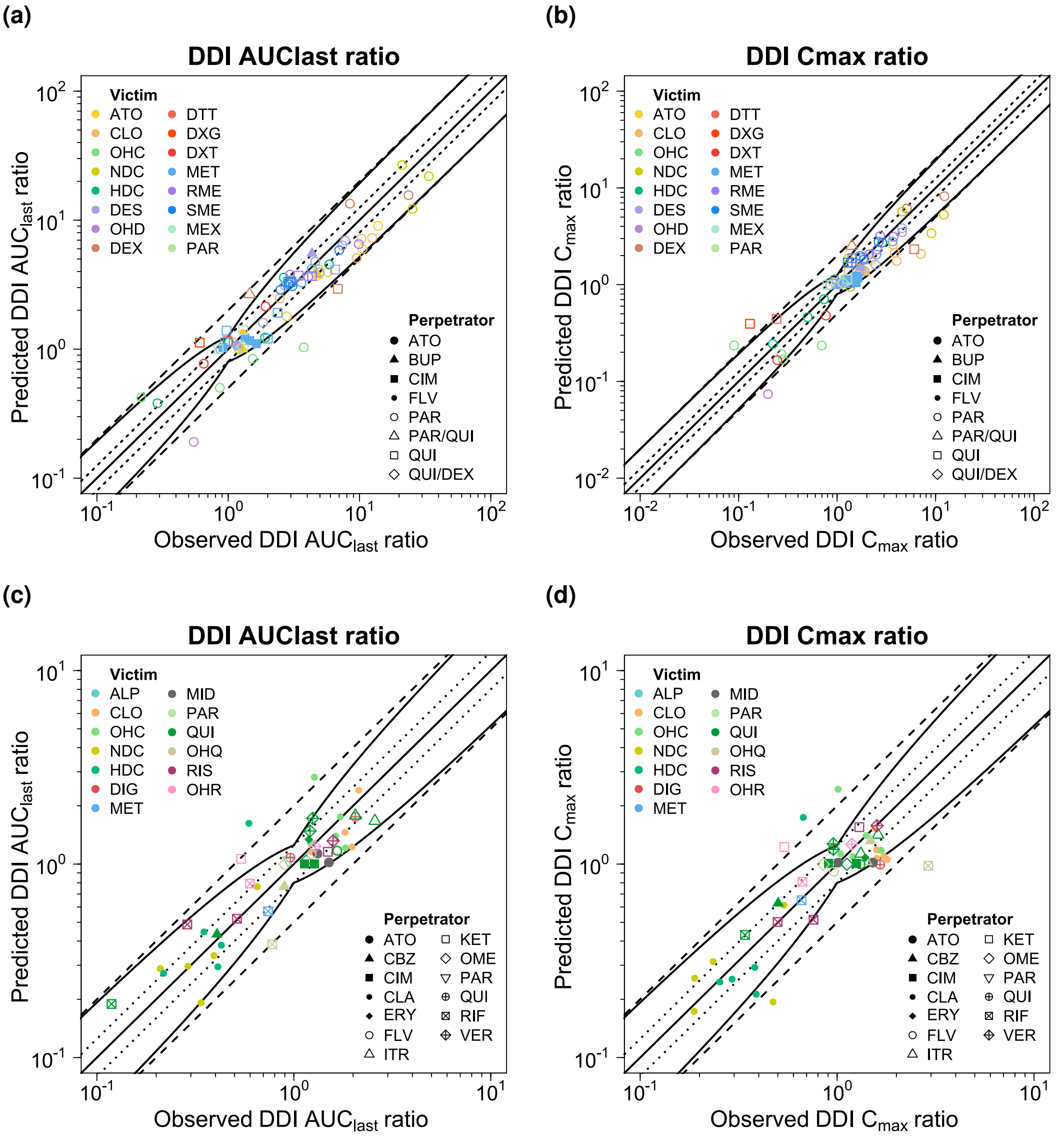

Figure S66: Goodness-of-fit plots comparing predicted and observed DDI AUC_{last} and C_{max} ratios. The solid line marks the line of identity. Dotted lines indicate 1.25-fold, dashed lines indicate 2-fold deviation. Prediction success limits proposed by Guest et al. [2] are shown as curved lines (including 20% variability). ALP: alprazolam, ATO: atomoxetine, AUC_{last}: area under the plasma concentration-time curve calculated between the first and last concentration measurement, BUP: bupropion, CBZ: carbamazepine, CIM: cimetidine, CLA: clarithromycin, CLO: (*E*)-clomiphene, C_{max}: maximum plasma concentration, DES: desipramine, DEX: dextromethorphan, DGI: drug-gene interaction, DIG: digoxin, DTT total dextrorphan:, DXG: dextrorphan-O-glucuronide, DXT: dextrorphan, ERX: erythromycin, FLV: fluvoxamine, HDC: (*E*)-4-hydroxy-N-desethylclomiphene, ITR: itraconazole, KET: ketoconazole, MET: metoprolol, MEX: mexiletine, MID: midazolam, NDC: (*E*)-N-desethylclomiphene, OHC: (*E*)-4-hydroxyclomiphene, OHD: 2-hydroxydesipramine, OHM: alpha-hydroxymetoprolol, OHQ: 3-hydroxyquinidine, OHR: 9-hydroxyrisperidone, OME: omeprazole, PAR: paroxetine, QUI: quinidine, RIF: rifampicin, RIS: risperidone, RME: R-metoprolol, SME: S-metoprolol, VER: verapamil.

S7 DDGI Model Evaluation

Comprehensive information on the modeled DDGI studies as well as respective predicted and observed plasma concentration-time profiles in semilogarithmic and linear representations can be found in Section S6 "DDI Model Evaluation".

S7.1 DDGI AUC_{last} and C_{max} Ratios

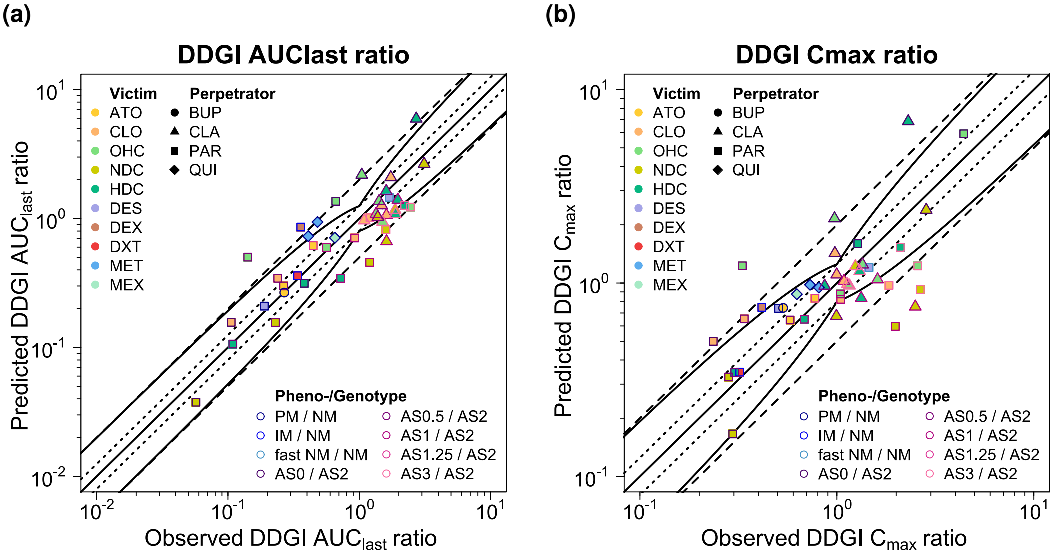

Figure S67: Goodness-of-fit plots comparing predicted and observed DDGI AUC_{last} and C_{max} ratios. The solid line marks the line of identity. Dotted lines indicate 1.25-fold, dashed lines indicate 2-fold deviation. Prediction success limits proposed by Guest et al. [2] are shown as curved lines (including 20% variability). AS: CYP2D6 activity score, ATO: atomoxetine, AUC_{last}: area under the plasma concentration-time curve calculated between the first and last concentration measurement, BUP: bupropion, CLA: clarithromycin, CLO: (*E*)-clomiphene, C_{max}: maximum plasma concentration, DES: desipramine, DEX: dextromethorphan, DDGI: drug-drug-gene interaction, DXT: dextrorphan, HDC: (*E*)-4-hydroxy-N-desethylclomiphene, IM: CYP2D6 intermediate metabolizer, MET: metoprolol, MEX: mexiletine, NDC: (*E*)-N-desethylclomiphene, NM: CYP2D6 normal metabolizer, OHC: (*E*)-4-hydroxyclomiphene, PAR: paroxetine, PM: CYP2D6 poor metabolizer, QUI: quinidine.

S7.2 Geometric Mean Fold Errors of Predicted DDGI AUC_{last} and C_{max} Ratios

Table S38: Predicted and observed DDGI AUC_{last} and C_{max} ratios

Drug administration			DDGI AUC_{last} ratio			DDGI C_{max} ratio					
Victim	Perpetrator	t_{last} [h]	Pred	Obs	Pred/Obs	Pred	Obs	Pred/Obs	Phenotype/AS	Molecule	Reference
Atomoxetine	***Bupropion***										
25 mg s.d. po	150/300 mg q.d. po	48 / 48	0.27	0.27	0.99	0.74	0.53	1.39	PM / NM	ATO	Todor 2016 [74]
**Mean GMFE (range):**					**1.01 (-), 1/1 with GMFE $\leq$ 2**			**1.39 (-), 1/1 with GMFE $\leq$ 2**			
Atomoxetine	***Paroxetine***										
20 mg s.d. po	20 mg q.d. po	24 / 24	0.30	0.26	1.13	0.64	0.58	1.11	AS0 / AS2	ATO	Jung 2020 [71]
20 mg s.d. po	20 mg q.d. po	24 / 24	0.62	0.45	1.39	0.83	0.77	1.08	AS1.25 / AS2	ATO	Jung 2020 [71]
**Mean GMFE (range):**					**1.26 (1.13–1.39), 2/2 with GMFE $\leq$ 2**			**1.10 (1.08–1.11), 2/2 with GMFE $\leq$ 2**			
(E)-Clomiphene	***Clarithromycin***										
42 mg s.d. po	500 mg b.i.d po	168 / 72	2.09	1.74	1.20	1.43	0.98	1.46	AS0 / AS2	CLO	Mürdter 2016 [76]
42 mg s.d. po	500 mg b.i.d po	168 / 168	2.18	1.04	2.09	2.16	0.97	2.22	AS0 / AS2	OHC	Mürdter 2016 [76]
42 mg s.d. po	500 mg b.i.d po	168 / 72	2.65	3.11	0.85	2.39	2.84	0.84	AS0 / AS2	NDC	Mürdter 2016 [76]
42 mg s.d. po	500 mg b.i.d po	168 / 72	5.95	2.71	2.20	6.86	2.30	2.98	AS0 / AS2	HDC	Mürdter 2016 [76]
42 mg s.d. po	500 mg b.i.d po	168 / 72	1.26	1.48	0.86	1.11	1.00	1.11	AS0.5 / AS2	CLO	Mürdter 2016 [76]
42 mg s.d. po	500 mg b.i.d po	168 / 168	1.36	1.41	0.96	1.24	1.35	0.92	AS0.5 / AS2	OHC	Mürdter 2016 [76]
42 mg s.d. po	500 mg b.i.d po	168 / 72	1.03	1.38	0.74	0.68	0.99	0.68	AS0.5 / AS2	NDC	Mürdter 2016 [76]
42 mg s.d. po	500 mg b.i.d po	168 / 72	1.64	1.60	1.02	0.97	0.87	1.12	AS0.5 / AS2	HDC	Mürdter 2016 [76]
42 mg s.d. po	500 mg b.i.d po	72 / 72	1.06	1.61	0.66	1.02	1.09	0.94	AS1 / AS2	CLO	Mürdter 2016 [76]
42 mg s.d. po	500 mg b.i.d po	168 / 168	1.08	1.34	0.81	1.04	1.61	0.65	AS1 / AS2	OHC	Mürdter 2016 [76]
42 mg s.d. po	500 mg b.i.d po	72 / 72	0.67	1.61	0.41	0.75	2.50	0.30	AS1 / AS2	NDC	Mürdter 2016 [76]
42 mg s.d. po	500 mg b.i.d po	168 / 72	1.40	1.96	0.71	0.84	1.33	0.63	AS1 / AS2	HDC	Mürdter 2016 [76]
42 mg s.d. po	500 mg b.i.d po	72 / 72	0.97	1.09	0.89	0.99	1.13	0.87	AS3 / AS2	CLO	Mürdter 2016 [76]
42 mg s.d. po	500 mg b.i.d po	168 / 168	0.94	1.50	0.63	0.97	1.16	0.84	AS3 / AS2	OHC	Mürdter 2016 [76]
42 mg s.d. po	500 mg b.i.d po	72 / 72	1.17	1.87	0.62	1.22	1.24	0.98	AS3 / As2	NDC	Mürdter 2016 [76]
42 mg s.d. po	500 mg b.i.d po	72 / 72	1.08	1.89	0.57	1.15	1.30	0.88	AS3 / AS2	HDC	Mürdter 2016 [76]
**Mean GMFE (range):**					**1.49 (1.02–2.42), 13/16 with GMFE $\leq$ 2**			**1.54 (1.02–3.31), 13/16 with GMFE $\leq$ 2**			
(E)-Clomiphene	***Paroxetine***										
42 mg s.d. po	40 mg q.d. po	168 / 168	0.16	0.11	1.48	0.50	0.24	2.12	AS0 / AS2	CLO	Mürdter 2016 [76]
42 mg s.d. po	40 mg q.d. po	168 / 168	1.36	0.66	2.06	5.92	4.40	1.34	AS0 / AS2	OHC	Mürdter 2016 [76]
42 mg s.d. po	40 mg q.d. po	168 / 168	0.04	0.06	0.66	0.17	0.30	0.56	AS0 / AS2	NDC	Mürdter 2016 [76]
42 mg s.d. po	40 mg q.d. po	168 / 168	0.31	0.38	0.83	1.60	1.28	1.25	AS0 / AS2	HDC	Mürdter 2016 [76]
42 mg s.d. po	40 mg q.d. po	168 / 168	0.34	0.24	1.44	0.65	0.34	1.93	AS0.5 / AS2	CLO	Mürdter 2016 [76]
42 mg s.d. po	40 mg q.d. po	168 / 168	0.50	0.14	3.55	1.23	0.33	3.71	AS0.5 / AS2	OHC	Mürdter 2016 [76]
42 mg s.d. po	40 mg q.d. po	168 / 168	0.16	0.23	0.68	0.33	0.28	1.16	AS0.5 / AS2	NDC	Mürdter 2016 [76]
42 mg s.d. po	40 mg q.d. po	168 / 168	0.11	0.11	0.98	0.34	0.30	1.14	AS0.5 / AS2	HDC	Mürdter 2016 [76]
42 mg s.d. po	40 mg q.d. po	168 / 168	0.71	0.92	0.77	0.82	1.04	0.79	AS1 / AS2	CLO	Mürdter 2016 [76]
42 mg s.d. po	40 mg q.d. po	168 / 168	0.60	0.56	1.06	0.88	1.04	0.84	AS1 / AS2	OHC	Mürdter 2016 [76]
42 mg s.d. po	40 mg q.d. po	168 / 168	0.46	1.20	0.38	0.60	1.98	0.30	AS1 / AS2	NDC	Mürdter 2016 [76]
42 mg s.d. po	40 mg q.d. po	168 / 168	0.34	0.72	0.48	0.65	0.68	0.95	AS1 / AS2	HDC	Mürdter 2016 [76]

AS: CYP2D6 activity score, ATO: atomoxetine, AUC_{last}: area under the plasma concentration-time curve calculated between the first and last concentration measurement, b.i.d.: twice daily, CLO: (*E*)-clomiphene, C_{max}: maximum plasma concentration, DES: desipramine, DEX: dextromethorphan, DDGI: drug-drug-gene interaction, DXT: dextrorphan, GMFE: geometric mean fold error, HDC: (*E*)-4-hydroxy-N-desethylclomiphene, IM: CYP2D6 intermediate metabolizer, iv: intravenous, MET: metoprolol, MEX: mexiletine, NDC: (*E*)-N-desethylclomiphene, NM: CYP2D6 normal metabolizer, obs: observed, OHC: (*E*)-4-hydroxyclomiphene, PM: CYP2D6 poor metabolizer, po: oral, pred: predicted, q.d.: once daily, s.d.: single dose, t_{last}: time of the last concentration measurement. If perpetrator or victim drugs were applied in form of salts, the respective dose of base was calculated and incorporated in simulations.

Table S38: Predicted and observed DDGI AUC_{last} and C_{max} ratios *(continued)*

Drug administration			DDGI AUC_{last} ratio			DDGI C_{max} ratio					
**Victim**	**Perpetrator**	**t_{last} [h]**	**Pred**	**Obs**	**Pred/Obs**	**Pred**	**Obs**	**Pred/Obs**	**Phenotype/AS**	**Molecule**	**Reference**
42 mg s.d. po	40 mg q.d. po	168 / 168	1.01	1.20	0.84	0.97	1.84	0.53	AS3 / AS2	CLO	Mürdter 2016 [76]
42 mg s.d. po	40 mg q.d. po	168 / 168	1.23	2.45	0.50	1.23	2.57	0.48	AS3 / AS2	OHC	Mürdter 2016 [76]
42 mg s.d. po	40 mg q.d. po	168 / 168	0.82	1.59	0.51	0.93	2.65	0.35	AS3 / AS2	NDC	Mürdter 2016 [76]
42 mg s.d. po	40 mg q.d. po	168 / 168	1.26	2.21	0.57	1.53	2.10	0.73	AS3 / AS2	HDC	Mürdter 2016 [76]
**Mean GMFE (range):**			**1.73 (1.02–3.55), 12/16 with GMFE $\leq$ 2**			**1.84 (1.05–3.71), 11/16 with GMFE $\leq$ 2**					
Desipramine	***Paroxetine***										
100 mg s.d. po	20 mg q.d. po	239 / 234	0.21	0.19	1.11	0.74	0.51	1.46	PM / NM	DES	Brøsen 1993 [22]
100 mg s.d. po	20 mg q.d. po	233 / 234	1.46	1.69	0.87	1.20	1.46	0.83	fast NM / NM	DES	Brøsen 1993 [22]
**Mean GMFE (range):**			**1.13 (1.11–1.16), 2/2 with GMFE $\leq$ 2**			**1.34 (1.21–1.46), 2/2 with GMFE $\leq$ 2**					
Dextromethorphan	***Paroxetine***										
5 mg s.d. po	20 mg b.i.d. po	24 / 24	0.86	0.36	2.40	0.75	0.42	1.80	IM / NM	DEX	Storelli 2018 [77]
5 mg s.d. po	20 mg b.i.d. po	24 / 24	0.36	0.34	1.06	0.35	0.32	1.08	IM / NM	DXT	Storelli 2018 [77]
**Mean GMFE (range):**			**1.73 (1.06–2.40), 1/2 with GMFE $\leq$ 2**			**1.44 (1.08–1.80), 2/2 with GMFE $\leq$ 2**					
Metoprolol	***Quinidine***										
20 mg s.d. iv	50 mg s.d. po	8 / 8	0.94	0.48	1.98	0.98	0.73	1.35	PM / NM	MET	Leemann 1993 [82]
20 mg s.d. iv	250 mg b.i.d. po	8 / 8	0.73	0.41	1.79	0.95	0.81	1.18	PM / NM	MET	Leemann 1993 [82]
**Mean GMFE (range):**			**1.87 (1.79–1.96), 2/2 with GMFE $\leq$ 2**			**1.26 (1.17–1.34), 2/2 with GMFE $\leq$ 2**					
Mexiletine	***Quinidine***										
200 mg s.d. po	50 mg q.i.d. po	48 / 48	0.71	0.65	1.09	0.88	0.63	1.40	PM / NM	MEX	Abolfathi 1993 [90]
**Mean GMFE (range):**			**1.09 (-), 1/1 with GMFE $\leq$ 2**			**1.40 (-), 1/1 with GMFE $\leq$ 2**					
**Overall GMFE (range):**			**1.56 (1.01–3.55), 34/42 with GMFE $\leq$ 2**			**1.60 (1.02–3.71), 34/42 with GMFE $\leq$ 2**					

AS: CYP2D6 activity score, ATO: atomoxetine, AUC_{last}: area under the plasma concentration-time curve calculated between the first and last concentration measurement, b.i.d.: twice daily, CLO: (*E*)-clomiphene, C_{max}: maximum plasma concentration, DES: desipramine, DEX: dextromethorphan, DDGI: drug-drug-gene interaction, DXT: dextrorphan, GMFE: geometric mean fold error, HDC: (*E*)-4-hydroxy-N-desethylclomiphene, IM: CYP2D6 intermediate metabolizer, iv: intravenous, MET: metoprolol, MEX: mexiletine, NDC: (*E*)-N-desethylclomiphene, NM: CYP2D6 normal metabolizer, obs: observed, OHC: (*E*)-4-hydroxyclomiphene, PM: CYP2D6 poor metabolizer, po: oral, pred: predicted, q.d.: once daily, s.d.: single dose, t_{last}: time of the last concentration measurement. If perpetrator or victim drugs were applied in form of salts, the respective dose of base was calculated and incorporated in simulations.

S8 Model-Informed Dose Adaptations

Table S39: Administration protocols used for model-informed dose adaptations

	Compound	Drug administration	Reference
Victim drugs	Atomoxetine	40 mg b.i.d. po	[128]
	Metoprolol	100 mg b.i.d. po	[129]
Perpetrator drugs	Cimetidine	200 mg b.i.d. po	[130]
	Itraconazole	200 mg q.d. po	[131]
	Paroxetine	20 mg q.d. po	[132]
	Quinidine	400 mg q.i.d. po	[133]

b.i.d.: twice daily, po: oral, q.d.: once daily, q.i.d.: four times daily. If perpetrator or victim drugs are clinically applied in form of salts, the respective dose of base was calculated and incorporated in simulations.

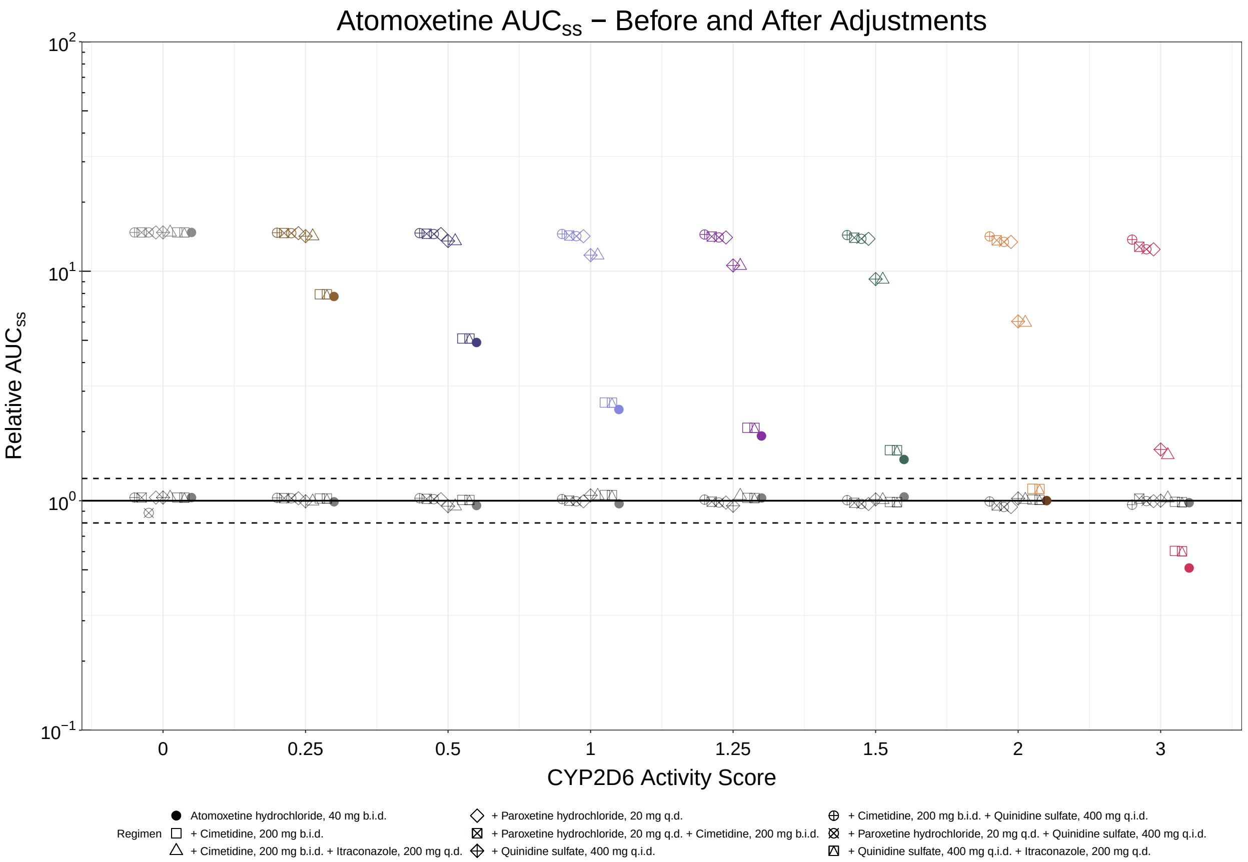

Figure S68: Dose adjustments for atomoxetine using the matching exposure strategy. Colored symbols indicate the AUC_{ss} before dose adjustments for the respective D(D)GI scenario. The corresponding black symbols indicate the matched AUC_{ss} after atomoxetine dose adjustments. Solid black lines show the respective reference AUC_{ss} (activity score 2, no DDI) and dashed black lines mark 80% and 125% of the reference AUC_{ss}. AUC_{ss}: area under the concentration–time curve during steady state, b.i.d.: twice a day, CYP: cytochrome P450, D(D)GI: drug(-drug)-gene interaction, q.d.: once daily, q.i.d.: four times a day.

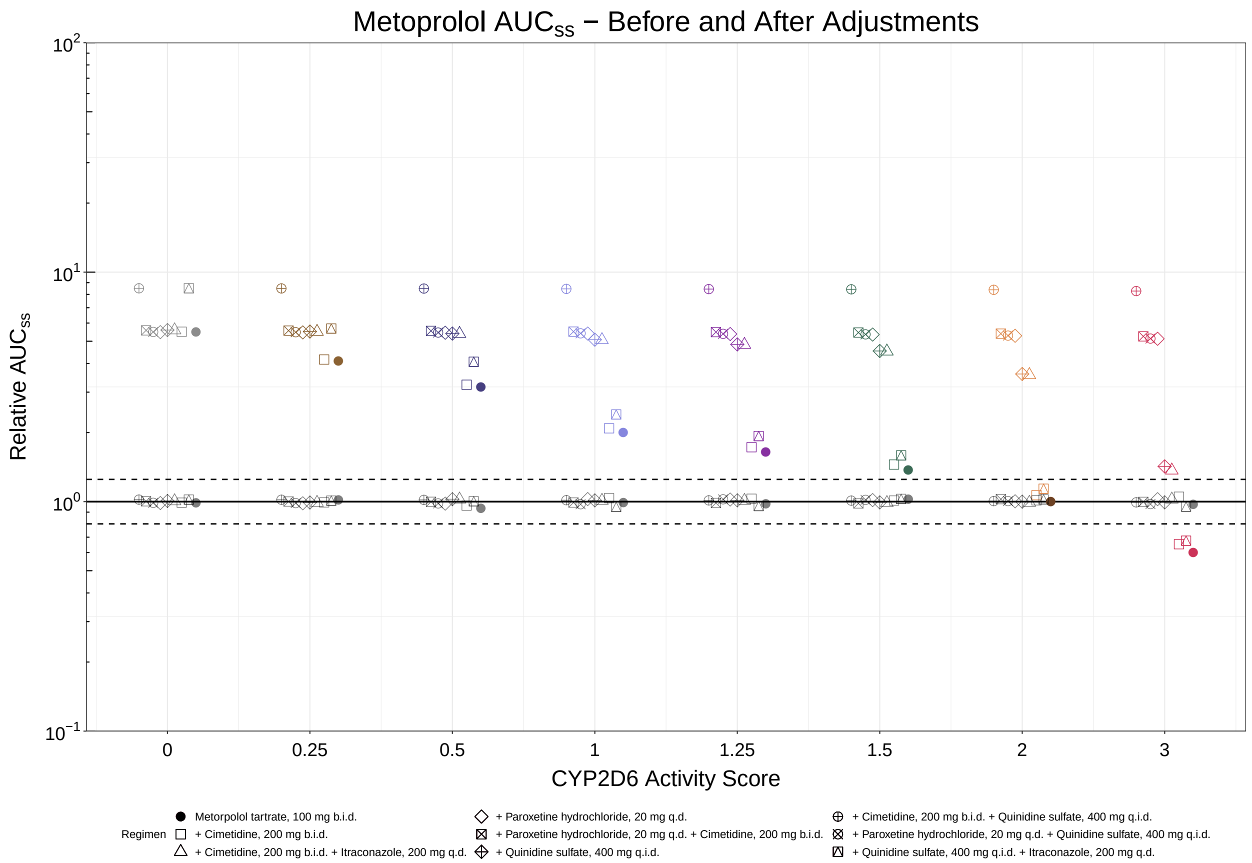

Figure S69: Dose adjustments for metoprolol using the matching exposure strategy. Colored symbols indicate the AUC$_{ss}$ before dose adjustments for the respective D(D)GI scenario. The corresponding black symbols indicate the matched AUC$_{ss}$ after metoprolol dose adjustments. Solid black lines show the respective reference AUC$_{ss}$ (activity score 2, no DDI) and dashed black lines mark 80% and 125% of the reference AUC$_{ss}$. AUC$_{ss}$: area under the concentration–time curve during steady state, b.i.d.: twice a day, CYP: cytochrome P450, D(D)GI: drug(-drug)-gene interaction, q.d.: once daily, q.i.d.: four times a day.

References

[1] S. Rüdesheim, D. Selzer, T. Mürdter, S. Igel, R. Kerb, M. Schwab, and T. Lehr. Physiologically Based Pharmacokinetic Modeling to Describe the CYP2D6 Activity Score-Dependent Metabolism of Paroxetine, Atomoxetine and Risperidone. *Pharmaceutics*, 14(8), 2022. doi: 10.3390/pharmaceutics14081734.

[2] E. J. Guest, L. Aarons, J. B. Houston, A. Rostami-Hodjegan, and A. Galetin. Critique of the two-fold measure of prediction success for ratios: application for the assessment of drug-drug interactions. *Drug metabolism and disposition: the biological fate of chemicals*, 39(2):170–3, 2011. doi: 10.1124/dmd.110.036103.

[3] ICRP Publication 89. Third National Health and Nutrition Examination Survey (NHANES III). *Annals of the ICRP*, 3–4(32):5–265, 2014.

[4] K. Brøsen and L. F. Gram. First-pass metabolism of imipramine and desipramine: Impact of the sparteine oxidation phenotype. *Clinical Pharmacology and Therapeutics*, 43(4):400–406, 1988. doi: 10.1038/clpt.1988.50.

[5] E. Spina, E. Steiner, Ö. Ericsson, and F. Sjöqvist. Hydroxylation of desmethylimipramine: Dependence on the debrisoquin hydroxylation phenotype. *Clinical Pharmacology and Therapeutics*, 41(3):314–319, 1987. doi: 10.1038/clpt.1987.33.

[6] R. E. Aarnoutse, J. Kleinnijenhuis, P. P. Koopmans, D. J. Touw, J. Wieling, Y. A. Hekster, and D. M. Burger. Effect of low-dose ritonavir (100 mg twice daily) on the activity of cytochrome P450 2D6 in healthy volunteers. *Clinical pharmacology and therapeutics*, 78(6):664–74, 2005. doi: 10.1016/j.clpt.2005.09.001.

[7] National Center for Health Statistics Hyattsville, MS 20782. Basic anatomical and physiological data for use in radiological protection: reference values. A report of age- and gender-related differences in the anatomical and physiological characteristics of reference individuals. 1997.

[8] R. F. Bergstrom, A. L. Peyton, and L. Lemberger. Quantification and mechanism of the fluoxetine and tricyclic antidepressant interaction. *Clinical pharmacology and therapeutics*, 51(3):239–48, 1992. doi: 10.1038/clpt.1992.18.

[9] J. Boni, R. Abbas, C. Leister, J. Burns, R. Jordan, M. Hoffmann, W. DeMaio, and B. Hug. Disposition of desipramine, a sensitive cytochrome P450 2D6 substrate, when coadministered with intravenous temsirolimus. *Cancer chemotherapy and pharmacology*, 64(2):263–70, 2009. doi: 10.1007/s00280-008-0865-9.

[10] R. Z. Harris, M. Salfi, E. Posvar, D. Hoelscher, and D. Padhi. Pharmacokinetics of desipramine HCl when administered with cinacalcet HCl. *European journal of clinical pharmacology*, 63(2):159–63, 2007. doi: 10.1007/s00228-006-0129-8.

[11] S. M. Hynes, E. Wickremsinhe, W. Zhang, R. Decker, J. Ott, J. Chandler, and M. Mitchell. Evaluation of the likelihood of a selective CHK1 inhibitor (LY2603618) to inhibit CYP2D6 with desipramine as a probe substrate in cancer patients. *Biopharmaceutics & Drug Disposition*, 36(1):49–63, 2015. doi: 10.1002/bdd.1922.

[12] S. Madani, D. Barilla, J. Cramer, Y. Wang, and C. Paul. Effect of terbinafine on the pharmacokinetics and pharmacodynamics of desipramine in healthy volunteers identified as cytochrome P450 2D6 (CYP2D6) extensive metabolizers. *Journal of clinical pharmacology*, 42(11):1211–8, 2002. doi: 10.1177/009127002762491299.

[13] A. I. Nichols, P. Fatato, M. Shenouda, J. Paul, J. A. Isler, R. D. Pedersen, Q. Jiang, S. Ahmed, and A. Patroneva. The effects of desvenlafaxine and paroxetine on the pharmacokinetics of the cytochrome P450 2D6 substrate desipramine in healthy adults. *Journal of clinical pharmacology*, 49(2):219–28, 2009. doi: 10.1177/0091270008326716.

[14] A. I. Nichols, A. Madelyn, Y. Chen, J. A. Behrle, G. Frick, and J. Paul. Effects of desvenlafaxine on the pharmacokinetics of desipramine in healthy adults. *International Clinical Psychopharmacology*, 28(2):99–105, 2013. doi: 10.1097/YIC.0b013e32835c1f49.

[15] A. Patroneva, S. M. Connolly, P. Fatato, R. Pedersen, Q. Jiang, J. Paul, C. Guico-Pabia, J. A. Isler, M. E. Burczynski, and A. I. Nichols. An assessment of drug-drug interactions: the effect of desvenlafaxine and duloxetine on the pharmacokinetics of the CYP2D6 probe desipramine in healthy subjects. *Drug metabolism and disposition: the biological fate of chemicals*, 36(12):2484–2491, 2008. doi: 10.1124/dmd.108.021527.

[16] M. J. Reese, R. M. Wurm, K. T. Muir, G. T. Generaux, L. St John-Williams, and D. J. McConn. An in vitro mechanistic study to elucidate the desipramine/bupropion clinical drug-drug interaction. *Drug metabolism and disposition: the biological fate of chemicals*, 36(7):1198–201, 2008. doi: 10.1124/dmd.107.020198.

[17] J.-M. Sauer, A. J. Long, B. Ring, J. S. Gillespie, N. P. Sanburn, K. A. DeSante, D. Petullo, M. R. VandenBranden, C. B. Jensen, S. A. Wrighton, B. P. Smith, H. A. Read, and J. W. Witcher. Atomoxetine Hydrochloride: Clinical Drug-Drug Interaction Prediction and Outcome. *Journal of Pharmacology and Experimental Therapeutics*, 308(2): 410–418, 2004. doi: 10.1124/jpet.103.058727.

[18] M. H. Skinner, H.-Y. Kuan, A. Pan, K. Sathirakul, M. P. Knadler, C. R. Gonzales, K. P. Yeo, S. Reddy, M. Lim, M. Ayan-Oshodi, and S. D. Wise. Duloxetine is both an inhibitor and a substrate of cytochrome P4502D6 in healthy volunteers. *Clinical pharmacology and therapeutics*, 73(3):170–7, 2003. doi: 10.1067/mcp.2003.28.

[19] J. Alderman, S. H. Preskorn, D. J. Greenblatt, W. Harrison, D. Penenberg, J. Allison, and M. Chung. Desipramine pharmacokinetics when coadministered with paroxetine or sertraline in extensive metabolizers. *Journal of clinical psychopharmacology*, 17(4):284–91, 1997. doi: 10.1097/00004714-199708000-00008.

[20] T. K. Bergmann, L. Bathum, and K. Brosen. Duplication of CYP2D6 predicts high clearance of desipramine but high clearance does not predict duplication of CYP2D6. *European journal of clinical pharmacology*, 57(2):123–7, 2001. doi: 10.1007/s002280100284.

[21] K. Brøsen, S. Victoria Otton, and L. F. Gram. Imipramine demethylation and hydroxylation: Impact of the sparteine oxidation phenotype. *Clinical Pharmacology and Therapeutics*, 40(5):543–549, 1986. doi: 10.1038/clpt.1986.221.

[22] K. Brøsen, J. G. Hansen, K. K. Nielsen, S. H. Sindrup, and L. F. Gram. Inhibition by paroxetine of desipramine metabolism in extensive but not in poor metabolizers of sparteine. *European journal of clinical pharmacology*, 44 (4):349–55, 1993. doi: 10.1007/BF00316471.

[23] K. Brøsen and L. F. Gram. Quinidine inhibits the 2-hydroxylation of imipramine and desipramine but not the demethylation of imipramine. *European Journal of Clinical Pharmacology*, 37(2):155–160, 1989. doi: 10.1007/BF00558224.

[24] E. Spina, A. Avenoso, G. M. Campo, A. P. Caputi, and E. Perucca. The effect of carbamazepine on the 2-hydroxylation of desipramine. *Psychopharmacology*, 117(4):413–6, 1995. doi: 10.1007/BF02246212.

[25] S. Kim, J. Chen, T. Cheng, A. Gindulyte, J. He, S. He, Q. Li, B. A. Shoemaker, P. A. Thiessen, B. Yu, L. Zaslavsky, J. Zhang, and E. E. Bolton. PubChem 2019 update: improved access to chemical data. *Nucleic acids research*, 47(D1):D1102–D1109, 2019.

[26] ChemAxon. Chemicalize, 2023. URL https://chemicalize.com.

[27] D. S. Wishart, C. Knox, A. C. Guo, S. Shrivastava, M. Hassanali, P. Stothard, Z. Chang, and J. Woolsey. DrugBank: a comprehensive resource for in silico drug discovery and exploration. *Nucleic acids research*, 34(Database issue): D668–72, 2006. doi: 10.1093/nar/gkj067.

[28] R. Watanabe, T. Esaki, H. Kawashima, Y. Natsume-Kitatani, C. Nagao, R. Ohashi, and K. Mizuguchi. Predicting Fraction Unbound in Human Plasma from Chemical Structure: Improved Accuracy in the Low Value Ranges. *Molecular Pharmaceutics*, 15(11):5302–5311, 2018. doi: 10.1021/acs.molpharmaceut.8b00785.

[29] S. E. Ball, D. Ahern, J. Scatina, and J. Kao. Venlafaxine: In vitro inhibition of CYP2D6 dependent imipramine and desipramine metabolism; comparative studies with selected SSRIs, and effects on human hepatic CYP3A4, CYP2C9 and CYP1A2. *British Journal of Clinical Pharmacology*, 43(6):619–626, 1997. doi: 10.1046/j.1365-2125.1997.00591.x.

[30] R. P. Austin, P. Barton, S. L. Cockroft, M. C. Wenlock, and R. J. Riley. The influence of nonspecific microsomal binding on apparent intrinsic clearance, and its prediction from physicochemical properties. *Drug Metabolism and Disposition*, 30(12):1497–1503, 2002. doi: 10.1124/dmd.30.12.1497.

[31] T. Rodgers, D. Leahy, and M. Rowland. Physiologically based pharmacokinetic modeling 1: Predicting the tissue distribution of moderate-to-strong bases. *Journal of Pharmaceutical Sciences*, 94(6):1259–1276, 2005. doi: 10.1002/jps.20322.

[32] T. Rodgers and M. Rowland. Physiologically based pharmacokinetic modelling 2: Predicting the tissue distribution of acids, very weak bases, neutrals and zwitterions. *Journal of Pharmaceutical Sciences*, 95(6):1238–57, 2006. doi: 10.1002/jps.20502.

[33] Open Systems Pharmacology Suite Community. Open Systems Pharmacology Documentation., 2018. URL https://docs.open-systems-pharmacology.org/working-with-pk-sim/pk-sim-documentation.

[34] M. Nishimura and S. Naito. Tissue-specific mRNA expression profiles of human phase I metabolizing enzymes except for cytochrome P450 and phase II metabolizing enzymes. *Drug metabolism and pharmacokinetics*, 21(5): 357–74, 2006. doi: 10.2133/dmpk.21.357.

[35] M. Meyer, S. Schneckener, B. Ludewig, L. Kuepfer, and J. Lippert. Using expression data for quantification of active processes in physiologically based pharmacokinetic modeling. *Drug metabolism and disposition: the biological fate of chemicals*, 40(5):892–901, 2012. doi: 10.1124/dmd.111.043174.

[36] M. Nishimura, H. Yaguti, H. Yoshitsugu, S. Naito, and T. Satoh. Tissue distribution of mRNA expression of human cytochrome P450 isoforms assessed by high-sensitivity real-time reverse transcription PCR. *Journal of the Pharmaceutical Society of Japan*, 123(5):369–75, 2003. doi: 10.1248/yakushi.123.369.

[37] A. D. Rodrigues. Integrated cytochrome P450 reaction phenotyping: attempting to bridge the gap between cDNA-expressed cytochromes P450 and native human liver microsomes. *Biochemical pharmacology*, 57(5): 465–80, 1999. doi: 10.1016/S0006-2952(98)00268-8.

[38] Open Systems Pharmacology Suite Community. Open Systems Pharmacology Suite Manual, 2018. URL https://docs.open-systems-pharmacology.org/.

[39] N. Kolesnikov, E. Hastings, M. Keays, O. Melnichuk, Y. A. Tang, E. Williams, M. Dylag, N. Kurbatova, M. Brandizi, T. Burdett, K. Megy, E. Pilicheva, G. Rustici, A. Tikhonov, H. Parkinson, R. Petryszak, U. Sarkans, and A. Brazma. ArrayExpress update-simplifying data submissions. *Nucleic Acids Research*, 43(D1):D1113–D1116, 2015. doi: 10.1093/nar/gku1057.

[40] B. Achour, M. R. Russell, J. Barber, and A. Rostami-Hodjegan. Simultaneous quantification of the abundance of several cytochrome P450 and uridine 5'-diphospho-glucuronosyltransferase enzymes in human liver microsomes using multiplexed targeted proteomics. *Drug metabolism and disposition: the biological fate of chemicals*, 42(4): 500–510, 2014. doi: 10.1124/dmd.113.055632.

[41] D. Scotcher, S. Billington, J. Brown, C. R. Jones, C. D. Brown, A. Rostami-Hodjegan, and A. Galetin. Microsomal and cytosolic scaling factors in dog and human kidney cortex and application for in vitro-in vivo extrapolation of renal metabolic clearance. *Drug Metabolism and Disposition*, 45(5):556–568, 2017. doi: 10.1124/dmd.117.075242.

[42] National Center for Biotechnology Information (NCBI). Expressed Sequence Tags (EST) from UniGene, 2019.

[43] G. Margaillan, M. Rouleau, J. K. Fallon, P. Caron, L. Villeneuve, V. Turcotte, P. C. Smith, M. S. Joy, and C. Guillemette. Quantitative profiling of human renal UDP-glucuronosyltransferases and glucuronidation activity: A comparison of normal and tumoral kidney tissues. *Drug Metabolism and Disposition*, 43(4):611–19, 2015. doi: 10.1124/dmd.114.062877.

[44] M. Otsuka, T. Matsumoto, R. Morimoto, S. Arioka, H. Omote, and Y. Moriyama. A human transporter protein that mediates the final excretion step for toxic organic cations. *Proceedings of the National Academy of Sciences of the United States of America*, 102(50):17923–8, 2005. doi: 10.1073/pnas.0506483102.

[45] S. Masuda, T. Terada, A. Yonezawa, Y. Tanihara, K. Kishimoto, T. Katsura, O. Ogawa, and K.-i. Inui. Identification and functional characterization of a new human kidney-specific H+/organic cation antiporter, kidney-specific multidrug and toxin extrusion 2. *Journal of the American Society of Nephrology : JASN*, 17(8):2127–35, 2006. doi: 10.1681/ASN.2006030205.

[46] B. Prasad, K. Johnson, S. Billington, C. Lee, G. W. Chung, C. D. Brown, E. J. Kelly, J. Himmelfarb, and J. D. Unadkat. Abundance of drug transporters in the human kidney cortex as quantified by quantitative targeted proteomics. *Drug Metabolism and Disposition*, 44(12):1920–1924, 2016. doi: 10.1124/dmd.116.072066.

[47] M. Nishimura and S. Naito. Tissue-specific mRNA expression profiles of human ATP-binding cassette and solute carrier transporter superfamilies. *Drug metabolism and pharmacokinetics*, 20(6):452–77, 2005. doi: 10.2133/dmpk.20.452.

[48] B. Prasad, R. Evers, A. Gupta, C. E. C. A. Hop, L. Salphati, S. Shukla, S. V. Ambudkar, and J. D. Unadkat. Interindividual variability in hepatic organic anion - transporting polypeptides and P-glycoprotein (ABCB1) protein expression: quantification by liquid chromatography tandem mass spectroscopy and influence of genotype, age, and sex. *Drug metabolism and disposition: the biological fate of chemicals*, 42(1):78–88, 2014. doi: 10.1124/dmd.113.053819.

[49] L. Wang, B. Prasad, L. Salphati, X. Chu, A. Gupta, C. E. Hop, R. Evers, and J. D. Unadkat. Interspecies variability in expression of hepatobiliary transporters across human, dog, monkey, and rat as determined by quantitative proteomics. *Drug Metabolism and Disposition*, 43(3):367–74, 2015. doi: 10.1124/dmd.114.061580.

[50] N. Hanke, S. Frechen, D. Moj, H. Britz, T. Eissing, T. Wendl, and T. Lehr. PBPK models for CYP3A4 and P-gp DDI prediction: A modeling network of rifampicin, itraconazole, clarithromycin, midazolam, alfentanil, and digoxin. *CPT: Pharmacometrics & Systems Pharmacology*, 7(10):647–59, 2018. doi: 10.1002/psp4.12343.

[51] Midazolam OSP Repository, 2023. URL https://github.com/Open-Systems-Pharmacology/Midazolam-Model.

[52] K. Rowland Yeo, R. L. Walsky, M. Jamei, A. Rostami-Hodjegan, and G. T. Tucker. Prediction of time-dependent CYP3A4 drug-drug interactions by physiologically based pharmacokinetic modelling: Impact of inactivation parameters and enzyme turnover. *European Journal of Pharmaceutical Sciences*, 43(3):160–73, 2011. doi: 10.1016/j.ejps.2011.04.008.

[53] D. J. Greenblatt, L. L. von Moltke, J. S. Harmatz, G. Chen, J. L. Weemhoff, C. Jen, C. J. Kelley, B. W. LeDuc, and M. A. Zinny. Time course of recovery of cytochrome P450 3A function after single doses of grapefruit juice. *Clinical Pharmacology and Therapeutics*, 74(2):121–29, 2003. doi: 10.1016/S0009-9236(03)00118-8.

[54] T. Kanacher, A. Lindauer, E. Mezzalana, I. Michon, C. Veau, J. D. G. Mantilla, V. Nock, and A. Fleury. A Physiologically-Based Pharmacokinetic (PBPK) Model Network for the Prediction of CYP1A2 and CYP2C19 Drug-Drug-Gene Interactions with Fluvoxamine, Omeprazole, S-mephenytoin, Moclobemide, Tizanidine, Mexiletine, Ethinylestradiol, and Caffeine. *Pharmaceutics*, 12(12):1–15, 2020. doi: 10.3390/pharmaceutics12121191.

[55] F. Z. Marok, L. M. Fuhr, N. Hanke, D. Selzer, and T. Lehr. Physiologically Based Pharmacokinetic Modeling of Bupropion and Its Metabolites in a CYP2B6 Drug-Drug-Gene Interaction Network. *Pharmaceutics*, 13(3), 2021. doi: 10.3390/pharmaceutics13030331.

[56] J. E. Sager, S. Tripathy, L. S. Price, A. Nath, J. Chang, A. Stephenson-Famy, and N. Isoherranen. In vitro to in vivo extrapolation of the complex drug-drug interaction of bupropion and its metabolites with CYP2D6; simultaneous reversible inhibition and CYP2D6 downregulation. *Biochemical Pharmacology*, 123:85–96, 2017. ISSN 18732968. doi: 10.1016/j.bcp.2016.11.007. URL http://dx.doi.org/10.1016/j.bcp.2016.11.007.

[57] J. E. Sager, S. Tripathy, L. S. Price, A. Nath, J. Chang, A. Stephenson-Famy, and N. Isoherranen. Corrigendum to “In vitro to in vivo extrapolation of the complex drug-drug interaction of bupropion and its metabolites with CYP2D6; simultaneous reversible inhibition and CYP2D6 downregulation” [Biochem. Pharmacol. 123(2017)85–96] (Biochemical Pharmacolo. *Biochemical Pharmacology*, 183(November 2020):114306, 2021. ISSN 18732968. doi: 10.1016/j.bcp.2020.114306. URL https://doi.org/10.1016/j.bcp.2020.114306.

[58] L. M. Fuhr, F. Z. Marok, N. Hanke, D. Selzer, and T. Lehr. Pharmacokinetics of the CYP3A4 and CYP2B6 Inducer Carbamazepine and Its Drug-Drug Interaction Potential: A Physiologically Based Pharmacokinetic Modeling Approach. *Pharmaceutics*, 13(2), 2021. doi: 10.3390/pharmaceutics13020270.

[59] N. Hanke, D. Türk, D. Selzer, N. Ishiguro, T. Ebner, S. Wiebe, F. Müller, P. Stopfer, V. Nock, and T. Lehr. A Comprehensive Whole-Body Physiologically Based Pharmacokinetic Drug-Drug-Gene Interaction Model of Metformin and Cimetidine in Healthy Adults and Renally Impaired Individuals. *Clinical pharmacokinetics*, 59(11): 1419–1431, 2020. doi: 10.1007/s40262-020-00896-w.

[60] M. Madeira, M. Levine, T. K. H. Chang, A. Mirfazaelian, and G. D. Bellward. The effect of cimetidine on

dextromethorphan O-demethylase activity of human liver microsomes and recombinant CYP2D6. *Drug metabolism and disposition: the biological fate of chemicals*, 32(4):460–7, 2004. doi: 10.1124/dmd.32.4.460.

[61] H. Britz, N. Hanke, A.-K. Volz, O. Spigset, M. Schwab, T. Eissing, T. Wendl, S. Frechen, and T. Lehr. Physiologically-Based Pharmacokinetic Models for CYP1A2 Drug-Drug Interaction Prediction: A Modeling Network of Fluvoxamine, Theophylline, Caffeine, Rifampicin, and Midazolam. *CPT: pharmacometrics & systems pharmacology*, 8(5):296–307, 2019. doi: 10.1002/psp4.12397.

[62] H. K. Crewe, M. S. Lennard, G. T. Tucker, F. R. Woods, and R. E. Haddock. The effect of selective serotonin re-uptake inhibitors on cytochrome P4502D6 (CYP2D6) activity in human liver microsomes. *British journal of clinical pharmacology*, 34(3):262–5, sep 1992. ISSN 0306-5251. doi: 10.1111/j.1365-2125.1992.tb04134.x. URL https://bpspubs.onlinelibrary.wiley.com/doi/10.1111/j.1365-2125.1992.tb04134.xhttp://www.ncbi.nlm.nih.gov/pubmed/1389951http://www.pubmedcentral.nih.gov/articlerender.fcgi?artid=PMC1381398.

[63] K. Iga. Dynamic and Static Simulations of Fluvoxamine-Perpetrated Drug-Drug Interactions Using Multiple Cytochrome P450 Inhibition Modeling, and Determination of Perpetrator-Specific CYP Isoform Inhibition Constants and Fractional CYP Isoform Contributions to Victim Clearance. *Journal of Pharmaceutical Sciences*, 105(3):1307–1317, mar 2016. ISSN 0022-3549. doi: 10.1016/J.XPHS.2015.11.044.

[64] F. Z. Marok, J.-G. Wojtyniak, L. M. Fuhr, D. Selzer, M. Schwab, J. Weiss, W. E. Haefeli, and T. Lehr. A Physiologically Based Pharmacokinetic Model of Ketoconazole and Its Metabolites as Drug-Drug Interaction Perpetrators. *Pharmaceutics*, 15(2), 2023. doi: 10.3390/pharmaceutics15020679.

[65] X. Q. Li, T. B. Andersson, M. Ahlström, and L. Weidolf. Comparison of inhibitory effects of the proton pump-inhibiting drugs omeprazole, esomeprazole, lansoprazole, pantoprazole, and rabeprazole on human cytochrome P450 activities. *Drug Metabolism and Disposition*, 32(8):821–827, 2004. doi: 10.1124/dmd.32.8.821.

[66] D. Feick, S. Rüdesheim, F. Z. Marok, D. Selzer, H. L. H. Loer, D. Teutonico, S. Frechen, M. van der Lee, D. J. A. R. Moes, J. J. Swen, M. Schwab, and T. Lehr. Physiologically Based Pharmacokinetic Modeling of Quinidine to Establish a CYP3A4, P-gp and CYP2D6 Drug-Drug-Gene Interaction Network. *CPT: pharmacometrics & systems pharmacology*, 2023. doi: 10.1002/psp4.12981.

[67] N. Hanke, D. Türk, D. Selzer, S. Wiebe, É. Fernandez, P. Stopfer, V. Nock, and T. Lehr. A Mechanistic, Enantioselective, Physiologically Based Pharmacokinetic Model of Verapamil and Norverapamil, Built and Evaluated for Drug-Drug Interaction Studies. *Pharmaceutics*, 12(6), 2020. doi: 10.3390/pharmaceutics12060556.

[68] C. Kovar, L. Kovar, S. Rüdesheim, D. Selzer, B. Ganchev, P. Kröner, S. Igel, R. Kerb, E. Schaeffeler, T. E. Mürdter, M. Schwab, and T. Lehr. Prediction of Drug-Drug-Gene Interaction Scenarios of (E)-Clomiphene and Its Metabolites Using Physiologically Based Pharmacokinetic Modeling. *Pharmaceutics*, 14(12), 2022. doi: 10.3390/pharmaceutics14122604.

[69] S. Rüdesheim, D. Selzer, U. Fuhr, M. Schwab, and T. Lehr. Physiologically-based pharmacokinetic modeling of dextromethorphan to investigate interindividual variability within CYP2D6 activity score groups. *CPT: pharmacometrics & systems pharmacology*, 11(4):494–511, 2022. doi: 10.1002/psp4.12776.

[70] S. Rüdesheim, J.-G. Wojtyniak, D. Selzer, N. Hanke, F. Mahfoud, M. Schwab, and T. Lehr. Physiologically Based Pharmacokinetic Modeling of Metoprolol Enantiomers and α-Hydroxymetoprolol to Describe CYP2D6 Drug-Gene Interactions. *Pharmaceutics*, 12(12), 2020. doi: 10.3390/pharmaceutics12121200.

[71] E. H. Jung, Y. J. Lee, D.-H. Kim, P. Kang, C. W. Lim, C.-K. Cho, C.-G. Jang, S.-Y. Lee, and J.-W. Bae. Effects of paroxetine on the pharmacokinetics of atomoxetine and its metabolites in different CYP2D6 genotypes. *Archives of pharmacal research*, 43(12):1356–1363, 2020. doi: 10.1007/s12272-020-01300-8.

[72] S. H. Kim, J. Y. Byeon, Y. H. Kim, C. M. Lee, Y. J. Lee, C. G. Jang, and S. Y. Lee. Physiologically based pharmacokinetic modelling of atomoxetine with regard to CYP2D6 genotypes. *Scientific Reports*, 8(1):12405, 2018. ISSN 20452322. doi: 10.1038/s41598-018-30841-8. URL http://dx.doi.org/10.1038/s41598-018-30841-8.

[73] J. M. Sauer, G. D. Ponsler, E. L. Mattiuz, A. J. Long, J. W. Witcher, H. R. Thomasson, and K. A. Desante. Disposition and metabolic fate of atomoxetine hydrochloride: The role of CYP2D6 in human disposition and metabolism. *Drug Metabolism and Disposition*, 31(1):98–107, 2003. ISSN 00909556. doi: 10.1124/dmd.31.1.98.

[74] I. Todor, A. Popa, M. Neag, D. Muntean, C. Bocsan, A. Buzoianu, L. Vlase, A. M. Gheldiu, and C. Briciu. Evaluation of a potential metabolism-mediated drug-drug interaction between atomoxetine and bupropion in healthy volunteers. *Journal of Pharmacy and Pharmaceutical Sciences*, 19(2):198–207, 2016. doi: 10.18433/J3H03R.

[75] J. Y. Byeon, Y. H. Kim, H. S. Na, J. H. Jang, S. H. Kim, Y. J. Lee, J. W. Bae, I. S. Kim, C. G. Jang, M. W. Chung, and S. Y. Lee. Effects of the CYP2-star allele on the pharmacokinetics of atomoxetine and its metabolites. *Archives of Pharmacal Research*, 38(11):2083–2091, 2015. ISSN 19763786. doi: 10.1007/s12272-015-0646-z.

[76] T. Mürdter. Impact of CYP2D6 genotype and co-medication with paroxetine and clarithromycin on clomiphene metabolism in vivo. *Abstracts of the 82nd Annual Meeting of the German Society for Experimental and Clinical Pharmacology and Toxicology (DGPT) in Naunyn- Schmiedeberg's Archives of Pharmacology*, 2016.

[77] F. Storelli, A. Matthey, S. Lenglet, A. Thomas, J. Desmeules, and Y. Daali. Impact of CYP2D6 Functional Allelic Variations on Phenoconversion and Drug–Drug Interactions. *Clinical Pharmacology and Therapeutics*, 104(1): 148–157, 2018. doi: 10.1002/cpt.889.

[78] F. Qiu, S. Liu, P. Miao, J. Zeng, L. Zhu, T. F. Zhao, Y. Ye, and J. Jiang. Effects of the Chinese herbal formula "Zuojin Pill" on the pharmacokinetics of dextromethorphan in healthy Chinese volunteers with CYP2D6*10 genotype. *European Journal of Clinical Pharmacology*, 72(6):689–695, 2016. ISSN 14321041. doi: 10.1007/s00228-016-2048-7.

[79] Pharmacokinetic effects of isavuconazole coadministration with the cytochrome P450 enzyme substrates bupropion, repaglinide, caffeine, dextromethorphan, and methadone in healthy subjects. *Clinical Pharmacology in Drug Development*, 6(1):54–65, 2017. ISSN 21607648. doi: 10.1002/cpdd.281.

[80] D. A. Capon, F. Bochner, N. Kerry, G. Mikus, C. Danz, and A. A. Somogyi. The influence of CYP2D6 polymorphism and quinidine on the disposition and antitussive effect of dextromethorphan in humans. *Clinical Pharmacology and Therapeutics*, 60(3):295–307, 1996. doi: 10.1016/S0009-9236(96)90056-9.

[81] J. C. Gorski, S. M. Huang, A. Pinto, M. A. Hamman, J. K. Hilligoss, N. A. Zaheer, M. Desai, M. Miller, and S. D. Hall. The effect of echinacea (Echinacea purpurea root) on cytochrome P450 activity in vivo. *Clinical Pharmacology and Therapeutics*, 75(1):89–100, 2004. ISSN 00099236. doi: 10.1016/j.clpt.2003.09.013.

[82] T. D. Leemann, K. P. Devi, and P. Dayer. Similar effect of oxidation deficiency (debrisoquine polymorphism) and quinidine on the apparent volume of distribution of (+/-)-metoprolol. *European journal of clinical pharmacology*, 45 (1):65–71, 1993. doi: 10.1007/BF00315352.

[83] S. H. Bae, J. K. Lee, D. Y. Cho, and S. K. Bae. Simultaneous determination of metoprolol and its metabolites, α-hydroxymetoprolol and O-desmethylmetoprolol, in human plasma by liquid chromatography with tandem mass spectrometry: Application to the pharmacokinetics of metoprolol associated with CYP2D6 genotypes. *Journal of Separation Science*, 37(11):1256–1264, 2014. ISSN 16159314. doi: 10.1002/jssc.201301353.

[84] S. K. Jin, H. J. Chung, M. W. Chung, J. I. Kim, J. H. Kang, S. W. Woo, S. Bang, S. H. Lee, H. J. Lee, and J. Roh. Influence of CYP2D6*10 on the pharmacokinetics of metoprolol in healthy Korean volunteers. *Journal of Clinical Pharmacy and Therapeutics*, 33(5):567–573, 2008. ISSN 02694727. doi: 10.1111/j.1365-2710.2008.00945.x.

[85] B. A. Hamelin, A. Bouayad, J. Méthot, J. Jobin, P. Desgagnés, P. Poirier, J. Allaire, J. Dumesnil, and J. Turgeon. Significant interaction between the nonprescription antihistamine diphenhydramine and the CYP2D6 substrate metoprolol in healthy men with high or low CYP2D6 activity. *Clinical Pharmacology and Therapeutics*, 67(5): 466–477, 2000. ISSN 00099236. doi: 10.1067/mcp.2000.106464.

[86] A. Sharma, P. Pibarot, S. Pilote, J. G. Dumesnil, M. Arsenault, P. M. Bélanger, B. Meibohm, and B. A. Hamelin. Modulation of metoprolol pharmacokinetics and hemodynamics by diphenhydramine coadministration during exercise testing in healthy premenopausal women. *The Journal of pharmacology and experimental therapeutics*, 313(3):1172–1181, 2005. doi: 10.1124/jpet.104.081109.

[87] J. D. Huang, S. K. Chuang, C. L. Cheng, and M. L. Lai. Pharmacokinetics of metoprolol enantiomers in Chinese subjects of major CΥP2D6 genotypes. *Clinical Pharmacology and Therapeutics*, 65(4):402–407, 1999. ISSN 00099236. doi: 10.1016/S0009-9236(99)70134-7.

[88] A. Seeringer, J. Brockmöller, S. Bauer, and J. Kirchheiner. Enantiospecific pharmacokinetics of metoprolol in CYP2D6 ultra-rapid metabolizers and correlation with exercise-induced heart rate. *European Journal of Clinical Pharmacology*, 64(9):883–888, 2008. ISSN 00316970. doi: 10.1007/s00228-008-0504-8.

[89] L. Labbé, G. O'Hara, M. Lefebvre, É. Lessard, M. Gilbert, A. Adedoyin, J. Champagne, B. Hamelin, and J. Turgeon. Pharmacokinetic and pharmacodynamic interaction be mexiletine and propafenone in human beings. *Clinical Pharmacology and Therapeutics*, 68(1):44–57, 2000. ISSN 00099236. doi: 10.1067/mcp.2000.108023.

[90] Z. Abolfathi, C. Fiset, M. Gilbert, K. Moerike, P. M. Belanger, and J. Turgeon. Role of polymorphic debrisoquin 4-hydroxylase activity in the stereoselective disposition of mexiletine in humans. *Journal of Pharmacology and Experimental Therapeutics*, 266(3):1196–1201, 1993.

[91] R. Chen, H. Wang, J. Shi, K. Shen, and P. Hu. Cytochrome P450 2D6 genotype affects the pharmacokinetics of controlled-release paroxetine in healthy Chinese subjects: comparison of traditional phenotype and activity score systems. *European Journal of Clinical Pharmacology*, 71(7):835–841, 2015. doi: 10.1007/s00228-015-1855-6.

[92] S. H. Sindrup, K. Brøsen, L. F. Gram, J. Hallas, E. Skjelbo, A. Allen, G. D. Allen, S. M. Cooper, G. Mellows, and T. C. Tasker. The relationship between paroxetine and the sparteine oxidation polymorphism. *Clinical Pharmacology & Therapeutics*, 51(3):278–287, 1992. doi: 10.1038/clpt.1992.23.

[93] Y. R. Yoon, I. J. Cha, J. H. Shon, K. A. Kim, Y. N. Cha, I. J. Jang, C. W. Park, S. G. Shin, D. A. Flockhart, and J. G. Shin. Relationship of paroxetine disposition to metoprolol metabolic ratio and CYP2D6*10 genotype of Korean subjects. *Clinical Pharmacology & Therapeutics*, 67(5):567–576, 2000. doi: 10.1067/mcp.2000.106128.

[94] J. Novalbos, R. López-Rodríguez, M. Román, S. Gallego-Sadín, D. Ochoa, and F. Abad-Santos. Effects of CYP2D6 genotype on the pharmacokinetics, pharmacodynamics, and safety of risperidone in healthy volunteers. *Journal of Clinical Psychopharmacology*, 30(5):504–511, 2010. doi: 10.1097/JCP.0b013e3181ee84c7.

[95] G. Bondolfi, C. B. Eap, G. Bertschy, D. Zullino, A. Vermeulen, and P. Baumann. The effect of fluoxetine on the pharmacokinetics and safety of risperidone in psychotic patients. *Pharmacopsychiatry*, 35(2):50–56, 2002. doi: 10.1055/s-2002-25026.

[96] G. Calvo, C. García-Gea, A. Luque, A. Morte, R. Dal-Ré, and M. Barbanoj. Lack of pharmacologic interaction between paroxetine and alprazolam at steady state in healthy volunteers. *Journal of clinical psychopharmacology*, 24(3):268–76, 2004. doi: 10.1097/01.jcp.0000125689.05091.c6.

[97] I. Todor, A. Popa, M. Neag, D. Muntean, C. Bocsan, A. Buzoianu, L. Vlase, A. M. Gheldiu, and C. Briciu. Evaluation of the potential pharmacokinetic interaction between atomoxetine and fluvoxamine in healthy volunteers. *Pharmacology*, 99(1-2):84–88, 2017. doi: 10.1159/000452223.

[98] Tanaka, G and Kawamura, H. Anatomical and physiological characteristics for Asian reference man: Male and female of different ages: Tanaka model. *Division of Radioecology. National Institute of Radiological Sciences. Hitachinaka 311-12 Japan. Report Number NIRS-M-115.*, 1996.

[99] I. Todor, A. Popa, M. Neag, D. Muntean, C. Bocsan, A. Buzoianu, L. Vlase, A. M. Gheldiu, R. Chira, and C. Briciu. The influence of paroxetine on the pharmacokinetics of atomoxetine and its main metabolite. *Clujul Medical*, 88(4): 513–520, 2015. doi: 10.15386/cjmed-488.

[100] D. J. Belle, C. S. Ernest, J.-M. Sauer, B. P. Smith, H. R. Thomasson, and J. W. Witcher. Effect of potent CYP2D6 inhibition by paroxetine on atomoxetine pharmacokinetics. *Journal of clinical pharmacology*, 42(11):1219–27, 2002. doi: 10.1177/009127002762491307.

[101] K. A. Schoedel, L. E. Pope, and E. M. Sellers. Randomized Open-Label Drug-Drug Interaction Trial of Dextromethorphan/Quinidine and Paroxetine in Healthy Volunteers. *Clinical Drug Investigation*, 32(3):157–169, 2012. doi: 10.2165/11599870-000000000-00000.

[102] M. Schadel, D. Wu, S. V. Otton, W. Kalow, and E. M. Sellers. Pharmacokinetics of Dextromethorphan and Metabolites in Humans. *Journal of Clinical Psychopharmacology*, 15(4):263–269, 1995. doi: 10.1097/00004714-199508000-00005.

[103] E. Steiness, S. Waldorff, P. B. Hansen, H. Egebald, J. Buch, and H. Egeblad. Reduction of digoxin-induced inotropism during quinidine administration. *Clinical Pharmacology and Therapeutics*, 27(6):791–795, 1980. doi: 10.1038/clpt.1980.112.

[104] H. R. Ochs, G. Bodem, and D. J. Greenblatt. Impairment of digoxin clearance by coadministration of quinidine. *Journal of clinical pharmacology*, 21(10):396–400, 1981. doi: 10.1002/j.1552-4604.1981.tb01739.x.

[105] M. C. Chellingsworth, S. Laugher, S. Akhlaghi, D. B. Jack, and M. J. Kendall. The effects of ranitidine and cimetidine on the pharmacokinetics and pharmacodynamics of metoprolol. *Alimentary pharmacology & therapeutics*, 2(6): 521–7, 1988. doi: 10.1111/j.1365-2036.1988.tb00726.x.

[106] S. Toon, E. M. Davidson, F. M. Garstang, H. Batra, R. J. Bowes, and M. Rowland. The racemic metoprolol H2-antagonist interaction. *Clinical pharmacology and therapeutics*, 43(3):283–9, 1988. doi: 10.1038/clpt.1988.34.

[107] W. Kirch, H. Spahn, H. Köhler, E. E. Ohnhaus, and E. Mutschler. Interaction of metoprolol, propranolol and atenolol with concurrent administration of cimetidine. *Klinische Wochenschrift*, 60(22):1401–7, 1982. doi: 10.1007/BF01716245.

[108] S. M. Stout, J. Nielsen, L. S. Welage, M. Shea, R. Brook, K. Kerber, and B. E. Bleske. Influence of metoprolol dosage release formulation on the pharmacokinetic drug interaction with paroxetine. *Journal of clinical pharmacology*, 51(3):389–96, 2011. doi: 10.1177/0091270010365559.

[109] R. B. Parker and J. E. Soberman. Effects of paroxetine on the pharmacokinetics and pharmacodynamics of immediate-release and extended-release metoprolol. *Pharmacotherapy*, 31(7):630–41, 2011. doi: 10.1592/phco.31.7.630.

[110] A. Hemeryck, R. A. Lefebvre, C. De Vriendt, and F. M. Belpaire. Paroxetine affects metoprolol pharmacokinetics and pharmacodynamics in healthy volunteers. *Clinical pharmacology and therapeutics*, 67(3):283–91, 2000. doi: 10.1067/mcp.2000.104788.

[111] J. A. Johnson and B. S. Burlew. Metoprolol metabolism via cytochrome P4502D6 in ethnic populations. *Drug metabolism and disposition: the biological fate of chemicals*, 24(3):350–5, 1996.

[112] P. N. Bennett, V. A. John, and V. B. Whitmarsh. Effect of rifampicin on metoprolol and antipyrine kinetics. *British journal of clinical pharmacology*, 13(3):387–91, 1982. doi: 10.1111/j.1365-2125.1982.tb01390.x.

[113] Open Systems Pharmacology. Japanese Population Report, 2019. URL https://github.com/Open-Systems-Pharmacology/OSPSuite.Documentation/blob/master/Japanese_Population/Report.md.

[114] N. Yasui-Furukori, M. Saito, T. Niioka, Y. Inoue, Y. Sato, and S. Kaneko. Effect of itraconazole on pharmacokinetics of paroxetine: the role of gut transporters. *Therapeutic drug monitoring*, 29(1):45–8, 2007. doi: 10.1097/FTD.0b013e31802bb20d.

[115] A.-H. Andreasen, K. Brøsen, and P. Damkier. A Comparative Pharmacokinetic Study in Healthy Volunteers of the Effect of Carbamazepine and Oxcarbazepine on Cyp3a4. *Epilepsia*, 48(3):490–496, 2007. doi: 10.1111/j.1528-1167.2007.00924.x.

[116] K. W. Kolb, W. R. Garnett, R. E. Small, G. W. Vetrovec, B. J. Kline, and T. Fox. Effect of Cimetidine on Quinidine Clearance. *Therapeutic Drug Monitoring*, 6(3):306–312, 1984. doi: 10.1097/00007691-198409000-00009.

[117] B. G. Hardy and J. J. Schentag. Lack of effect of cimetidine on the metabolism of quinidine: effect on renal clearance. *International journal of clinical pharmacology, therapy, and toxicology*, 26(8):388–91, 1983.

[118] P. Damkier, L. L. Hansen, and K. Brøsen. Effect of diclofenac, disulfiram, itraconazole, grapefruit juice and erythromycin on the pharmacokinetics of quinidine. *British Journal of Clinical Pharmacology*, 48(6):829–838, 1999. doi: 10.1046/j.1365-2125.1999.00099.x.

[119] P. Damkier, L. L. Hansen, and K. Brøsen. Rifampicin treatment greatly increases the apparent oral clearance of quinidine. *Pharmacology and Toxicology*, 85(6):257–262, 1999. doi: 10.1111/j.1600-0773.1999.tb02019.x.

[120] K. M. Kaukonen, K. T. Olkkola, and P. J. Neuvonen. Itraconazole increases plasma concentrations of quinidine. *Clinical pharmacology and therapeutics*, 62(5):510–7, 1997. doi: 10.1016/S0009-9236(97)90046-1.

[121] M. S. Ching, S. L. Elliott, C. K. Stead, R. T. Murdoch, S. Devenish-Meares, D. J. Morgan, and R. A. Smallwood. Quinidine single dose pharmacokinetics and pharmacodynamics are unaltered by omeprazole. *Alimentary pharmacology & therapeutics*, 5(5):523–31, 1991. doi: 10.1111/j.1365-2036.1991.tb00521.x.

[122] P. Damkier, L. L. Hansen, and K. Brøsen. Effect of fluvoxamine on the pharmacokinetics of quinidine. *European Journal of Clinical Pharmacology*, 55(6):451–456, 1999. doi: 10.1007/s002280050655.

[123] D. J. Edwards, R. Lavoie, H. Beckman, R. Blevins, and M. Rubenfire. The effect of coadministration of verapamil on the pharmacokinetics and metabolism of quinidine. *Clinical Pharmacology and Therapeutics*, 41(1):68–73, 1987. doi: 10.1038/clpt.1987.11.

[124] W. Mahatthanatrakul, S. Sriwiriyajan, W. Ridtitid, J. Boonleang, M. Wongnawa, N. Rujimamahasan, and W. Pipatrattanaseree. Effect of cytochrome P450 3A4 inhibitor ketoconazole on risperidone pharmacokinetics in healthy volunteers. *Journal of clinical pharmacy and therapeutics*, 37(2):221–5, 2012. doi: 10.1111/j.1365-2710.2011.01271.x.

[125] K.-A. Kim, P.-W. Park, K.-H. Liu, K.-B. Kim, H.-J. Lee, J.-G. Shin, and J.-Y. Park. Effect of rifampin, an inducer of CYP3A and P-glycoprotein, on the pharmacokinetics of risperidone. *Journal of clinical pharmacology*, 48(1): 66–72, 2008. doi: 10.1177/0091270007309888.

[126] W. Mahatthanatrakul, T. Nontaput, W. Ridtitid, M. Wongnawa, and M. Sunbhanich. Rifampin, a cytochrome P450 3A inducer, decreases plasma concentrations of antipsychotic risperidone in healthy volunteers. *Journal of clinical pharmacy and therapeutics*, 32(2):161–7, 2007. doi: 10.1111/j.1365-2710.2007.00811.x.

[127] T. Nakagami, N. Yasui-Furukori, M. Saito, T. Tateishi, and S. Kaneo. Effect of verapamil on pharmacokinetics and pharmacodynamics of risperidone: in vivo evidence of involvement of P-glycoprotein in risperidone disposition. *Clinical pharmacology and therapeutics*, 78(1):43–51, 2005. doi: 10.1016/j.clpt.2005.03.009.

[128] FDA. Medication guide STRATTERA® (atomoxetine hydrochloride), 2007. URL https://www.accessdata.fda.gov/drugsatfda_docs/label/2007/021411s004s012s013s015s021lbl.pdf.

[129] FDA. Highlights of prescribing information LOPRESSOR (metoprolol tartrate), 2023. URL https://www.accessdata.fda.gov/drugsatfda_docs/label/2023/017963s074lbl.pdf.

[130] FDA. Outer packaging TAGAMET® HB (cimetidine), 2019. URL https://www.accessdata.fda.gov/drugsatfda_docs/label/2020/020238Orig1s024lbl.pdf.

[131] FDA. Medication guide SPORANOX® (itraconazole), 2018. URL https://www.accessdata.fda.gov/drugsatfda_docs/label/2018/020083s062lbl.pdf.

[132] FDA. Highlights of prescribing information PAXIL (paroxetine), 2021. URL https://www.accessdata.fda.gov/drugsatfda_docs/label/2021/020031s077lbl.pdf.

[133] National Library of Medicine. Drug label information quinidine sulfate, 2023. URL https://dailymed.nlm.nih.gov/dailymed/drugInfo.cfm?setid=b0d6341b-d880-4f0b-8844-bc788c03004a.
